# Horizontal Transposon Transfer and Their Ecological Drivers: The Case of Flower-breeding *Drosophila*

**DOI:** 10.1093/gbe/evad068

**Published:** 2023-04-26

**Authors:** Tuane L Carvalho, Juliana Cordeiro, Jeferson Vizentin-Bugoni, Pedro M Fonseca, Elgion L S Loreto, Lizandra J Robe

**Affiliations:** Programa de Pós-Graduação em Biodiversidade Animal - PPGBA, Universidade Federal de Santa Maria—UFSM, Santa Maria, Rio Grande do Sul, Brazil; Departamento de Ecologia, Zoologia e Genética, Instituto de Biologia, Universidade Federal de Pelotas – UFPel, Pelotas, RS, Brazil; Departamento de Ecologia, Zoologia e Genética, Instituto de Biologia, Universidade Federal de Pelotas – UFPel, Pelotas, RS, Brazil; Programa de Pós-Graduação em Biodiversidade Animal - PPGBA, Universidade Federal de Santa Maria—UFSM, Santa Maria, Rio Grande do Sul, Brazil; Programa de Pós-Graduação em Genética e Biologia Molecular, Universidade Federal do Rio Grande do Sul—UFRGS, Porto Alegre, Rio Grande do Sul, Brazil; Programa de Pós-Graduação em Biodiversidade Animal - PPGBA, Universidade Federal de Santa Maria—UFSM, Santa Maria, Rio Grande do Sul, Brazil; Programa de Pós-Graduação em Biodiversidade Animal - PPGBA, Universidade Federal de Santa Maria—UFSM, Santa Maria, Rio Grande do Sul, Brazil

**Keywords:** anthophilic drosophilids, horizontal transfer, molecular adaptation, specialization gradient, transposable element

## Abstract

Understanding the mechanisms that shape the architecture, diversity, and adaptations of genomes and their ecological and genetic interfaces is of utmost importance to understand biological evolution. Transposable elements (TEs) play an important role in genome evolution, due to their ability to transpose within and between genomes, providing sites of nonallelic recombination. Here we investigate patterns and processes of TE-driven genome evolution associated with niche diversification. Specifically, we compared TE content, TE landscapes, and frequency of horizontal transposon transfers (HTTs) across genomes of flower-breeding *Drosophila* (FBD) with different levels of specialization on flowers. Further, we investigated whether niche breadth and ecological and geographical overlaps are associated with a potential for HTT rates. Landscape analysis evidenced a general phylogenetic pattern, in which species of the *D. bromeliae* group presented L-shaped curves, indicating recent transposition bursts, whereas *D. lutzii* showed a bimodal pattern. The great frequency of highly similar sequences recovered for all FBD suggests that these species probably experienced similar ecological pressures and evolutionary histories that contributed to the diversification of their mobilomes. Likewise, the richness of TEs superfamilies also appears to be associated with ecological traits. Furthermore, the two more widespread species, the specialist *D. incompta* and the generalist *D. lutzii*, presented the highest frequency of HTT events. Our analyses also revealed that HTT opportunities are positively influenced by abiotic niche overlap but are not associated with phylogenetic relationships or niche breadth. This suggests the existence of intermediate vectors promoting HTTs between species that do not necessarily present overlapping biotic niches.

**Significance Statement**

Transposable elements (TE) are a dynamic component of genomes and may be transferred between species in non-sexual means, through mechanisms of horizontal transfer (HT). This study employs flower-breeding *Drosophila* (FBD) species with different levels of specialization to show that, despite biotic niche breadth and biotic niche overlap, larger distribution ranges and abiotic niche overlap provide the greatest opportunities to HT. Thus, our careful characterization of the mobilome, the phylogeny, and the ecology of a specific set of species supports the idea that intermediate vectors connect species presenting nonoverlapping biotic niches, helping to transfer TEs even among distantly related species.

## Introduction

Environmental variation shape genome evolution, leading to differences in genetic composition (Franks and Hoffmann 2012; Rey et al. 2016; Schrader and Schmitz 2019). These determine morphological and physiological characteristics that enable adaptation to environmental changes and filling of novel niches (Aminetzach et al. 2005; González et al. 2010; Piacentini et al. 2014; Fanti et al. 2017; Layton and Bradbury 2021). Due to their ability to transpose within and even between genomes, transposable elements (TEs) play an important role in genome evolution (Biemont and Vieira 2006; Oliver and Greene 2009). According to their mechanism of transposition, TEs can be divided into two major classes: Class I, the retrotransposons, which use RNA as a transposition intermediate; and Class II, the transposons, which transpose via a DNA intermediate (Wicker et al. 2007). Within each of these classes, TEs can be additionally subdivided into autonomous elements, which encode the proteins necessary for transposition; and, non-autonomous elements, which use the proteins provided by other closely related TEs (Platt et al. 2016). The entire set of TEs within a single genome is called mobilome, whose diversity and dynamics can be interpreted from a landscape presenting the abundance (frequency distribution) and age (genetic distance) of the TE families/superfamilies (Fonseca et al. 2019; Petersen et al. 2019).

During mobilization, excision and/or insertion events produce breakpoints and genome rearrangements, making TEs an extensive source of mutations and genetic polymorphisms (Böhne et al. 2008; Schnable et al. 2009). Furthermore, even after silencing, TEs can be targets of nonallelic recombination, another important source of genomic rearrangements (Oliver and Greene 2009; Bourque et al. 2018; Almojil et al. 2021). In fact, many studies have reported negative consequences of TEs on the genomes of their hosts, especially regarding human diseases (Lee et al. 2012; Hancks and Kazazian 2016). Nonetheless, TEs also have positive impacts on the evolution of eukaryotic genomes (Feschotte and Pritham 2007; Cordaux and Batzer 2009), influencing the adaptation of the host, its response to stress, behavior, and even development (Franchini et al. 2011; Rostant et al. 2012; Woronik et al. 2019). In fact, mobilization can be enhanced, and silencing may be reversed during stressful conditions, which are usually associated with a higher frequency of mutations induced by TEs, evidencing the role of these mobile elements in adaptive phenotypic and genotypic responses to changes in the environment (McClintock 1984; Capy et al. 2000; Stoebel and Dorman 2010; Casacuberta and González 2013).

TE invasion of new genomes occurs by horizontal transposon transfers (HTTs) between distinct species, especially those with spatiotemporal overlap (Wallau et al. 2012; Zhang et al. 2020). In this scenario, HTT among species can be described as complex networks of shared TEs shaped by ecological interactions, in which a species may interact with another either directly or indirectly, via an intermediate species (Gilbert and Feschotte 2018). Such HTT events provide a new set of autonomous TEs until their domestication, silencing, or degradation and loss (Ortiz et al. 2015). Thus, demographic events such as host colonization history and niche expansion can lead to new ecological interactions or even intensify previous interactions between species that belong to the same guild in the community, leaving signals in each species mobilome (Chalopin et al. 2015; Petersen et al. 2019), by driving mobilization or providing new opportunities for HTTs. However, the relationships between niche diversification and TEs content and dynamics remains poorly understood, limiting our understanding of genomic evolution. Specifically, there is a lack of studies evaluating the role of biotic interactions and environmental factors on the HTT rate of TEs. This gap is due to the challenge of combining and testing the effect of distinct ecological traits on the potential for HTT as well as to the lack of comprehensive approaches including multiple factors.

**Fig. 1:**
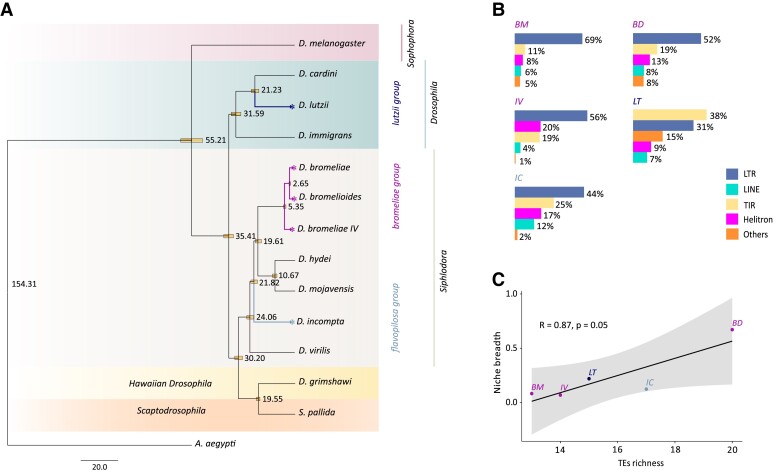
(*A*) Species tree reconstructed in Astral based on nucleotide sequences of 30 nuclear genes, with divergence times estimated in MCMCTree of PAML using fossil calibration to the root age of the divergence between Brachycera and Nematocera. (*B*) The proportion of the mobilome of each evaluated FBD occupied by different TE Orders, as obtained by homology-based searches. (*C*) Pearson correlation between TE richness and niche breadth for the five evaluated FDB species. BM, *D. bromeliae*; BD, D*. bromelioides*; IV, D*. bromeliae* sp. IV; IC, D*. incompta*; LT, *D. lutzii*.

Drosophilidae comprises one of the most diverse families of Diptera, encompassing species distributed in nearly all biogeographic regions that are adapted to a high diversity of environments (Throckmorton 1975). Ecological studies using Drosophilidae species as a model system often focuses on morphologically similar species that may also share ecological similarities (e.g., Cordeiro et al. 2020). The *Drosophila bromeliae*, *D. flavopilosa*, and *D. lutzii* species groups encompass the most representative FBD across the Neotropics (Robe et al. 2013; Schmitz and Valente 2019). These species groups use a wide range of host plants as sites for oviposition and larval development and may use the same flower as feeding, mating, and breeding sites, frequently presenting sympatric and even syntopic occurrences (Schmitz and Hofmann 2005; Schmitz and Valente 2019). Taxonomically, they are all included in non-*Sophophora* subgenera of *Drosophila*, like *Drosophila* (as for the *D. lutzii* group) or *Siphlodora* (as for the *D. bromeliae* and *D. flavopilosa* groups) (Yassin 2013; Suvorov et al. 2022) (fig. 1*A*). Nevertheless, whereas species of the *D. lutzii* group are usually generalists, using flowers of different families, species of the *D. bromeliae* group have a gradient of niche breadth, ranging from more generalist species, such as *D. bromeliae*, *D. bromelioides*, *D. bromeliae* sp. morphotypes III and III″, to specialist species, which use flowers of *Solanum*, such as *D. bromeliae* sp. morphotypes IV, IV″, V, and VI (Cordeiro et al. 2020). Species of the *D. flavopilosa* group, in turn, only interact with flowers of *Cestrum* (Brncic et al. 1983). Such a well-characterized variation in resource specialization makes these groups an ideal model system to explore the poorly understood relationship between niche diversification and genomic adaptations, including the roles of TEs interchange and mobilization on evolution.

Here we investigated the mobilomes of FBD belonging to the *D. bromeliae*, *D. flavopilosa,* and *D. lutzii* species groups, aiming to advance our understanding of the patterns and processes of genomic evolution associated with TE diversification. To evaluate the association between TEs and species specialization (niche breadth), we analyzed the mobilome composition and its associated landscape, as well as differences in the frequency of HTT in FDB species with varying degrees of specialization. Our hypothesis is that mobilome composition, landscape, and frequency of HTT events vary according to the ecological traits of each species. Thus, generalist and/or FBD species with broad geographic distribution, due to their interaction with more resources or contact with more species, would likely have more opportunities for HTT occurrence than specialist and/or species with restricted geographic distribution. This would be reflected in the mobilome landscape of species. We also expect that HTT rates among FBD are correlated with niche breadth, and with abiotic, biotic, and geographic overlap, providing means for ecological interactions that enable HTT events.

## Results

### Draft Genomes Quality

We generated de novo genome assemblies for *D. bromelioides* and *D. bromeliae* sp. morphotype IV, from the *bromeliae* species group, and for *D. lutzii*, from the *lutzii* group, using SPAdes (Bankevich et al. 2012). The contig and N50 lengths reported by QUAST v5 (Mikheenko et al. 2018) for each of our assemblies are reported in supplementary table S4, Supplementary Material online. The BUSCO (Simão et al. 2015) completeness index further highlighted the quality of the obtained assemblies, recovering complete sequences for at least 96.1% of the target Diptera genes, in addition to low values of missing data (supplementary table S4, Supplementary Material online). For *D. bromeliae*, the assembly previously obtained by Kim et al. (2021) was used for mobilome characterization. Conversely, for *D. incompta*, whose mobilome was already described by Fonseca et al. (2019), the set of TEs previously characterized was directly employed for HTT assessments.

### Mobilome

We first performed a de novo approach, in which raw filtered Illumina reads were filtered, clustered, and then related to individual repeat superfamilies using RepeatExplorer 2.0 pipeline. According to this strategy, TEs genome content ranged from 5.75% (for *D. lutzii*) to 20.38% (for *D. bromelioides*) (table 1). For the homology-based approaches performed with the assembled genomes, using RMBlast 2.10.0 against a curated TE database (Repbase v. 20181026 database), a substantial difference was detected in the TE content for all species. The greater difference was encountered for *D. bromelioides* and *D. bromeliae* sp. morphotype IV, where values dropped to approximately 9.73% to 9.11%, respectively, of the genomes (table 1).

**Table 1:** Summary Describing the Main Characteristics of the Mobilome of Each FBD Species

Drosophila Species	% of the Genome Occupied by TEs		Absolute Number of TEs	Number of TEs Superfamilies	Maximum and Minimum TEs Sequence Length	Number of HTT
	RepeatExplorer^a^	RMBlast				
*D. bromeliae*	7.84	11.27	628	13	131–6457pb	22
*D. bromelioides*	20.38	9.73	770	20	130–6458pb	84
*D. bromeliae* sp. IV	15.14	9.11	563	14	131–5765pb	88
*D. lutzii*	4.40	5.75	1077	15	130–5763pb	101
*D. incompta*	13.00^b^	12.98^b^	596^b^	17^b^	105–8029pb^b^	123

Values for genome content were obtained through different methodologies, involving *de novo* (as performed in repeatexplorer) and *homology-based* (as performed with rmblast against repbase v. 20181026 database) searches.

a Values recovered after trimming DNA satellites and other families of repeated sequences.

b Values reported by Fonseca et al. (2019).

After all homology-based searches using RepBase TEs and species-specific customized TE libraries against the final database of RepeatExplorer 2.0 or against the draft genome of each FBD species, 628 TEs were recovered for *D. bromeliae*, 770 for *D. bromelioides*, 563 for *D. bromeliae* sp. morphotype IV, and 1,077 for *D. lutzii* (table 1; https://doi.org/10.5061/dryad.qz612jmjs). These TEs ranged in length from 130 bp to 6,558 bp (table 1). In general, this pattern suggests a lower abundance of TEs in ecologically and geographically restricted species, in contrast to the greater abundance of TEs found in more generalist and broadly distributed species. Nevertheless, when correlation tests were performed with all five FBD species (including the mobilome of *D. incompta* obtained by Fonseca et al. (2019), no significant association was found between the abundance of TEs and species niche breadth (Spearman correlation = 0.8, *P* = 0.13) or distribution area (Spearman correlation = 0.6, *P* = 0.35).

In all cases, LTRs (Class I—retrotransposons) and TIRs (Class II—Subclass 1—DNA transposons) were among the two most frequent TE orders, followed by Helitrons (Class II—Subclass 2—DNA transposons) (fig. 1*B*; table 2). Nevertheless, although LTRs encompass most of the identified TE sequences for the three species of the *D. bromeliae* group, TIRs encompass the most frequent TE order for *D. lutzii* (fig. 1*B*; table 2). Regarding TE richness, the number of superfamilies varied from 13 (for *D. bromeliae*) to 20 (for *D. bromelioides*) (table 1). Interestingly, when all five FBD species were taken into account, a strong and significant correlation could be detected between TEs richness and species niche breadth (Pearson correlation = 0.85, *P* = 0.05) (fig. 1C) but not between TEs richness and distribution area (Pearson correlation = 0.33, *P* = 0.59).

**Table 2:** Summary of the Mobilome Content Recovered for Each FBD Considering the Subdivision Among TE Class, Subclass, Order, and Superfamily, as Obtained Through the Combined Results of Both *de Novo* and *Homology-Based* Searches

Transposable Element		*Drosophila* Species											
Order	Superfamily	*D. bromeliae*			*D. bromelioides*			*D.bromeliae sp. IV*			*D. lutzii*		
		TS	PS	TO	TS	PS	TO	TS	PS	TO	TS	PS	TO
Class I (retrotransposons)													
LTR	Copia	77	0.123	0.693	57	0.074	0.522	47	0.083	0.558	35	0.032	0.309
	Gypsy	278	0.443		295	0.383		195	0.346		255	0.237	
	BEL	80	0.127		50	0.065		71	0.126		43	0.040	
	Ginger	0	0.000		0	0.000		1	0.002		0	0.000	
LINE	R1	15	0.024	0.064	26	0.034	0.083	15	0.027	0.044	40	0.037	0.071
	RTE	9	0.014		17	0.022		5	0.009		2	0.002	
	Jockey	12	0.019		14	0.018		3	0.005		22	0.020	
	I	4	0.006		7	0.009		2	0.004		13	0.012	
SINE	MeloSINE	0	0.000	0.000	2	0.003	0.004	0	0.000	0.000	0	0.000	0.000
	TS2	0	0.000		1	0.001		0	0.000		0	0.000	
Class II (DNA transposons)—subclass I													
TIR	Mariner	29	0.046	0.107	59	0.077	0.186	73	0.130	0.199	295	0.274	0.376
	hAT	12	0.019		27	0.035		23	0.041		51	0.047	
	Mutator	0	0.000		1	0.001		0	0.000		0	0.000	
	Transib	14	0.022		26	0.034		0	0.000		48	0.045	
	P	0	0.000		5	0.006		0	0.000		4	0.004	
	PiggyBac	0	0.000		7	0.009		11	0.020		6	0.006	
	Harbinger	12	0.019		16	0.021		5	0.009		1	0.001	
	CACTA	0	0.000		2	0.003		0	0.000		0	0.000	
Crypton	Crypton	0	0.000	0.000	1	0.001	0.001	0	0.000	0.000	0	0.000	0.000
Class II (DNA transposons)—subclass II													
Helitron	Helitron	52	0.083	0.083	99	0.129	0.129	108	0.192	0.192	99	0.092	0.092
Maverick	Polinton	1	0.002	0.002	1	0.001	0.001	3	0.005	0.005	1	0.001	0.001
Unknown	MINIME	33	0.053	0.053	57	0.074	0.074	1	0.002	0.002	162	0.150	0.150
Total		628	1	1	770	1	1	563	1	1	1077	1	1

TS, total per subfamily; PS, percent per subfamily; TO, total per order.

Gypsy, BELPao, and Copia superfamilies were the most abundant LTR element in all FBD species (table 2). For long interspersed nuclear elements (LINEs), R1, non-LTR transposable element (RTE), and Jockey were the most abundant superfamilies in the four genomes (table 2). Mariner and hAT were commonly recovered as the most abundant Class II—Subclass 1 TE, whereas Helitron was the most common TE from Class II—Subclass 2 (table 2). Interestingly, the unclassified MINIME and the TIR Transib corresponded to a significant proportion of the mobilome of all generalist species, but were rarely or not at all detected for the specialist *D. bromeliae* sp. morphotype IV (table 2). Among the rarest TEs, short interspersed elements (SINEs) were only detected in *D. bromelioides* (table 2).

**Fig. 2:**
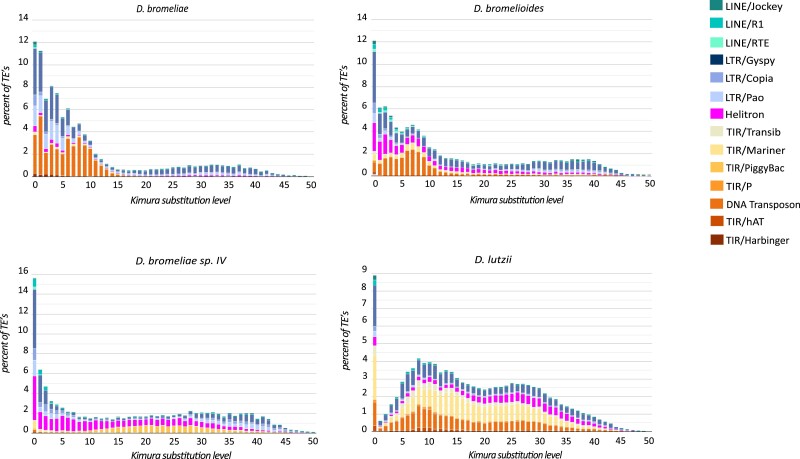
Characterization of the mobilome according to TEs landscapes, reflecting the dynamics of different superfamilies of TEs. The graphic depicts the relative frequency (*y* axis) of different values of Kimura two-parameters distances (*x* axis) between each TE and the consensus sequences of each superfamily, as obtained from the customized species-specific database.

The TEs landscapes reconstructed on RepeatMasker 4.1.2 (http://www.repeatmasker.org) using the draft genome of each species as input against the customized species-specific TE library, characterized after trimming the results obtained by the complementary de novo and homology-based search strategies were essentially characterized by an L-shape in all species of the *D. bromeliae* species group (fig. 2). This pattern suggests a recent transposition burst, with predominance of young active copies. *Drosophila bromeliae* and *D. bromelioides* presented signals of transposition bursts for LTRs and TIRs (and Helitrons, for the second species), with a high predominance of elements presenting distance values lower than 10%. Conversely, for *D. bromeliae* sp. morphotype IV, transposition bursts were detected mostly for LTRs and Helitrons, which usually presented distances smaller than 5% (fig. 2). Nevertheless, the most differential patterns were detected in the TEs landscape of *D. lutzii*, which showed a bimodal pattern, with intercalated events of excision and transposition involving LTRs, TIRs, and Helitrons. Even so, in agreement with the three species of the *D. bromeliae* group, a very recent and isolated transposition burst for similar TEs superfamilies is also observed in *D. lutzii* (fig. 2).

### HT

HT analysis among FBD performed on vertical and horizontal inheritance consistence analysis (VHICA) (Wallau et al. 2015) recovered 247 pairwise comparisons in which dS values were significantly lower than those presented by nuclear genes with similar codon usage bias (CUB) values (supplementary table S5, Supplementary Material online). As a single HTT event could result in more than one significant pairwise comparison involving, for example, a transfer to an ancestral species or from one of two closely related species to another, more distantly related (Walau et al. 2012) a careful evaluation was performed to estimate the putative number of HTT events (see Material and Methods—HT analysis). This evidenced the occurrence of 209 HTTs: 22 for *D. bromeliae*, 84 for *D. bromelioides*, 88 for *D. bromeliae* sp. morphotype IV, 123 for *D. incompta*, and 101 for *D. lutzii* (table 1; supplementary table S6, Supplementary Material online). Among these, 121, 46, and 30 events involved LTRs, TIRs, and Helitrons, respectively (table 3).

**Table 3:** Number of TE Superfamilies Involved in HTT Events in Each Pairwise Comparison Involving Different FBD Species

Transposable Element		HTT Event Between FBD Species										
Order	Superfamily	BMxBD	BMxIV	BMxIC	BMxLT	BDxIV	BDxIC	BDxLT	IVxIC	IVxLT	ICxLT	Total
Class I (retrotransposons)												
LTR	BEL	0	3	0	2	4	0	2	0	3	1	15
	Copia	1	3	0	1	5	3	5	2	0	2	22
	Gypsy	0	5	2	1	16	4	9	11	2	34	84
LINE	R1	0	0	0	0	0	0	0	0	0	2	2
	RTE	0	0	0	0	1	0	0	0	0	0	1
	Jockey	0	0	1	0	0	1	3	0	0	0	5
	I2	0	0	0	0	0	1	1	0	0	1	3
Class II (DNA transposons)—subclass I												
TIR	Mariner	0	0	0	0	1	4	0	5	2	4	16
	hAT	0	0	0	0	0	1	1	3	0	4	9
	Transib	0	0	0	1	0	2	0	0	0	4	7
	PiggyBAC	0	0	0	0	0	3	1	3	0	2	9
	Harbinger	0	0	0	0	0	1	1	2	0	1	5
Class II (DNA transposons)—subclass II												
Helitron	Helitron	0	0	1	0	7	5	3	7	2	5	30
Maverick	Polinton	0	0	0	0	0	0	0	0	0	1	1
Total		1	11	4	5	34	25	26	33	9	61	209

BM, *D. Bromeliae*; BD, *D. Bromelioides*; IV, *D. Bromeliae* sp. IV; IC, *D. Incompta*; LT, *D. Lutzii.*

**Fig. 3:**
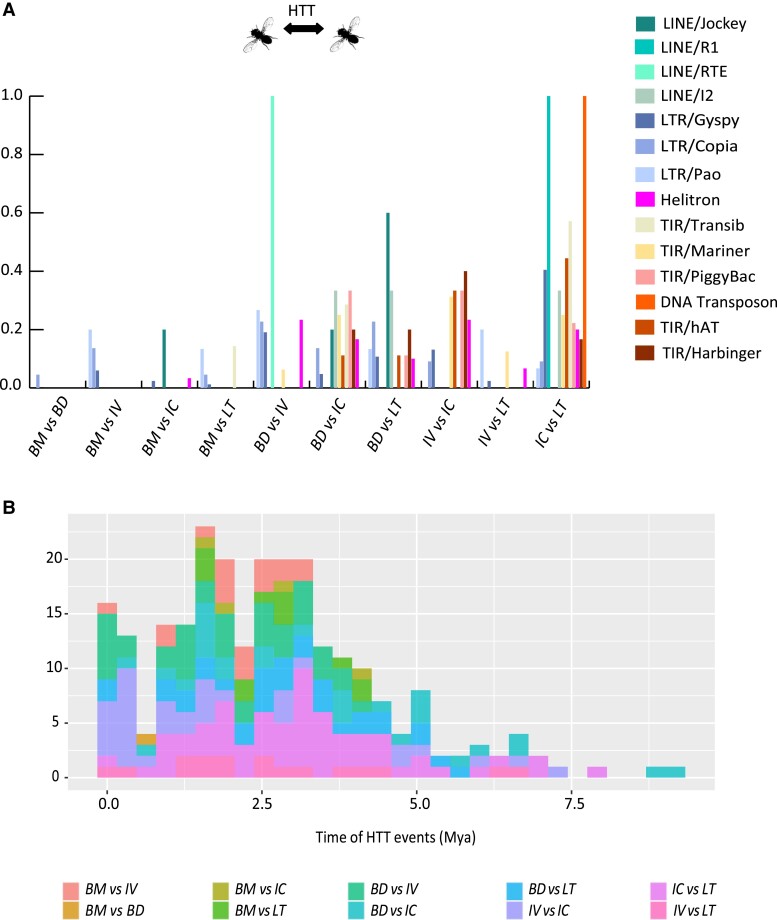
(*A*) Percentage of the total HTTs recovered for each TE subclass involving different pairs of FBD species. (*B*) Time distribution of the HTT events involving different pairs of FBD species. BM, D*. bromeliae*; BD, D*. bromelioides*; IV, D*. bromeliae* sp. IV; IC, D*. incompta*; LT, D*. lutzii*.

Among HTT events, 61 occurred between *D. incompta* and *D. lutzii* and involved 12 superfamilies, representing 29.18% of the detections (table 3, fig. 3). Additionally, 34 (16.27%) events (six superfamilies) were detected between *D. bromelioides* and *D. bromeliae* sp. morphotype IV, and 33 (15.79%) (seven superfamilies) between *D. bromeliae* sp. morphotype IV and *D. incompta*. Interestingly, *D. incompta* and *D. lutzii* were the species with the higher richness of superfamilies involved in HTT events for most pairwise comparisons, except between *D. bromeliae* sp. morphotype IV and *D. lutzii* (table 3, fig. 3*A*).

Most HTT events of LTRs retrotransposons (Class I) and TIRs DNA transposons (Class II—Subclass I) involved *D. incompta* and *D. lutzii* (fig. 3*A*). For LINEs retrotransposons, most HTT events involved *D. incompta*, *D. bromelioides* and *D. lutzii.* Conversely, HTTs for Helitron and Maverick (Class II—Subclass II) were more evenly distributed among *D. bromelioides*, *D. bromeliae* sp. morphotype IV, *D. incompta*, and *D. lutzii*, but only rarely involved *D. bromeliae* (fig. 3*A*).

Although the search for HTT events was only performed in the comparisons among FBD species, some suggestive cases were also observed from homology-based searches against TEs from different species using RepBase database (supplementary table S4, Supplementary Material online). This is the case of the following sequences:

1) Class II—Subclass 1: a fragment of 1,298 and 855 bp of *D. bromelioides* and *D. bromeliae* sp. morphotype IV, respectively, presented at least 98% of similarity with a Mariner sequence of *Schmidtea mediterranea* (Platyhelminthes).
2) Class II DNA transposons—Subclass 2: *D. bromelioides*, *D. bromeliae* sp. morphotype IV and *D. lutzii* presented a fragment of 2,565, 2,565, and 951 bp, respectively, with at least 97% of similarity with a Helitron of *Bombyx mori* (domestic silkmoth from the Bombycidae family); *D. bromelioides* presented a fragment of 1,010 bp with 90% of similarity with a Helitron of *Rhodnius prolixus* (an insect vector of the Chagas Disease from the Triatominae subfamily); and *D. bromeliae* sp. morphotype IV presented a fragment of 850 bp with 94% of similarity with a Helitron of *Heliconius melpomene melpomene* (common postman butterfly from the Nymphalidae family).

When divergence times of HTTs were estimated using the formula *T* = *k*/2*r* (Graur and Li, 2000) and a synonymous substitution rate measured for nuclear gens of *Drosophila* (*r* = 0.02022), it was found that almost all HTT events were dated to the last 7.5 Mya (fig. 3*B*). This suggests that most HTT events occurred after most of the target FDB species diverged from their most recent common ancestors (fig. 1*A*) to the exception of the speciation events involving the *D. bromeliae* species group.

### Predictors of Potential Effects in HT

**Fig. 4:**
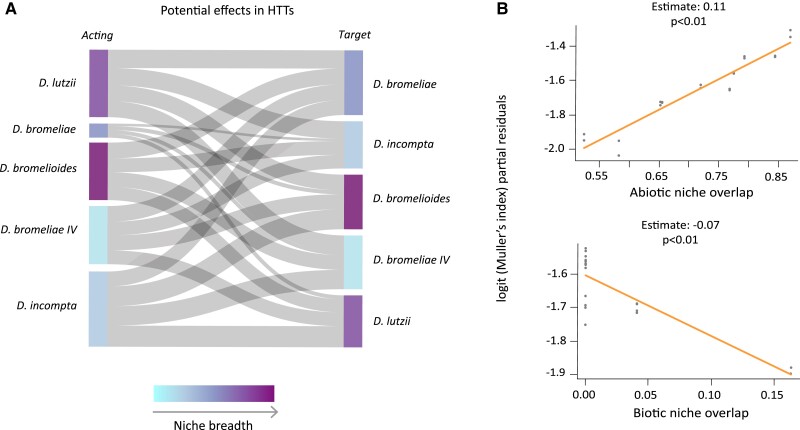
(*A*) Graph of potential effects on HTT rates among FBD, as measured by the Müller index. Bar thickness varies according to the Müller index, which ranges from 0.21–0.29, and 0.22–0.25, for the most influential species *D. lutzii* and *D. incompta*, respectively, from 0.18–0.22, and 0.19–0.22 for the intermediate *D. bromelioides* and *D. bromeliae* sp. IV, respectively, and from 0.03–0.05 for the least influential species, *D. bromeliae* (supplementary table S7, Supplementary material online). Niche breadth varies from the more specialized species (represented by lighter shades) to the more generalist species (represented by darker shades). (*B*) Relationships between the Müller index (response variable) and the two significant predictors: abiotic and biotic niche overlap. Gray dots represent each pair of FBD species, and the red line is the adjusted fitted trend.

As it was not possible to measure the direction of HTT events on VHICA, we evaluated the potential of each species (“acting”) to influence another (“target”) via HTT using the Müller index, as estimated by the potential for apparent competition (PAC) function of the bipartite R-package considering the distribution of the TEs and HTT events described above. According to this measure, *D. incompta* and *D. lutzii* are the most influential species in the set of recovered HTT events (Müller index = 0.21–0.29, and 0.22–0.25, respectively), whereas *D. bromeliae* was the most influenced species (Müller index = 0.035–0.054). Furthermore, *D. lutzii* was the species with the greatest estimated effect on *D. incompta*, and *D. incompta* was the species with the greatest estimated effect on *D. lutzii* (fig. 4*A*; supplementary table S7, Supplementary Material online).

To test whether these effects are significantly correlated to ecological, geographical, or evolutionary parameters, we employed Muller's index as the response variable in generalized linear mixed models. To this task, abiotic niche overlap, geographical overlap, biotic niche overlap, niche breadth, and phylogenetic relationships were estimated and employed as predictor variables (supplementary table S7, Supplementary Material online). The best-fit model revealed that abiotic niche overlap plays an important positive role in explaining the probability of one FBD species influencing another via HTT, whereas biotic niche overlap presents a negative association with the rate of TEs sharing by HTT (*β* = 0.11, *P* < 0.05, and *β* = –0.07, *P* < 0.05, respectively) (fig. 4*B*; supplementary tables S8 and S9, Supplementary Material online). Geographic overlap, niche breadth, and phylogenetic signals were not significantly associated with potential effects.

## Discussion

### Mobilome

The abundance of transposable elements in eukaryotic genomes and their importance for genomic evolution has been increasingly recognized (Laciano and Cristofari 2020; Senft and Macfarlan 2021; Frith 2022). In insects, the extent of TEs can range from ∼1% in *Belgica antarctica* (Diptera), to 60% in the migratory locust *Locusta migratoria* (Orthoptera) (Petersen et al. 2019). In *Drosophila*, percentage of TEs is known to range from 4.65% in *D. busckii* to 40% in *D. ananassae* (Sessegolo et al. 2016; Petersen et al. 2019). These values are within the range of variation reported here, in which TEs encompassed between 5.75% and 20.38% of the evaluated genomes (as recovered for *D. lutzii* and *D. bromelioides*, respectively). Nevertheless, it is important to note that the values reported for the de novo and *homology-based* searches for *D. bromelioides* and *D. bromeliae* sp. morphotype IV presented a substantial difference. Similar discrepancies involving TE content estimations obtained with different methodologies were also observed for the mobilome of some mosquito species (De Melo and Wallau 2020) and can be ascribed not only to differences in the sensitivity of both approaches but also to differences in the estimations of the total genome length. In this sense, this study reinforces the idea that a combined approach involving different strategies is recommended in studies aimed to characterize the mobilome of a species.

Considering the abundance and diversity of TEs in the final mobilomes characterized here, the values varied among species. Overall, the most generalist FBD species (*D. bromelioides*) and the most widely distributed FBD species (*D. lutzii*), presented the highest TE richness and abundance, respectively, whereas *D. bromeliae* sp. morphotype IV, the most specialized and geographically restricted FBD species, presented some of the lowest values. Such findings seem to support the hypothesis that species with broader distributions or niche breadths may have a higher extent and diversity of TEs, due to their wider or richer interaction networks, enabling more opportunities for TEs diversification. Nevertheless, a statistically significant positive correlation was only found between TEs diversity and species niche breadth, suggesting that geographical and ecological properties may have lower associations with the abundance of TEs. However, it remains to be tested whether this result holds with the inclusion of more species, as the lack of significant correlation in some of our tests may be an outcome of reduced sample size.

Concerning the general mobilome content, all species displayed high levels of LTRs, TIR-Mariner, and Helitrons. Although LTR elements are usually less abundant in insects and even in some Diptera, they constitute a significant part of the mobilome of different *Drosophila* species (Nene et al. 2007; Osanai-Futahashi et al. 2008; Peccoud et al. 2017; Fonseca et al. 2019). Among LTR elements, Gypsy was recovered as the most abundant TE in all species of the *bromeliae* group and also in *D. incompta* (Fonseca et al. 2019). The LTR-Gypsy superfamily is widely distributed and conserved within *Drosophila*, especially within the *Sophophora* subgenus, indicating an old diversification associated with numerous recent HT events (Herédia et al. 2007; Loreto et al. 2008; Cordeiro et al. 2019). This study shows that it is also the most abundant TE in different FBD species of the *Siphlodora* subgenus. Conversely, a different pattern was detected here for the evaluated FBD of the *Drosophila* subgenus, *D. lutzii*, for which the most abundant TE was the TIR-Mariner from Class 2—Subclass 1—DNA transposons, as also shown for many genomes across Eukaryota (Dupeyron et al. 2020). This rank changes in the order of the most abundant TEs between the two groups of FBD species suggest a phylogenetic pattern among the target species, which should be additionally tested through the inclusion of different Drosophilidae species.

Mobilome landscapes reinforce the idea that phylogenetic and species-specific factors shape the mobilome in FDB groups analyzed here. In fact, whereas all species of the *D. bromeliae* group recovered landscapes with an L-shape pattern, *D. lutzii* showed bimodal curves. Even so, when considered in more detail, it is also possible to find some differences among landscapes within the *D. bromeliae* species group, as well as to find shared patterns between them and *D. lutzii.* In the first case, it is possible to detect that *D. bromeliae* morphotype IV not only presents a higher frequency of recent transpositions but also differs from the other two species of the group by the paucity of TIR elements involved in this burst. Conversely, *D. bromelioides* differs from *D. bromeliae* but resembles *D. bromeliae* morphotype IV by the influence of Helitrons in the recent transposition burst. Moreover, a recent transposition burst involving LTRs, TIRs, and Helitrons could also be detected for *D. lutzii*. These findings support our hypothesis that similar ecological traits and/or evolutionary pressures may determine the opportunities for HTT or diversification of mobilomes. Within this scenario, all FDB species possibly experienced new habitats/conditions or were subject to stressful conditions that lead to an increase in the rates of TE mobilization and invasion (Schrader et al. 2014; Stapley et al. 2015; Dennenmoser et al. 2017). It is also possible that recent range expansions lead to secondary contacts between several of these species, increasing the rates of TEs exchange. In fact, recent expansions were already detected by population-level genetic data for *D. lutzii* (Carvalho et al. 2023) and *D. incompta* (Moreira et al.—in preparation). Accordingly, almost all detected HTT events were dated to the last 7.5 Mya, and putatively occurred after divergence among the target species.

### Potential for HT Effects and Their Drivers

Although the HT is not infrequent, it appears to be related to phylogenetic, biogeographic, and ecological factors (Bartolomé et al. 2009; Kelley et al. 2014; Venner et al. 2017). In this sense, a geographic and ecological overlap is commonly advocated to be necessary, but not sufficient, to enable HTT (Gilbert et al. 2020). Moreover, considering that virus or prokaryote or eukaryote parasites are usually invoked as putative vectors for HTT (Gilbert et al. 2010; Suh et al. 2016; Metzger et al. 2018) and that most parasites usually present some level of phylogenetic specialization (Kuraku et al. 2012), it is also expected that phylogenetically close species that co-occur geographically under similar resources, present greater opportunities for HTT. Besides, as closely related species usually share transcription factors and other proteins that may be related to transposition mechanisms, transfer among such species might have been more successful (Bartolomé et al. 2009; Peccoud et al. 2017; De Melo and Wallau 2020).

However, in this study, we did not find a significant influence of phylogeny or niche breadth on patterns of HTT. In fact, only abiotic niche overlap was significantly and positively associated with the potential for HTT, with the generalist species *D. lutzii* and the specialist species *D. incompta* being the most influential species. Both species have a wide geographic distribution and broadly overlapping distribution between themselves and with other species of the *D. bromeliae* group, especially with *D. bromelioides* and *D. bromeliae* sp. morphotype IV. The greater influence on the potential for HTT detected here for the most widespread species corroborates the hypothesis that TEs are more likely to be exchanged if species are in direct or indirect contact, as suggested by many studies that report HTT between animal hosts and their parasites (Walsh et al. 2013; Gilbert et al. 2020). Interestingly, this seems to occur despite the frequency of syntopic co-occurrence, as our best-fit model revealed that biotic niche overlap does not play a positive role in explaining the probability of HTT. This highlights the potential of intermediate vectors to mediate and enhance opportunities for HTT by linking species that co-occur in the same area but do not necessarily use the same resources. Another interesting pattern is that our models recovered significance for abiotic niche overlap, but not for geographical overlap. This probably occurred because the currently observed geographical overlap, as measured by the set of records compiled from the literature, represents an underestimation of the actual geographical overlap. This underestimation can be attributed not only to poor and biased sampling but also to difficulties involved in the identification of some cryptic FBD species (Carvalho et al 2023). In fact, the usual method of identifying *Drosophila* species based on morphological characters of the male genitalia can be problematic and dependent on molecular confirmation given the high frequency of cryptic species (Fonseca et al. 2017; Machado et al. 2022). Therefore, in this case, abiotic overlap measured from environmental niche models (ENMs) is likely to provide a better proxy for measuring the probability or frequency of two species being encountered in the same area.

The scarce knowledge of the life-history, biology, and host interactions in all stages of the life cycle of FDB species also helps to explain why biotic niche breadth was not positively associated with the potential for HTT among FBD species. We first hypothesized that, as generalist species might share resources with many different species and are highly connected in a *Drosophila*-flower interaction network, they would have greater opportunities for HTT, and so, a higher potential for HTT with a wider range of species. Nonetheless, this was not the case for the five target species studied here. In fact, the two species presenting the highest number of detected HTT were *D. lutzii* and *D. incompta*, which have largely different niche breadths. Moreover, the lowest number of HTT was detected for *D. bromeliae*, which is also a generalist species. However, the weak negative association detected between biotic niche overlap and HTT potential should be interpreted with caution. Indeed, besides the cryptic nature of several *Drosophila* species, identification of FBD occurs primarily after the emergence of adult males from flower hosts maintained in the laboratory (Schmitz and Valente 2019; Carvalho et al. 2023), limiting our knowledge of the biotic niche to the early stages of the life cycle. During the early stages, species exhibit more limited locomotion and greater resource dependance, leading to an underestimation of the true effective niche of these species.

Another interesting result relates to the absence of a significant influence of phylogenetic signal on the potential for HTT among species that diverged in the last 35 Mya (fig. 1*A*). In this context, it is interesting to note clear exceptions to the expected pattern in which the more closely related species would present a wider frequency of HTT, either due to a higher frequency of introgressive hybridization or because parasites would be better adapted to invade closely related hosts (Bartolomé et al. 2009; Peccoud et al. 2017). For example, a single HTT event could be detected between *D. bromeliae* and *D. bromelioides*, despite their recent divergence dated at approximately 2.65 Mya (fig. 1*A*). In contrast, 61 HTT were detected between *D. incompta* and *D. lutzii*, which belong to different *Drosophila* subgenera and appear to have diverged about 35 Mya (fig. 1*A*). Similar results were previously observed in several other taxonomic groups and at different time scales (Gilbert et al. 2010; Dupeyron et al. 2014; Gao et al. 2007; Metzger et al. 2018; De Melo and Wallau 2020), suggesting that genetic distance alone is insufficient to define the potential for HTT.

Finally, we found the presence of similar TEs between some of our FBD species and other distantly related herbivore insects, such as *Bombyx mori* and *Heliconius melpomene melpomene*. In this case, it is possible that HTT occurred when such species were syntopic in the same host species. Such co-occurrence is more difficult to be visualized in the case of the Helitron shared between *D. bromelioides* and *Rhodnius prolixus*, and especially in the case involving Mariner sequences of some species of the *bromeliae* group or *D. incompta* (Fonseca et al. 2019) and *Schmidtea mediterranea*. Nevertheless, these results also highlight the potential interference of intermediate vectors in the connections among different niches and even trophic levels, providing opportunities for HTTs among phylogenetically and ecologically distant species.

## Conclusions

Horizontal transfer is an important evolutionary mechanism for the spread and management of transposable elements in eukaryotic genomes. Here, we tested for the first time the role of different ecological traits in the potential for HTT, using FBD as a model. We suggest that abiotic niche overlap was an important driver in HTTs rates, while phylogenetic signal or niche breadth has not played a significant effect. We also showed that the two species with the highest influence on the potential for HTT were those presenting wider distribution ranges, despite the presence of contrasting niche breadths. We propose that the occurrence of these evolutionary events was possibly mediated or promoted by intermediate species in areas of sympatry, highlighting the role of environmental requirements overlaps on HTTs events. These results are reinforced when long fragments of TEs with a significant similarity with those characterized for *Drosophila* were recovered in distantly related species of Lepidoptera, Hemiptera, and Tricladida (Phylum: Platyhelminthes) orders.

## Materials and Methods

### Species and Sampling

We selected species of the *D. bromeliae*, *D. flavopilosa*, and *D. lutzii* groups because they present a well-characterized gradient of specialization-generalization (Schmitz and Valente 2019; Cordeiro et al. 2020). *Drosophila bromelioides* and *D. lutzii* are generalist FBD belonging to different species groups, and *D. bromeliae* sp. morphotype IV (Schmitz 2010) encompasses a specialist species, which oviposits only in flowers of *Solanum*. In addition, we also evaluated the genome of *D. bromeliae* published by Surovov et al. (2022), representing a third generalist FBD, and the mobilome of *D. incompta* published by Fonseca et al. (2019), as representative of a second specialist FBD.

All specimens whose genomes were newly characterized were collected in Southern Brazil. Adults of *Drosophila lutzii* emerged from *Cucurbita pepo* flowers (Cucurbitacea—29°40′48.6″S-53°48′18.4″W), whereas adults of *D. bromelioides* and *D. bromeliae* sp. morphotype IV emerged from flowers of *Brugmansia sualovensis* (Solanaceae—27°27′15.7″S-53°56′16.1″W) and *Solanum commrsonii* (Solanaceae—27°37′28.3″S-52°19′31.8″W), respectively. The identification of specimens was performed through external morphology and male genitalia patterns, using specialized taxonomic literature as a reference (Bachli et al. 2004; Schmitz 2010).

### Genome Sequencing and Assembly

Genomic DNA was extracted from a pool of 15 and 20 males of *D. bromelioides* and *D. lutzii*, respectively, following the NucleoSpin DNA Insect Kit protocol (Macherey-Nagel, Düren, Germany). For *D. bromeliae* sp. morphotype IV, a pool of 15 males had their genomic DNA extracted by the Macrogen^®^ company (https://dna.macrogen.com/eng/). All libraries were sequenced using the Illumina NovaSeq platform, with paired-end methodology (200x 150 bp), at the Macrogen^®^ Genome Center of Seoul. The quality of reads was assessed using FastQC (Andrews 2010) and the choice of kmers for assembler scripts was performed in kmergenie (Chikhi and Medvedev 2013). Given the high quality of sequencing and the absence of adaptors, trimming was not performed. *De novo* assemblies were obtained for each species through SPAdes (Bankevich et al. 2012), and analyzed through Quast v5, based on sequence length, number of contigs, and N50 (Mikheenko et al. 2018). Although SPADES was originally developed for de novo assembly of unicellular bacteria genomes and recently adapted for small eukaryotic genomes (Prjibelski et al. 2020), it has proven to be an efficient assembler for many *Drosophila* genomes (Vanderlinde et al. 2019; Kim et al. 2021; Surovov et al. 2022). Additionally, a gene space completeness analysis was performed on each genome assembly using the BUSCO package version 3.0.2 (Simão et al. 2015), to further address the adequacy of genome assemblies.

### Mobilome *de Novo* and Homology-Based Searches

A de novo search for repetitive elements was performed using the raw filtered Illumina reads on the Galaxy platform (Afgan et al. 2016) through the RepeatExplorer 2.0 tool (Novák et al. 2013). This algorithm makes TE discovery and characterization easier by using graph-based sequence clustering and then evaluating the similarity of each hit to reference databases (Novák et al. 2020). As a first step, FASTQ paired-end reads were preprocessed on the Galaxy platform, through interlacing and quality filtering. The file thus obtained was then employed as input for the graph-based clustering. After, the detected read similarities were used to build a virtual graph that is partitioned into clusters, returning contigs related to individual repeat superfamilies. In addition, supplementary information was also obtained for each supercluster, as the proportions of shared paired-end read among clusters. At this stage, sequences corresponding to satellites and other families of repetitive sequences were removed to evaluate the proportion of TEs along each genome. To detect and remove false overlaps, a second contig assembly was performed with the results obtained from RepeatExplorer in the program CAP3 (Huang and Madan 1999) using the parameters: -a 20 -b 20 -c 12 -d 200 -e 30 -f 20 -g 6 -m 2 -n −5 -*P* 80 -r 1 -s 900 -t 300 -u 3 -v 2 -o 40. The final contigs annotated by superclusters were then used in a homology-based search using RMBlast 2.10.0 against a curated TE database (Repbase v. 20181026 database) to evaluate TEs identity.

A second homology-based search using RMBlast 2.10.0 was performed with the draft assembled genome recovered for each species against Repbase v. 20181026 database, to obtain a complementary TE library. Further, to generate a robust characterization of the mobilome of each target species, we conducted a third and recursive homology-based search using RMBlast. For this, we used the assembled genomes against the de novo and homology-based combined TE libraries of the four anthophilous species whose mobilomes were newly characterized here, and added to the previously published mobilome of *D. incompta* (Fonseca et al. 2019). In all cases, the best query recovered for each contig was manually selected based on the best bit-score number, with a threshold of at least 160 bit-score and a minimum length of 130 bp, considering the minimum start and maximum end query sequences. Finally, the combined TEs libraries of each species were processed by CD-HIT-est clustering algorithm, removing redundant sequences with more than 95% identity (Fu et al. 2012). The mobilome thus characterized for each species represents a customized species-specific TE library.

To assess the relationship between TE richness (as given by the number of TEs superfamilies of each mobilome), or TE abundance (based on the total number of TEs) and species niche breadth or distribution area, we used Pearson's or Spearman correlations, respectively, using “ggpubr” and “ggoplot2” on R v. 3.6.3 (R Core Team 2020).

### TE Landscapes

Mobilome landscapes plot the distribution frequency of divergences between each TE and its respective consensus sequence using the Kimura two-parameters substitution model (Kimura 1980). From the graph, it is possible to evaluate the insertion and/or deletion events as well as the organization of specific superfamilies, which can reflect a rapid increase, stability, or decrease in the repeat copy number through time (Barrón et al. 2014; Petersen et al. 2019). For this purpose, we used the draft genome of each species as input against customized species-specific TE libraries obtained by the combined mobilome characterization strategies described above. The functions “calcdivergencefromalign.pl” and “createrepeatlandscape.pl” were employed to reconstruct landscapes after calculating the Kimura divergence values and plotting the graph on RepeatMasker 4.1.2, respectively.

### HT Analysis

For HTT analysis, only sequences with a minimum of 600 pb and at least 90% of similarity with TEs of another species were used. These thresholds were chosen to establish a conservative framework for recent HTT inferences, involving either the target species or their common ancestors. Sets of sequences with more than 90% identity were clustered by CD-HIT-est to facilitate alignment. Each cluster or family of sequences was finally aligned by codon using MACSE software (Wicker et al. 2007).

The R-package VHICA (Wallau et al. 2015) was used to identify signals of HT among the five FBD species, as implemented in R v. 3.6.3 (R Core Team 2020). This software searches for evidence of HT by comparing the relationship between synonymous substitution distances (dS) and CUB of TEs with those presented by a set of vertically transferred reference genes. If an element was horizontally transferred, a statistically significant deviation is detected in the CUB–dS values recovered for TEs in comparison to the values presented by reference genes. For this task, we employed 30 single-copy, well-conserved, and orthologous nuclear genes as a reference (see supplementary table S1, Supplementary Material online), as previously suggested by Wallau et al. (2015). These genes were isolated from the genomes of each of the three FDB species of the *bromeliae* species group and *D. lutzii*, using BLASTn and the orthologous gene sequences of *D. mojavensis* and *D. cardini* as a query. The reference genes of *D. incompta* were already isolated by Fonseca et al. (2019). Codon-based alignments of the coding sequences of this set of genes were then employed as a reference in VHICA tests.

To estimate the number of HTT involving each pair of species, without inferring direction, we first grouped the events according to superfamily and cluster. Each HTT event was standardized to “sum” four, considering the alternative scenarios and putative donor and acceptor species. According to the number of species involved in each HTT, we subdivided HTTs into two main groups:

1. When just two species presented signals of HTT for a given cluster, a single scenario was assumed (disregarding direction), and a value of 2 was assigned for each species.
2. When more than two species presented signals of HTT for the same cluster, alternative scenarios involving donor and acceptor species were considered in the face of the phylogenetic relationships among the target species (see below) and the magnitude of the dS values. In this case, if one of the directions of the event might have involved ancestral species, as inferred when similar dS values were recovered for a set of closely related species, the “sum” value of the scenario was equally divided among species. For example, the BEL element CL133 presented significant signals of HTT in the comparisons involving both, *D. bromeliae* sp. morphotype IV versus *D. bromeliae* and *D. bromeliae* sp. morphotype IV versus *D. bromelioides* (but not in the comparison involving *D. bromeliae* vs. *D. bromelioides*, showing that between these species the TE was vertically inherited). As *D. bromeliae* and *D. bromelioides* are sister species (see fig. 1*A*) and as both comparisons presented quite similar dS values, one of the putative directions of the HTT could be from *D. bromelioides* to *D. bromeliae* sp. morphotype IV (since this comparison presented a somewhat lower dS value), and the other was from *D. bromeliae* sp. morphotype IV to the ancestor of *D. bromeliae* and *D. bromelioides*. In this case, the first scenario summed 2, with a value of 1 being attributed to both, *D. bromelioides* and *D. bromeliae* sp. morphotype IV; although the second scenario also summed 2, in this case, a value of 0.5 was attributed to both, *D. bromeliae* and *D. bromelioides* (since they could have inherited the element from their common ancestor), and a value of 1 was attributed to *D. bromeliae* sp. morphotype IV (which would be directly involved in the HTT in both considered directions).

Summing the numbers obtained for each species and dividing them by four leads to the total number of HTT events per species.

### Dating HT Events

To estimate the time of HTT events, we used the formula *T* = *k*/2*r* (Graur and Li, 2000), where *T* is the time, *k* is the synonymous substitution rate (dS) between TE copies of two FBD species, and *r* is the synonymous substitution rate of the *Drosophila* clade. This rate was measured using the mean divergence time *T* between *Scaptomyza pallida* and the Hawaiian *Drosophila grimshawi*, dated to around 17.5 Mya by fossil evidence (Grimaldi 1987; Iturralde-Vinent and MacPhee 1996), and the mean dS values presented between these two species for the 30 single-copy orthologous genes employed in VHICA (see above). This led to a rate of 0.02022 substitutions per site per million years.

### Potential Effects in HT

To investigate potential effects in HTT rates among FBD, we used the Müller network index. This index estimates how one species (“acting”) affects another (“target”) of the same trophic level under shared interactions (Müller et al. 1999). For our purpose, we calculated the potential of each species (“acting”) to influence another (“target”) via shared TEs putatively obtained through HTT, as detected by VHICA, using the PAC function of the bipartite R-package (Dormann et al. 2009). This strategy results in an estimation of how *Drosophila* interactions influence the diffusion of TEs across the FDB network. Specifically, we applied the equation:


$$d_{ij\;}=\;\overset{}{\underset{k}{\sum }}\;\left[\frac{\alpha_{ik}}{\sum_{l}^{}\;\alpha_{il}}\;\frac{\alpha_{\;jk}}{\sum_{m}^{}\;\alpha_{mk}}\right]$$

where $\alpha_{ik}$ is how much of TE *k* is presented in *Drosophila* species *i*, and $\overset{}{\underset{l}{\sum }}\;\alpha_{il}$ is the total number of TEs, for all superfamilies, that species *i* receives via HTT. Otherwise, $\alpha_{\;jk}$ is how much of TE *k* is presented in *Drosophila* species *j*, and $\overset{}{\underset{m}{\sum }}\;\alpha_{mk}$ is the total number of HTT involving element *k* considering all *m Drosophila* species. In this way, the second term within the square brackets measures the contribution of *Drosophila* species *j* to the total number of TE *k* shared by HTT for all *Drosophila* species *m*. Thus, $d_{ij\;}$ summarizes interactions between two *Drosophila* species via all shared TEs. The result of this equation was plotted using Cytoscape v. 3.8.2 (Shannon et al. 2003).

We then tested if potential effects on HTT rates involving each pair of *Drosophila* species could be significantly influenced by:

- abiotic niche overlap, using Schoener's D climatic niche overlap statistics, calculated with the software ENMTools 1.0 for R (Warren et al. 2021). For this task, ENMs were first reconstructed for each species using the set of occurrences previously reported for them in the literature (Vilela and Mori 1999; Schmitz and Hofmann 2005; Chaves and Tidon 2008; Roque and Tidon 2008; Grimaldi 2016; Santa-Brígida et al. 2017; Schmitz and Valente 2019) (supplementary table S2, Supplementary Material online), added to our own records, and the set of climatic variables previously selected by Carvalho et al. (2023). These ENM were reconstructed using the Maxent algorithm (Phillips et al. 2006), with 10,000 background points selected along a radius of 500 km of each presence point.
- geographic overlap among species, using range overlap statistics calculated in the software QGIS 3.22.3 (QGIS Development Team, 2021) after reconstructing the minimum convex polygonum with the set of distribution points compiled for each species (supplementary table S2, Supplementary Material online). As intersection values were first measured in Km^2^, these values were divided by the total area of each species to get an asymmetric matrix of range overlap in percentage values.
- biotic niche overlap, using the Czechanowski index available on “spaa” R-package (Zhang 2013) and the set of interactions among drosophilids and angiosperms compiled by Cordeiro et al. (2020) (supplementary table S3, Supplementary Material online);
- niche breadth, calculated as the number of flower species used by each target species, divided by the total number of flower species in the Neotropical interaction network compiled by Cordeiro et al. (2020). This niche breadth ranges from 0 to 1, with values close to 0 indicating higher levels of specialization, whereas values close to 1 indicate higher levels of generalization (supplementary table S3, Supplementary Material online);
- phylogenetic signal, based on a phylogenetic tree reconstructed for the target species using the set of 30 protein-coding genes employed as reference genes in VHICA analysis. For this task, sequences of other Drosophilidae species were also added to the alignment, including three species of the subgenus *Siphlodora* (*D. mojavensis* and *D. hydei*, from the *repleta* group, and *D. virilis*, from the *virilis* group), two species of the *Drosophila* subgenus (*D. immigrans* and *D. cardini*, from the *immigrans* and *cardini* groups, respectively), two species of the *Idiomyia* lineage (*D. grimshawi* and *Scaptomyza pallida*, from the Hawaiian *Drosophila* and *Scaptomyza*, respectively) and one species of the *Sophophora* subgenus (*D. melanogaster*). Sequences of *Aedes aegypti* were employed as an outgroup. Individual gene trees were first reconstructed through Bayesian Analysis in MrBayes 3.2.7 (Ronquist et al. 2012), using the best-partitioned scheme and the respective substitution models selected by Bayesian information criterion (BIC) in PartitionFinder (Lanfear et al. 2012). Each analysis was run for at least 1,000,000 generations, saving every 1,000, and burning 25% of the results. Runs were increased every 1,000,000 generations until the standard deviation between the two runs dropped below 0.01. Unrooted gene trees were then employed to reconstruct the species tree in Astral 5.7.8 (Mirarab et al. 2014) under a multiple-species coalescent approach. In this case, support for each node was measured using local posterior probabilities (LPP). This species tree was then employed to measure divergence times under the Bayesian algorithm implemented in MCMCTree of the PAML X package (Xu and Yang 2013), using correlated substitution rates. The Markov Chain Monte Carlo (MCMC) process of PAML MCMCTree was run to sample 750 times, with sample frequency set to 1.000, after a burn-in of 250.000 iterations. In this case, the root age was set between 195 and 230 Mya, based on fossil estimations provided by Grimaldi and Engels (2005) for the older known specimen of Brachycera (*Oligophryne*) and Diptera (*Grauvogelia*), respectively.

To test the influence of abiotic niche overlap, geographic overlap, biotic niche overlap, niche breadth, and phylogenetic signal (predictor variables) in the potential for HTT, we used Muller’s index as the response variable in generalized linear mixed models using the *lme4* R-package (Bates et al. 2015). Models were built following Bergamo et al. (2017). We applied a logit transformation to the response variable to normalize the distribution of residuals and log-transformed niche overlap of the acting FBD to avoid overdispersion. Zero-inflation was not an issue. Since a species can influence any other species in the network, we accounted for identity by naming species as “acting” or “target” as random variables.

We tested models with all possible combinations between the fixed variables and a benchmark “Null” model with only the response variable and the random effects (“random effects model”) using the “dredge” function on *MuMin* R-package (Barton 2018) for R 3.6.3 (R Development Core Team 2020). Best models were selected based on Akaike Information Criteria (AIC) values, considering models with ΔAIC ≤ 2 as equivalents (Burnham and Anderson 2002).

## Supplementary Material

evad068_Supplementary_Data

## Data Availability

**Genetic data:** Nucleotide sequences of the entire mobilomes characterized for *D. bromeliae*, *D. bromelioides*, *D. bromeliae* sp. IV, and *D. lutzii* were submitted to Dryad https://doi.org/10.5061/dryad.qz612jmjs. The mobilome of *D. incompta* was previously reported by Fonseca et al. (2019). Nucleotide sequences of the 30 nuclear genes employed as reference in HTT analyses performed in VHICA were submitted to GenBank (Acc. Nos. OP168387—OP168476) regarding *D. bromelioides*, *D. bromeliae* sp. IV, and *D. lutzii*. The reference genes of *D. incompta* were already isolated by Fonseca et al. (2019), whereas the assembled genome of *D. bromeliae* was previously employed by Surovov et al. (2022) and is available in NCBI under Bioproject PRJNA611543. Benefits Generated: Doesn’t apply.
