# Supplementary material for: Horizontal Transposon Transfer and Their Ecological Drivers: The Case of Flower-breeding Drosophila
Source: Genome Biol Evol. 2023 Apr 26;15(5):evad068. doi: 10.1093/gbe/evad068 (PMC10167997; doi:10.1093/gbe/evad068)
Supplement: evad068_Supplementary_Data [file evad068_supplementary_data.pdf]

Supplementary data

Table S1. List of the 30 nuclear genes employed as reference in HTT analyses performed in VHICA, as well as in the estimation of the species tree, with their respective sequence, as recovered for each of the five target FBD species.

Nucler Genes	Flower-breeding species				
	D. bromeliae	*D. bromelioides*	*D. bromeliae* sp. IV	*D. incompta*	*D. lutzii*
Adar	Suvorov et al., (2022)	OP168387	OP168388	Fonseca et al., (2019)	OP168389
Adh	Suvorov et al., (2022)	OP168390	OP168391	Fonseca et al., (2019)	OP168392
Amd	Suvorov et al., (2022)	OP168393	OP168394	Fonseca et al., (2019)	OP168395
annexin	Suvorov et al., (2022)	OP168396	OP168397	Fonseca et al., (2019)	OP168398
Ap-1gamma	Suvorov et al., (2022)	OP168399	OP168400	Fonseca et al., (2019)	OP168401
blw	Suvorov et al., (2022)	OP168402	OP168403	Fonseca et al., (2019)	OP168404
Ca-P60A	Suvorov et al., (2022)	OP168405	OP168406	Fonseca et al., (2019)	OP168407
CG12065	Suvorov et al., (2022)	OP168426	OP168427	Fonseca et al., (2019)	OP168428
CG14434	Suvorov et al., (2022)	OP168429	OP168430	Fonseca et al., (2019)	OP168431
CG16912	Suvorov et al., (2022)	OP168432	OP168433	Fonseca et al., (2019)	OP168434
CG2812	Suvorov et al., (2022)	OP168408	OP168409	Fonseca et al., (2019)	OP168410
CG3419	Suvorov et al., (2022)	OP168411	OP168412	Fonseca et al., (2019)	OP168413
CG42265	Suvorov et al., (2022)	OP168435	OP168436	Fonseca et al., (2019)	OP168437
CG42388	Suvorov et al., (2022)	OP168438	OP168439	Fonseca et al., (2019)	OP168440
CG4386	Suvorov et al., (2022)	OP168414	OP168415	Fonseca et al., (2019)	OP168416
CG4585	Suvorov et al., (2022)	OP168417	OP168418	Fonseca et al., (2019)	OP168419
CG4593	Suvorov et al., (2022)	OP168420	OP168421	Fonseca et al., (2019)	OP168422
CG4610	Suvorov et al., (2022)	OP168474	OP168475	Fonseca et al., (2019)	OP168476
CG4797	Suvorov et al., (2022)	OP168423	OP168424	Fonseca et al., (2019)	OP168425
cln3	Suvorov et al., (2022)	OP168441	OP168442	Fonseca et al., (2019)	OP168443
Ddc	Suvorov et al., (2022)	OP168444	OP168445	Fonseca et al., (2019)	OP168446
G1cT-1	Suvorov et al., (2022)	OP168447	OP168448	Fonseca et al., (2019)	OP168449
Gclc	Suvorov et al., (2022)	OP168450	OP168451	Fonseca et al., (2019)	OP168452
grau	Suvorov et al., (2022)	OP168453	OP168454	Fonseca et al., (2019)	OP168455
Mcmc3	Suvorov et al., (2022)	OP168456	OP168457	Fonseca et al., (2019)	OP168458
Muscle_protein_20	Suvorov et al., (2022)	OP168459	OP168460	Fonseca et al., (2019)	OP168461
pgd	Suvorov et al., (2022)	OP168462	OP168463	Fonseca et al., (2019)	OP168464
TER-94	Suvorov et al., (2022)	OP168465	OP168466	Fonseca et al., (2019)	OP168467
Vps25	Suvorov et al., (2022)	OP168468	OP168469	Fonseca et al., (2019)	OP168470
Vps35	Suvorov et al., (2022)	OP168471	OP168472	Fonseca et al., (2019)	OP168473

Table S2. List of geographical records compiled for each of the five target FDBs. These datasets were used to measure geographical overlap, as also to reconstruct ENMs, which were employed to measure abiotic niche overlap.

Species	Locality	Long	Lat	Reference
D. bromeliae	Belém, Reserva do Mocambo, PA, Brazil	-1.442875	-48.409886	Schmitz & Valente (2019)
D. bromeliae	Recife, PE, Brazil	-8.053889	-34.950000	Schmitz & Valente (2019)
D. bromeliae	Recife, PE, Brazil	-8.012500	-34.949167	Schmitz & Valente (2019)
D. bromeliae	Vitória de Santo Antão, PE, Brazil	-8.101139	-35.294806	Schmitz & Valente (2019)
D. bromeliae	Tangará da Serra, MT, Brazil	-14.077222	-57.062500	Schmitz & Valente (2019)
D. bromeliae	Alejandro Labaka, Ecuardor	-0.66	-76.45	Confirmed by Grimaldi (2016)
D. bromeliae	São Salvador, El Salvador	13.69	-89.25	Confirmed by Grimaldi (2016)
D. bromeliae	San Luis, Honduras	15.21	-88.45	Confirmed by Grimaldi (2016)
D. bromeliae	Portland, Jamaica	18.10	-76.35	Confirmed by Grimaldi (2016)
D. bromeliae	Yucatan, Mexico	20.8283	-89.0001	Confirmed by Grimaldi (2016)
D. bromelioides	Anhatomirim Island, SC, Brazil	-27.26	-48.34	Schmitz & Valente (2019)
D. bromelioides	Antônio Carlos, SC, Brazil	-27.31	-48.46	Schmitz & Valente (2019)
D. bromelioides	Balneario dos Prazeres, RS, Brazil	-31.43	-52.12	Schmitz & Valente (2019)
D. bromelioides	Bossoroca, RS, Brazil	-28.44	-54.55	Poppe et al. (2014)
D. bromelioides	Brasília, DF, Brazil	-15.47	-47.59	Roque et al. (2015)
D. bromelioides	Campeche Island, SC, Brazil	-27.41	-48.28	De Toni et al. (2007)
D. bromelioides	Caxias do Sul, RS, Brazil	-29.10	-51.09	Schmitz & Valente (2019)
D. bromelioides	Planaltina, GO, Brazil	-15.47	-47.59	Cahaves & Tidon (2005)
D. bromelioides	Estrada Velha, RS, Brazil	-32.07	-52.09	Duarte et al. (2017)
D. bromelioides	Fazenda Brandalise, PR, Brazil	-25.19	-51.25	Cavasini et al. (2014)
D. bromelioides	Fazenda Trijuncao, BA, Brazil	-14.49	-45.58	Roque & Tidon (2008)
D. bromelioides	Florianópolis, SC, Brazil	-27.35	-48.32	Gottschalk et al. (2007)
D. bromelioides	Pelotas, RS, Brazil	-31.45	-52.23	Schmitz & Valente (2019)
D. bromelioides	Guarapuava, PR, Brazil	-25.24	-51.27	Schmitz & Valente (2019)
D. bromelioides	Joaquina, SC, Brazil	-27.37	-48.27	Schmitz & Valente (2019)
D. bromelioides	Joinville, SC, Brazil	-26.18	-48.51	Doge et al. (2008)
D. bromelioides	Laranjal, RS, Brazil	-31.45	-52.16	Schmitz & Valente (2019)
D. bromelioides	Morro da Lagoa da Conceição, SC, Brazil	-27.36	-48.29	Schmitz & Valente (2019)
D. bromelioides	Santo Antônio da Platina, PR, Brazil	-23.40	-50.00	Petersen (1960)
D. bromelioides	Parque das Araucarias, PR, Brazil	-25.24	-51.27	Cavasini et al. (2014)
D. bromelioides	Planaltina, DF, Brazil	-15.39	-47.42	Emerich et al. (2012)
D. bromelioides	Ponta do Coral, SC, Brazil	-27.34	-48.32	Schmitz & Valente (2019)
D. bromelioides	Porto Alegre, RS, Brazil	-30.02	-51.13	Schmitz & Valente (2019)
D. bromelioides	Praia da Joaquina, SC, Brazil	-27.38	-48.28	Bizzo et al. (2010)
D. bromelioides	Ratones, SC, Brazil	-27.27	-48.30	Schmitz et al. (2007)
D. bromelioides	Ratones, SC, Brazil	-27.28	-48.34	Schmitz & Valente (2019)
D. bromelioides	Rio de Janeiro, RJ, Brazil	-22.53	-43.17	Frota-Pessoa (1952)
D. bromelioides	Salvador, BA, Brazil	-12.58	-38.29	Malogolowkin (1951)
D. bromelioides	Santa Monica, SC, Brazil	-27.36	-48.31	Schmitz & Valente (2019)
D. bromelioides	São Jorge, GO, Brazil	-14.08	-47.31	Chaves & Tidon (2008)
D. bromelioides	São Paulo, SP, Brazil	-23.33	-46.39	Grimaldi (2016)
D. bromelioides	São Paulo, SP, Brazil	-22.00	-48.00	Tidon & Sene (1999)
D. bromelioides	Serra do Cipó, MG, Brazil	-19.17	-43.35	Vilela & Mori (1999)
D. bromelioides	Serra do Cipó, MG, Brazil	-19.00	-43.40	Chaves & Tidon (2008)
D. bromelioides	Tavares, SC, Brazil	-27.39	-48.32	Schmitz et al. (2007)
D. bromelioides	Quito, Ecuador	-0.40	-78.28	Acurio & Rafael (2009)
Dbromeliae sp. IV	Córrego Grande, SC, Brazil	-27.593778	-48.493611	Schmitz & Valente (2019)
Dbromeliae sp. IV	Cruz Alta, RS, Brazil	-28.569722	-53.614722	Schmitz & Valente (2019)
Dbromeliae sp. IV	Flores da Cunha, RS, Brazil	-29.024417	-51.194722	Schmitz & Valente (2019)
Dbromeliae sp. IV	Flores da Cunha, RS, Brazil	-29.027056	-51.195472	Schmitz & Valente (2019)
Dbromeliae sp. IV	Porto Alegre, RS, Brazil	-30.070111	-51.118583	Schmitz & Valente (2019)
Dbromeliae sp. IV	Erechim, RS	-27.624528	-52.325500	this study
D. lutzii	Governador Celso Ramos, SC, Brazil	-27.427247	-48.564969	Schmitz & Valente (2019)
D. lutzii	Antônio Carlos, SC, Brazil	-27.517604	-48.771731	Schmitz & Hoffman (2005)
D. lutzii	Armação, SC, Brazil	-27.354444	-48.539167	Schmitz & Hoffman (2005)
D. lutzii	Belém, PA, Brazil	-1.440833	-48.413889	Santa-Brigida et al. (2017)
D. lutzii	Chapadão do céu, GO, Brazil	-18.25	-52.88	Roque & Tidon (2008)
D. lutzii	Chapada dos guimarrães, MT, Brazil	-15.24	-55.49	Roque & Tidon (2008)
D. lutzii	Guarapuava, PR, Brazil	-25.402527	-51.475228	Schmitz & Valente (2019)
D. lutzii	Florianópolis, SC, Brazil	-27.602258	-48.525108	Schmitz & Hoffman (2005)
D. lutzii	Caxias do Sul, RS, Brazil	-29.183694	-51.170667	Schmitz & Valente (2019)
D. lutzii	Águas de Lindóia, SP, Brazil	-22.478611	-46.626111	Schmitz & Valente (2019)
D. lutzii	Porto Alegre, RS, Brazil	-30.0500	-51.2333	Schmitz & Hoffman (2005)
D. lutzii	Porto Alegre, RS, Brazil	-30.042778	-51.231944	Schmitz & Valente (2019)

D. lutzii	Bagé, RS, Brazil	-31.335126	-54.111468	Carvalho et al. (2023)
D. lutzii	Erechim, RS, Brazil	-27.622652	-52.267423	Carvalho et al. (2023)
D. lutzii	Santa Maria, RS, Brazil	-29.6848025	-53.8029788	this study
D. lutzii	Porto Alegre, RS, Brazil	-30.070111	-51.118583	Carvalho et al. (2023)
D. lutzii	Bogotá, Colômbia	4.48	-74.05	Tidon & Almeida (2016)
D. lutzii	Santa Gertrudis, Limón Province, Limon, Costa Rica	9.990694	-83.040444	Sturtevant (1916)
D. lutzii	Trinidad, Cuba	21.876489	-79.8794681	Sturtevant (1916)
D. lutzii	Pichincha, Ecuador	-1.044035	-79.8312951	Acurio & Rafael (2009)
D. lutzii	North Pacific Ocean, Hawaii	20.79557	-156.282169	Leblanc et al. (2009)
D. lutzii	Boston Bay, Jamaica	18.12	-76.34	Sturtevant (1916)
D. lutzii	Portland Parish, Jamaica	18.10	-76.35	Johnson (1919)
D. lutzii	Hanover Parish, Jamaica	18.4028341	-78.1745327	Gowdey (1926)
D. lutzii	231 Old Hope Road, Kingston, Jamaica	18.016417	-76.747361	Sturtevant (1921)
D. lutzii	Jojutla, Morelos, Mexico	18.57	-99.15	Chassagnard & Tsacas (1992)
D. lutzii	Santa Rosa Cintepec, Veracruz, Mexico	18.30	-95.10	Chassagnard & Tsacas (1992)
D. lutzii	Xochimilco, CDMX, Mexico	19.23	-99.08	Sturtevant (1921)
D. lutzii	Chiapas, Mexico	17.45	-92.48	Vilela & Bächli (2002)
D. lutzii	Mae Ho Phra, Mae Taeng District, Chiang Mai 50150, Thailand	19.08	-99.09	Chassagnard & Tsacas (1992)
D. lutzii	Santa Catalina, Coamo, Puerto Rico	18.10	-66.42	Sturtevant (1916)
D. lutzii	Luquillo, Puerto Rico	18.3728286	-65.7296007	Wolcott (1951)
D. lutzii	Unknown Street, Hormigueros, San Germán 00660, Puerto Rico	18.13	-67.09	Sturtevant (1916)
D. lutzii	Puerto Real, Vieques, Puerto Rico	18.12	-65.47	Sturtevant (1916)
D. lutzii	Lajas, Puerto Rico	18.00	-67.00	Townsend & Wheeler (1955)
D. lutzii	Miami, FL 33173, United States of America	25.6913793	-80.3511791	Sturtevant (1916)
D. lutzii	Key West, Florida, United States of America	24.553529	-81.8041724	Sturtevant (1916)
D. incompta	Agudo, RS, Brazil	-22.27	-49.03	Robe et al. (2013)
D. incompta	Panamá	9.20	-82.22	Tidon & Almeida (2016)
D. incompta	Panamá	9.19	-82.15	Brncic (1978)
D. incompta	Córdova, Argentina	-31.25	-64.11	Brncic (1978)
D. incompta	Mexico	18.55	-96.55	Wheeler et al. (1962)
D. incompta	El Dorado do Sul, SP, Brazil	-24.33	-48.08	Brncic (1978)
D. incompta	Federico westphalen, RS, Brazil	-27.20	-53.24	Wallau et al. (2016)
D. incompta	Horizontina, RS, Brazil	-27.38	-54.18	Robe et al. (2013)
D. incompta	ibiuna, SP, Brazil	-23.44	-47.12	Santos & Vilela (2005)
D. incompta	Puerto Lleras, Meta, Colombia	3.00	-73.00	Brncic (1978)
D. incompta	Montenegro, Brazil	-29.39	-51.29	Brncic (1978)
D. incompta	Saldanha Marinho, RS, Brazil	-28.25	-53.06	Robe et al. (2013)
D. incompta	Igaratá, SP, Brazil	-23.19	-46.13	Santos & Vilela (2005)
D. incompta	Santo Cristo, RS, Brazil	-27.50	-54.40	Robe et al. (2013)
D. incompta	Guarulhos, SP, Brazil	-23.33	-46.39	Santos & Vilela (2005)
D. incompta	São Sepe, RS, Brazil	-30.10	-53.35	Robe et al. (2013)
D. incompta	Sapucaia do Sul, RS, Brazil	-30.00	-52.00	Santos & Vilela (2005)
D. incompta	Taim, RS, Brazil	-32.29	-52.32	Hofmann & Napp (1984)
D. incompta	Turvo, RS, Brazil	-28.50	-49.42	Hofmann & Napp (1984)
D. incompta	Mexico	19.11	-96.15	Brncic (1978)
D. incompta	Viamão, RS, Brazil	-30.01	-51.06	Robe et al. (2013)
D. incompta	Meta, Colombia	4.09	-73.38	Brncic (1978)
D. incompta	Belo Horizonte, MG, Brazil	-19.868085	-43.965771	Moreira et al. (2017)
D. incompta	Antonina, PR, Brazil	-25.432782	-48.713803	Moreira et al. (in progress)
D. incompta	Bage, RS, Brazil	-31.330142	-54.100461	Carvalho et al. (in progress)
D. incompta	Cachoeira do Sul, RS, Brazil	-30.038889	-52.893889	Moreira et al. (in progress)
D. incompta	Canguçu, RS, Brazil	-31.396478	-52.678786	Moreira et al. (in progress)
D. incompta	Cruz Alta, RS, Brazil	-28.568597	-53.622422	Moreira et al. (in progress)
D. incompta	Curitiba, PR, Brazil	-25.424428	-49.265381	Moreira et al. (in progress)
D. incompta	Florianópolis, SC, Brazil	-27.596944	-48.548889	Moreira et al. (in progress)
D. incompta	Frederico Westphalen, RS, Brazil	-27.359122	-53.396336	Moreira et al. (in progress)
D. incompta	Horizontina, RS, Brazil	-27.625833	-54.307778	Moreira et al. (in progress)
D. incompta	Itaara, RS, Brazil	-29.590933	-53.758611	Moreira et al. (in progress)
D. incompta	Pelotas, RS, Brazil	-31.771944	-52.342778	Carvalho et al. (in progress)
D. incompta	Porto Alegre, RS, Brazil	-30.076544	-51.124361	Carvalho et al. (in progress)
D. incompta	Rio de Janeiro, RJ, Brazil	-32.033499	-52.099490	Carvalho et al. (in progress)
D. incompta	Santa Maria, RS, Brazil	-29.683889	-53.806944	Carvalho et al. (in progress)
D. incompta	Santiago, RS, Brazil	-29.17	-54.86	Moreira et al. (in progress)
D. incompta	São João do Polêsine, RS, Brazil	-29.649925	-53.516758	Moreira et al. (in progress)
D. incompta	Torres, RS, Brazil	-29.302380	-49.747749	Moreira et al. (in progress)
D. incompta	Arroi Grande, RS, Brazil	-29.651183	-53.673447	Moreira et al. (in progress)
D. incompta	Três Passos, RS, Brazil	-27.455933	-53.930158	Carvalho et al. (in progress)

Table S3. List of registers compiled for each of the five target FBDs in flowers of different angiosperm species, as evidenced in the neotropical interaction network reconstructed by Cordeiro et al. (2020). Generalization degree presented in the last line was calculated as the number of different host flower species used by each target species, divided by the total number of evaluated flower species.

Angiosperm species	*D. bromeliae*	*D. bromelioides*	*D. bromeliae* sp. IV	*D. incompta*	*D. lutzii*
Abutilon sp.	0	1	0	0	0
Allamanda polyantha	0	1	0	0	0
Amphilophium cuneifolium	1	0	0	0	0
Aristolochia gigantea	0	1	0	0	0
Bauhinia variegata	0	1	0	0	0
Brugmansia arborea	0	0	0	0	1
Brugmansia suaveolens	0	1	0	0	1
Brunfelsia grandiflora	0	1	0	0	0
Ceiba speciosa	0	1	0	0	0
*Convolvulaceae**	1	0	0	0	0
Cordia superba	0	1	0	0	0
Cucurbita maxima	0	0	0	0	1
Cucurbita pepo	0	1	0	0	1
Dietes bicolor	0	1	0	0	0
Dolichandra unguis-cati	0	1	0	0	0
Dombeya wallichii	0	1	0	0	0
Erythrina crista-galli	0	1	0	0	0
Gossypium sp.	0	0	0	0	1
Handroanthus albus	0	1	0	0	0
Handroanthus heptaphyllus	0	1	0	0	0
Hedychium coronarium	0	1	0	0	0
Heliconia sp.	1	0	0	0	0
Hibiscus rosa-sinensis	1	1	0	0	0
Hibiscus sabdariffa	0	0	0	0	1
Hibiscus sp.	0	0	0	0	1
Hibiscus syriacus	0	1	0	0	0
Hibiscus tiliaceus	0	1	0	0	0
Hippeastrum sp.	0	1	0	0	0
Hypericum sp.	0	1	0	0	0
Ipomoea aff. chiliantha	0	1	0	0	1
Ipomoea alba	0	1	0	0	0
Ipomoea asarifolia	1	0	0	0	0
Ipomoea batatas	0	1	0	0	1
Ipomoea cairica	0	1	0	0	1
Ipomoea carnea	0	1	0	0	1
Ipomoea indica	0	1	0	0	1
Ipomoea pes-caprae	0	1	0	0	1
Ipomoea purpurea	0	1	0	0	0
Ipomoea sp.	0	0	0	0	1
Ipomoea ternata	0	1	0	0	0
Jacaranda mimosifolia	0	1	0	0	0
Jacaranda sp.	0	1	0	0	0
Luffa cylindrica	0	1	0	0	0
Malvaviscus penduliflorus	0	1	0	0	0
Passiflora alata	0	1	0	0	0
Passiflora edulis	0	1	0	0	0
Pseudobombax grandiflorum	0	0	0	0	1
Pyrostegia venusta	0	1	0	0	0
Solanum guaraniticum	0	1	1	0	0
Solanum mauritianum	0	0	1	0	0
Solanum paniculatum	0	1	1	0	0
Solanum commersonii	0	0	1	0	0
Solanum sanctae-catharinae	0	0	1	0	0
Solanum sp.	0	1	0	0	0
Spathodea campanulata	0	1	0	0	0
Talipariti tiliaceum var. pernambucense	0	1	0	0	0
Tecoma stans	0	1	0	0	0
Thunbergia alata	0	1	0	0	0
Thunbergia grandiflora	1	1	0	0	0
Tibouchina sp.	0	1	0	0	0
Tropaeolum majus	0	1	0	0	0
Wisteria floribunda	0	1	0	0	0
Yucca gigantea	0	1	0	0	0
Zantedeschia sp.	0	0	0	0	1
Cestrum amictum	0	0	0	1	0
Cestrum calycinum	0	0	0	1	0
Cestrum corymbosum	0	0	0	1	0
Cestrum intermedium	0	0	0	1	0
Cestrum nocturnum	0	0	0	1	0
Cestrum parqui	0	0	0	1	0
Cestrum schlechtendalii	0	0	0	1	0
Cestrum sendtnerianum	0	0	0	1	0
Sessea brasiliensis	0	0	0	1	0
Total	6	49	5	9	16
Generalization degree	0,082191781	0,671232877	0,068493151	0,123287671	0,2191781

Table S4. Results of Quast and Busco analyses reporting the quality of the genome assemblies recovered in Spades for each of the three FDBs that were sequenced in this study.

Quality assemblies test	Parameters	*Drosophila* species		
	Statistics without reference	*D. bromelioides*	*D. bromeliae* sp. IV	*D. lutzii*
Quast	# contigs	18489	40455	19245
	# contigs (>= 0 bp)	378573	223535	481191
	# contigs (>= 1000 bp)	8881	16041	9850
	Largest contig	770493	553267	689130
	Total length	152262188	158415734	147986388
	Total length (>= 0 bp)	202799381	200288127	199160793
	Total length (>= 1000 bp)	145409858	141668134	141090954
	N50	67870	40224	46424
	N75	25408	6053	17614
	L50	525	889	771
	L75	1425	3328	2040
	GC (%)	46.24	44.86	46.44
	# N's	1122841	603987	1370446
	# N's per 100 kbp	737.44	381.27	926.06
BUSCO	Complete BUSCOs	97.6%	96.1%	96.2%
	Complete and single-copy BUSCOs	97.1%	95.7%	95.8%
	Complete and duplicated BUSCOs	0.5%	0.4%	0.4%
	Fragmented BUSCOs	1.2%	2.3%	2.2%
	Missing BUSCOs	1.2%	1.6%	1.6%
	Total BUSCO groups searched	3285	3285	3285

Table S5. Results of VHICA analysis performed to identify signals of HTTs among the five evaluated FBD species, based on comparisons of dS (synonymous substitution distances) and codon usage bias (CUB) presented by each pair of FBD species for each TE and the set of 30 vertically transferred reference genes. Divergence times of HTTs were estimated using the formula T = k/2r (Graur & Li, 2000) and a synonymous substitution rate measured for nuclear gens of *Drosophila* (r = 0.02022).

Cluster	dS	time	Code sp1	Code sp2	pvalue	Alignment lenght (pb)	Match in RepBase database
CL00	0,13054	3,22806	Dbromelioides_0152Contig1	Dbromeliae_sp_IV_NODE_2863_length_7911_cov_209.060349	0,00668	654	Class I retrotransposons/LTR/BELPao
CL07	0,09784	2,41936	Dbromeliae_Contig0038_4	Dlutzii_NODE_8780_length_5347_cov_94.336948	0,00000	2916	Class I retrotransposons/LTR/BELPao
CL07	0,06829	1,68859	Dbromelioides_0075Contig2	Dlutzii_NODE_8780_length_5347_cov_94.336948	0,00000	2916	Class I retrotransposons/LTR/BELPao
CL07	0,16115	3,98480	Dlutzii_NODE_8780_length_5347_cov_94.336948	Dbromeliae_sp_IV_CL85_Contig6	0,00011	2916	Class I retrotransposons/LTR/BELPao
CL129	0,10841	2,68079	Dbromeliae_contig_302256	Dbromeliae_sp_IV_CL39_Contig3	0,00271	858	Class I retrotransposons/LTR/BELPao
CL13	0,11862	2,93319	Dbromeliae_Contig0038_4	Dbromeliae_sp_IV_NODE_4490_length_3542_cov_38.499269	0,00522	1707	Class I retrotransposons/LTR/BELPao
CL133	0,08230	2,03508	Dbromeliae_Contig0038_4	Dbromeliae_sp_IV_NODE_7472_length_1675_cov_38.742268	0,00522	1407	Class I retrotransposons/LTR/BELPao
CL133	0,08230	2,03508	Dbromeliae_Contig0038_4	Dbromeliae_sp_IV_NODE_7472_length_1675_cov_38.742268	0,00378	1407	Class I retrotransposons/LTR/BELPao
CL133	0,09676	2,39259	Dbromelioides_0075Contig2	Dbromeliae_sp_IV_NODE_7472_length_1675_cov_38.742268	0,01440	1407	Class I retrotransposons/LTR/BELPao
CL16	0,16068	3,97339	Dbromelioides_0152Contig1	Dbromeliae_sp_IV_NODE_2792_length_8322_cov_18.589340	0,04357	2793	Class I retrotransposons/LTR/BELPao
CL165	0,09837	2,43257	Dbromelioides_0075Contig2	Dbromeliae_sp_IV_NODE_5718_length_2391_cov_62.447972	0,04357	1074	Class I retrotransposons/LTR/BELPao
CL165	0,05888	1,45605	Dbromeliae_Contig0038_4	Dlutzii_NODE_20796_length_2003_cov_157.419456	0,00000	1074	Class I retrotransposons/LTR/BELPao
CL165	0,07064	1,74679	Dbromelioides_0075Contig2	Dlutzii_NODE_20796_length_2003_cov_157.419456	0,00000	1074	Class I retrotransposons/LTR/BELPao
CL165	0,10334	2,55527	Dlutzii_NODE_20796_length_2003_cov_157.419456	Dbromeliae_sp_IV_NODE_5718_length_2391_cov_62.447972	0,00000	1074	Class I retrotransposons/LTR/BELPao
CL165	0,08701	2,15155	Dbromeliae_Contig0038_4	Dbromeliae_sp_IV_NODE_5718_length_2391_cov_62.447972	0,01020	1074	Class I retrotransposons/LTR/BELPao
CL165	0,09837	2,43257	Dbromelioides_0075Contig2	Dbromeliae_sp_IV_NODE_5718_length_2391_cov_62.447972	0,03077	1074	Class I retrotransposons/LTR/BELPao
CL22	0,16185	4,00232	Dbromeliae_Contig0025_3	Dlutzii_NODE_3493_length_12119_cov_51.094613	0,00000	2232	Class I retrotransposons/LTR/BELPao
CL22	0,18473	4,56807	Dbromelioides_0152Contig1	Dlutzii_NODE_3493_length_12119_cov_51.094613	0,00000	2232	Class I retrotransposons/LTR/BELPao
CL22	0,11928	2,94955	Dlutzii_NODE_3493_length_12119_cov_51.094613	Dbromeliae_sp_IV_NODE_2744_length_8571_cov_80.840791	0,00000	2232	Class I retrotransposons/LTR/BELPao
CL320	0,08526	2,10821	Dincompta_NODE_129974_length_717_cov_5.477273	Dlutzii_NODE_7551_length_6185_cov_73.280276	0,00000	723	Class I retrotransposons/LTR/BELPao
CL370	0,13054	3,22806	Dbromelioides_NODE_2261_length_10852_cov_96.771938	Dbromeliae_sp_IV_NODE_4977_length_2997_cov_28.924843	0,00668	654	Class I retrotransposons/LTR/BELPao
CL40	0,06135	1,51700	Dbromeliae_Contig0038_4	Dlutzii_NODE_10777_length_4227_cov_79.290619	0,00000	2103	Class I retrotransposons/LTR/BELPao
CL40	0,04084	1,00984	Dbromelioides_0075Contig2	Dlutzii_NODE_10777_length_4227_cov_79.290619	0,00000	2103	Class I retrotransposons/LTR/BELPao
CL40	0,13167	3,25602	Dlutzii_NODE_10777_length_4227_cov_79.290619	Dbromeliae_sp_IV_NODE_2654_length_9112_cov_11.563355	0,00003	2103	Class I retrotransposons/LTR/BELPao
CL77	0,09578	2,36834	Dbromeliae_Contig0038_4	Dlutzii_NODE_7544_length_6188_cov_99.502378	0,00000	1317	Class I retrotransposons/LTR/BELPao
CL77	0,11454	2,83231	Dbromelioides_0075Contig2	Dlutzii_NODE_7544_length_6188_cov_99.502378	0,00000	1317	Class I retrotransposons/LTR/BELPao
CL77	0,17791	4,39943	Dlutzii_NODE_7544_length_6188_cov_99.502378	Dbromeliae_sp_IV_NODE_5762_length_2366_cov_51.164958	0,00003	702	Class I retrotransposons/LTR/BELPao
CL82	0,15524	3,83867	Dlutzii_NODE_10950_length_4156_cov_64.799262	Dbromeliae_sp_IV_NODE_3793_length_4822_cov_57.393062	0,00003	702	Class I retrotransposons/LTR/BELPao
CL134	0,04481	1,10814	Dlutzii_NODE_5674_length_8132_cov_441.757866	Dbromeliae_sp_IV_CL39_Contig3	0,00001	840	Class I retrotransposons/LTR/BELPao
CL62	0,00696	0,17218	Dincompta_NODE_3154_length_4748_cov_21.751598	Dbromeliae_sp_IV_NODE_6616_length_1955_cov_79.354803	0,00033	1605	Class I retrotransposons/LTR/Copia
CL101	0,15272	3,77651	Dbromelioides_NODE_3954_length_2953_cov_609.759582	Dbromeliae_sp_IV_CL145_Contig2	0,01842	1545	Class I retrotransposons/LTR/Copia
CL106	0,05426	1,34168	Dbromelioides_0145Contig1	Dlutzii_NODE_20311_length_2057_cov_336.481689	0,00000	1530	Class I retrotransposons/LTR/Copia
CL106	0,08218	2,03202	Dbromelioides_0145Contig1	Dbromeliae_sp_IV_CL145_Contig2	0,01044	1530	Class I retrotransposons/LTR/Copia
CL106	0,07795	1,92749	Dlutzii_NODE_20311_length_2057_cov_336.481689	Dbromeliae_sp_IV_CL145_Contig2	0,00000	1530	Class I retrotransposons/LTR/Copia
CL106	0,11075	2,73874	Dbromeliae_contig_218177	Dlutzii_NODE_20311_length_2057_cov_336.481689	0,00000	1530	Class I retrotransposons/LTR/Copia
CL106	0,07439	1,83941	Dbromeliae_contig_218177	Dbromeliae_sp_IV_CL145_Contig2	0,00479	1530	Class I retrotransposons/LTR/Copia
CL111	0,14434	3,56922	Dincompta_Copia-5_Dinc-I	Dlutzii_NODE_19552_length_2156_cov_102.044068	0,00004	1494	Class I retrotransposons/LTR/Copia
CL94	0,12102	2,99268	Dincompta_Copia-5_Dinc-I	Dlutzii_NODE_15546_length_2782_cov_161.890004	0,00001	1491	Class I retrotransposons/LTR/Copia
CL120	0,13935	3,44576	Dbromelioides_0093Contig2	Dbromeliae_sp_IV_NODE_2108_length_13095_cov_16.993216	0,01951	1449	Class I retrotransposons/LTR/Copia
CL120	0,13437	3,32260	Dbromeliae_Contig0085_1	Dbromeliae_sp_IV_NODE_2108_length_13095_cov_16.993216	0,00659	1449	Class I retrotransposons/LTR/Copia
CL136	0,13330	3,29618	Dbromelioides_0084Contig3	Dincompta_NODE_417_length_8020_cov_21.711167	0,00000	1386	Class I retrotransposons/LTR/Copia
CL240	0,12321	3,04666	Dbromelioides_0145Contig1	Dbromeliae_sp_IV_CL145_Contig2	0,02204	954	Class I retrotransposons/LTR/Copia
CL240	0,09396	2,32341	Dbromeliae_contig_67788	Dbromeliae_sp_IV_CL145_Contig2	0,00338	954	Class I retrotransposons/LTR/Copia
CL196	0,11367	2,81089	Dbromelioides_NODE_3288_length_4334_cov_100.013644	Dlutzii_NODE_18883_length_2244_cov_48.006503	0,00000	933	Class I retrotransposons/LTR/Copia
CL249	0,07928	1,96037	Dincompta_Copia-1_Dper-I_8	Dbromeliae_sp_IV_NODE_13036_length_1107_cov_78.618902	0,00000	918	Class I retrotransposons/LTR/Copia
CL142	0,09027	2,23225	Dbromelioides_NODE_6320_length_1396_cov_74.892612	Dbromeliae_sp_IV_CL67_Contig2	0,00561	843	Class I retrotransposons/LTR/Copia
CL282	0,09688	2,39556	Dbromelioides_NODE_4573_length_2238_cov_89.910441	Dbromeliae_sp_IV_CL67_Contig2	0,00303	813	Class I retrotransposons/LTR/Copia
CL289	0,26573	6,57098	Dbromelioides_NODE_12073_length_793_cov_446.447887	Dincompta_Copia-5_Dinc-I	0,00003	795	Class I retrotransposons/LTR/Copia
CL310	0,02335	0,57729	Dbromeliae_Contig0085_1	Dbromelioides_NODE_10884_length_866_cov_64.730524	0,02146	744	Class I retrotransposons/LTR/Copia

CL313	0,20902	5,16863	Dbromelioides_0094Contig1	Dlutzii_NODE_15167_length_2864_cov_52.874865	0,00008	732	Class I retrotransposons/LTR/Copia
CL313	0,15426	3,81442	Dbromeliae_contig_73339	Dlutzii_NODE_15167_length_2864_cov_52.874865	0,00009	732	Class I retrotransposons/LTR/Copia
CL315	0,16352	4,04348	Dbromelioides_0094Contig1	Dlutzii_NODE_15167_length_2864_cov_52.874865	0,00000	726	Class I retrotransposons/LTR/Copia
CL337	0,10617	2,62543	Dbromelioides_NODE_3268_length_4394_cov_17.128740	Dlutzii_NODE_7537_length_6196_cov_4.825880	0,00000	699	Class I retrotransposons/LTR/Copia
CL342	0,24945	6,16846	Dbromelioides_NODE_13674_length_697_cov_112.387622	Dincompta_Copia-6_Dinc-I_2	0,00000	681	Class I retrotransposons/LTR/Copia
CL352	0,08427	2,08385	Dbromeliae_contig_223252	Dbromeliae_sp_IV_CL145_Contig2	0,00087	672	Class I retrotransposons/LTR/Copia
CL390	0,06664	1,64785	Dbromelioides_NODE_14740_length_637_cov_223.341155	Dbromeliae_sp_IV_CL67_Contig2	0,00099	624	Class I retrotransposons/LTR/Copia
CL391	0,13012	3,21770	Dbromelioides_0127Contig1	Dlutzii_NODE_21412_length_1938_cov_40.810504	0,00000	624	Class I retrotransposons/LTR/Copia
CL06	0,00500	0,12372	Dincompta_NODE_1316_length_6098_cov_36.814104	Dbromeliae_sp_IV_CL161_Contig1	0,00000	4506	Class I retrotransposons/LTR/Gypsy/Gypsy
CL01	0,15954	3,94523	Dbromelioides_NODE_2683_length_6926_cov_81.649569	Dincompta_NODE_88_length_11151_cov_5.004146	0,00000	3141	Class I retrotransposons/LTR/Gypsy/Gypsy
CL01	0,12311	3,04423	Dbromelioides_NODE_2683_length_6926_cov_81.649569	Dlutzii_NODE_5969_length_7739_cov_62.463781	0,00000	3141	Class I retrotransposons/LTR/Gypsy/Gypsy
CL01	0,14050	3,47421	Dincompta_NODE_88_length_11151_cov_5.004146	Dlutzii_NODE_5969_length_7739_cov_62.463781	0,00000	3141	Class I retrotransposons/LTR/Gypsy/Gypsy
CL11	0,08167	2,01954	Dincompta_Gypsy-12_Dinc_2	Dlutzii_NODE_4894_length_9300_cov_57.105875	0,00010	2528	Class I retrotransposons/LTR/Gypsy/Gypsy
CL26	0,12512	3,09391	Dincompta_Gypsy-24_Dinc_3	Dlutzii_NODE_15959_length_2707_cov_295.162844	0,00079	2517	Class I retrotransposons/LTR/Gypsy/Gypsy
CL30	0,08493	2,10014	Dbromelioides_NODE_3136_length_4759_cov_65.788922	Dbromeliae_sp_IV_NODE_5695_length_2400_cov_59.615722	0,00970	2220	Class I retrotransposons/LTR/Gypsy/Gypsy
CL30	0,00337	0,08330	Dbromeliae_Contig0053_16	Dbromeliae_sp_IV_NODE_5695_length_2400_cov_59.615722	0,00337	2220	Class I retrotransposons/LTR/Gypsy/Gypsy
CL37	0,13235	3,27287	Dincompta_NODE_809_length_6881_cov_29.714055	Dlutzii_NODE_9644_length_4806_cov_43.361400	0,00032	2189	Class I retrotransposons/LTR/Gypsy/Gypsy
CL37	0,11864	2,93363	Dincompta_NODE_809_length_6881_cov_29.714055	Dbromeliae_sp_IV_CL46_Contig2	0,00035	2189	Class I retrotransposons/LTR/Gypsy/Gypsy
CL37	0,09768	2,41553	Dlutzii_NODE_9644_length_4806_cov_43.361400	Dbromeliae_sp_IV_CL46_Contig2	0,00006	2189	Class I retrotransposons/LTR/Gypsy/Gypsy
CL44	0,06059	1,49825	Dincompta_Gypsy-15_Dinc_2	Dlutzii_NODE_13815_length_3184_cov_68.655027	0,00020	2148	Class I retrotransposons/LTR/Gypsy/Gypsy
CL47	0,04332	1,07122	Dincompta_Gypsy-26_Dan-I_3	Dlutzii_NODE_3563_length_11932_cov_55.123469	0,00000	2136	Class I retrotransposons/LTR/Gypsy/Gypsy
CL50	0,04209	1,04072	Dincompta_Gypsy-37_Dinc	Dbromeliae_sp_IV_NODE_6310_length_2085_cov_87.513761	0,00008	2091	Class I retrotransposons/LTR/Gypsy/Gypsy
CL51	0,20057	4,95977	Dbromelioides_NODE_3349_length_4153_cov_74.657740	Dincompta_NODE_1724_length_5668_cov_34.073592	0,00001	2091	Class I retrotransposons/LTR/Gypsy/Gypsy
CL51	0,13515	3,34206	Dbromelioides_NODE_3349_length_4153_cov_74.657740	Dlutzii_NODE_1721_length_19128_cov_57.522299	0,00000	2091	Class I retrotransposons/LTR/Gypsy/Gypsy
CL51	0,09499	2,34883	Dincompta_NODE_1724_length_5668_cov_34.073592	Dlutzii_NODE_1721_length_19128_cov_57.522299	0,00018	2091	Class I retrotransposons/LTR/Gypsy/Gypsy
CL51	0,18610	4,60178	Dincompta_NODE_1724_length_5668_cov_34.073592	Dbromeliae_sp_IV_CL46_Contig7	0,00027	2091	Class I retrotransposons/LTR/Gypsy/Gypsy
CL51	0,26200	6,47866	Dlutzii_NODE_1721_length_19128_cov_57.522299	Dbromeliae_sp_IV_CL46_Contig7	0,00126	2091	Class I retrotransposons/LTR/Gypsy/Gypsy
CL52	0,23388	5,78328	Dbromelioides_NODE_2724_length_6684_cov_47.292077	Dlutzii_NODE_13740_length_3208_cov_63.110042	0,00000	2067	Class I retrotransposons/LTR/Gypsy/Gypsy
CL61	0,08282	2,04788	Dincompta_Gypsy-12_Dinc_1	Dlutzii_NODE_6345_length_7327_cov_61.776396	0,00004	1962	Class I retrotransposons/LTR/Gypsy/Gypsy
CL42	0,05381	1,33059	Dbromelioides_0040Contig15	Dlutzii_NODE_5673_length_8133_cov_113.794703	0,00000	1791	Class I retrotransposons/LTR/Gypsy/Gypsy
CL42	0,17160	4,24328	Dbromeliae_Contig0045_5	Dlutzii_NODE_5673_length_8133_cov_113.794703	0,00000	1791	Class I retrotransposons/LTR/Gypsy/Gypsy
CL74	0,23784	5,88120	Dincompta_Gypsy-28_Dinc	Dbromeliae_sp_IV_NODE_5300_length_2697_cov_30.065268	0,00000	1734	Class I retrotransposons/LTR/Gypsy/Gypsy
CL76	0,17964	4,44217	Dincompta_NODE_9648_length_3138_cov_31.881076	Dlutzii_NODE_14197_length_3087_cov_49.446262	0,00020	1710	Class I retrotransposons/LTR/Gypsy/Gypsy
CL71	0,03416	0,84474	Dincompta_Gypsy-26_Dan-I_3	Dlutzii_NODE_7969_length_5867_cov_50.901662	0,00049	1561	Class I retrotransposons/LTR/Gypsy/Gypsy
CL117	0,04126	1,02037	Dbromelioides_NODE_2703_length_6812_cov_66.412543	Dlutzii_NODE_3436_length_12280_cov_61.308885	0,00000	1458	Class I retrotransposons/LTR/Gypsy/Gypsy
CL131	0,12493	3,08938	Dincompta_NODE_25751_length_1974_cov_16.500261	Dlutzii_NODE_14429_length_3037_cov_47.983707	0,00000	1443	Class I retrotransposons/LTR/Gypsy/Gypsy
CL151	0,19142	4,73343	Dbromelioides_NODE_2455_length_8603_cov_65.666549	Dincompta_Gypsy-41_Dinc	0,00041	1299	Class I retrotransposons/LTR/Gypsy/Gypsy
CL19	0,08888	2,19793	Dbromelioides_0191Contig1	Dlutzii_NODE_16517_length_2604_cov_72.482292	0,00000	1284	Class I retrotransposons/LTR/Gypsy/Gypsy
CL171	0,21449	5,30390	Dincompta_Gypsy9-I_Dya_2	Dlutzii_NODE_27834_length_1494_cov_61.003564	0,00043	1182	Class I retrotransposons/LTR/Gypsy/Gypsy
CL174	0,09684	2,39473	Dincompta_Gypsy-6_Dwil-I_2	Dlutzii_NODE_18350_length_2319_cov_314.773788	0,00000	1164	Class I retrotransposons/LTR/Gypsy/Gypsy
CL184	0,00485	0,11990	Dincompta_Gypsy-22_Dwil-I_2	Dbromeliae_sp_IV_NODE_7380_length_1697_cov_124.907243	0,00000	1119	Class I retrotransposons/LTR/Gypsy/Gypsy
CL193	0,13921	3,44229	Dbromelioides_0095Contig10	Dbromeliae_sp_IV_NODE_1560_length_20367_cov_21.184944	0,00464	1095	Class I retrotransposons/LTR/Gypsy/Gypsy
CL190	0,05098	1,26066	Dbromelioides_0141Contig1	Dbromeliae_sp_IV_NODE_6131_length_2163_cov_20.780392	0,00122	1074	Class I retrotransposons/LTR/Gypsy/Gypsy
CL203	0,12328	3,04841	Dincompta_Gypsy-6_Dwil-I_1	Dlutzii_NODE_6647_length_7016_cov_63.766209	0,00001	1050	Class I retrotransposons/LTR/Gypsy/Gypsy
CL206	0,09767	2,41517	Dbromeliae_contig_302263	Dbromeliae_sp_IV_CL173_Contig1	0,00052	1044	Class I retrotransposons/LTR/Gypsy/Gypsy
CL68	0,12781	3,16045	Dincompta_GYPSY2_I	Dlutzii_Contig58_3	0,00000	1035	Class I retrotransposons/LTR/Gypsy/Gypsy
CL209	0,12356	3,05528	Dbromelioides_NODE_5527_length_1656_cov_541.810553	Dbromeliae_sp_IV_CL56_Contig1	0,03384	1029	Class I retrotransposons/LTR/Gypsy/Gypsy
CL213	0,09698	2,39823	Dincompta_Gypsy9-I_Dya_2	Dlutzii_NODE_12691_length_3509_cov_98.339965	0,00000	1005	Class I retrotransposons/LTR/Gypsy/Gypsy
CL36	0,10833	2,67882	Dincompta_Gypsy-12_Dinc_1	Dlutzii_NODE_3983_length_11008_cov_77.421086	0,00057	987	Class I retrotransposons/LTR/Gypsy/Gypsy
CL218	0,24215	5,98798	Dincompta_NODE_4259_length_4305_cov_10.786504	Dlutzii_NODE_7210_length_6476_cov_210.632890	0,00001	987	Class I retrotransposons/LTR/Gypsy/Gypsy
CL219	0,36028	8,90904	Dbromelioides_NODE_3554_length_3710_cov_197.416598	Dincompta_Gypsy-42_Dinc	0,00407	983	Class I retrotransposons/LTR/Gypsy/Gypsy
CL227	0,19170	4,74030	Dincompta_Gypsy-5_Dinc	Dbromeliae_sp_IV_NODE_9660_length_1325_cov_8.579867	0,00000	963	Class I retrotransposons/LTR/Gypsy/Gypsy
CL234	0,04847	1,19860	Dbromelioides_0141Contig1	Dbromeliae_sp_IV_NODE_11231_length_1199_cov_11.646840	0,00327	945	Class I retrotransposons/LTR/Gypsy/Gypsy
CL248	0,17806	4,40310	Dincompta_Gypsy-34_Dinc	Dlutzii_NODE_34949_length_1250_cov_48.300259	0,00022	927	Class I retrotransposons/LTR/Gypsy/Gypsy
CL246	0,25945	6,41564	Dincompta_Gypsy-62_Dwil-I_2	Dlutzii_NODE_4543_length_9925_cov_67.988611	0,01022	918	Class I retrotransposons/LTR/Gypsy/Gypsy
CL252	0,03590	0,88762	Dbromelioides_NODE_3633_length_3514_cov_174.824541	Dbromeliae_sp_IV_NODE_4136_length_4099_cov_20.803320	0,00028	912	Class I retrotransposons/LTR/Gypsy/Gypsy
CL252	0,09908	2,45016	Dbromeliae_contig_304324	Dbromeliae_sp_IV_NODE_4136_length_4099_cov_20.803320	0,00220	912	Class I retrotransposons/LTR/Gypsy/Gypsy
CL251	0,12413	3,06939	Dincompta_Gypsy-37_Dinc	Dbromeliae_sp_IV_NODE_15037_length_1031_cov_76.210352	0,00088	906	Class I retrotransposons/LTR/Gypsy/Gypsy
CL256	0,05226	1,29219	Dbromelioides_NODE_4734_length_2106_cov_106.011864	Dbromeliae_sp_IV_CL103_Contig2	0,00057	894	Class I retrotransposons/LTR/Gypsy/Gypsy
CL268	0,11813	2,92110	Dincompta_Gypsy-10_Dinc	Dbromeliae_sp_IV_NODE_2154_length_12706_cov_17.602956	0,00002	855	Class I retrotransposons/LTR/Gypsy/Gypsy
CL09	0,11892	2,94071	Dincompta_NODE_88_length_11151_cov_5.004146	Dlutzii_NODE_2602_length_14880_cov_99.097370	0,00000	843	Class I retrotransposons/LTR/Gypsy/Gypsy
CL242	0,12142	3,00248	Dbromeliae_contig_302336	Dbromeliae_sp_IV_NODE_3752_length_4909_cov_11.975763	0,04772	843	Class I retrotransposons/LTR/Gypsy/Gypsy
CL269	0,27692	6,84755	Dincompta_NODE_104136_length_843_cov_80.837150	Dlutzii_NODE_4543_length_9925_cov_67.988611	0,00079	840	Class I retrotransposons/LTR/Gypsy/Gypsy
CL113	0,12111	2,99469	Dbromelioides_0040Contig2	Dbromeliae_sp_IV_NODE_5844_length_2311_cov_12.001828	0,04242	822	Class I retrotransposons/LTR/Gypsy/Gypsy
CL285	0,17780	4,39675	Dbromelioides_NODE_3045_length_5105_cov_29.018917	Dlutzii_NODE_6220_length_7445_cov_62.955806	0,00000	807	Class I retrotransposons/LTR/Gypsy/Gypsy
CL285	0,08125	2,00918	Dlutzii_NODE_6220_length_7445_cov_62.955806	Dbromeliae_sp_IV_NODE_4717_length_3266_cov_23.064270	0,00039	807	Class I retrotransposons/LTR/Gypsy/Gypsy

CL291	0,04263	1,05424	Dbromeliae_Contig0053_21	Dbromeliae_sp_IV_NODE_5133_length_2831_cov_12.865953	0,02031	804	Class I retrotransposons/LTR/Gypsy/Gypsy
CL286	0,01303	0,32225	Dbromelioides_NODE_11963_length_800_cov_228.364017	Dbromeliae_sp_IV_NODE_4777_length_3187_cov_149.070496	0,00012	801	Class I retrotransposons/LTR/Gypsy/Gypsy
CL287	0,11401	2,81919	Dbromelioides_NODE_3633_length_3514_cov_174.824541	Dbromeliae_sp_IV_NODE_4136_length_4099_cov_20.803320	0,00649	801	Class I retrotransposons/LTR/Gypsy/Gypsy
CL302	0,12103	2,99277	Dbromelioides_NODE_3633_length_3514_cov_174.824541	Dbromeliae_sp_IV_NODE_2840_length_7997_cov_17.219964	0,00970	753	Class I retrotransposons/LTR/Gypsy/Gypsy
CL304	0,00000	0,00000	Dincompta_Gypsy-24_Dwil-I	Dlutzii_NODE_25704_length_1607_cov_847.825858	0,00001	750	Class I retrotransposons/LTR/Gypsy/Gypsy
CL10	0,15375	3,80185	Dbromelioides_NODE_3435_length_3982_cov_30.480893	Dlutzii_NODE_5969_length_7739_cov_62.463781	0,00000	726	Class I retrotransposons/LTR/Gypsy/Gypsy
CL328	0,20257	5,00912	Dincompta_NODE_134864_length_697_cov_52.518750	Dlutzii_NODE_10533_length_4343_cov_79.218015	0,00214	708	Class I retrotransposons/LTR/Gypsy/Gypsy
CL325	0,00843	0,20845	Dbromelioides_NODE_5308_length_1755_cov_153.382177	Dbromeliae_sp_IV_NODE_24323_length_727_cov_188.701987	0,00004	705	Class I retrotransposons/LTR/Gypsy/Gypsy
CL325	0,12249	3,02886	Dbromeliae_contig_81785	Dbromeliae_sp_IV_NODE_24323_length_727_cov_188.701987	0,00506	705	Class I retrotransposons/LTR/Gypsy/Gypsy
CL341	0,06381	1,57790	Dbromelioides_NODE_3602_length_3579_cov_8.449943	Dbromeliae_sp_IV_NODE_4717_length_3266_cov_23.064270	0,00161	678	Class I retrotransposons/LTR/Gypsy/Gypsy
CL345	0,05917	1,46305	Dincompta_Gypsy-19_Dinc	Dlutzii_NODE_43831_length_1071_cov_376.764286	0,00000	678	Class I retrotransposons/LTR/Gypsy/Gypsy
CL362	0,00000	0,00000	Dbromelioides_0131Contig2	Dbromeliae_sp_IV_NODE_7565_length_1648_cov_161.480000	0,01866	654	Class I retrotransposons/LTR/Gypsy/Gypsy
CL371	0,07725	1,91027	Dbromelioides_NODE_4265_length_2551_cov_56.627229	Dbromeliae_sp_IV_NODE_3181_length_6591_cov_21.444960	0,00412	654	Class I retrotransposons/LTR/Gypsy/Gypsy
CL368	0,19062	4,71360	Dincompta_Gypsy-62_Dwil-I_1	Dlutzii_NODE_6274_length_7409_cov_55.949303	0,00057	651	Class I retrotransposons/LTR/Gypsy/Gypsy
CL369	0,04905	1,21290	Dincompta_Gypsy-37_Dinc	Dbromeliae_sp_IV_NODE_27897_length_647_cov_125.517176	0,00004	648	Class I retrotransposons/LTR/Gypsy/Gypsy
CL376	0,10924	2,70131	Dincompta_Gypsy-35_Dinc	Dlutzii_NODE_2067_length_17134_cov_53.752626	0,00032	645	Class I retrotransposons/LTR/Gypsy/Gypsy
CL383	0,07623	1,88503	Dbromelioides_NODE_9294_length_971_cov_228.393018	Dbromeliae_sp_IV_NODE_5133_length_2831_cov_12.865953	0,02291	642	Class I retrotransposons/LTR/Gypsy/Gypsy
CL383	0,03709	0,91707	Dbromeliae_Contig0053_30	Dbromeliae_sp_IV_NODE_5133_length_2831_cov_12.865953	0,00373	642	Class I retrotransposons/LTR/Gypsy/Gypsy
CL381	0,28515	7,05108	Dincompta_Gypsy-1_Dinc	Dlutzii_NODE_7399_length_6305_cov_45.289025	0,00053	633	Class I retrotransposons/LTR/Gypsy/Gypsy
CL382	0,13989	3,45932	Dincompta_Gypsy-6_Dwil-I_2	Dlutzii_NODE_8353_length_5620_cov_53.385241	0,00000	633	Class I retrotransposons/LTR/Gypsy/Gypsy
CL395	0,13523	3,34401	Dbromelioides_NODE_7681_length_1117_cov_64.190522	Dbromeliae_sp_IV_NODE_4835_length_3129_cov_127.515635	0,02196	624	Class I retrotransposons/LTR/Gypsy/Gypsy
CL162	0,32155	7,95122	Dincompta_Gypsy-37_Dinc	Dlutzii_NODE_13695_length_3218_cov_147.804285	0,02113	621	Class I retrotransposons/LTR/Gypsy/Gypsy
CL162	0,05538	1,36939	Dincompta_Gypsy-37_Dinc	Dbromeliae_sp_IV_NODE_29615_length_618_cov_153.503030	0,00006	621	Class I retrotransposons/LTR/Gypsy/Gypsy
CL162	0,26889	6,64920	Dlutzii_NODE_13695_length_3218_cov_147.804285	Dbromeliae_sp_IV_NODE_29615_length_618_cov_153.503030	0,00432	621	Class I retrotransposons/LTR/Gypsy/Gypsy
CL399	0,17589	4,34946	Dincompta_NODE_7088_length_3566_cov_40.536905	Dlutzii_NODE_10533_length_4343_cov_79.218015	0,00002	618	Class I retrotransposons/LTR/Gypsy/Gypsy
CL99	0,14006	3,46334	Dincompta_NODE_3156_length_4750_cov_6.945664	Dlutzii_NODE_9280_length_5022_cov_36.809369	0,00037	1572	Class I retrotransposons/LTR/Gypsy/Gypsy
CL172	0,04495	1,11141	Dbromelioides_0095Contig7	Dbromeliae_sp_IV_NODE_6197_length_2134_cov_85.794132	0,00046	1173	Class I retrotransposons/LTR/Gypsy/GTWIN
CL172	0,07343	1,81583	Dbromeliae_contig_189433	Dbromeliae_sp_IV_NODE_6197_length_2134_cov_85.794132	0,00464	1173	Class I retrotransposons/LTR/Gypsy/GTWIN
CL84	0,07125	1,76194	Dbromelioides_NODE_6993_length_1232_cov_118.086162	Dincompta_TABOR_Dinc_3	0,00018	1233	Class I retrotransposons/LTR/Gypsy/TABOR
CL84	0,10707	2,64752	Dbromelioides_NODE_6993_length_1232_cov_118.086162	Dlutzii_Contig21_3	0,00008	1233	Class I retrotransposons/LTR/Gypsy/TABOR
CL84	0,07611	1,88208	Dincompta_TABOR_Dinc_3	Dlutzii_Contig21_3	0,00079	1233	Class I retrotransposons/LTR/Gypsy/TABOR
CL84	0,07299	1,80485	Dbromeliae_contig_302254	Dincompta_TABOR_Dinc_3	0,00001	1233	Class I retrotransposons/LTR/Gypsy/TABOR
CL84	0,11479	2,83862	Dbromeliae_contig_302254	Dlutzii_Contig21_3	0,00004	1233	Class I retrotransposons/LTR/Gypsy/TABOR
CL161	0,20261	5,01005	Dbromelioides_NODE_3010_length_5263_cov_275.798263	Dincompta_TABOR_Dinc_3	0,00042	1272	Class I retrotransposons/LTR/Gypsy/TABOR
CL161	0,20268	5,01175	Dbromelioides_NODE_3010_length_5263_cov_275.798263	Dlutzii_Contig21_3	0,00006	1272	Class I retrotransposons/LTR/Gypsy/TABOR
CL161	0,15342	3,79367	Dincompta_TABOR_Dinc_3	Dlutzii_Contig21_3	0,00446	1272	Class I retrotransposons/LTR/Gypsy/TABOR
CL85	0,07104	1,75673	Dincompta_NODE_35559_length_1655_cov_0,711334	Dlutzii_NODE_9360_length_4973_cov_1.490987	0,00032	1647	Class I retrotransposons/LINE/R1
CL378	0,05244	1,29675	Dincompta_NODE_145118_length_660_cov_0,756219	Dlutzii_NODE_9360_length_4973_cov_1.490987	0,00453	636	Class I retrotransposons/LINE/R1
CL163	0,08290	2,05006	Dbromelioides_NODE_4238_length_2578_cov_38.349098	Dbromeliae_sp_IV_CL106_Contig1	0,00047	1257	Class I retrotransposons/LINE/RTE
CL12	0,10976	2,71419	Dbromelioides_0102Contig1	Dlutzii_NODE_6500_length_7166_cov_63.366502	0,00000	3069	Class I retrotransposons/LINE/Jockey/Jockey
CL233	0,10578	2,61577	Dbromelioides_NODE_2687_length_6897_cov_106.741415	Dlutzii_NODE_5795_length_7979_cov_71.083418	0,00000	951	Class I retrotransposons/LINE/Jockey/Jockey
CL116	0,16786	4,15084	Dbromelioides_NODE_3509_length_3796_cov_139.746297	Dlutzii_NODE_3080_length_13259_cov_47.822828	0,00000	872	Class I retrotransposons/LINE/Jockey/Jockey
CL92	0,06933	1,71447	Dbromeliae_contig_144511	Dincompta_Jocey-4_Dinc_1	0,00000	618	Class I retrotransposons/LINE/Jockey/Jockey
CL160	0,06369	1,57484	Dbromelioides_NODE_3676_length_3433_cov_32.977910	Dincompta_NODE_821_length_6860_cov_19.107143	0,00000	1284	Class I retrotransposons/LINE/Jockey/G3
CL160	0,12061	2,98241	Dincompta_NODE_821_length_6860_cov_19.107143	Dbromeliae_sp_IV_NODE_3606_length_5275_cov_12.950311	0,00000	1284	Class I retrotransposons/LINE/Jockey/G3
CL39	0,15696	3,88119	Dbromelioides_NODE_2945_length_5514_cov_39.323881	Dincompta_NODE_11155_length_2953_cov_5.022445	0,00000	2238	Class I retrotransposons/LINE/I2
CL39	0,14430	3,56815	Dbromelioides_NODE_2945_length_5514_cov_39.323881	Dlutzii_NODE_8381_length_5600_cov_52.048829	0,00000	2238	Class I retrotransposons/LINE/I2
CL39	0,08731	2,15894	Dincompta_NODE_11155_length_2953_cov_5.022445	Dlutzii_NODE_8381_length_5600_cov_52.048829	0,00003	2238	Class I retrotransposons/LINE/I2
CL83	0,06832	1,68942	Dincompta_Mariner-9_Dan	Dlutzii_NODE_23092_length_1787_cov_427.541274	0,00001	1407	Class II DNA transposons - Subclass 1/TIR/Mariner/Mariner
CL83	0,01910	0,47243	Dincompta_Mariner-9_Dan	Dbromeliae_sp_IV_NODE_3726_length_4959_cov_21.861869	0,00000	1407	Class II DNA transposons - Subclass 1/TIR/Mariner/Mariner
CL83	0,05657	1,39898	Dlutzii_NODE_23092_length_1787_cov_427.541274	Dbromeliae_sp_IV_NODE_3726_length_4959_cov_21.861869	0,00000	1407	Class II DNA transposons - Subclass 1/TIR/Mariner/Mariner
CL80	0,06003	1,48444	Dbromelioides_NODE_5271_length_1770_cov_79.474807	Dincompta_SMAR18_1	0,00000	1677	Class II DNA transposons - Subclass 1/TIR/Mariner/SMAR
CL327	0,15761	3,89748	Dincompta_NODE_54328_length_1292_cov_10.626721	Dlutzii_NODE_5832_length_7920_cov_66.962192	0,00100	714	Class II DNA transposons - Subclass 1/TIR/Mariner/MINOS
CL312	0,15025	3,71550	Dbromelioides_NODE_13155_length_727_cov_66.169255	Dincompta_SMAR7_2	0,00000	726	Class II DNA transposons - Subclass 1/TIR/Mariner/SMAR
CL239	0,08303	2,05318	Dincompta_Mariner-4_Dinc	Dlutzii_NODE_4552_length_9895_cov_79.443696	0,00000	948	Class II DNA transposons - Subclass 1/TIR/Mariner/Mariner
CL220	0,00000	0,00000	Dincompta_SMAR25	Dbromeliae_sp_IV_NODE_11956_length_1157_cov_78.385880	0,00010	990	Class II DNA transposons - Subclass 1/TIR/Mariner/SMAR
CL217	0,19940	4,93087	Dincompta_PARISa_Dbu	Dbromeliae_sp_IV_NODE_5016_length_2959_cov_14.327221	0,00000	990	Class II DNA transposons - Subclass 1/TIR/Mariner/PARISa
CL216	0,15783	3,90274	Dincompta_Mariner-N7_Dinc	Dlutzii_NODE_4534_length_9941_cov_69.694112	0,00001	999	Class II DNA transposons - Subclass 1/TIR/Mariner/Mariner
CL212	0,10855	2,68428	Dbromelioides_0085Contig2	Dlutzii_NODE_3991_length_10995_cov_55.542003	0,00000	1023	Class II DNA transposons - Subclass 1/TIR/Mariner/Mariner
CL212	0,04981	1,23163	Dbromelioides_0085Contig2	Dbromeliae_sp_IV_NODE_7987_length_1558_cov_49.947735	0,00315	1023	Class II DNA transposons - Subclass 1/TIR/Mariner/Mariner
CL212	0,05926	1,46545	Dlutzii_NODE_3991_length_10995_cov_55.542003	Dbromeliae_sp_IV_NODE_7987_length_1558_cov_49.947735	0,00000	1023	Class II DNA transposons - Subclass 1/TIR/Mariner/Mariner
CL212	0,09386	2,32098	Dbromeliae_contig_305402	Dlutzii_NODE_3991_length_10995_cov_55.542003	0,00000	1023	Class II DNA transposons - Subclass 1/TIR/Mariner/Mariner
CL212	0,06120	1,51328	Dbromeliae_contig_305402	Dbromeliae_sp_IV_NODE_7987_length_1558_cov_49.947735	0,00214	1023	Class II DNA transposons - Subclass 1/TIR/Mariner/Mariner
CL200	0,21046	5,20416	Dbromelioides_0086Contig3	Dincompta_NODE_37072_length_1615_cov_8.223363	0,00000	1050	Class II DNA transposons - Subclass 1/TIR/Mariner/PARISa
CL200	0,26953	6,66494	Dincompta_NODE_37072_length_1615_cov_8.223363	Dbromeliae_sp_IV_NODE_5016_length_2959_cov_14.327221	0,00001	1050	Class II DNA transposons - Subclass 1/TIR/Mariner/PARISa
CL179	0,00250	0,06190	Dincompta_SMAR18_1	Dbromeliae_sp_IV_NODE_12513_length_1129_cov_60.114314	0,00000	1134	Class II DNA transposons - Subclass 1/TIR/Mariner/SMAR

CL152	0,00973	0,24066	Dbromelioides_NODE_6032_length_1476_cov_36.126346	Dincompta_SMAR25	0,00000	1302	Class II DNA transposons - Subclass 1/TIR/Mariner/SMAR
CL145	0,00000	0,00000	Dincompta_Mariner-3_Dinc	Dbromeliae_sp_IV_NODE_9127_length_1382_cov_351.545671	0,00000	1326	Class II DNA transposons - Subclass 1/TIR/Mariner/Mariner
CL264	0,29176	7,21454	Dincompta_hAT-1_Din	Dbromeliae_sp_IV_NODE_5089_length_2873_cov_39.054545	0,00061	858	Class II DNA transposons - Subclass 1/TIR/hAT/hAT
CL392	0,01925	0,47595	Dincompta_NODE_4718_length_4155_cov_5.265007	Dlutzii_Contig38_1	0,00004	633	Class II DNA transposons - Subclass 1/TIR/hAT/hAT
CL79	0,20936	5,17707	Dincompta_Hoin6	Dlutzii_NODE_2024_length_17349_cov_74.859601	0,00001	1698	Class II DNA transposons - Subclass 1/TIR/hAT/Hoin
CL126	0,01013	0,25050	Dincompta_Hoin3	Dbromeliae_sp_IV_NODE_4634_length_3371_cov_17.714901	0,00000	1419	Class II DNA transposons - Subclass 1/TIR/hAT/Hoin
CL333	0,37127	9,18083	Dbromelioides_NODE_5500_length_1669_cov_165.501892	Dincompta_Hoin9	0,00000	693	Class II DNA transposons - Subclass 1/TIR/hAT/Hoin
CL41	0,06521	1,61252	Dincompta_Homo7_2	Dbromeliae_sp_IV_NODE_5779_length_2354_cov_91.852084	0,00000	2130	Class II DNA transposons - Subclass 1/TIR/hAT/Homo
CL41	0,06964	1,72212	Dlutzii_NODE_9515_length_4877_cov_106.002716	Dbromeliae_sp_IV_NODE_5779_length_2354_cov_91.852084	0,00000	2130	Class II DNA transposons - Subclass 1/TIR/hAT/Homo
CL194	0,09485	2,34557	Dbromelioides_0167Contig4	Dlutzii_NODE_5039_length_9072_cov_35.020488	0,00000	1065	Class II DNA transposons - Subclass 1/TIR/hAT/Homo
CL211	0,04511	1,11550	Dincompta_NODE_7175_length_3549_cov_12.374857	Dlutzii_NODE_48318_length_1004_cov_172.220153	0,00002	1029	Class II DNA transposons - Subclass 1/TIR/hAT/Homo
CL43	0,05338	1,32006	Dbromelioides_NODE_2448_length_8663_cov_40.391841	Dincompta_NODE_11030_length_2966_cov_15.404467	0,00000	2078	Class II DNA transposons - Subclass 1/TIR/Transib
CL66	0,18516	4,57856	Dbromelioides_0040Contig12	Dincompta_NODE_734_length_7022_cov_73.507105	0,00037	1896	Class II DNA transposons - Subclass 1/TIR/Transib
CL132	0,16805	4,15547	Dincompta_Transib-5_Dan	Dlutzii_NODE_16409_length_2625_cov_102.883583	0,00008	1401	Class II DNA transposons - Subclass 1/TIR/Transib
CL178	0,12203	3,01765	Dincompta_NODE_1666_length_5719_cov_53.190678	Dlutzii_NODE_3574_length_11913_cov_150.547877	0,00001	1167	Class II DNA transposons - Subclass 1/TIR/Transib
CL272	0,12513	3,09428	Dincompta_Transib-5_Dan	Dlutzii_NODE_61947_length_834_cov_200.177658	0,00019	840	Class II DNA transposons - Subclass 1/TIR/Transib
CL281	0,06684	1,65293	Dbromeliae_contig_302225	Dlutzii_NODE_4818_length_9422_cov_56.437574	0,00000	834	Class II DNA transposons - Subclass 1/TIR/Transib
CL339	0,11375	2,81281	Dincompta_Transib-2_Dinc	Dlutzii_NODE_3574_length_11913_cov_150.547877	0,00004	690	Class II DNA transposons - Subclass 1/TIR/Transib
CL183	0,01845	0,45631	Dincompta_PiggyBac-N2_Dinc_1	Dbromeliae_sp_IV_NODE_12738_length_1119_cov_31.421687	0,00000	1122	Class II DNA transposons - Subclass 1/TIR/PiggyBAC
CL187	0,02757	0,68171	Dbromelioides_0187Contig1	Dincompta_NODE_15131_length_2573_cov_15.978546	0,00000	1098	Class II DNA transposons - Subclass 1/TIR/PiggyBAC
CL208	0,07161	1,77069	Dbromelioides_NODE_8663_length_1015_cov_909.051502	Dincompta_PiggyBac-N1_Dinc	0,00000	1017	Class II DNA transposons - Subclass 1/TIR/PiggyBAC
CL221	0,01717	0,42450	Dincompta_PiggyBac-N2_Dinc_1	Dbromeliae_sp_IV_NODE_7330_length_1710_cov_15.052930	0,00000	984	Class II DNA transposons - Subclass 1/TIR/PiggyBAC
CL255	0,02514	0,62174	Dincompta_PiggyBac-N2_Dinc_1	Dbromeliae_sp_IV_NODE_4020_length_4304_cov_21.627123	0,00000	885	Class II DNA transposons - Subclass 1/TIR/PiggyBAC
CL270	0,22416	5,54299	Dbromelioides_NODE_5847_length_1532_cov_248.191856	Dincompta_PiggyBac-N3_DinC	0,00001	837	Class II DNA transposons - Subclass 1/TIR/PiggyBAC
CL270	0,21310	5,26955	Dbromelioides_NODE_5847_length_1532_cov_248.191856	Dlutzii_NODE_57610_length_890_cov_41.429287	0,00000	837	Class II DNA transposons - Subclass 1/TIR/PiggyBAC
CL270	0,14178	3,50585	Dincompta_PiggyBac-N3_DinC	Dlutzii_NODE_57610_length_890_cov_41.429287	0,00001	837	Class II DNA transposons - Subclass 1/TIR/PiggyBAC
CL283	0,16715	4,13340	Dincompta_PiggyBac-N3_DinC	Dlutzii_NODE_13193_length_3363_cov_155.732579	0,00000	819	Class II DNA transposons - Subclass 1/TIR/PiggyBAC
CL64	0,10097	2,49671	Dbromelioides_0114Contig1	Dincompta_Harbinger-1_Dinc	0,00000	1953	Class II DNA transposons - Subclass 1/TIR/Harbinger
CL64	0,14278	3,53059	Dbromelioides_0114Contig1	Dlutzii_NODE_19294_length_2190_cov_90.600286	0,00000	1953	Class II DNA transposons - Subclass 1/TIR/Harbinger
CL64	0,16264	4,02188	Dincompta_Harbinger-1_Dinc	Dlutzii_NODE_19294_length_2190_cov_90.600286	0,00061	1953	Class II DNA transposons - Subclass 1/TIR/Harbinger
CL64	0,11175	2,76323	Dbromeliae_Contig0094_2	Dincompta_Harbinger-1_Dinc	0,00002	1953	Class II DNA transposons - Subclass 1/TIR/Harbinger
CL64	0,11655	2,88200	Dbromeliae_Contig0094_2	Dlutzii_NODE_19294_length_2190_cov_90.600286	0,00005	1953	Class II DNA transposons - Subclass 1/TIR/Harbinger
CL123	0,10575	2,61506	Dbromelioides_NODE_3164_length_4685_cov_91.081486	Dincompta_Harbinger-1_Dinc	0,00002	1428	Class II DNA transposons - Subclass 1/TIR/Harbinger
CL123	0,01370	0,33872	Dincompta_Harbinger-1_Dinc	Dbromeliae_sp_IV_NODE_6625_length_1950_cov_66.229885	0,00001	1428	Class II DNA transposons - Subclass 1/TIR/Harbinger
CL176	0,01384	0,34229	Dincompta_Harbinger-1_Dinc	Dbromeliae_sp_IV_NODE_12315_length_1139_cov_115.366142	0,00000	1161	Class II DNA transposons - Subclass 1/TIR/Harbinger
CL04	0,03367	0,83271	Dincompta_NODE_1489_length_5887_cov_8.296226	Dbromeliae_sp_IV_CL89_Contig3	0,00000	4662	Class II DNA transposons - Subclass 2/Helitron
CL25	0,01019	0,25193	Dincompta_NODE_697_length_7103_cov_38.374539	Dbromeliae_sp_IV_CL52_Contig7	0,00000	2550	Class II DNA transposons - Subclass 2/Helitron
CL31	0,00564	0,13952	Dbromelioides_CL1060106Contig2	Dbromeliae_sp_IV_CL113_Contig1	0,00037	2550	Class II DNA transposons - Subclass 2/Helitron
CL31	0,00930	0,22985	Dlutzii_NODE_17431_length_2455_cov_171.428934	Dbromeliae_sp_IV_CL113_Contig1	0,00000	2550	Class II DNA transposons - Subclass 2/Helitron
CL31	0,00363	0,08977	Dbromelioides_CL1060106Contig2	Dlutzii_NODE_17431_length_2455_cov_171.428934	0,00000	2490	Class II DNA transposons - Subclass 2/Helitron
CL53	0,12264	3,03254	Dincompta_NODE_14556_length_2620_cov_27.805772	Dlutzii_Contig77_1	0,00000	2079	Class II DNA transposons - Subclass 2/Helitron
CL65	0,03672	0,90811	Dincompta_NODE_417_length_8020_cov_21.711167	Dlutzii_Contig77_1	0,00000	1932	Class II DNA transposons - Subclass 2/Helitron
CL70	0,04059	1,00380	Dbromelioides_CL1060106Contig2	Dincompta_Helitron-1_BM_1	0,00000	1812	Class II DNA transposons - Subclass 2/Helitron
CL70	0,00156	0,03869	Dbromelioides_CL1060106Contig2	Dlutzii_NODE_22971_length_1797_cov_238.963072	0,00000	1812	Class II DNA transposons - Subclass 2/Helitron
CL70	0,04229	1,04582	Dincompta_Helitron-1_BM_1	Dlutzii_NODE_22971_length_1797_cov_238.963072	0,00005	1812	Class II DNA transposons - Subclass 2/Helitron
CL70	0,00000	0,00001	Dbromelioides_CL1060106Contig2	Dbromeliae_sp_IV_CL113_Contig1	0,00090	1812	Class II DNA transposons - Subclass 2/Helitron
CL70	0,04058	1,00350	Dincompta_Helitron-1_BM_1	Dbromeliae_sp_IV_CL113_Contig1	0,00001	1812	Class II DNA transposons - Subclass 2/Helitron
CL70	0,00156	0,03863	Dlutzii_NODE_22971_length_1797_cov_238.963072	Dbromeliae_sp_IV_CL113_Contig1	0,00001	1812	Class II DNA transposons - Subclass 2/Helitron
CL98	0,06741	1,66703	Dbromelioides_NODE_2247_length_11031_cov_72.941542	Dlutzii_NODE_4702_length_9622_cov_71.715979	0,00000	1569	Class II DNA transposons - Subclass 2/Helitron
CL109	0,13419	3,31827	Dincompta_Helitron-1_Dvir	Dlutzii_NODE_28472_length_1464_cov_217.005827	0,00003	1491	Class II DNA transposons - Subclass 2/Helitron
CL125	0,14249	3,52350	Dincompta_NODE_2001_length_5452_cov_7.846339	Dlutzii_Contig77_1	0,00001	1380	Class II DNA transposons - Subclass 2/Helitron
CL159	0,06842	1,69185	Dbromelioides_NODE_6834_length_1270_cov_1602.935973	Dincompta_Helitron-1_BM_1	0,00001	1272	Class II DNA transposons - Subclass 2/Helitron
CL159	0,00216	0,05341	Dbromelioides_NODE_6834_length_1270_cov_1602.935973	Dbromeliae_sp_IV_CL113_Contig1	0,00150	1272	Class II DNA transposons - Subclass 2/Helitron
CL159	0,06615	1,63564	Dincompta_Helitron-1_BM_1	Dbromeliae_sp_IV_CL113_Contig1	0,00003	1272	Class II DNA transposons - Subclass 2/Helitron
CL149	0,07004	1,73190	Dbromelioides_CL1060106Contig2	Dincompta_NODE_46517_length_1417_cov_8.767647	0,00001	1257	Class II DNA transposons - Subclass 2/Helitron
CL149	0,00000	0,00005	Dbromelioides_CL1060106Contig2	Dbromeliae_sp_IV_CL113_Contig1	0,00121	1257	Class II DNA transposons - Subclass 2/Helitron
CL149	0,07019	1,73573	Dincompta_NODE_46517_length_1417_cov_8.767647	Dbromeliae_sp_IV_CL113_Contig1	0,00004	1257	Class II DNA transposons - Subclass 2/Helitron
CL195	0,06002	1,48409	Dbromelioides_CL1060106Contig2	Dincompta_NODE_74405_length_1060_cov_8.027916	0,00001	1062	Class II DNA transposons - Subclass 2/Helitron
CL195	0,00263	0,06491	Dbromelioides_CL1060106Contig2	Dbromeliae_sp_IV_CL113_Contig1	0,00179	1062	Class II DNA transposons - Subclass 2/Helitron
CL195	0,06287	1,55474	Dincompta_NODE_74405_length_1060_cov_8.027916	Dbromeliae_sp_IV_CL113_Contig1	0,00008	1062	Class II DNA transposons - Subclass 2/Helitron
CL225	0,15733	3,89036	Dbromelioides_0027Contig9	Dbromeliae_sp_IV_NODE_3014_length_7165_cov_39.971457	0,01882	978	Class II DNA transposons - Subclass 2/Helitron
CL303	0,16416	4,05926	Dbromeliae_contig_305364	Dincompta_NODE_22160_length_2134_cov_3.160250	0,00000	756	Class II DNA transposons - Subclass 2/Helitron
CL319	0,03295	0,81479	Dbromelioides_NODE_5987_length_1491_cov_995.717330	Dbromeliae_sp_IV_NODE_11337_length_1193_cov_270.856075	0,00037	726	Class II DNA transposons - Subclass 2/Helitron
CL364	0,26824	6,63299	Dbromelioides_NODE_5461_length_1686_cov_1416.399251	Dincompta_Helitron-1_Rpr	0,00012	651	Class II DNA transposons - Subclass 2/Helitron
CL394	0,00931	0,23019	Dincompta_Helitron-1_Dinc	Dbromeliae_sp_IV_NODE_29726_length_616_cov_557.851927	0,00000	618	Class II DNA transposons - Subclass 2/Helitron
CL295	0,10613	2,62432	Dincompta_Polinton-N3_Dinc_1	Dlutzii_NODE_64688_length_778_cov_2595.170306	0,00002	783	Class II DNA transposons - Subclass 2/Maverick/Polinton

Table S6. Sum of HTT events inferred for each different superfamily of TEs. *Each HTT event sums 4, and is depicted by alternating colors in the table. When different scenarios are possible, both alternatives are considered and presented by the same color in the table.

Cluster	Species					Sum *	Scenarios	Element
	D. bromeliae	*D. bromelioides*	*D. bromeliae sp. IV*	*D. incompta*	*D. lutzii*			
CL00	0	2	2	0	0	4	*D. bromelioides →← D. bromeliae sp. IV*	BELPao
CL07	0	1	0	0	1	2	*D. bromelioides → D. lutzii*	BELPao
CL07	0,33	0,3	0,33	0	1	1,96	*D. lutzii → D. bromeliae* species group ancestral	BELPao
CL13	2	0	2	0	0	4	*D. bromeliae →← D. bromeliae sp. IV*	BELPao
CL16	0	2	2	0	0	4	*D. bromelioides →← D. bromeliae sp. IV*	BELPao
CL22	0	0	1	0	1	2	*D. bromeliae sp. IV → D. lutzii*	BELPao
CL22	0,33	0,33	0,33	0	1	1,99	*D. lutzii → D. bromeliae* species group ancestral	BELPao
CL129	2	0	2	0	0	4	*D. bromeliae →← D. bromeliae sp. IV*	BELPao
CL133	1	0	1	0	0	2	*D. bromelioides → D. bromeliae sp. IV*	BELPao
CL133	0,5	0,5	1	0	0	2	*D. bromeliae sp. IV → D. bromeliae and D. bromelioides ancestral*	BELPao
CL134	0	0	2	0	2	4	*D. bromeliae sp. IV →← D. lutzii*	BELPao
CL165	1	0	0	0	1	2	*D. bromeliae → D. lutzii*	BELPao
CL165	0,33	0,33	0,33	0	1	1,99	*D. lutzii → D. bromeliae* species group ancestral	BELPao
CL165	1	0	1	0	0	2	*D. bromeliae → D. bromeliae sp. IV*	BELPao
CL165	0,5	0,5	1	0	0	2	*D. bromeliae sp. IV → D. bromeliae and D. bromelioides ancestral*	BELPao
CL320	0	0	0	2	2	4	*D. incompta →← D. lutzii*	BELPao
CL370	0	2	2	0	0	4	*D. bromelioides →← D. bromeliae sp. IV*	BELPao
CL40	0	1	0	0	1	2	*D. bromelioides → D. lutzii*	BELPao
CL40	0,33	0,33	0,33	0	1	1,99	*D. lutzii → D. bromeliae* species group ancestral	BELPao
CL77	1	0	0	0	1	2	*D. bromeliae → D. lutzii*	BELPao
CL77	0,33	0,33	0,33	0	1	1,99	*D. lutzii → D. bromeliae* species group ancestral	BELPao
CL82	0	0	2	0	2	4	*D. bromeliae sp. IV →← D. lutzii*	BELPao
CL101	0	2	2	0	0	4	*D. bromelioides →← D. bromeliae sp. IV*	Copia
CL106	0	1	0	0	1	2	*D. bromelioides → D. lutzii*	Copia
CL106	0,33	0,33	0,33	0	1	1,99	*D. lutzii → D. bromeliae* species group ancestral	Copia
CL106	1	0	1	0	0	2	*D. bromeliae → D. bromeliae sp. IV*	Copia
CL106	0,5	0,5	1	0	0	2	*D. bromeliae sp. IV → D. bromeliae and D. bromelioides ancestral*	Copia
CL111	0	0	0	2	2	4	*D. incompta →← D. lutzii*	Copia
CL120	1	1	2	0	0	4	*D. bromeliae sp. IV → D. bromeliae and D. bromelioides ancestral*	Copia
CL136	0	2	0	2	0	4	*D. bromelioides →← D. incompta*	Copia
CL142	0	2	2	0	0	4	*D. bromelioides →← D. bromeliae sp. IV*	Copia
CL196	0	2	0	0	2	4	*D. bromelioides →← D. lutzii*	Copia
CL240	1	0	1	0	0	2	*D. bromeliae → D. bromeliae sp. IV*	Copia
CL240	0,5	0,5	1	0	0	2	*D. bromeliae sp. IV → D. bromeliae and D. bromelioides ancestral*	Copia
CL249	0	0	2	2	0	4	*D. bromeliae sp. IV →← D. incompta*	Copia
CL282	0	2	2	0	0	4	*D. bromelioides →← D. bromeliae sp. IV*	Copia
CL289	0	2	0	2	0	4	*D. bromelioides →← D. incompta*	Copia
CL310	2	2	0	0	0	4	*D. bromeliae →← D. bromelioides*	Copia
CL313	1	0	0	0	1	2	*D. bromeliae → D. lutzii*	Copia
CL313	0,5	0,5	0	0	1	2	*D. lutzii → D. bromeliae* and *D. bromelioides* ancestral	Copia
CL315	0	2	0	0	2	4	*D. bromelioides →← D. lutzii*	Copia
CL337	0	2	0	0	2	4	*D. bromelioides →← D. lutzii*	Copia
CL342	0	2	0	2	0	4	*D. bromelioides →← D. incompta*	Copia

CL352	2	0	2	0	0	4	*D. bromeliae →← D. bromeliae sp. IV*	Copia
CL390	0	2	2	0	0	4	*D. bromelioides →← D. bromeliae sp. IV*	Copia
CL391	0	2	0	0	2	4	*D. bromelioides →← D. lutzii*	Copia
CL62	0	0	2	2	0	4	*D. bromeliae sp. IV →← D. incompta*	Copia
CL94	0	0	0	2	2	4	*D. incompta →← D. lutzii*	Copia
CL01	0	2	0	2	0	4	*D. bromelioides →← D. incompta*	Gypsy
CL01	0	2	0	0	2	4	*D. bromelioides →← D. lutzii*	Gypsy
CL01	0	0	0	2	2	4	*D. incompta →← D. lutzii*	Gypsy
CL06	0	0	2	2	0	4	*D. bromeliae sp. IV →← D. incompta*	Gypsy
CL09	0	0	0	2	2	4	*D. incompta →← D. lutzii*	Gypsy
CL10	0	2	0	0	2	4	*D. bromelioides →← D. lutzii*	Gypsy
CL11	0	0	0	2	2	4	*D. incompta →← D. lutzii*	Gypsy
CL113	0	2	2	0	0	4	*D. bromelioides →← D. bromeliae sp. IV*	Gypsy
CL117	0	2	0	0	2	4	*D. bromelioides →← D. lutzii*	Gypsy
CL131	0	0	0	2	2	4	*D. incompta →← D. lutzii*	Gypsy
CL151	0	2	0	2	0	4	*D. bromelioides →← D. incompta*	Gypsy
CL162	0	0	2	2	0	4	*D. bromeliae sp. IV →← D. incompta*	Gypsy
CL162	0	0	0	2	2	4	*D. incompta →← D. lutzii*	Gypsy
CL162	0	0	2	0	2	4	*D. bromeliae sp. IV →← D. lutzii*	Gypsy
CL171	0	0	0	2	2	4	*D. incompta →← D. lutzii*	Gypsy
CL174	0	0	0	2	2	4	*D. incompta →← D. lutzii*	Gypsy
CL184	0	0	2	2	0	4	*D. bromeliae sp. IV →← D. incompta*	Gypsy
CL19	0	2	0	0	2	4	*D. bromelioides →← D. lutzii*	Gypsy
CL190	0	2	2	0	0	4	*D. bromelioides →← D. bromeliae sp. IV*	Gypsy
CL193	0	2	2	0	0	4	*D. bromelioides →← D. bromeliae sp. IV*	Gypsy
CL203	0	0	0	2	2	4	*D. incompta →← D. lutzii*	Gypsy
CL206	2	0	2	0	0	4	*D. bromeliae →← D. bromeliae sp. IV*	Gypsy
CL209	0	2	2	0	0	4	*D. bromelioides →← D. bromeliae sp. IV*	Gypsy
CL213	0	0	0	2	2	4	*D. incompta →← D. lutzii*	Gypsy
CL218	0	0	0	2	2	4	*D. incompta →← D. lutzii*	Gypsy
CL219	0	2	0	2	0	4	*D. bromelioides →← D. incompta*	Gypsy
CL227	0	0	2	2	0	4	*D. bromeliae sp. IV →← D. incompta*	Gypsy
CL234	0	2	2	0	0	4	*D. bromelioides →← D. bromeliae sp. IV*	Gypsy
CL242	2	0	2	0	0	4	*D. bromeliae →← D. bromeliae sp. IV*	Gypsy
CL246	0	0	0	2	2	4	*D. incompta →← D. lutzii*	Gypsy
CL248	0	0	0	2	2	4	*D. incompta →← D. lutzii*	Gypsy
CL251	0	0	2	2	0	4	*D. bromeliae sp. IV →← D. incompta*	Gypsy
CL252	0	1	1	0	0	2	*D. bromelioides → D. bromeliae sp. IV*	Gypsy
CL252	0,5	0,5	1	0	0	2	*D. bromeliae sp. IV →D. bromeliae and D. bromelioides ancestral*	Gypsy
CL256	0	2	2	0	0	4	*D. bromelioides →← D. bromeliae sp. IV*	Gypsy
CL26	0	0	0	2	2	4	*D. incompta →← D. lutzii*	Gypsy
CL268	0	0	2	2	0	4	*D. bromeliae sp. IV →← D. incompta*	Gypsy
CL269	0	0	0	2	2	4	*D. incompta →← D. lutzii*	Gypsy
CL285	0,33	0,33	0,33	0	1	1,99	*D. lutzii → D. bromelioides and D. bromeliae sp. IV ancestral*	Gypsy
CL285	0	0	1	0	1	2	*D. bromeliae sp. IV → D. lutzii*	Gypsy
CL286	0	2	2	0	0	4	*D. bromelioides →← D. bromeliae sp. IV*	Gypsy
CL287	0	2	2	0	0	4	*D. bromelioides →← D. bromeliae sp. IV*	Gypsy
CL291	2	0	2	0	0	4	*D. bromeliae →← D. bromeliae sp. IV*	Gypsy
CL30	2	0	2	0	0	4	*D. bromeliae →← D. bromeliae sp. IV*	Gypsy
CL302	0	2	2	0	0	4	*D. bromelioides →← D. bromeliae sp. IV*	Gypsy

CL304	0	0	0	2	2	4	*D. incompta* →← *D. lutzii*	Gypsy
CL325	0	2	2	0	0	4	*D. bromelioides* →← *D. bromeliae sp. IV*	Gypsy
CL328	0	0	0	2	2	4	*D. incompta* →← *D. lutzii*	Gypsy
CL341	0	2	2	0	0	4	*D. bromelioides* →← *D. bromeliae sp. IV*	Gypsy
CL345	0	0	0	2	2	4	*D. incompta* →← *D. lutzii*	Gypsy
CL36	0	0	0	2	2	4	*D. incompta* →← *D. lutzii*	Gypsy
CL362	0	2	2	0	0	4	*D. bromelioides* →← *D. bromeliae sp. IV*	Gypsy
CL368	0	0	0	2	2	4	*D. incompta* →← *D. lutzii*	Gypsy
CL369	0	0	2	2	0	4	*D. bromeliae sp. IV* →← *D. incompta*	Gypsy
CL37	0	0	0	2	2	4	*D. incompta* →← *D. lutzii*	Gypsy
CL37	0	0	2	2	0	4	*D. bromeliae sp. IV* →← *D. incompta*	Gypsy
CL37	0	0	2	0	2	4	*D. bromeliae sp. IV* →← *D. lutzii*	Gypsy
CL371	0	2	2	0	0	4	*D. bromelioides* →← *D. bromeliae sp. IV*	Gypsy
CL376	0	0	0	2	2	4	*D. incompta* →← *D. lutzii*	Gypsy
CL381	0	0	0	2	2	4	*D. incompta* →← *D. lutzii*	Gypsy
CL382	0	0	0	2	2	4	*D. incompta* →← *D. lutzii*	Gypsy
CL383	1	0	1	0	0	2	*D. bromeliae* → *D. bromeliae sp. IV*	Gypsy
CL383	0,5	0,5	1	0	0	2	*D. bromeliae sp. IV* → *D. bromeliae and D. bromelioides ancestral*	Gypsy
CL395	0	2	2	0	0	4	*D. bromelioides* →← *D. bromeliae sp. IV*	Gypsy
CL399	0	0	0	2	2	4	*D. incompta* →← *D. lutzii*	Gypsy
CL42	0	2	0	0	2	4	*D. bromelioides* →← *D. lutzii*	Gypsy
CL44	0	0	0	2	2	4	*D. incompta* →← *D. lutzii*	Gypsy
CL47	0	0	0	2	2	4	*D. incompta* →← *D. lutzii*	Gypsy
CL50	0	0	2	2	0	4	*D. bromeliae sp. IV* →← *D. incompta*	Gypsy
CL51	0	0	0	2	2	4	*D. incompta* →← *D. lutzii*	Gypsy
CL51	0,33	0,33	0,33	0	1	1,99	*D. lutzii* → *D. bromelioides and D. bromeliae sp. IV ancestral*	Gypsy
CL51	0	1	0	0	1	2	*D. bromelioides* → *D. lutzii*	Gypsy
CL51	0,33	0,33	0,33	1	0	1,99	*D. incompta* → *D. bromelioides and D. bromeliae sp. IV ancestral*	Gypsy
CL51	0	0	1	1	0	2	*D. bromeliae sp. IV* → *D. incompta*	Gypsy
CL52	0	2	0	0	2	4	*D. bromelioides* →← *D. lutzii*	Gypsy
CL61	0	0	0	2	2	4	*D. incompta* →← *D. lutzii*	Gypsy
CL68	0	0	0	2	2	4	*D. incompta* →← *D. lutzii*	Gypsy
CL71	0	0	0	2	2	4	*D. incompta* →← *D. lutzii*	Gypsy
CL74	0	0	2	2	0	4	*D. bromeliae sp. IV* →← *D. incompta*	Gypsy
CL76	0	0	0	2	2	4	*D. incompta* →← *D. lutzii*	Gypsy
CL321	2	0	0	2	0	4	*D. bromeliae* → *D. incompta*	Gypsy
CL99	0	0	0	2	2	4	*D. incompta* →← *D. lutzii*	Gypsy
CL172	0	1	1	0	0	2	*D. bromelioides* → *D. bromeliae sp. IV*	GTWIN
CL172	0,5	0,5	1	0	0	2	*D. bromeliae sp. IV* → *D. bromeliae and D. bromelioides ancestral*	GTWIN
CL161	0	2	0	2	0	4	*D. bromelioides* →← *D. incompta*	TABOR
CL161	0	2	0	0	2	4	*D. bromelioides* →← *D. lutzii*	TABOR
CL161	0	0	0	2	2	4	*D. incompta* →← *D. lutzii*	TABOR
CL84	0	0	0	2	2	4	*D. incompta* →← *D. lutzii*	TABOR
CL84	1	1	0	2	0	4	*D. incompta* → *D. bromeliae* and *D. bromelioides* ancestral	TABOR
CL84	0,5	0,5	0	0	1	2	*D. lutzii* → *D. bromeliae* and *D. bromelioides* ancestral	TABOR
CL84	0	1	0	0	1	2	D. bromelioides → *D. lutzii*	TABOR
CL85	0	0	0	2	2	4	*D. incompta* →← *D. lutzii*	R1
CL378	0	0	0	2	2	4	*D. incompta* →← *D. lutzii*	R1
CL163	0	2	2	0	0	4	*D. bromelioides* →← *D. bromeliae sp. IV*	RTE-2
CL12	0	2	0	0	2	4	*D. bromelioides* →← *D.lutzii*	Jockey

CL233	0	2	0	0	2	4	*D. bromelioides →← D.lutzii*	Jockey
CL116	0	2	0	0	2	4	*D. bromelioides →← D.lutzii*	Jockey
CL92	2	0	0	2	0	4	*D. bromeliae →← D. incompta*	Jockey
CL160	0,33	0,33	0,33	1	0	1,99	*D. incompta → D. bromelioides and D. bromeliae sp. IV ancestral*	G3
CL160	0	1	0	1	0	2	*D. bromelioides →← D. incompta*	G3
CL39	0	2	0	2	0	4	*D. bromelioides →← D. incompta*	I2
CL39	0	2	0	0	2	4	*D. bromelioides →← D. lutzii*	I2
CL39	0	0	0	2	2	4	*D. incompta →← D. lutzii*	I2
CL145	0	0	2	2	0	4	*D. bromeliae sp. IV →← D. incompta*	Mariner
CL212	0,5	0,5	1	0	0	2	*D. bromeliae sp. IV → D. bromeliae and D. bromelioides ancestral*	Mariner
CL212	0	1	1	0	0	2	*D. bromelioides → D. bromeliae sp. IV*	Mariner
CL212	0	0	1	0	1	2	*D. bromeliae sp. IV → D. lutzii*	Mariner
CL212	0,33	0,33	0,33	0	1	1,99	*D. lutzii → D. bromeliae* species group ancestral	Mariner
CL216	0	0	0	2	2	4	*D. incompta →← D. lutzii*	Mariner
CL239	0	0	0	2	2	4	*D. incompta →← D. lutzii*	Mariner
CL83	0	0	0	2	2	4	*D. incompta → D. lutzii*	Mariner
CL83	0	0	2	2	0	4	*D. bromeliae sp. IV ← D. incompta*	Mariner
CL83	0	0	2	0	2	4	*D. bromeliae sp. IV →← D. lutzii*	Mariner
CL327	0	0	0	2	2	4	*D. incompta →← D. lutzii*	MINOS
CL200	0,33	0,33	0,33	1	0	1,99	*D. incompta → D. bromelioides and D. bromeliae sp. IV ancestral*	PARISa
CL200	0	1	0	1	0	2	*D. bromelioides → D. incompta*	PARISa
CL217	0	0	2	2	0	4	*D. bromeliae sp. IV →← D. incompta*	PARISa
CL80	0	2	0	2	0	4	*D. bromelioides →← D. incompta*	SMAR
CL152	0	2	0	2	0	4	*D. bromelioides →← D. incompta*	SMAR
CL179	0	0	2	2	0	4	*D. bromeliae sp. IV →← D. incompta*	SMAR
CL220	0	0	2	2	0	4	*D. bromeliae sp. IV →← D. incompta*	SMAR
CL312	0	2	0	2	0	4	*D. bromelioides →← D. incompta*	SMAR
CL264	0	0	2	2	0	4	*D. bromeliae sp. IV →← D. incompta*	hAt
CL392	0	0	0	2	2	4	*D. incompta →← D. lutzii*	hAt
CL79	0	0	0	2	2	4	*D. incompta →← D. lutzii*	Hoin
CL126	0	0	2	2	0	4	*D. bromeliae sp. IV →← D. incompta*	Hoin
CL333	0	2	0	2	0	4	*D. bromelioides →← D. incompta*	Hoin
CL41	0	0	2	2	0	4	*D. bromeliae sp. IV →← D. incompta*	Homo
CL41	0	0	0	2	2	4	*D. incompta →← D. lutzii*	Homo
CL194	0	2	0	0	2	4	*D. bromelioides →← D. lutzii*	Homo
CL211	0	0	0	2	2	4	*D. incompta →← D. lutzii*	Homo
CL132	0	0	0	2	2	4	*D. incompta →← D. lutzii*	Transib
CL178	0	0	0	2	2	4	*D. incompta →← D. lutzii*	Transib
CL272	0	0	0	2	2	4	*D. incompta →← D. lutzii*	Transib
CL281	2	0	0	0	2	4	*D. bromeliae →← D. lutzii*	Transib
CL339	0	0	0	2	2	4	*D. incompta →← D. lutzii*	Transib
CL43	0	2	0	2	0	4	*D. bromeloides →← D. incompta*	Transib
CL66	0	2	0	2	0	4	*D. bromelioides →← D. incompta*	Transib
CL183	0	0	2	2	0	4	*D. bromeliae sp. IV →← D. incompta*	PiggyBAC
CL187	0	2	0	2	0	4	*D. bromelioides →← D. incompta*	PiggyBAC
CL208	0	2	0	2	0	4	*D. bromelioides →← D. incompta*	PiggyBAC
CL221	0	0	2	2	0	4	*D. bromeliae sp. IV →← D. incompta*	PiggyBAC
CL255	0	0	2	2	0	4	*D. bromeliae sp. IV →← D. incompta*	PiggyBAC
CL270	0	2	0	2	0	4	*D. bromelioides →← D. incompta*	PiggyBAC
CL270	0	2	0	0	2	4	*D. bromelioides →← D. lutzii*	PiggyBAC

CL270	0	0	0	2	2	4	*D. incompta* →← *D. lutzii*	PiggyBAC
CL283	0	0	0	2	2	4	*D. incompta* →← *D. lutzii*	PiggyBAC
CL123	0,33	0,33	0,33	1	0	1,99	*D. incompta* → *D. bromelioides and D. bromeliae sp. IV ancestral*	Harbinger
CL123	0	0	1	1	0	2	*D. bromeliae sp. IV* → *D. incompta*	Harbinger
CL176	0	0	2	2	0	4	*D. bromeliae sp. IV* →← *D. incompta*	Harbinger
CL64	0	0	0	2	2	4	*D. incompta* →← *D. lutzii*	Harbinger
CL64	0,5	0,5	0	1	0	2	*D. incompta* → *D. bromeliae* and *D. bromelioides* ancestral	Harbinger
CL64	0	1	0	1	0	2	*D. bromelioides* →*D. incompta*	Harbinger
CL64	0,5	0,5	0	0	1	2	*D. lutzii* → *D. bromeliae* and *D. bromelioides* ancestral	Harbinger
CL64	0	1	0	0	1	2	*D. bromelioides* →*D. lutzii*	Harbinger
CL04	0	0	2	2	0	4	*D. bromeliae sp. IV* →← *D. incompta*	Helitron
CL109	0	0	0	2	2	4	*D. incompta* →← *D. lutzii*	Helitron
CL125	0	0	0	2	2	4	*D. incompta* →← *D. lutzii*	Helitron
CL149	0	2	0	2	0	4	*D. bromelioides* →← *D. incompta*	Helitron
CL149	0	2	2	0	0	4	*D. bromelioides* →← *D. bromeliae sp. IV*	Helitron
CL149	0	0	2	2	0	4	*D. bromeliae sp. IV* →← *D. incompta*	Helitron
CL159	0	2	0	2	0	4	*D. bromelioides* →← *D. incompta*	Helitron
CL159	0	2	2	0	0	4	*D. bromelioides* →← *D. bromeliae sp. IV*	Helitron
CL159	0	0	2	2	0	4	*D. bromeliae sp. IV* →← *D. incompta*	Helitron
CL195	0	2	0	2	0	4	*D. bromelioides* →← *D. incompta*	Helitron
CL195	0	2	2	0	0	4	*D. bromelioides* →← *D. bromeliae sp. IV*	Helitron
CL195	0	0	2	2	0	4	*D. bromeliae sp. IV* →← *D. incompta*	Helitron
CL225	0	2	2	0	0	4	*D. bromelioides* →← *D. bromeliae sp. IV*	Helitron
CL25	0	0	2	2	0	4	*D. bromeliae sp. IV* →← *D. incompta*	Helitron
CL303	2	0	0	2	0	4	*D. bromeliae* →← *D. incompta*	Helitron
CL31	0	2	2	0	0	4	*D. bromelioides* →← *D. bromeliae sp. IV*	Helitron
CL31	0	0	2	0	2	4	*D. bromeliae sp. IV* →← *D. lutzii*	Helitron
CL31	0	2	0	0	2	4	*D. bromelioides* →← *D. lutzii*	Helitron
CL319	0	2	2	0	0	4	*D. bromelioides* →← *D. bromeliae sp. IV*	Helitron
CL364	0	2	0	2	0	4	*D. bromelioides* →← *D. incompta*	Helitron
CL394	0	0	2	2	0	4	*D. bromeliae sp. IV* →← *D. incompta*	Helitron
CL53	0	0	0	2	2	4	*D. incompta* →← *D. lutzii*	Helitron
CL65	0	0	0	2	2	4	*D. incompta* →← *D. lutzii*	Helitron
CL70	0	2	2	0	0	4	*D. bromelioides* →← *D. bromeliae sp. IV*	Helitron
CL70	0	2	0	2	0	4	*D. bromelioides* →← *D. incompta*	Helitron
CL70	0	0	2	2	0	4	*D. bromeliae sp. IV* →← *D. incompta*	Helitron
CL70	0	2	0	0	2	4	*D. bromelioides* →← *D. lutzii*	Helitron
CL70	0	0	2	0	2	4	*D. bromeliae sp. IV* →← *D. lutzii*	Helitron
CL70	0	0	0	2	2	4	*D. incompta* →← *D. lutzii*	Helitron
CL98	0	2	0	0	2	4	*D. bromelioides* →← *D. lutzii*	Helitron
CL95	0	0	0	2	2	4	*D. incompta* →← *D. lutzii*	Polinton
Total	44,29	168,26	175,29	246	202	835,84	209 horizontal transfer events	

Table S7. Input table used on GLM to estimate the putative influence of abiotic niche overlap, geographic overlap, biotic niche overlap, generalization degree and phylogenetic signal (predictor variables) on the potential for HTT among FBDs (as estimated with the Müller index, which was used as the response variable).

target	effect	abiotic_niche_overlap	geographic_overlap	biotic_niche_overlap	niche_breadth	phylogenetic_signal
Dbromelioides	0,0545064	0,8441641	0,220337	0,04081633	0,0714	2,6559
Dbromeliae sp IV	0,0529092	0,8697461	0,000000	0	0,0714	5,3531
Dincompta	0,0354597	0,5824017	0,442238	0	0,0714	21,8277
Dlutzii	0,0529646	0,7927191	0,488252	0	0,0714	35,4149
Dbromeliae	0,2206212	0,8441641	0,139522	4,08E-02	0,5833	2,6559
Dbromeliae sp IV	0,2043975	0,7749557	1,000000	4,08E-02	0,5833	5,3531
Dincompta	0,1924454	0,5238796	0,312477	1,11E-16	0,5833	21,8277
Dlutzii	0,18493	0,7195121	0,292147	1,63E-01	0,5833	35,4149
Dbromeliae	0,2191954	0,8697461	0,000000	0	0,0476	5,3531
Dbromelioides	0,2092069	0,7749557	0,012288	0,04081633	0,0476	5,3531
Dincompta	0,1900089	0,6548037	0,004379	0	0,0476	21,8277
Dlutzii	0,1932683	0,7680561	0,004308	0	0,0476	35,4149
Dbromeliae	0,2076927	0,5824017	0,784856	0,00E+00	0,1071	21,8277
Dbromelioides	0,2784798	0,5238796	0,875785	1,11E-16	0,1071	21,8277
Dbromeliae sp IV	0,2686333	0,6548037	0,998727	0,00E+00	0,1071	21,8277
Dlutzii	0,2942229	0,6518382	0,842354	0,00E+00	0,1071	35,4149
Dbromeliae	0,2547343	0,7927191	0,878363	0	0,1905	35,4149
Dbromelioides	0,2197404	0,7195121	0,829997	0,1632653	0,1905	35,4149
Dbromeliae sp IV	0,2243689	0,7680561	0,995904	0	0,1905	35,4149
Dincompta	0,2415977	0,6518382	0,853867	0	0,1905	35,4149

Table S8. Top models on the test of the influence of abiotic niche overlap, geographic overlap, biotic niche overlap, niche breadth and phylogenetic signal (predictor variable) on the potential for HTT among FBD species (as estimated with the Müller index, which was used as the response variable).

Model	d.f.	logLik	AICc	ΔAICc	weight
abiotic_niche_overlap (ANO)	5	1,61	11,06	0	0,41
ANO + biotic_niche_overlap	6	2,72	13,02	1,95	0,15
Null (Random effects only)	4	-1,66	13,98	2,91	0,09

Table S9. Model averaged results of the influence of abiotic niche overlap, geographic overlap, biotic niche overlap, niche breadth and phylogenetic signal (predictor variable) in predicting the potential for HTT among FBD species. Bold indicates statistically significant ($p < 0.05$) variables (as estimated with the Müller index, which was used as the response variable.

Predictor	β (Estimate)	Standard Error	Adjusted SE	P-value
abiotic niche overlap	0.113031	0.048905	0.051235	0.0274
biotic niche overlap	-0.074586	0.034325	0.036573	0.0414
geographic overlap	-0.008487	0.058853	0.064364	0.8951
niche breadth (log10)	0.266719	0.310047	0.339581	0.4322
phylogenetic signal	-0.014865	0.042420	0.045533	0.7441

File S1. VHICA output images. Each graph shows the distribution of p-values supporting the occurrence of HTT for different TEs between each pair of FBDs.

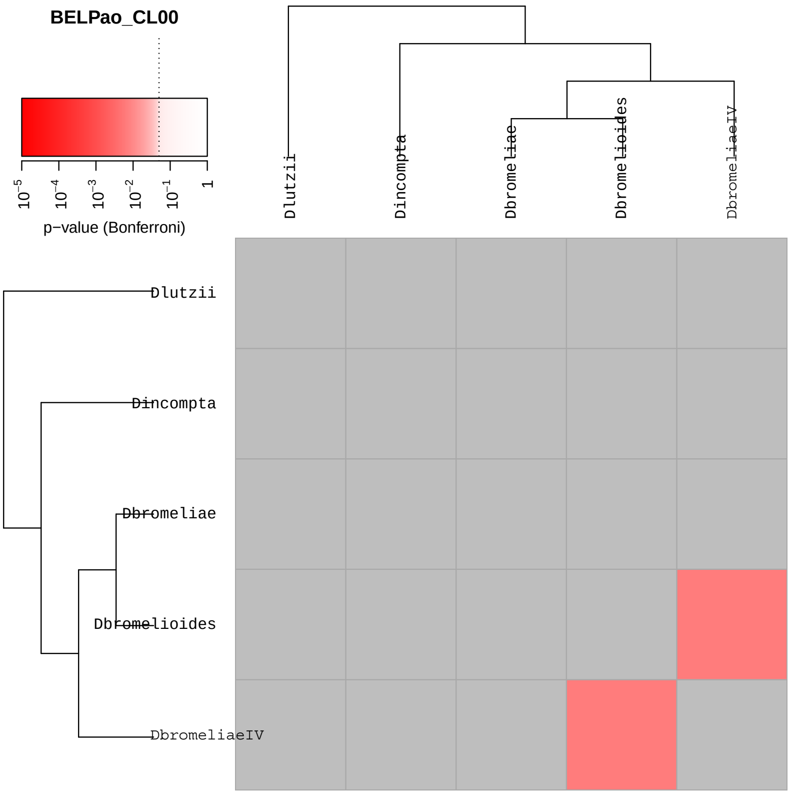

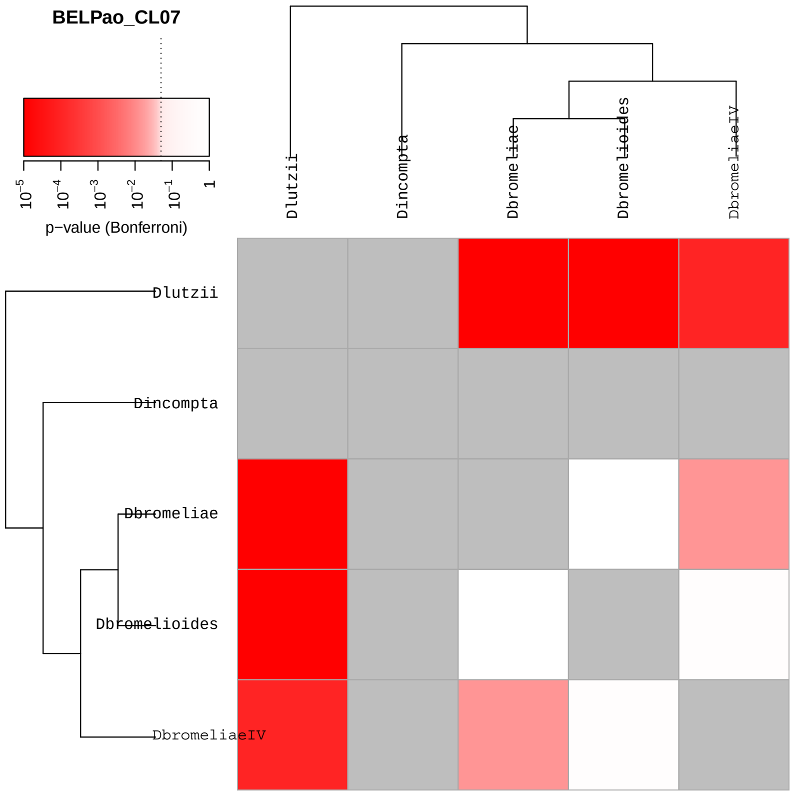

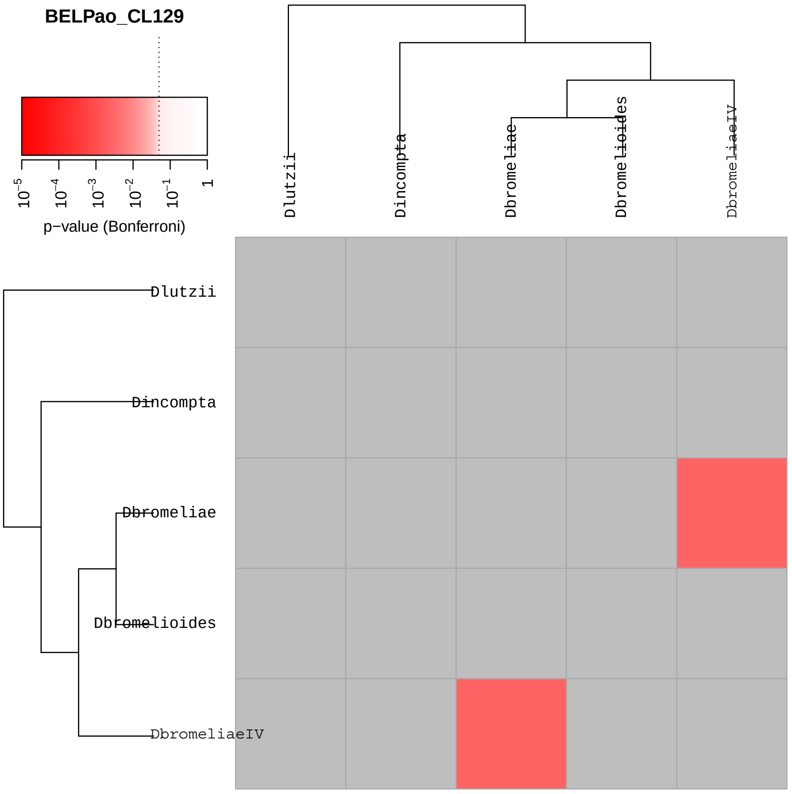

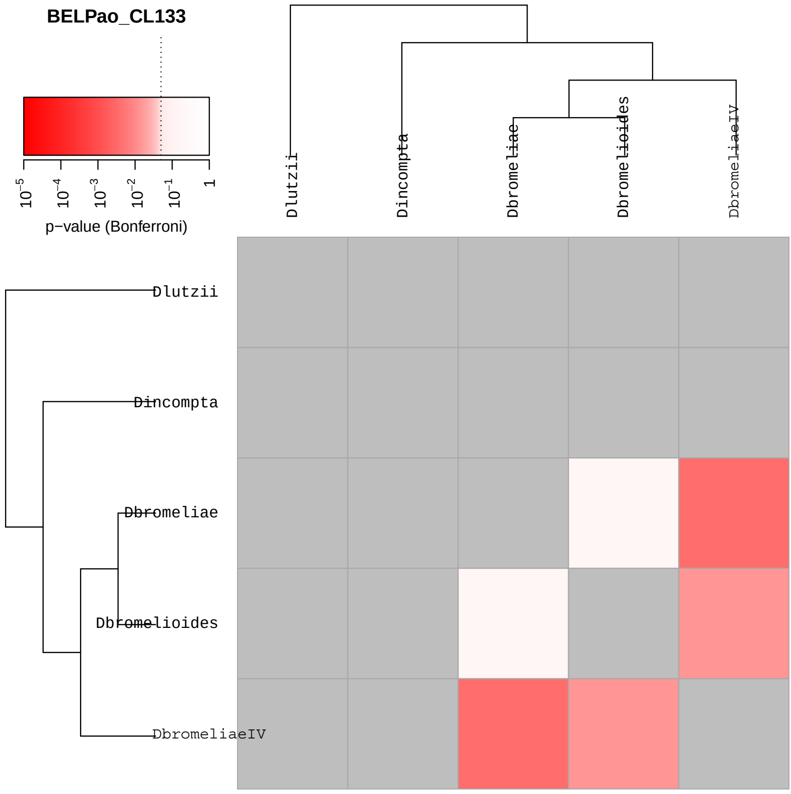

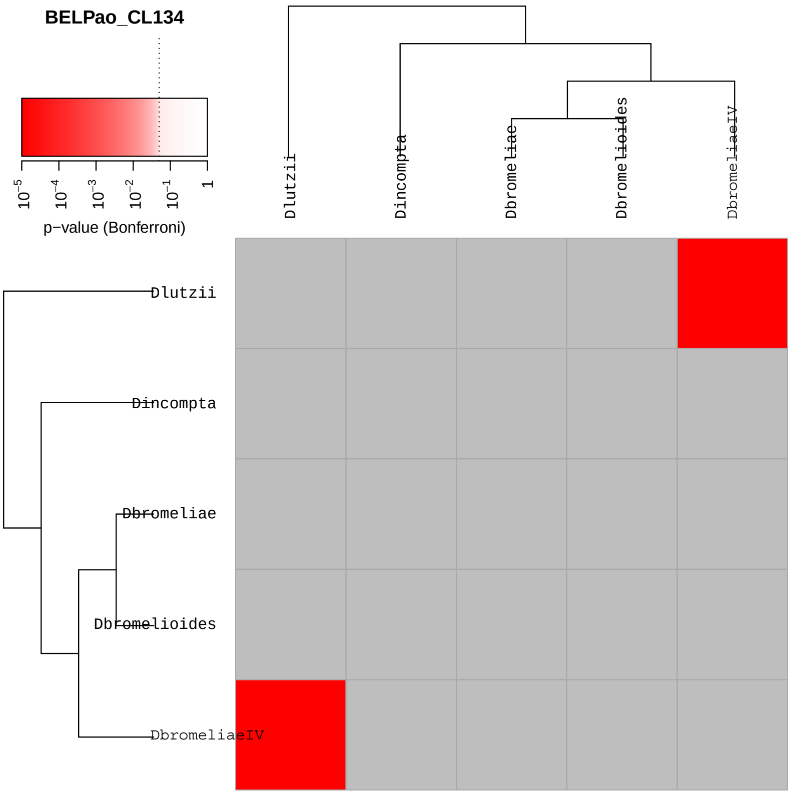
BELPao_CL134
10^-5 10^-4 10^-3 10^-2 10^-1 1
p−value (Bonferroni)
Dlutzii
Dincompta
Dbromeliae
Dbromelioides
DbromeliaeIV

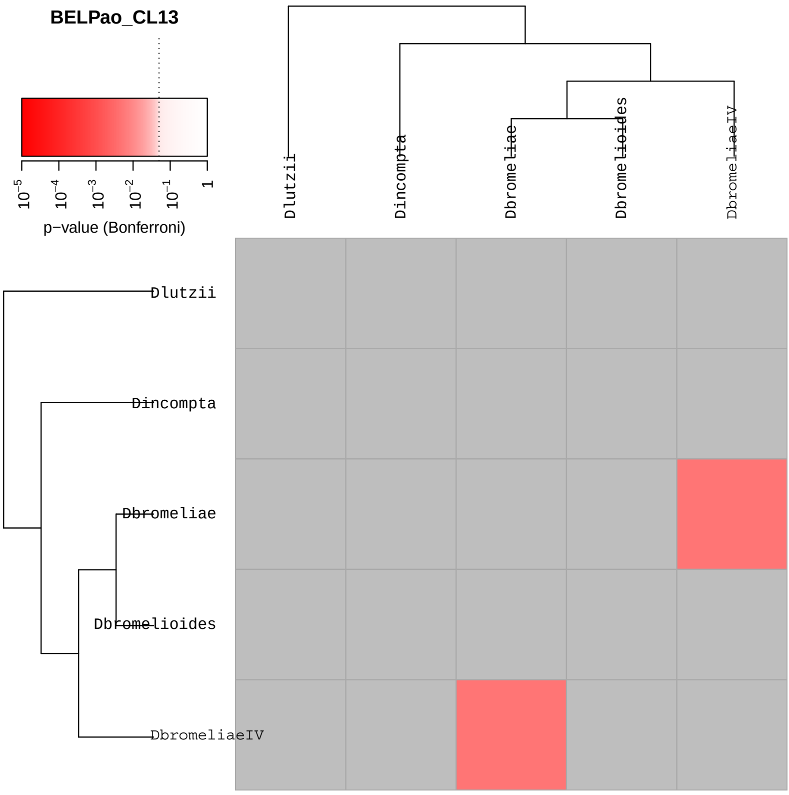
BELPao_CL13
10^-5 10^-4 10^-3 10^-2 10^-1 1
p−value (Bonferroni)
Dlutzii
Dincompta
Dbromeliae
Dbromelioides
DbromeliaeIV

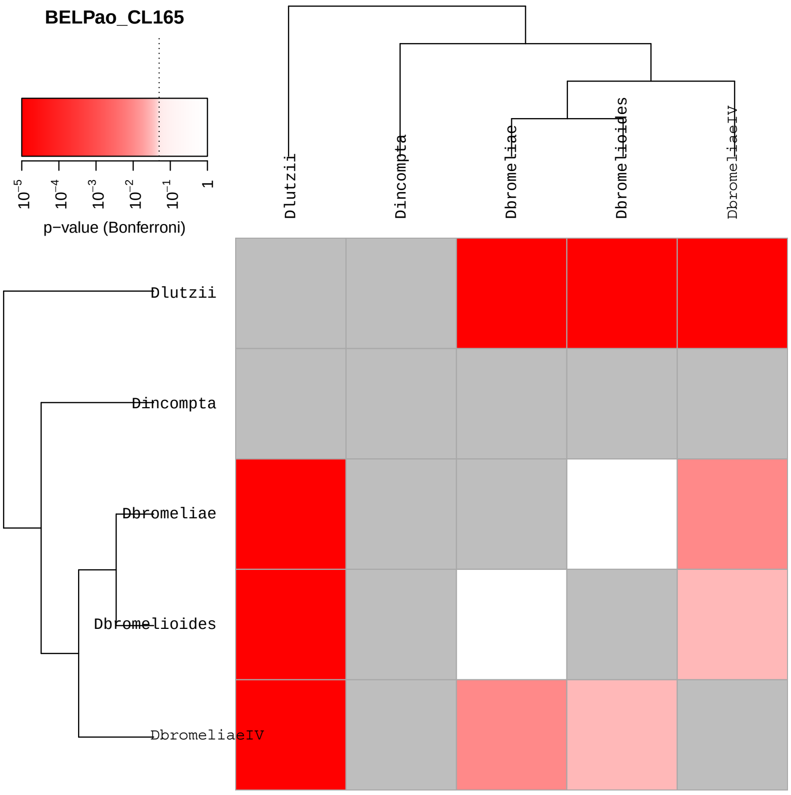
BELPao_CL165
10^-5 10^-4 10^-3 10^-2 10^-1 1
p−value (Bonferroni)
Dlutzii
Dincompta
Dbromeliae
Dbromelioides
DbromeliaeIV

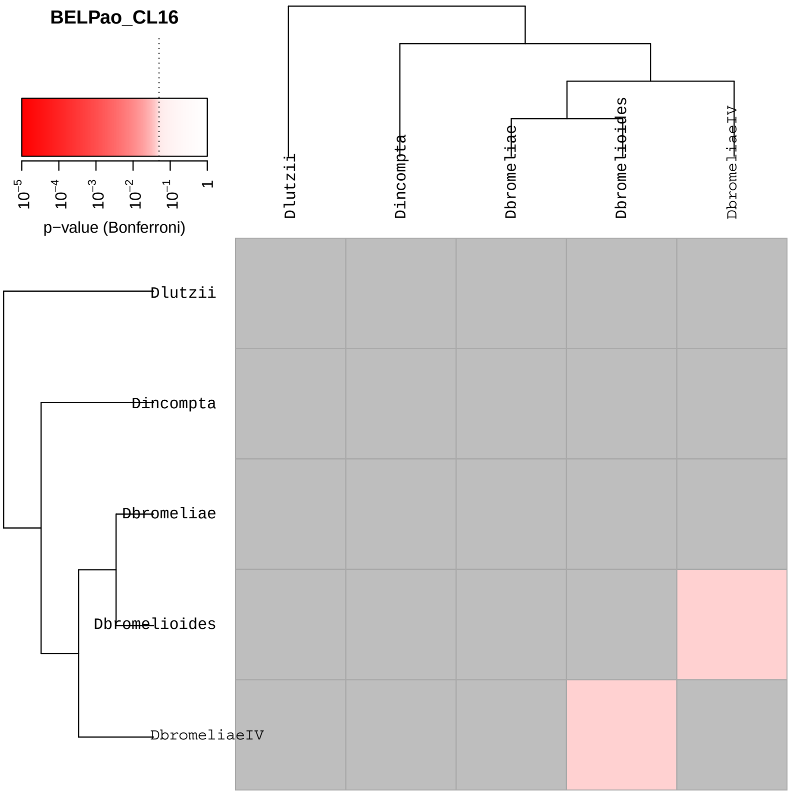
BELPao_CL16
10^-5 10^-4 10^-3 10^-2 10^-1 1
p−value (Bonferroni)
Dlutzii
Dincompta
Dbromeliae
Dbromelioides
DbromeliaeIV

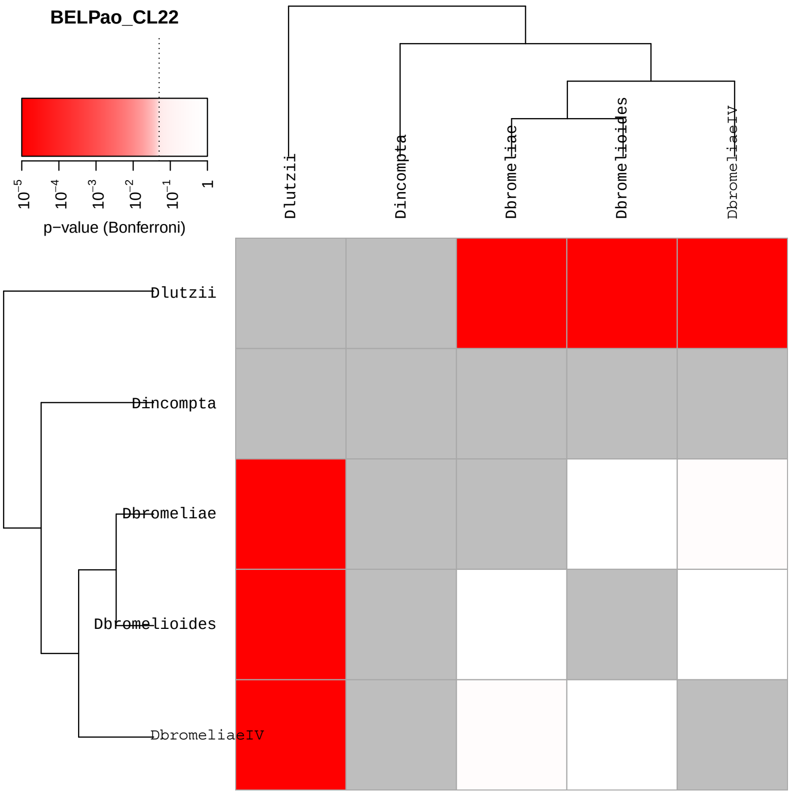
BELPao_CL22
p−value (Bonferroni)
Dlutzii
Dincompta
Dbromeliae
Dbromelioides
DbromeliaeIV

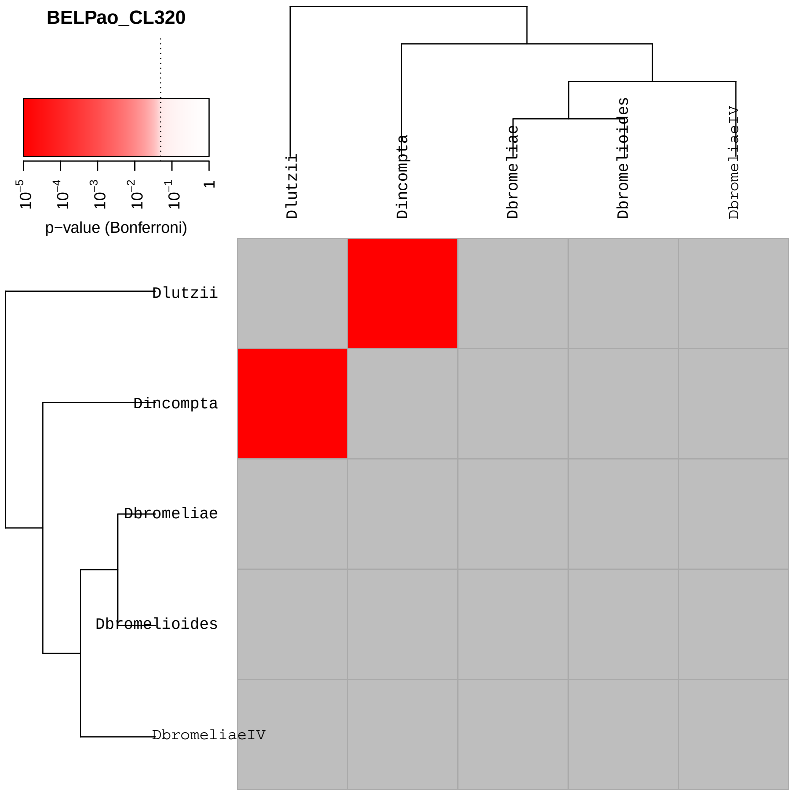
BELPao_CL320
p−value (Bonferroni)
Dlutzii
Dincompta
Dbromeliae
Dbromelioides
DbromeliaeIV

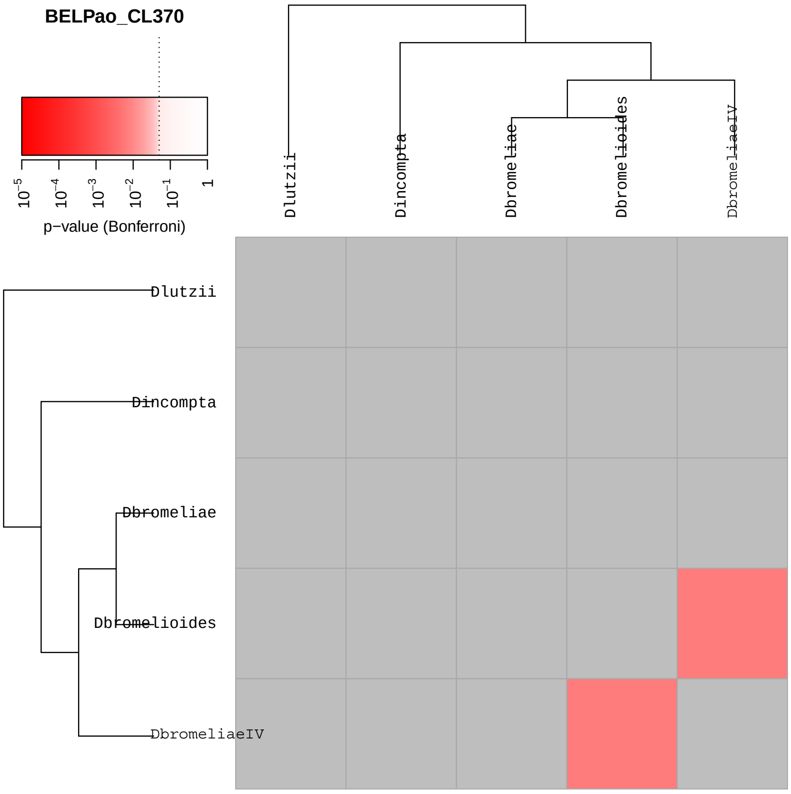
BELPao_CL370
p−value (Bonferroni)
Dlutzii
Dincompta
Dbromeliae
Dbromelioides
DbromeliaeIV

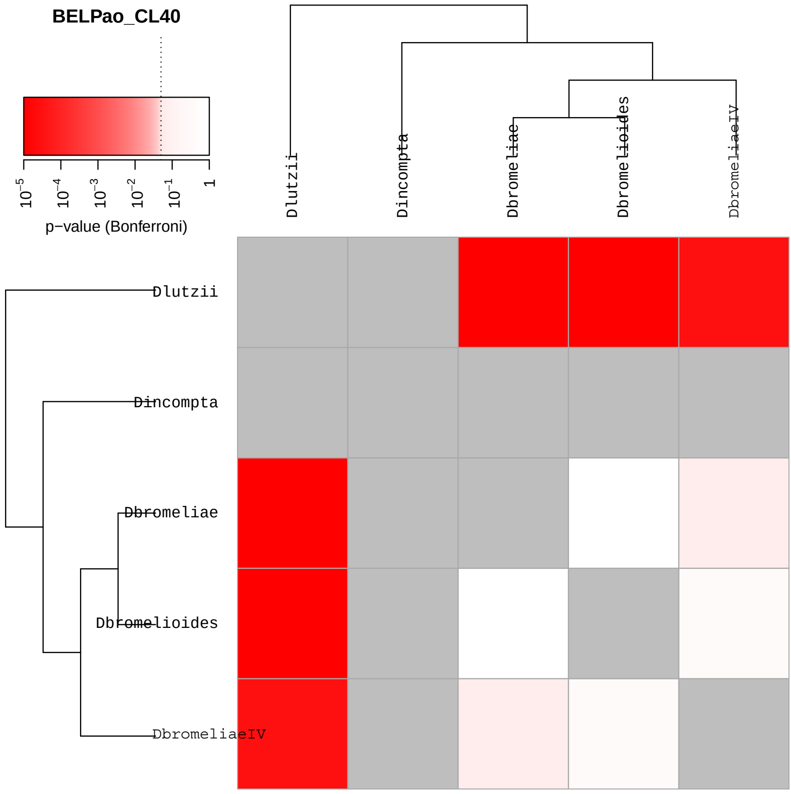
BELPao_CL40
p−value (Bonferroni)
Dlutzii
Dincompta
Dbromeliae
Dbromelioides
DbromeliaeIV

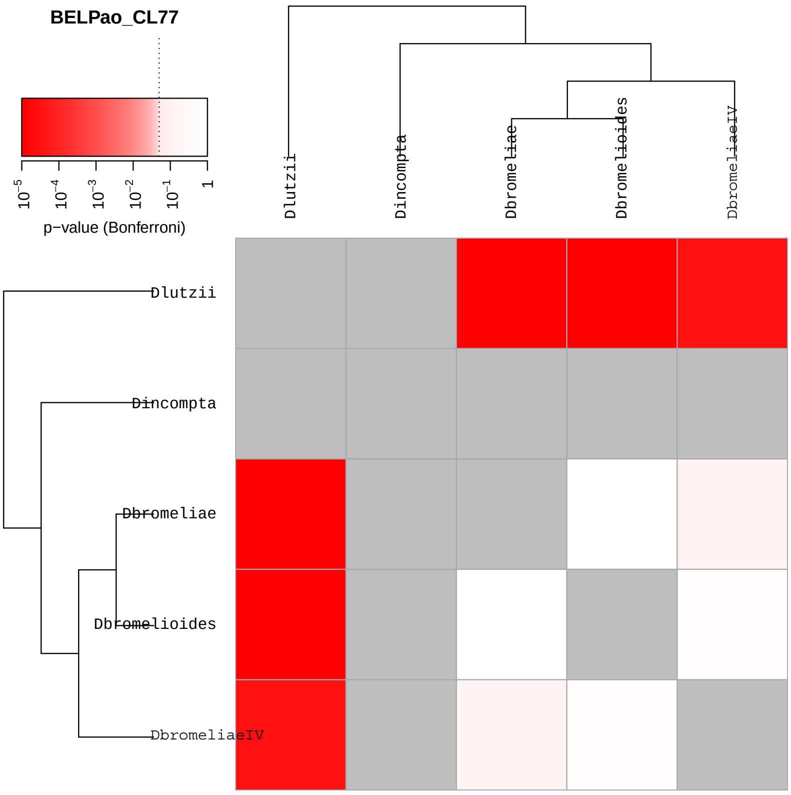
BELPao_CL77
10^{-5} 10^{-4} 10^{-3} 10^{-2} 10^{-1} 1
p−value (Bonferroni)
Dlutzii
Dincompta
Dbromeliae
Dbromelioides
DbromeliaeIV

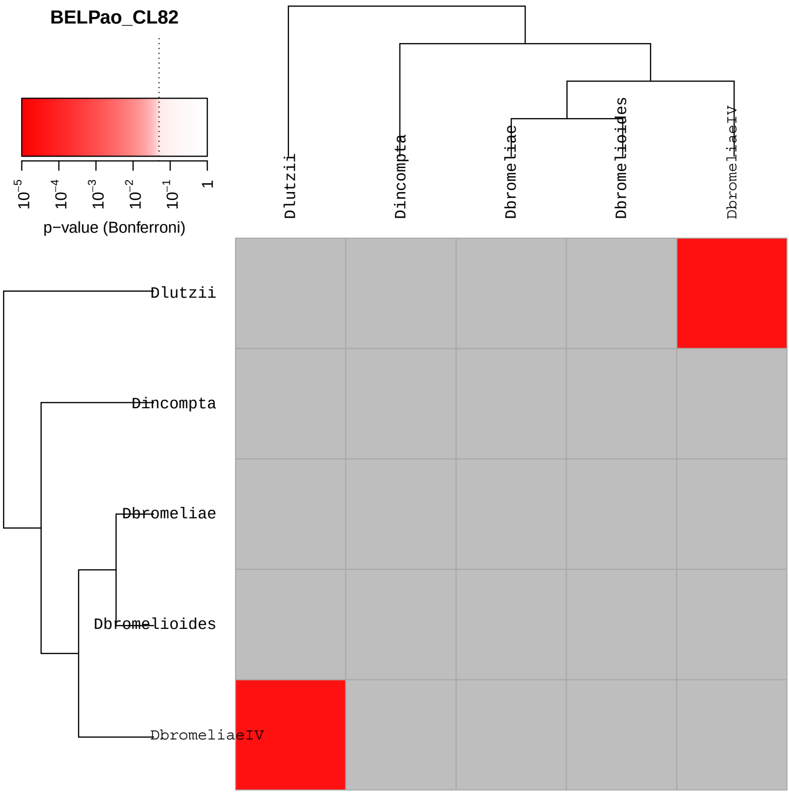
BELPao_CL82
10^{-5} 10^{-4} 10^{-3} 10^{-2} 10^{-1} 1
p−value (Bonferroni)
Dlutzii
Dincompta
Dbromeliae
Dbromelioides
DbromeliaeIV

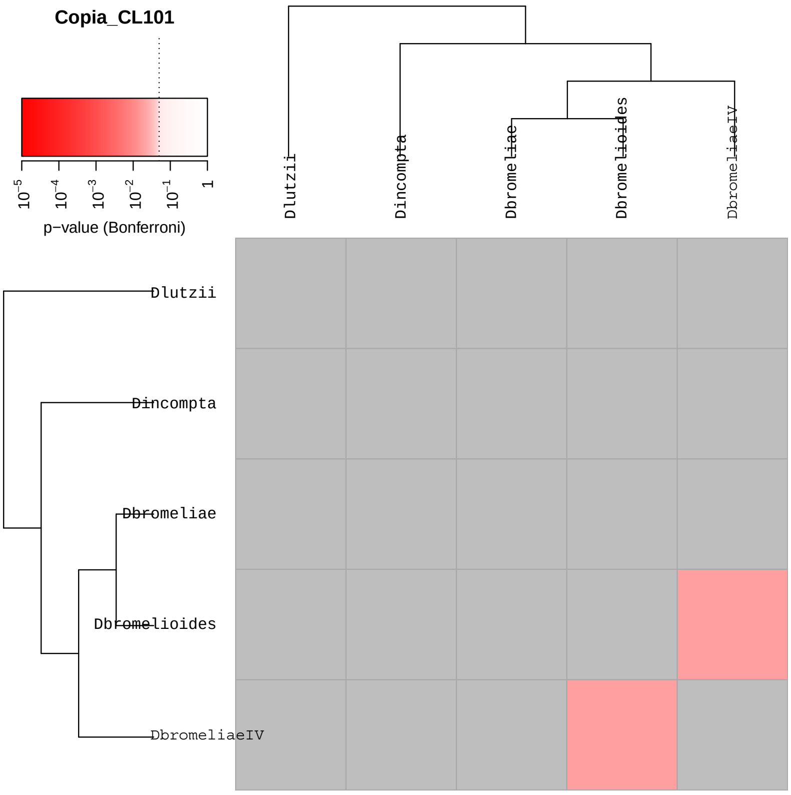
Copia_CL101
10^{-5} 10^{-4} 10^{-3} 10^{-2} 10^{-1} 1
p−value (Bonferroni)
Dlutzii
Dincompta
Dbromeliae
Dbromelioides
DbromeliaeIV

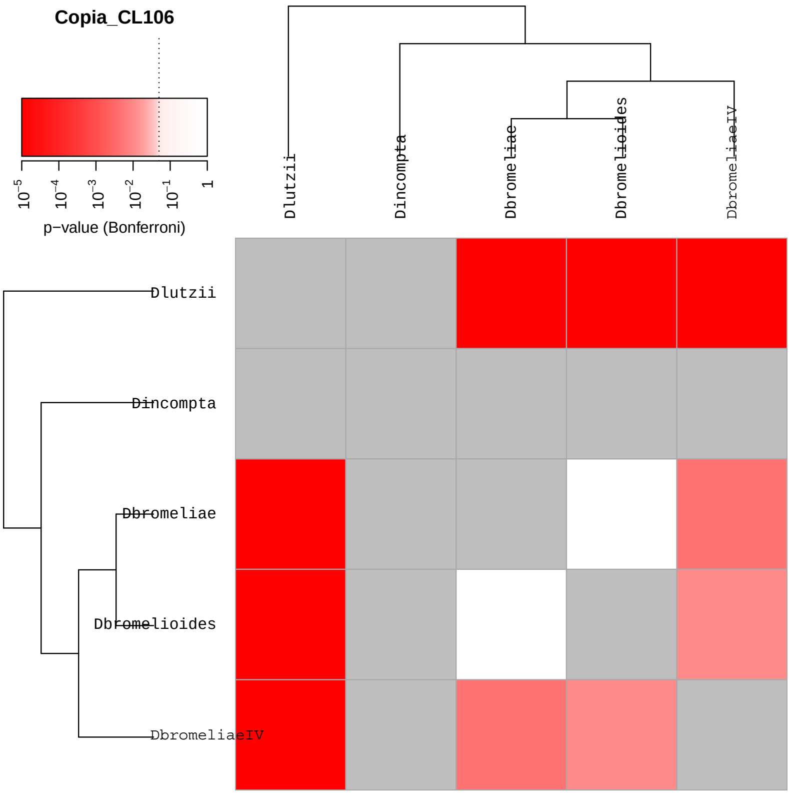
Copia_CL106
10^{-5} 10^{-4} 10^{-3} 10^{-2} 10^{-1} 1
p−value (Bonferroni)
Dlutzii
Dincompta
Dbromeliae
Dbromelioides
DbromeliaeIV

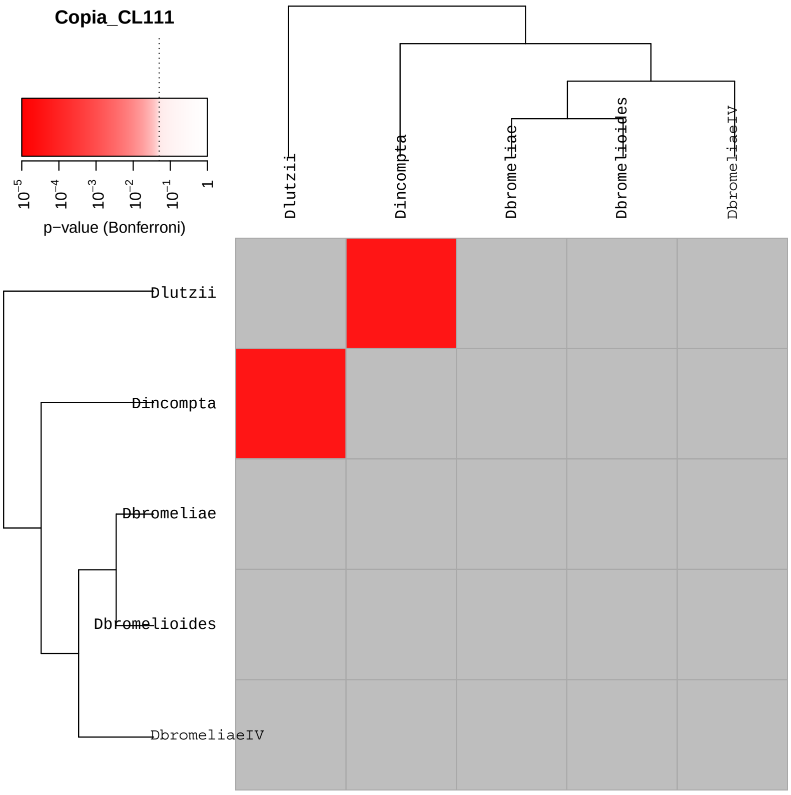
Copia_CL111
p−value (Bonferroni)
Dlutzii
Dincompta
Dbromeliae
Dbromelioides
DbromeliaeIV

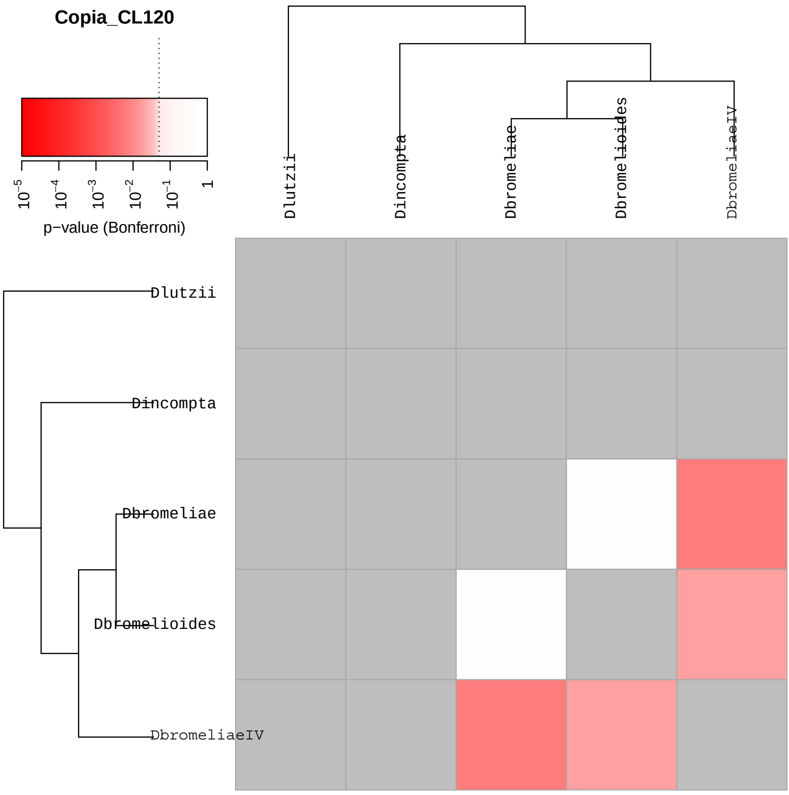
Copia_CL120
p−value (Bonferroni)
Dlutzii
Dincompta
Dbromeliae
Dbromelioides
DbromeliaeIV

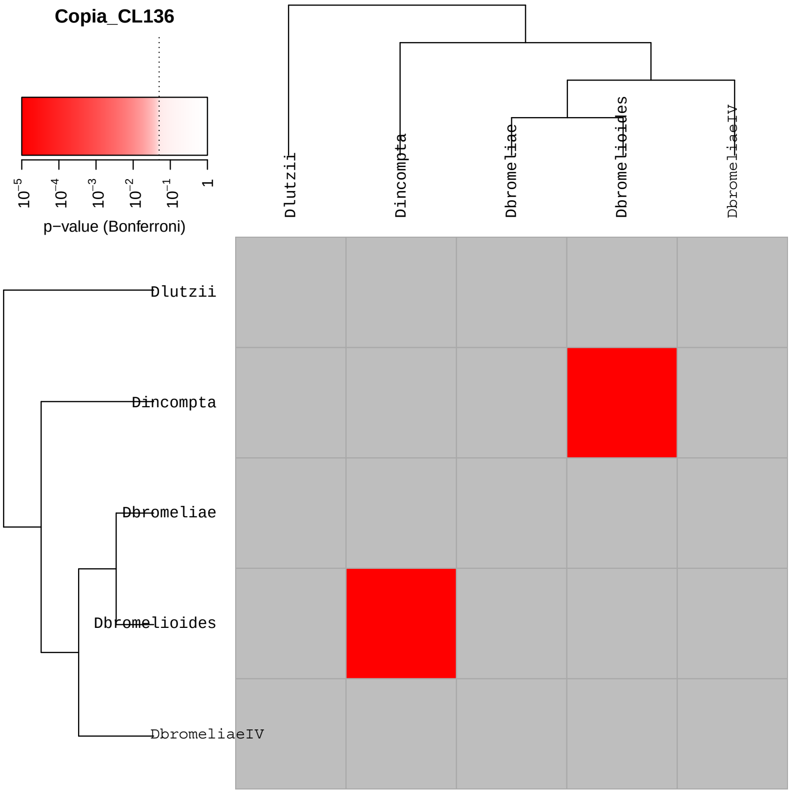
Copia_CL136
p−value (Bonferroni)
Dlutzii
Dincompta
Dbromeliae
Dbromelioides
DbromeliaeIV

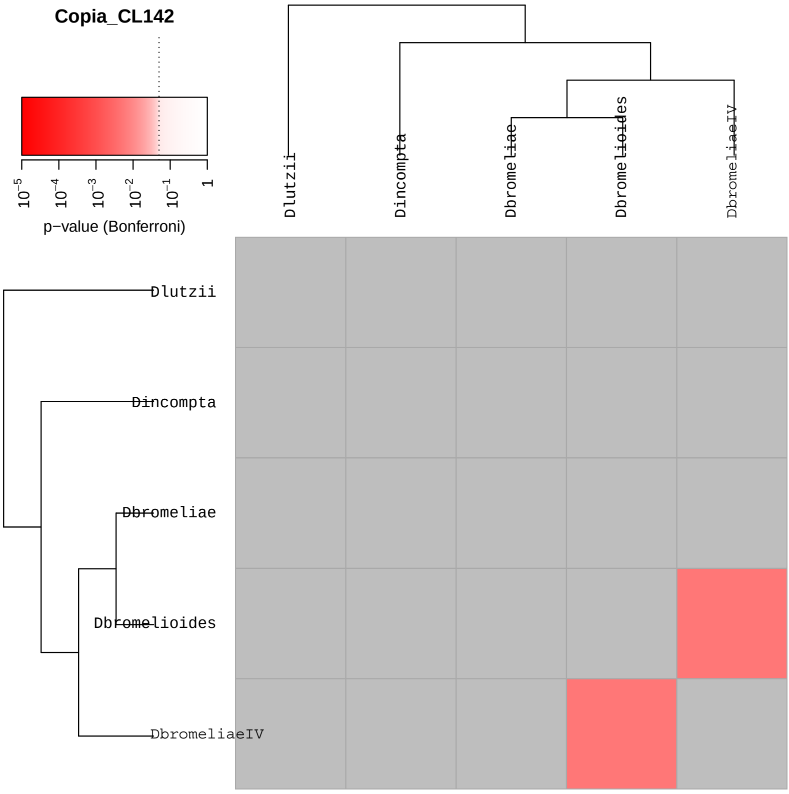
Copia_CL142
p−value (Bonferroni)
Dlutzii
Dincompta
Dbromeliae
Dbromelioides
DbromeliaeIV

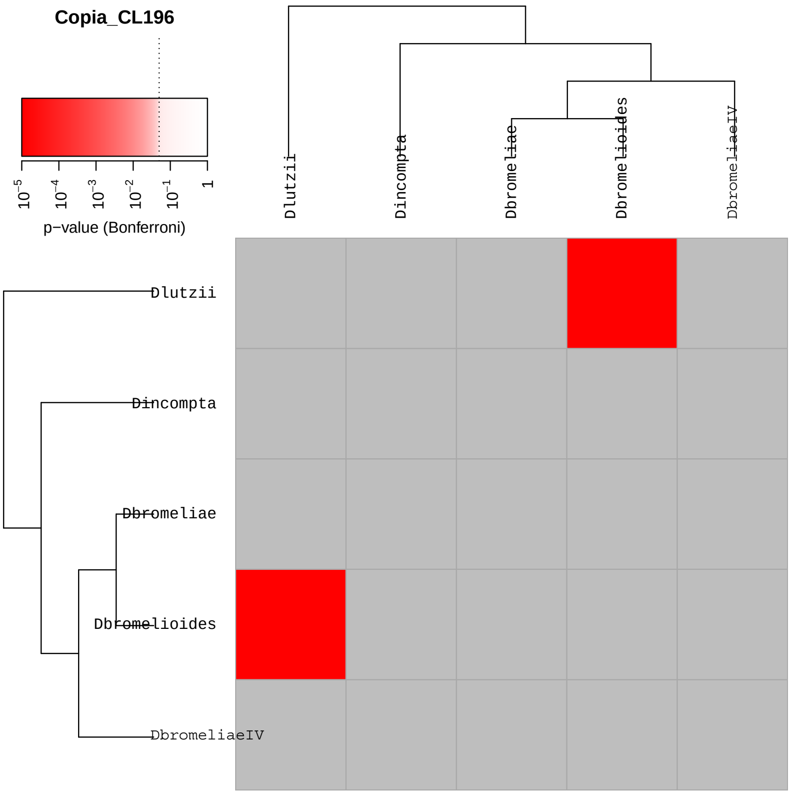
Copia_CL196
10−5 10−4 10−3 10−2 10−1 1
p−value (Bonferroni)
Dlutzii
Dincompta
Dbromeliae
Dbromelioides
DbromeliaeIV

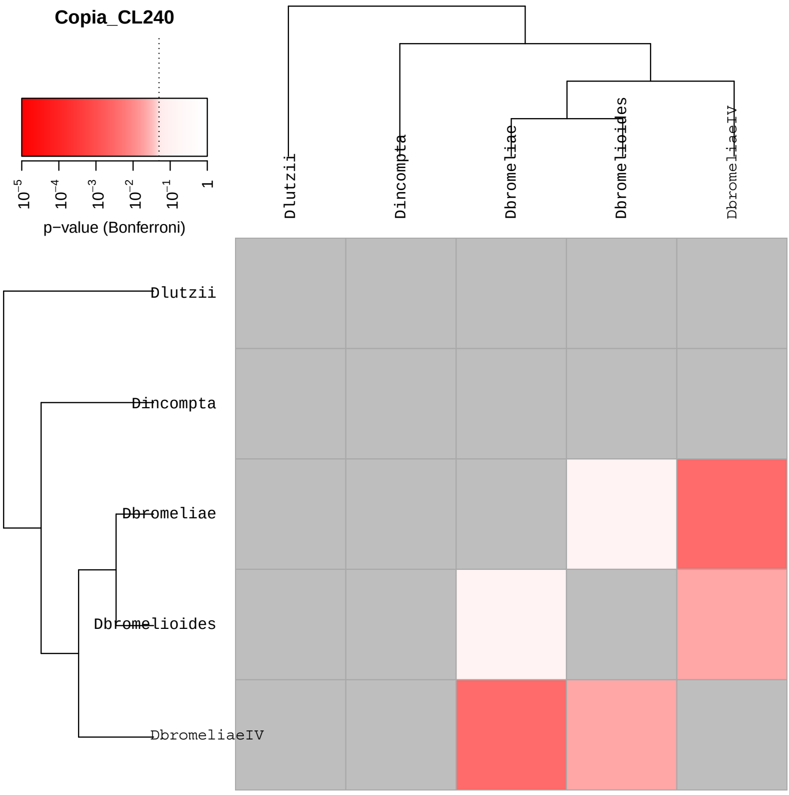
Copia_CL240
10−5 10−4 10−3 10−2 10−1 1
p−value (Bonferroni)
Dlutzii
Dincompta
Dbromeliae
Dbromelioides
DbromeliaeIV

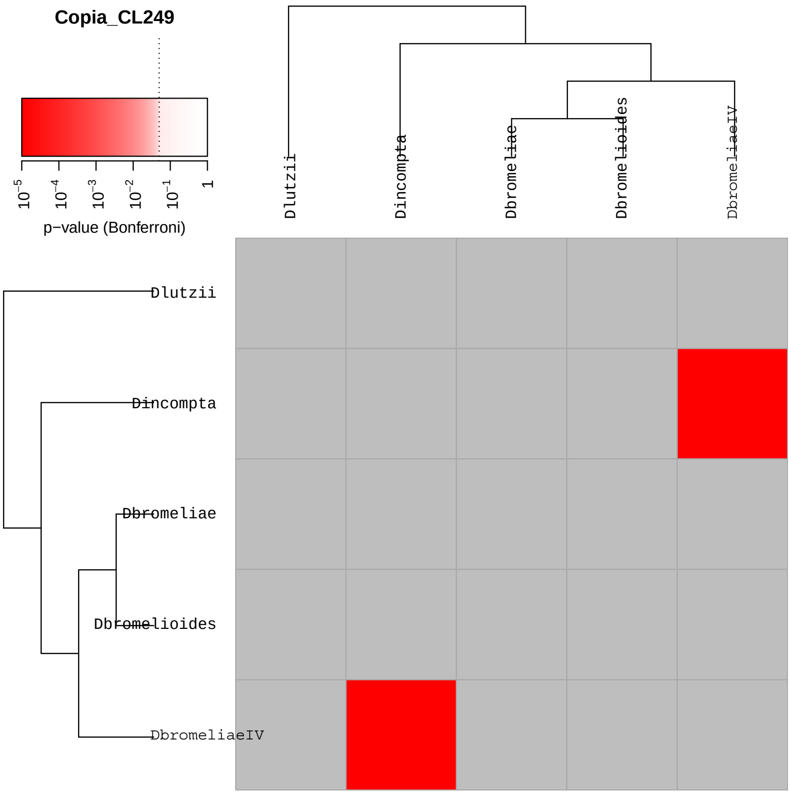
Copia_CL249
10−5 10−4 10−3 10−2 10−1 1
p−value (Bonferroni)
Dlutzii
Dincompta
Dbromeliae
Dbromelioides
DbromeliaeIV

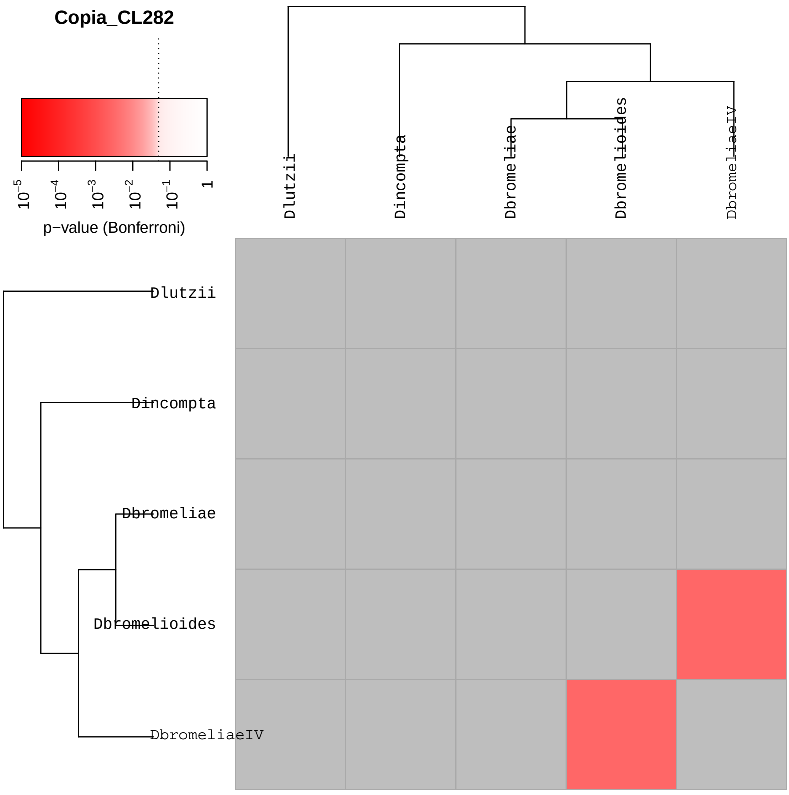
Copia_CL282
10−5 10−4 10−3 10−2 10−1 1
p−value (Bonferroni)
Dlutzii
Dincompta
Dbromeliae
Dbromelioides
DbromeliaeIV

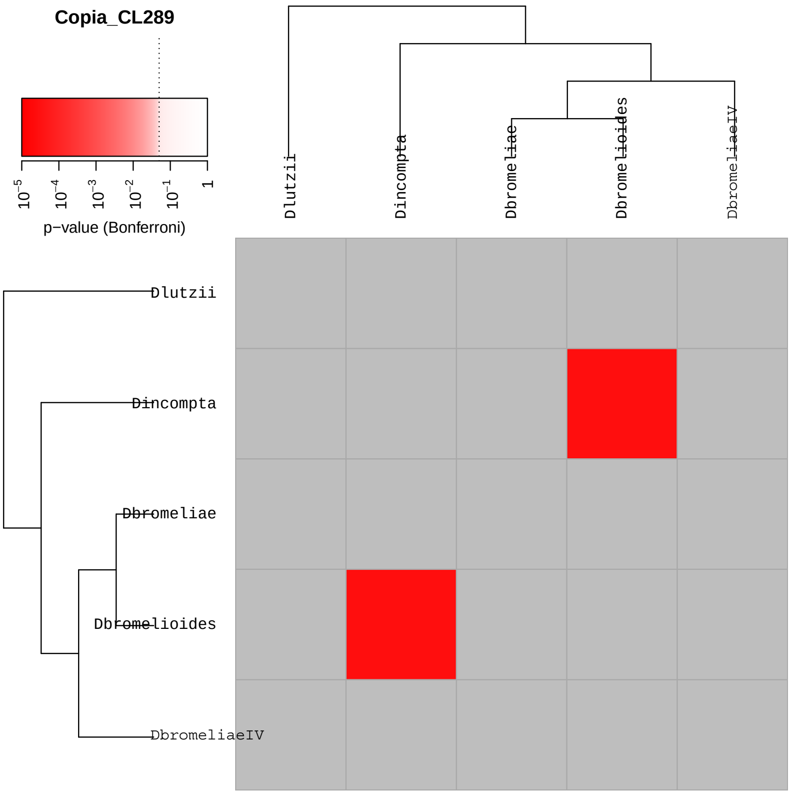
Copia_CL289
p−value (Bonferroni)
10−5 10−4 10−3 10−2 10−1 1
Dlutzii
Dincompta
Dbromeliae
Dbromelioides
DbromeliaeIV
Dlutzii
Dincompta
Dbromeliae
Dbromelioides
DbromeliaeIV

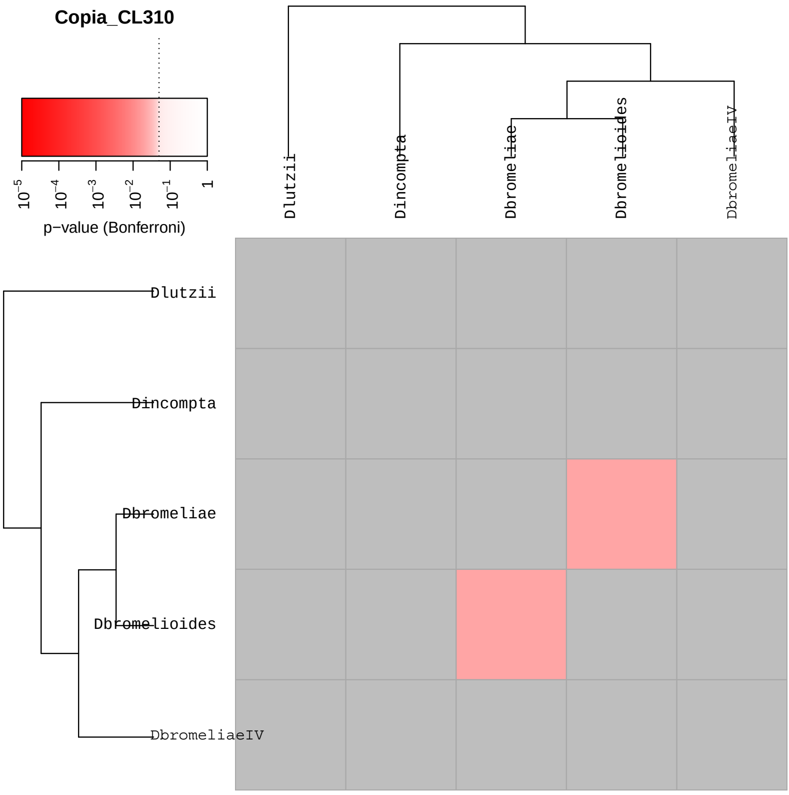
Copia_CL310
p−value (Bonferroni)
10−5 10−4 10−3 10−2 10−1 1
Dlutzii
Dincompta
Dbromeliae
Dbromelioides
DbromeliaeIV
Dlutzii
Dincompta
Dbromeliae
Dbromelioides
DbromeliaeIV

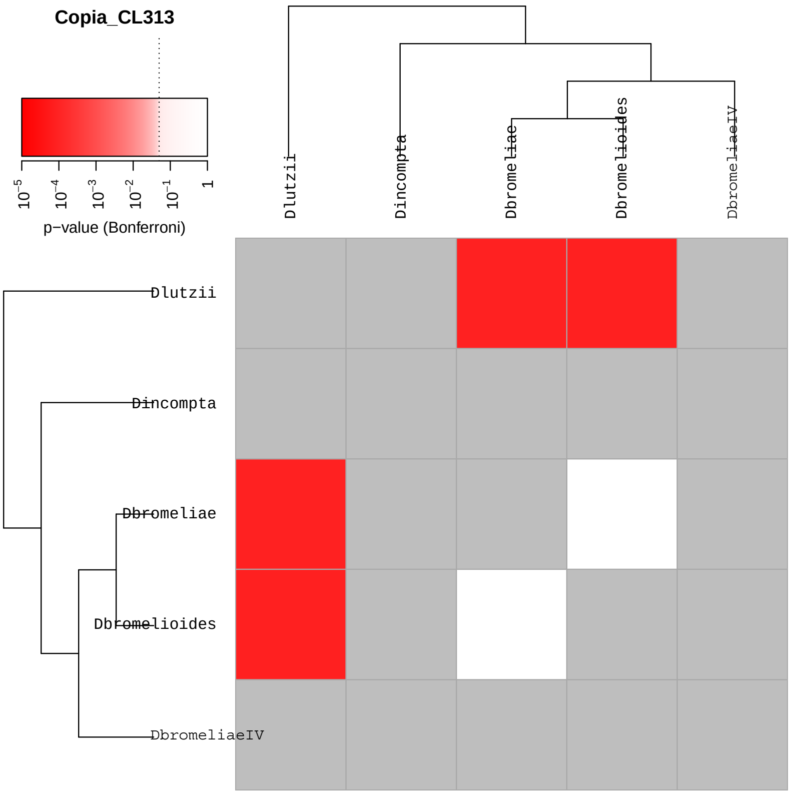
Copia_CL313
p−value (Bonferroni)
10−5 10−4 10−3 10−2 10−1 1
Dlutzii
Dincompta
Dbromeliae
Dbromelioides
DbromeliaeIV
Dlutzii
Dincompta
Dbromeliae
Dbromelioides
DbromeliaeIV

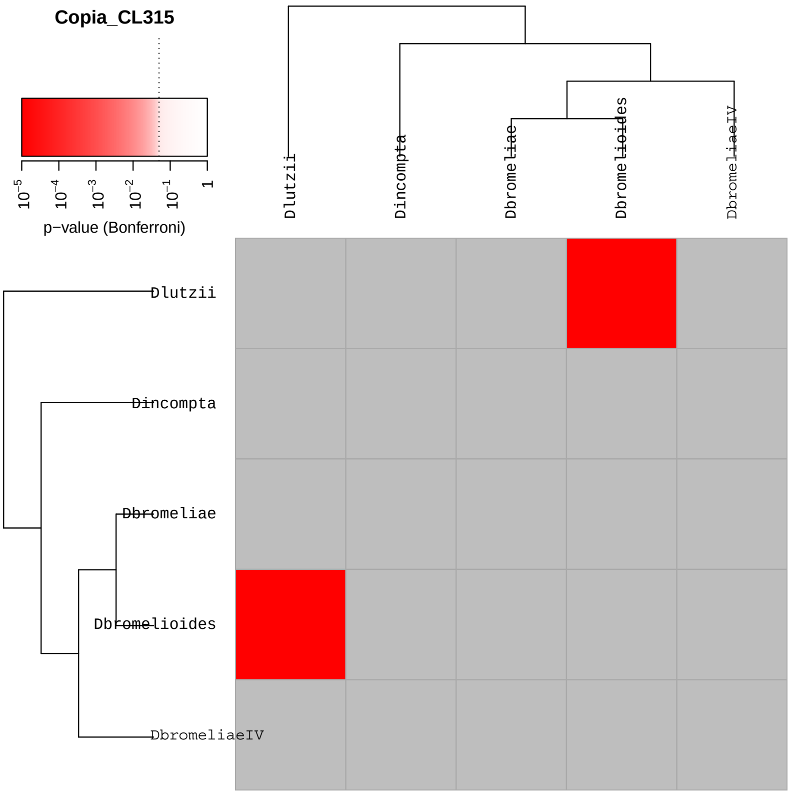
Copia_CL315
p−value (Bonferroni)
10−5 10−4 10−3 10−2 10−1 1
Dlutzii
Dincompta
Dbromeliae
Dbromelioides
DbromeliaeIV
Dlutzii
Dincompta
Dbromeliae
Dbromelioides
DbromeliaeIV

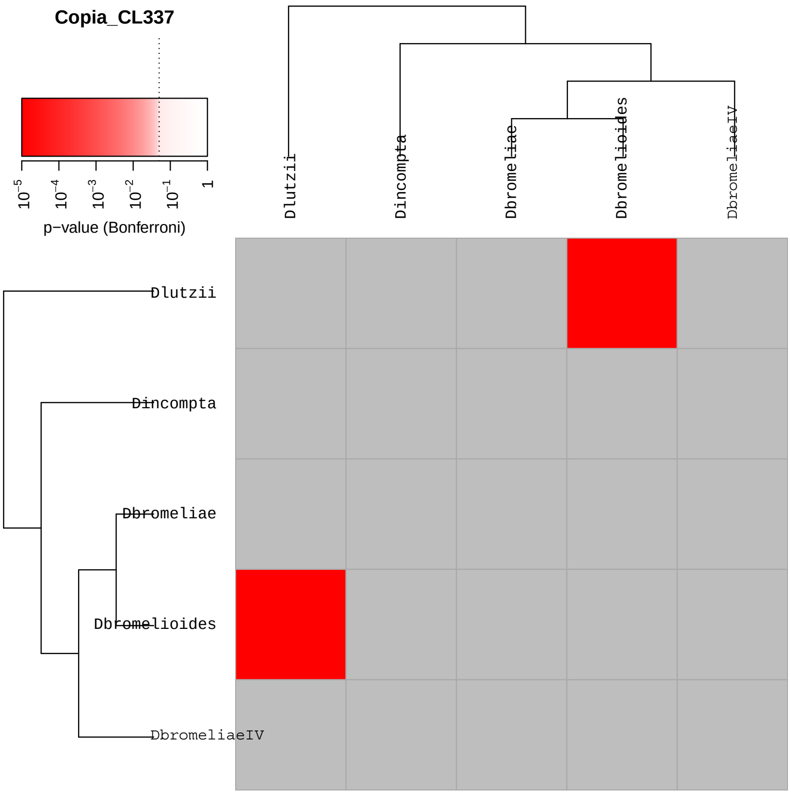
Copia_CL337
10^{-5} 10^{-4} 10^{-3} 10^{-2} 10^{-1} 1
p−value (Bonferroni)
Dlutzii
Dincompta
Dbromeliae
Dbromelioides
DbromeliaeIV

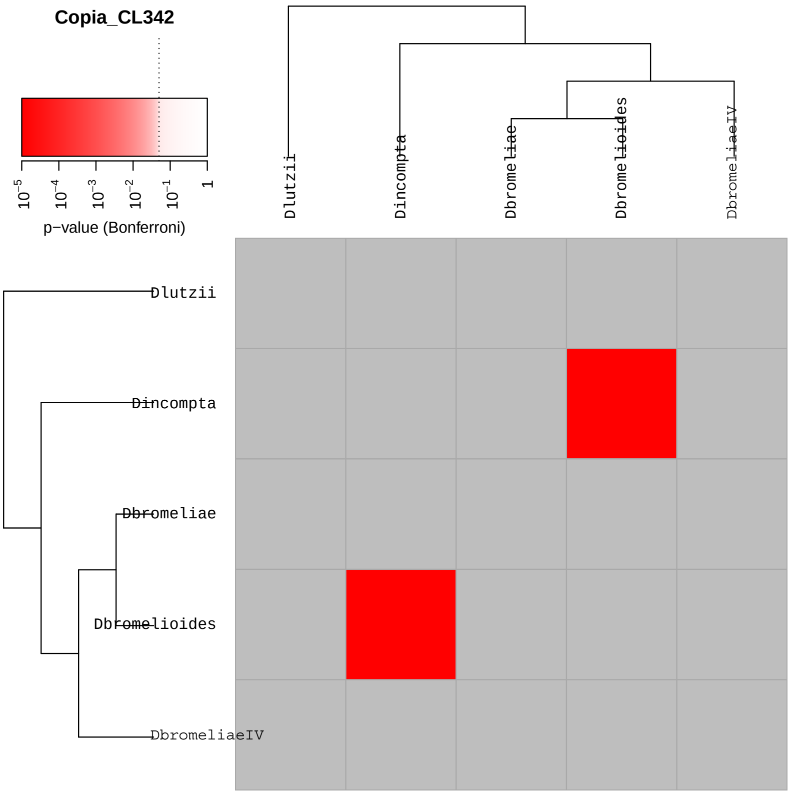
Copia_CL342
10^{-5} 10^{-4} 10^{-3} 10^{-2} 10^{-1} 1
p−value (Bonferroni)
Dlutzii
Dincompta
Dbromeliae
Dbromelioides
DbromeliaeIV

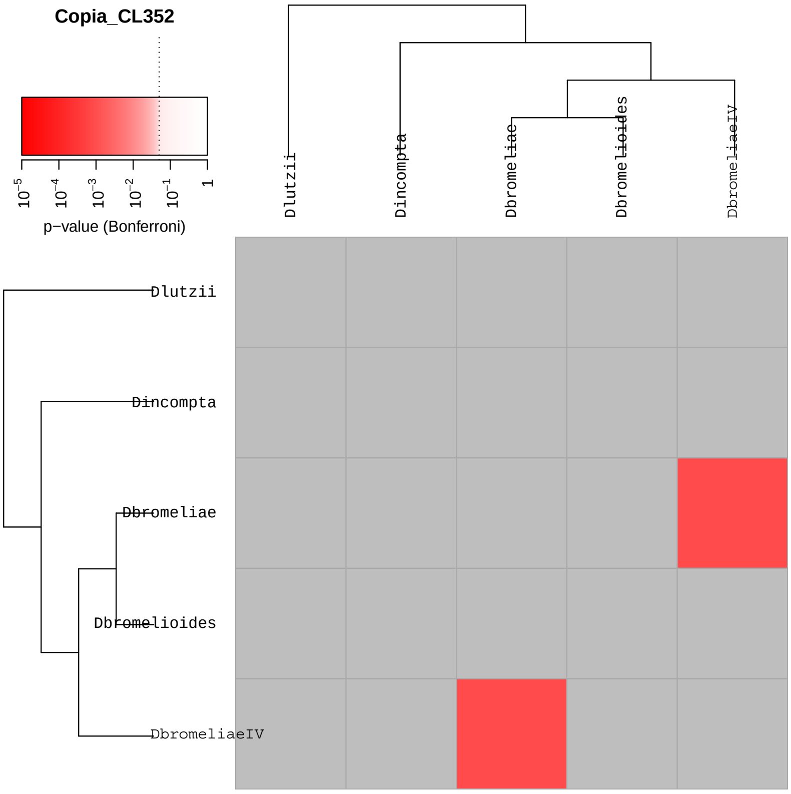
Copia_CL352
10^{-5} 10^{-4} 10^{-3} 10^{-2} 10^{-1} 1
p−value (Bonferroni)
Dlutzii
Dincompta
Dbromeliae
Dbromelioides
DbromeliaeIV

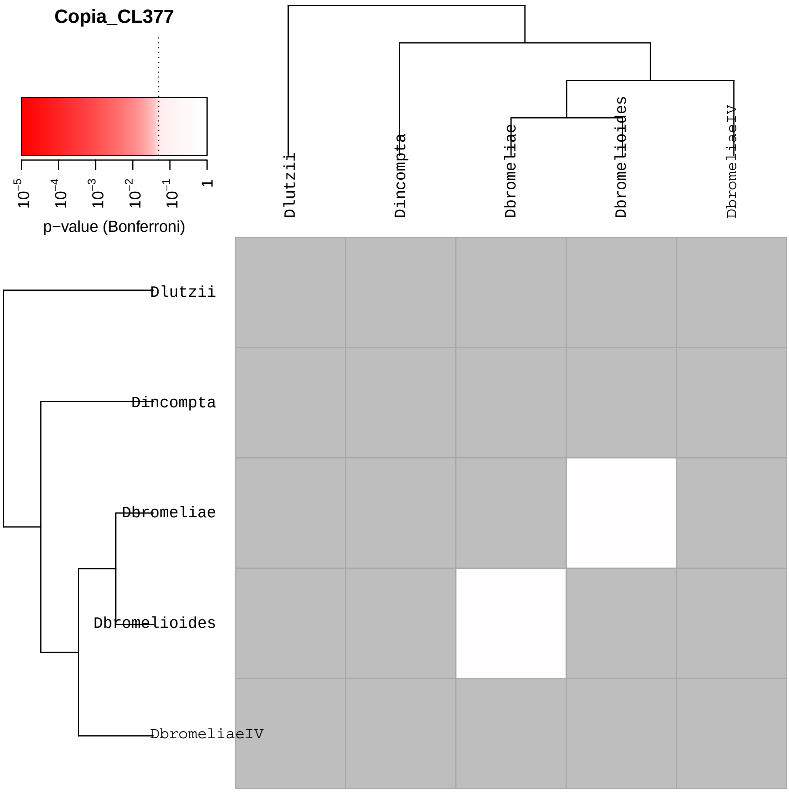
Copia_CL377
10^{-5} 10^{-4} 10^{-3} 10^{-2} 10^{-1} 1
p−value (Bonferroni)
Dlutzii
Dincompta
Dbromeliae
Dbromelioides
DbromeliaeIV

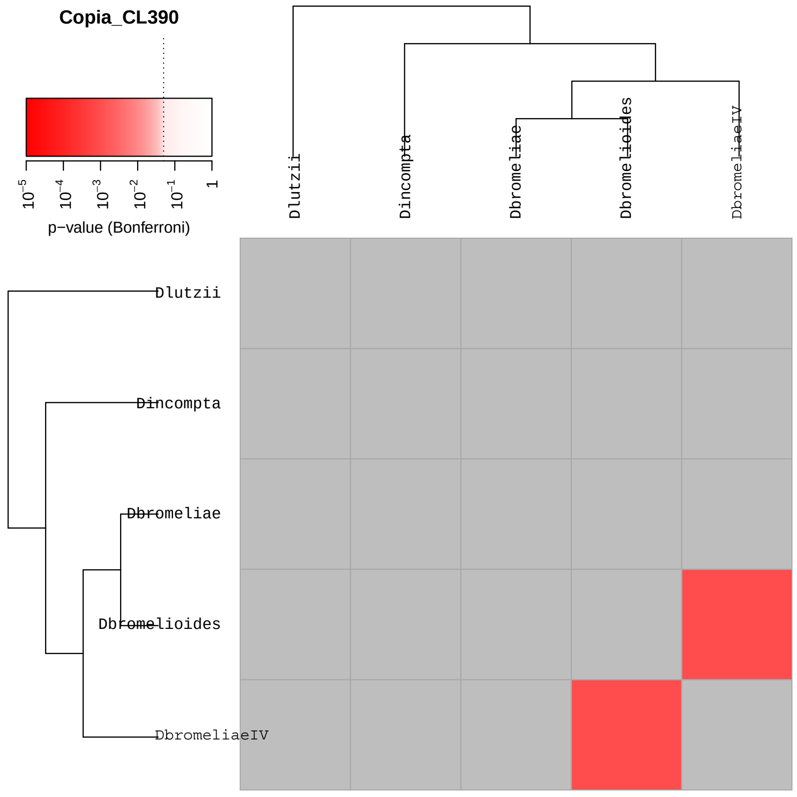
Copia_CL390
10^{-5} 10^{-4} 10^{-3} 10^{-2} 10^{-1} 1
p−value (Bonferroni)
Dlutzii
Dincompta
Dbromeliae
Dbromelioides
DbromeliaeIV

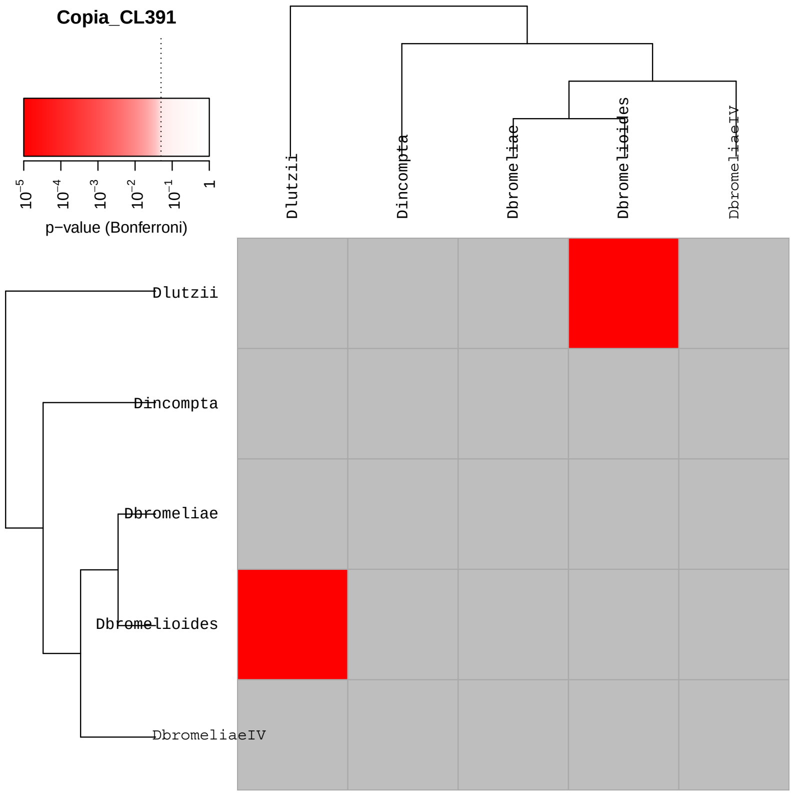
Copia_CL391
10^{-5} 10^{-4} 10^{-3} 10^{-2} 10^{-1} 1
p−value (Bonferroni)
Dlutzii
Dincompta
Dbromeliae
Dbromelioides
DbromeliaeIV

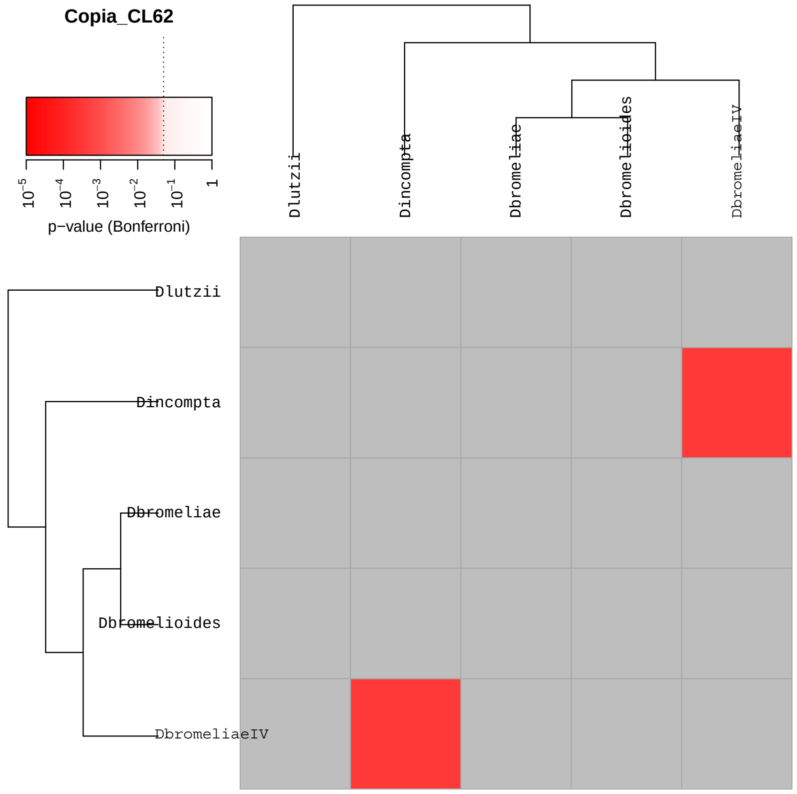
Copia_CL62
10^{-5} 10^{-4} 10^{-3} 10^{-2} 10^{-1} 1
p−value (Bonferroni)
Dlutzii
Dincompta
Dbromeliae
Dbromelioides
DbromeliaeIV

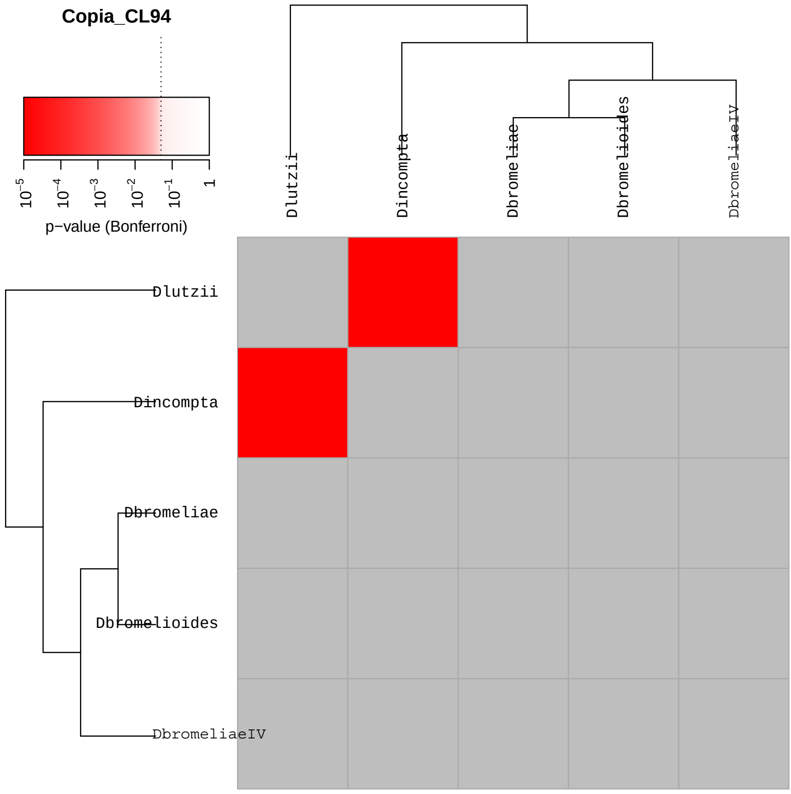
Copia_CL94
10^{-5} 10^{-4} 10^{-3} 10^{-2} 10^{-1} 1
p−value (Bonferroni)
Dlutzii
Dincompta
Dbromeliae
Dbromelioides
DbromeliaeIV

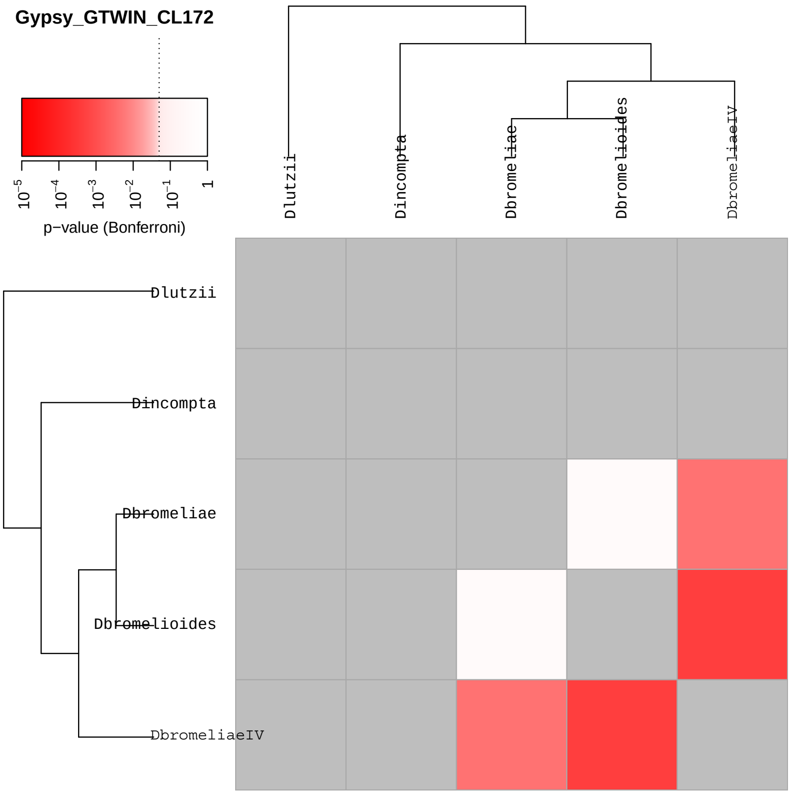
Gypsy_GTWIN_CL172
10^{-5} 10^{-4} 10^{-3} 10^{-2} 10^{-1} 1
p−value (Bonferroni)
Dlutzii
Dincompta
Dbromeliae
Dbromelioides
DbromeliaeIV

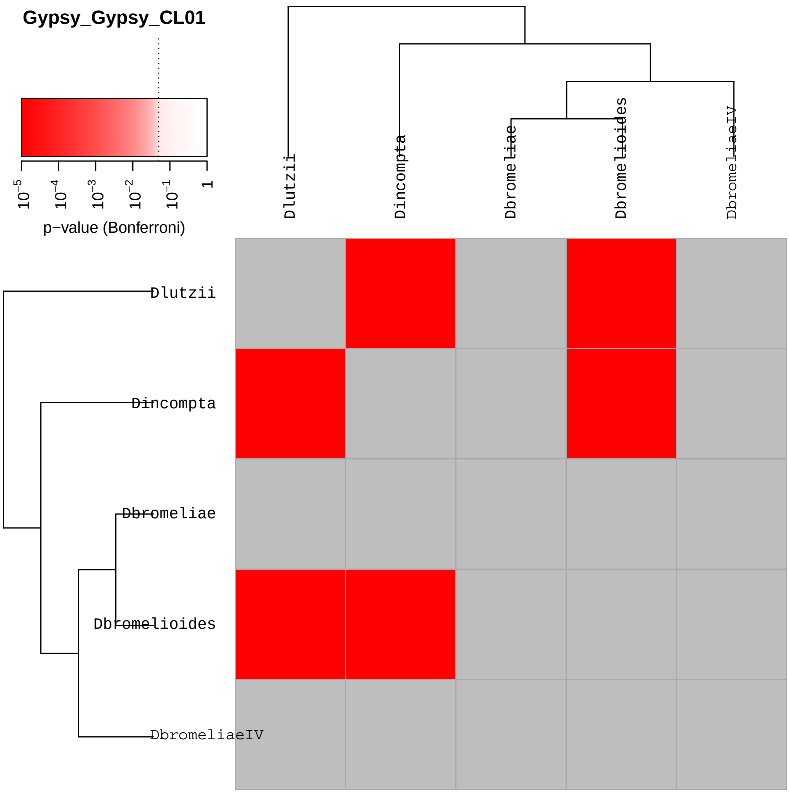
Gypsy_Gypsy_CL01
10^{-5} 10^{-4} 10^{-3} 10^{-2} 10^{-1} 1
p−value (Bonferroni)
Dlutzii
Dincompta
Dbromeliae
Dbromelioides
DbromeliaeIV

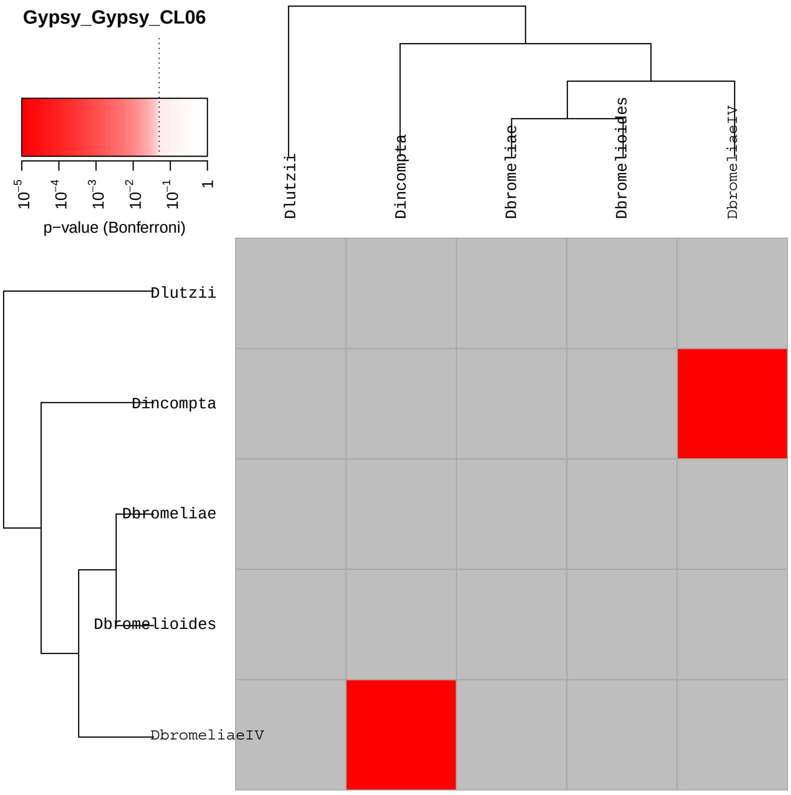
Gypsy_Gypsy_CL06
10^{-5} 10^{-4} 10^{-3} 10^{-2} 10^{-1} 1
p−value (Bonferroni)
Dlutzii
Dincompta
Dbromeliae
Dbromelioides
DbromeliaeIV

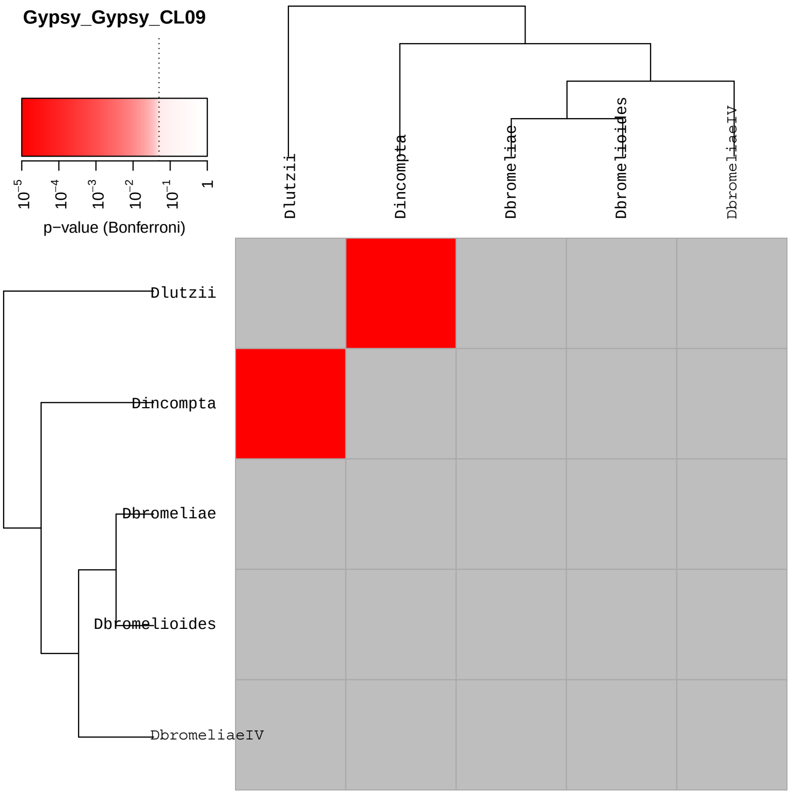
Gypsy_Gypsy_CL09
10^{-5} 10^{-4} 10^{-3} 10^{-2} 10^{-1} 1
p−value (Bonferroni)
Dlutzii
Dincompta
Dbromeliae
Dbromelioides
DbromeliaeIV

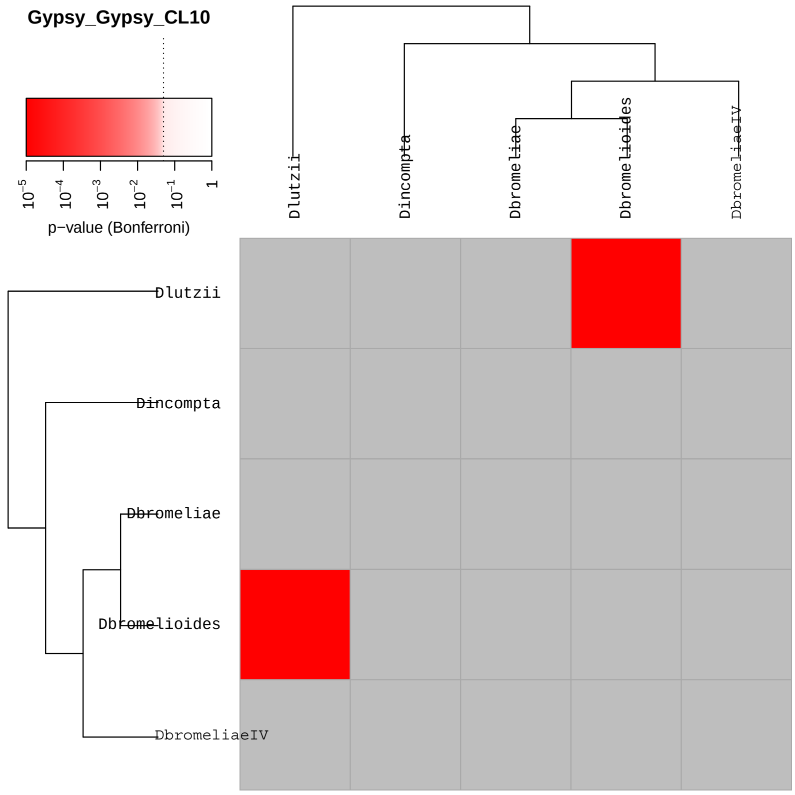
Gypsy_Gypsy_CL10
10^-5 10^-4 10^-3 10^-2 10^-1 1
p−value (Bonferroni)
Dlutzii
Dincompta
Dbromeliae
Dbromelioides
DbromeliaeIV

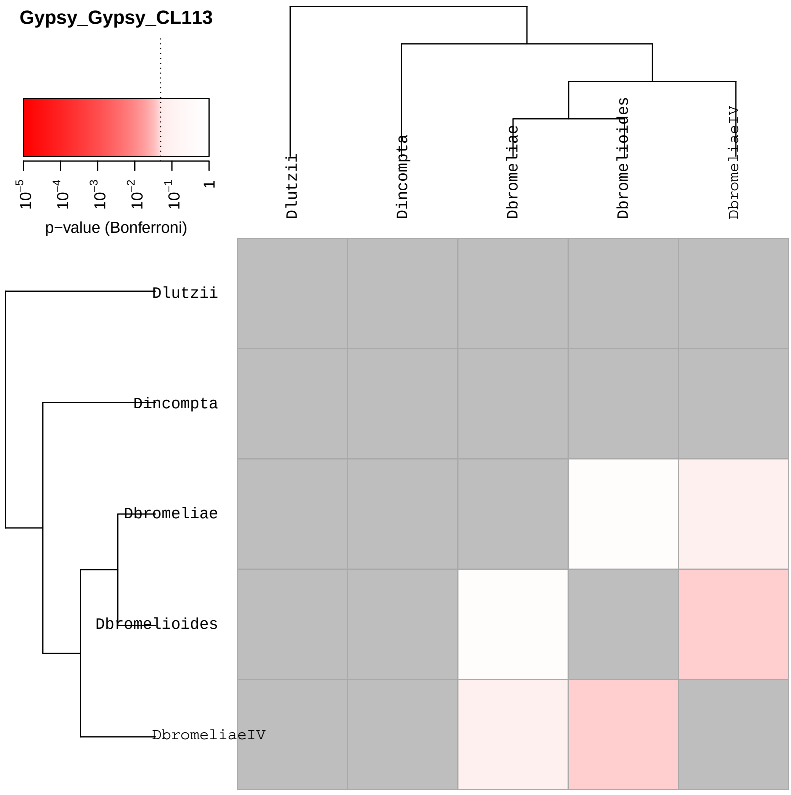
Gypsy_Gypsy_CL113
10^-5 10^-4 10^-3 10^-2 10^-1 1
p−value (Bonferroni)
Dlutzii
Dincompta
Dbromeliae
Dbromelioides
DbromeliaeIV

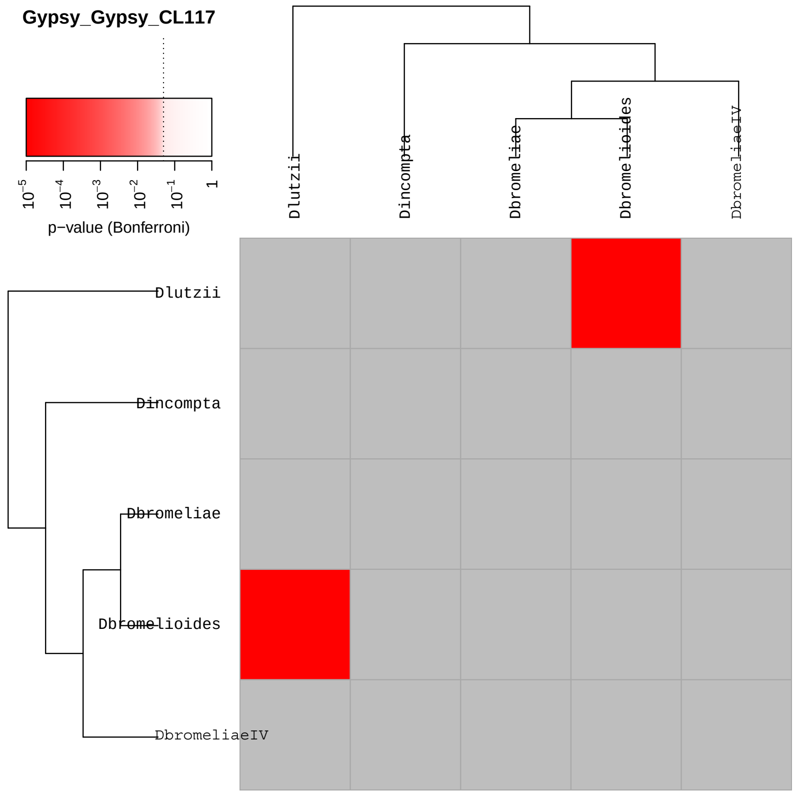
Gypsy_Gypsy_CL117
10^-5 10^-4 10^-3 10^-2 10^-1 1
p−value (Bonferroni)
Dlutzii
Dincompta
Dbromeliae
Dbromelioides
DbromeliaeIV

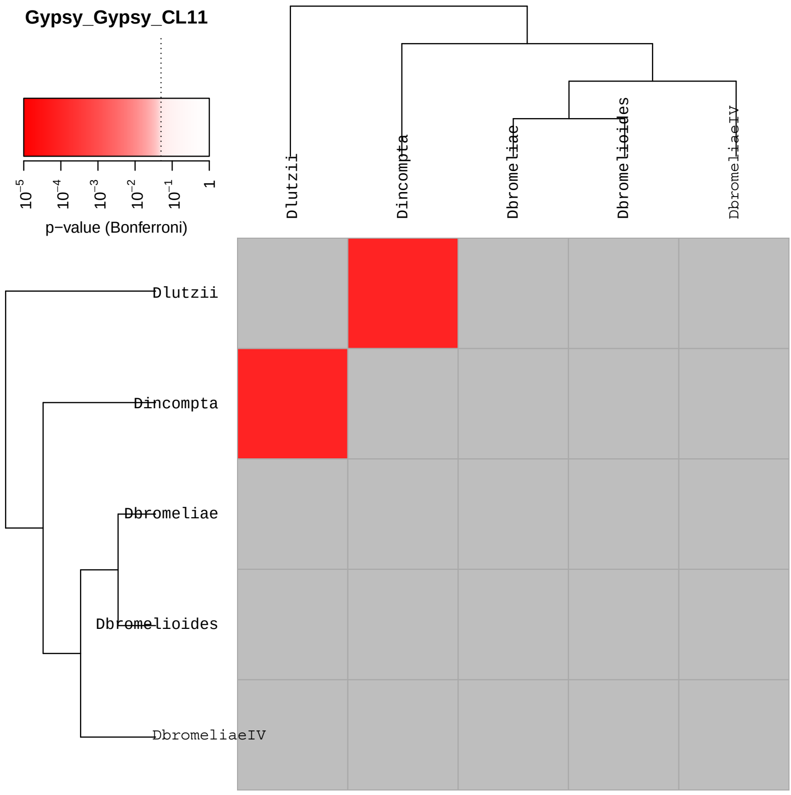
Gypsy_Gypsy_CL11
10^-5 10^-4 10^-3 10^-2 10^-1 1
p−value (Bonferroni)
Dlutzii
Dincompta
Dbromeliae
Dbromelioides
DbromeliaeIV

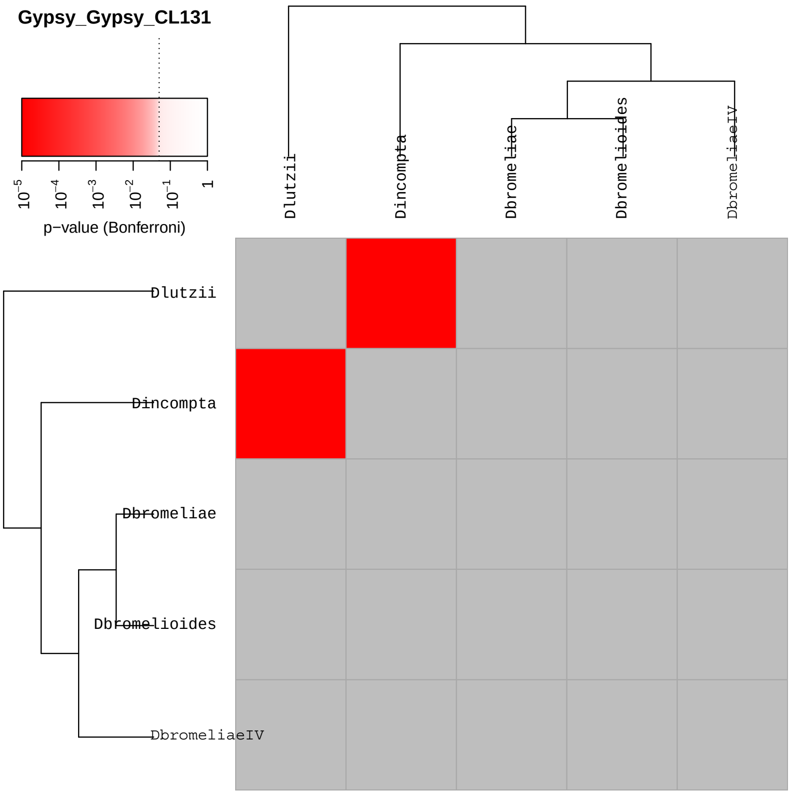
Gypsy_Gypsy_CL131
10^{-5} 10^{-4} 10^{-3} 10^{-2} 10^{-1} 1
p−value (Bonferroni)
Dlutzii
Dincompta
Dbromeliae
Dbromelioides
DbromeliaeIV

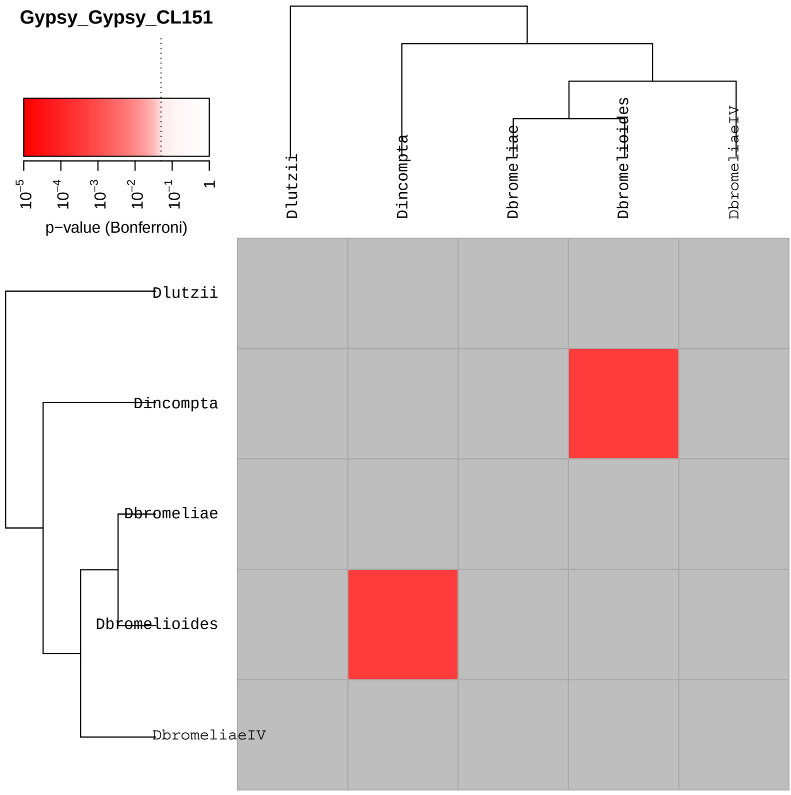
Gypsy_Gypsy_CL151
10^{-5} 10^{-4} 10^{-3} 10^{-2} 10^{-1} 1
p−value (Bonferroni)
Dlutzii
Dincompta
Dbromeliae
Dbromelioides
DbromeliaeIV

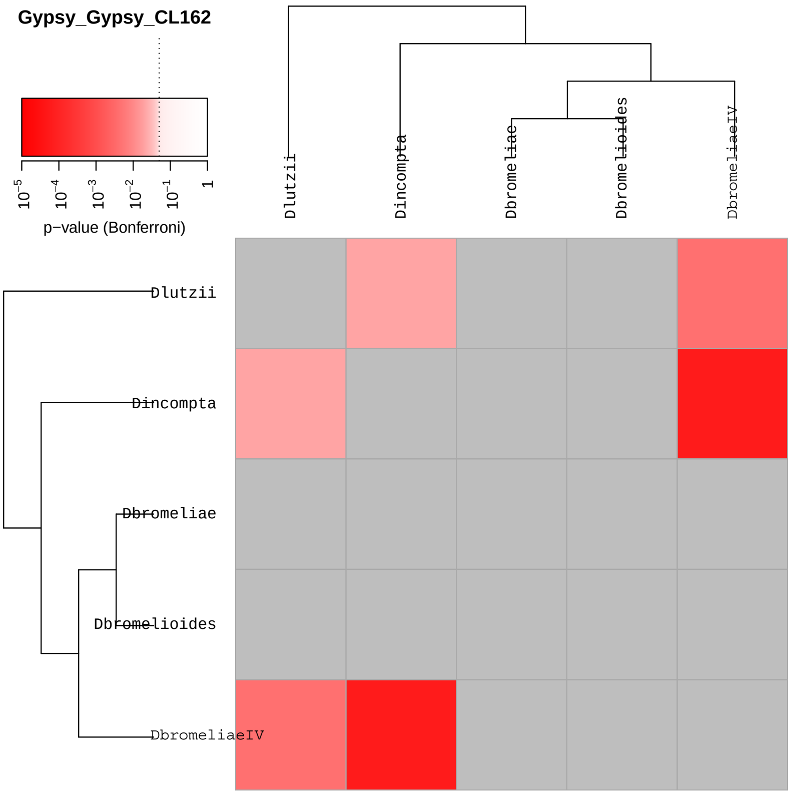
Gypsy_Gypsy_CL162
10^{-5} 10^{-4} 10^{-3} 10^{-2} 10^{-1} 1
p−value (Bonferroni)
Dlutzii
Dincompta
Dbromeliae
Dbromelioides
DbromeliaeIV

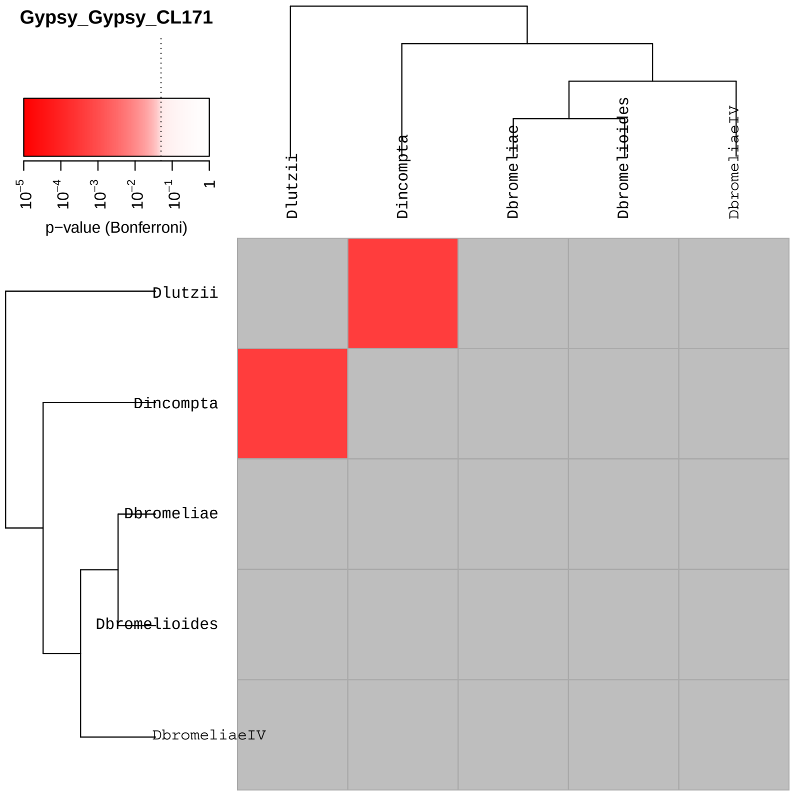
Gypsy_Gypsy_CL171
10^{-5} 10^{-4} 10^{-3} 10^{-2} 10^{-1} 1
p−value (Bonferroni)
Dlutzii
Dincompta
Dbromeliae
Dbromelioides
DbromeliaeIV

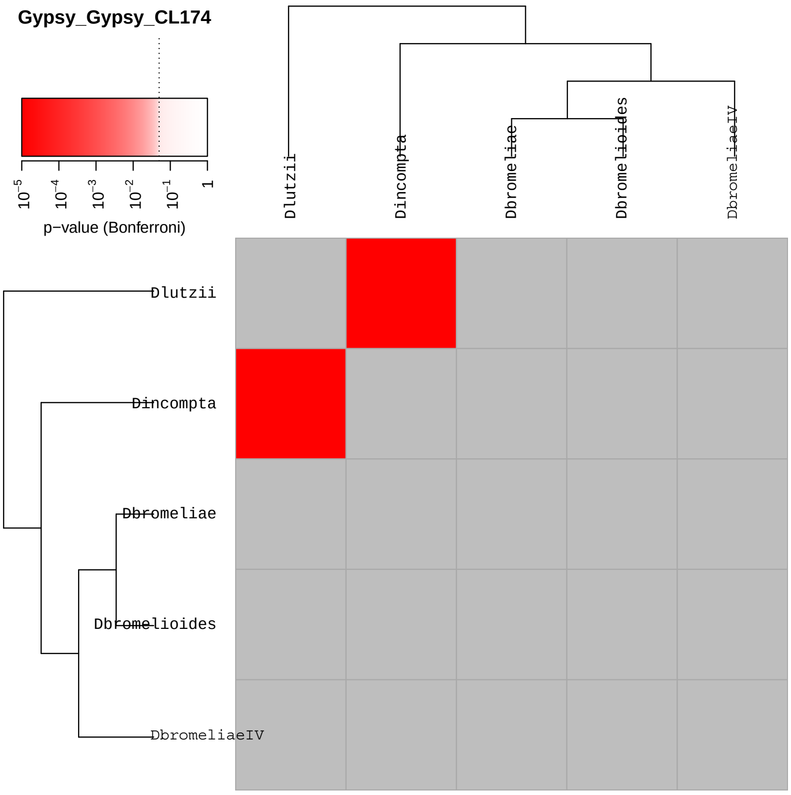
Gypsy_Gypsy_CL174
10^{-5} 10^{-4} 10^{-3} 10^{-2} 10^{-1} 1
p−value (Bonferroni)
Dlutzii
Dincompta
Dbromeliae
Dbromelioides
DbromeliaeIV

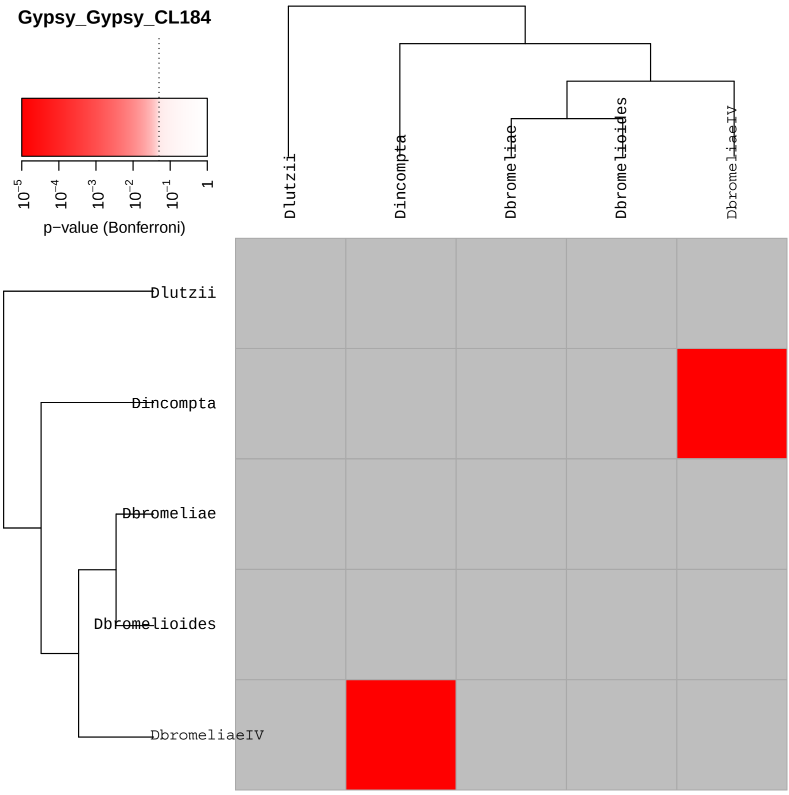
Gypsy_Gypsy_CL184
10^{-5} 10^{-4} 10^{-3} 10^{-2} 10^{-1} 1
p−value (Bonferroni)
Dlutzii
Dincompta
Dbromeliae
Dbromelioides
DbromeliaeIV

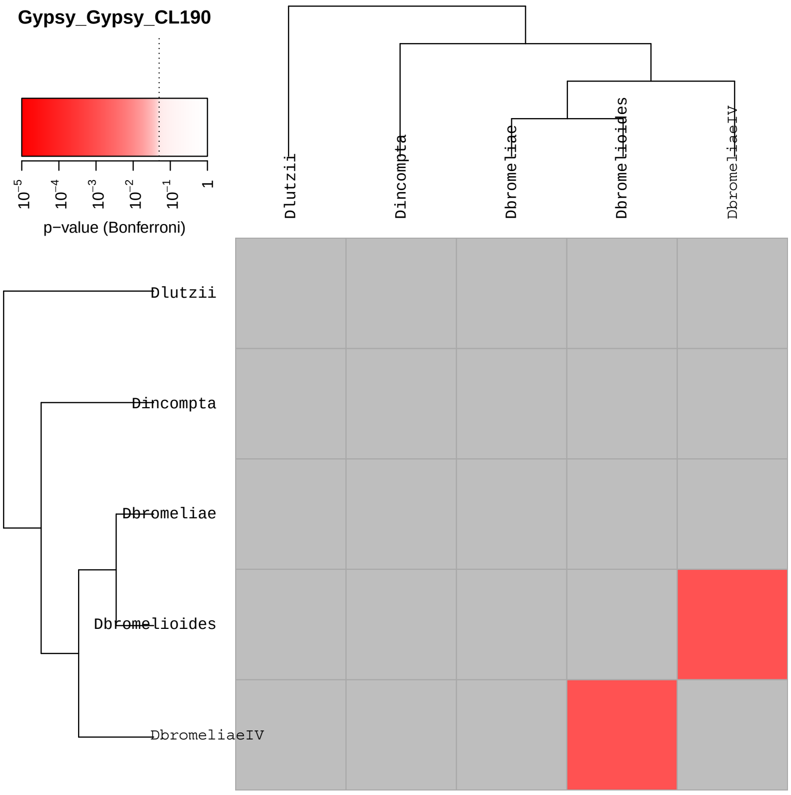
Gypsy_Gypsy_CL190
10^{-5} 10^{-4} 10^{-3} 10^{-2} 10^{-1} 1
p−value (Bonferroni)
Dlutzii
Dincompta
Dbromeliae
Dbromelioides
DbromeliaeIV

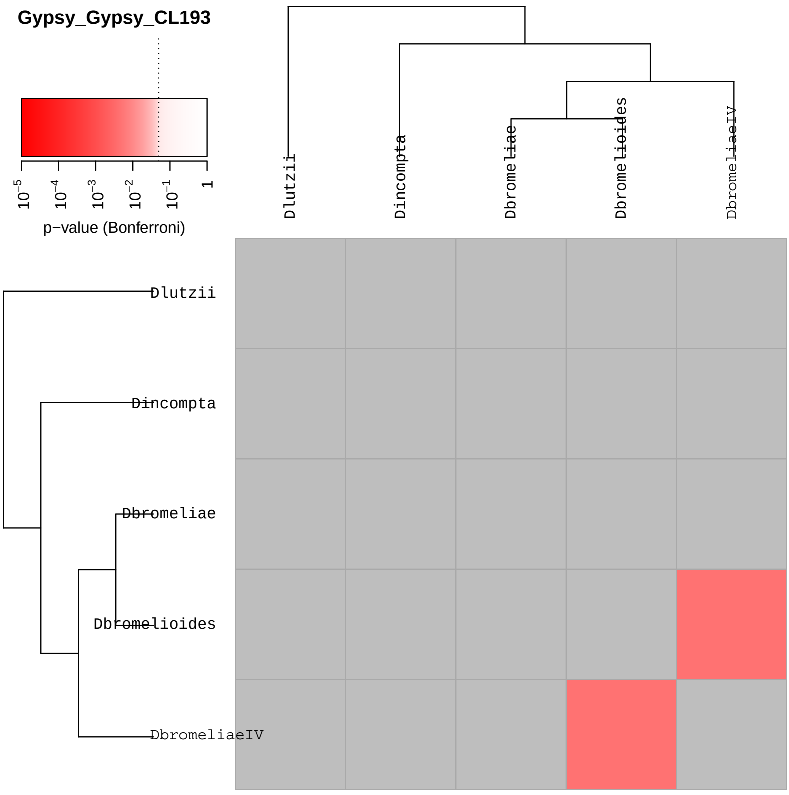
Gypsy_Gypsy_CL193
10^{-5} 10^{-4} 10^{-3} 10^{-2} 10^{-1} 1
p−value (Bonferroni)
Dlutzii
Dincompta
Dbromeliae
Dbromelioides
DbromeliaeIV

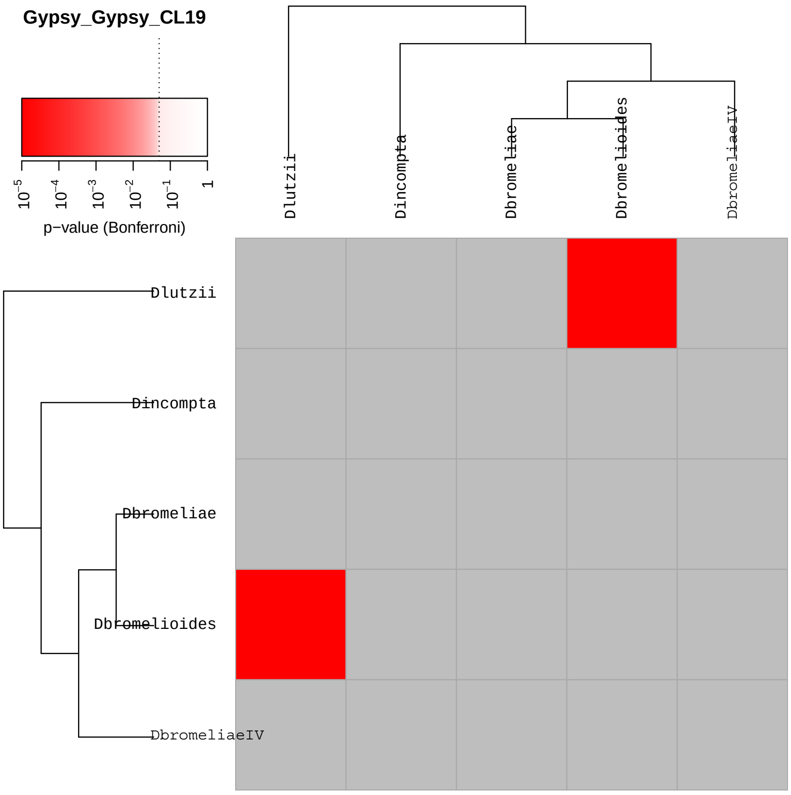

Gypsy_Gypsy_CL19
10^{-5} 10^{-4} 10^{-3} 10^{-2} 10^{-1} 1
p−value (Bonferroni)
Dlutzii
Dincompta
Dbromeliae
Dbromelioides
DbromeliaeIV

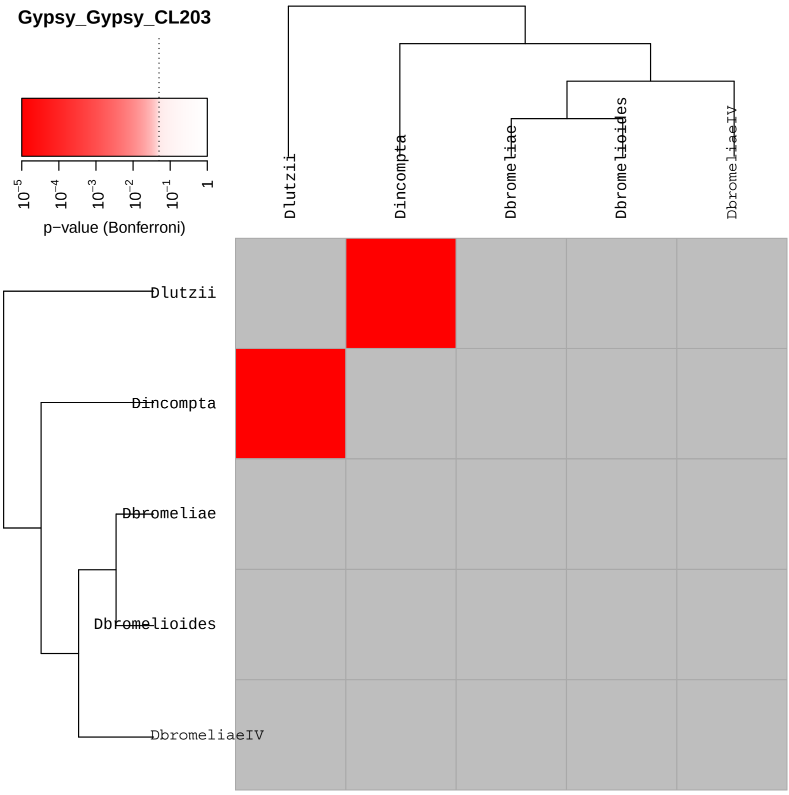

Gypsy_Gypsy_CL203
10^{-5} 10^{-4} 10^{-3} 10^{-2} 10^{-1} 1
p−value (Bonferroni)
Dlutzii
Dincompta
Dbromeliae
Dbromelioides
DbromeliaeIV

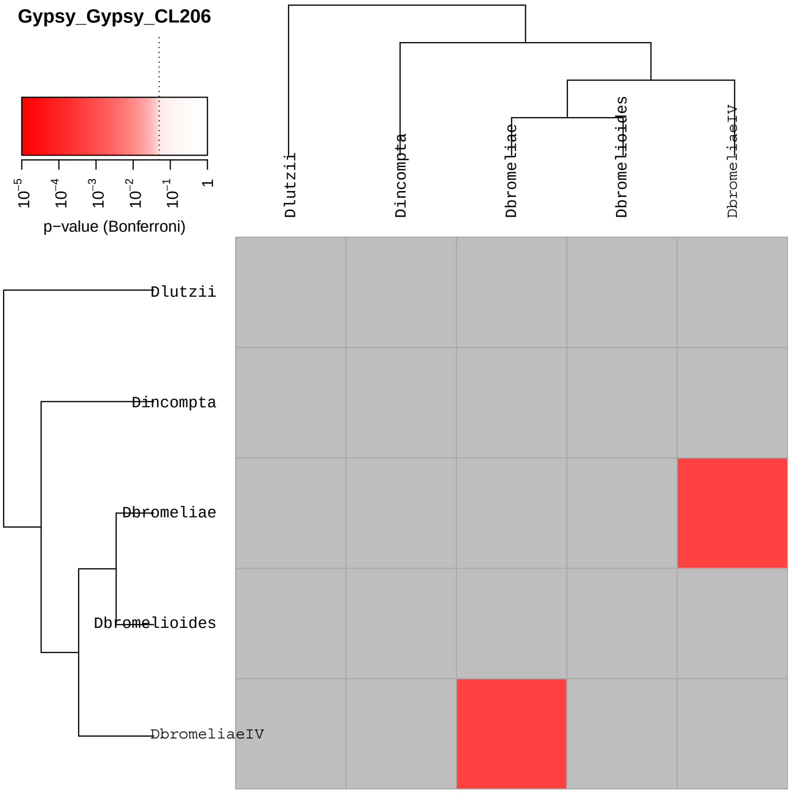

Gypsy_Gypsy_CL206
10^{-5} 10^{-4} 10^{-3} 10^{-2} 10^{-1} 1
p−value (Bonferroni)
Dlutzii
Dincompta
Dbromeliae
Dbromelioides
DbromeliaeIV

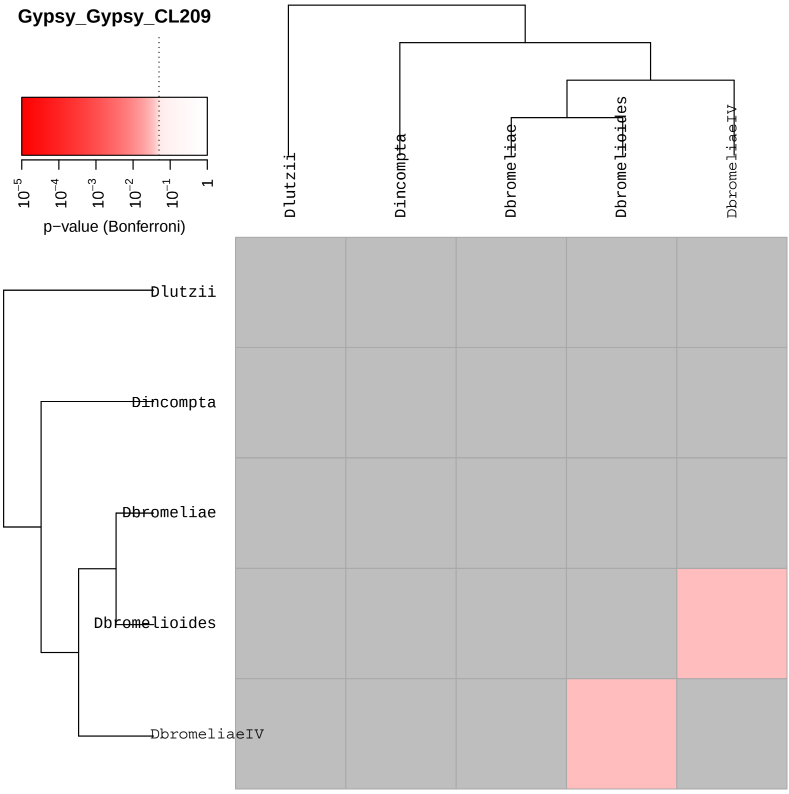

Gypsy_Gypsy_CL209
10^{-5} 10^{-4} 10^{-3} 10^{-2} 10^{-1} 1
p−value (Bonferroni)
Dlutzii
Dincompta
Dbromeliae
Dbromelioides
DbromeliaeIV

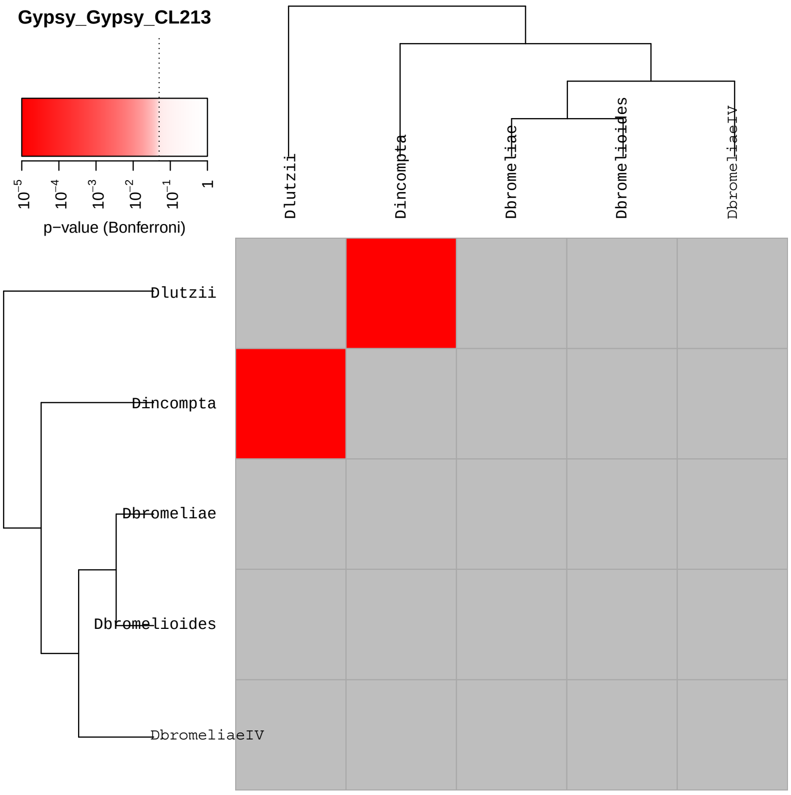
Gypsy_Gypsy_CL213
p−value (Bonferroni)
Dlutzii
Dincompta
Dbromeliae
Dbromelioides
DbromeliaeIV

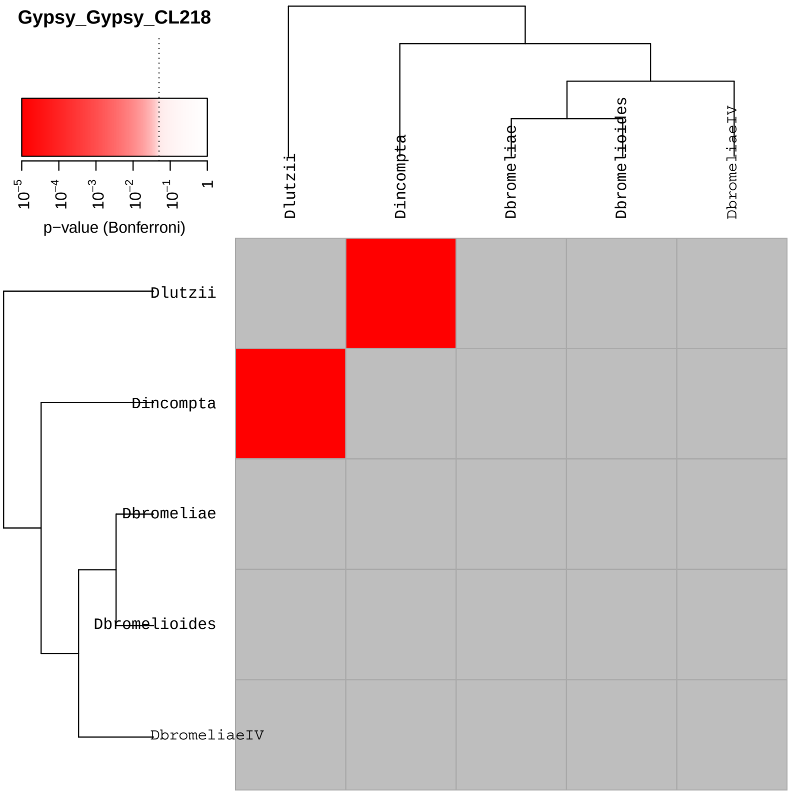
Gypsy_Gypsy_CL218
p−value (Bonferroni)
Dlutzii
Dincompta
Dbromeliae
Dbromelioides
DbromeliaeIV

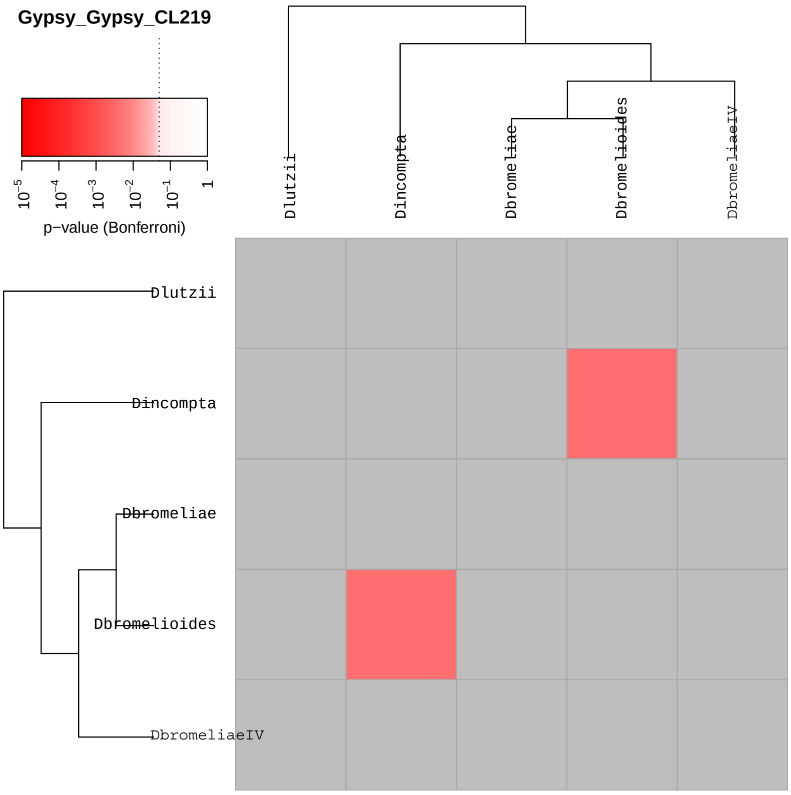
Gypsy_Gypsy_CL219
p−value (Bonferroni)
Dlutzii
Dincompta
Dbromeliae
Dbromelioides
DbromeliaeIV

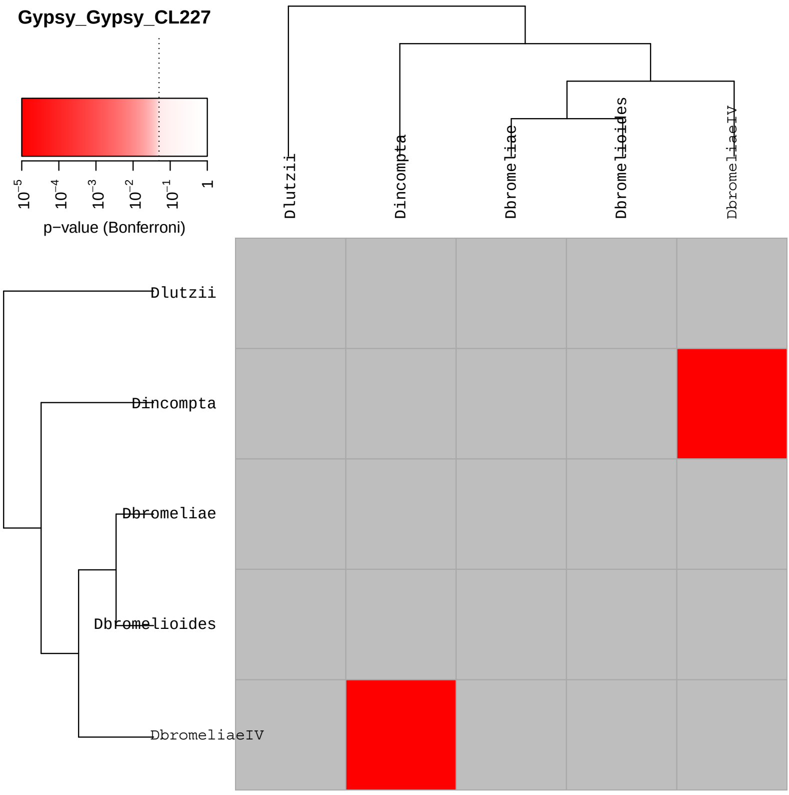
Gypsy_Gypsy_CL227
p−value (Bonferroni)
Dlutzii
Dincompta
Dbromeliae
Dbromelioides
DbromeliaeIV

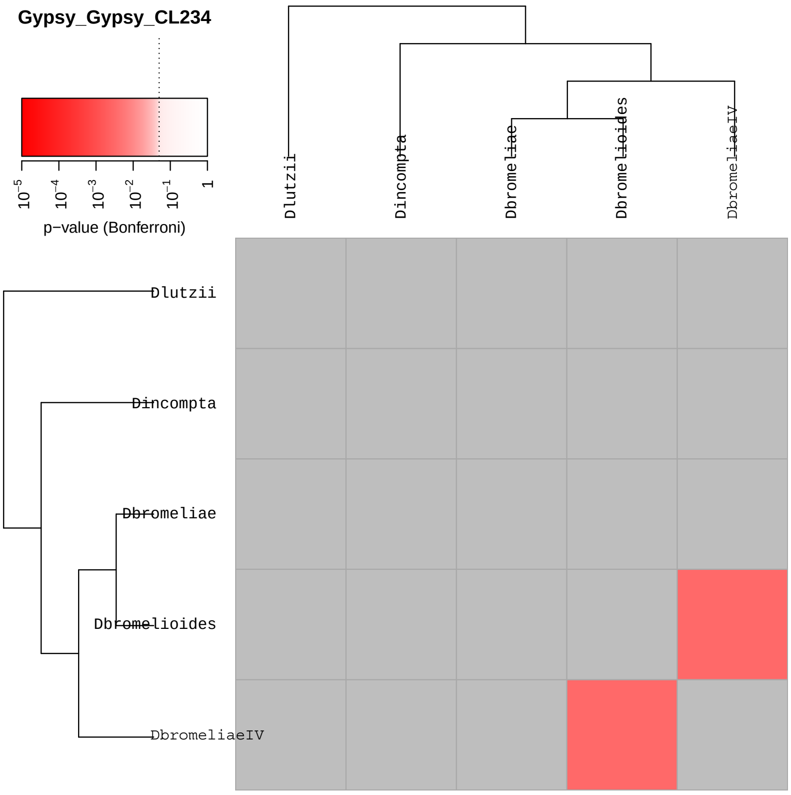
Gypsy_Gypsy_CL234
p−value (Bonferroni)
Dlutzii
Dincompta
Dbromeliae
Dbromelioides
DbromeliaeIV

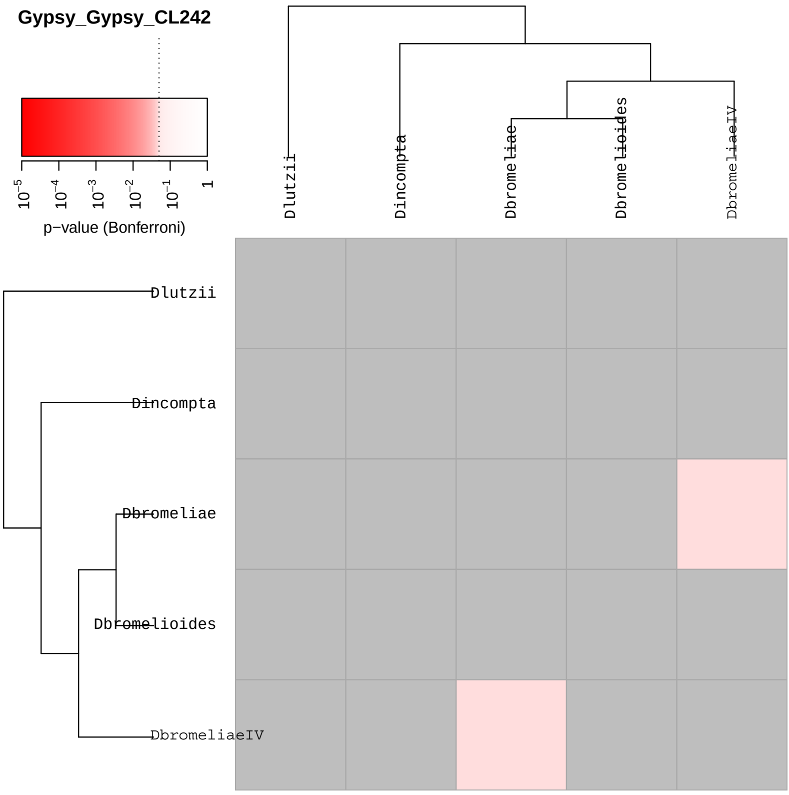
Gypsy_Gypsy_CL242
p−value (Bonferroni)
Dlutzii
Dincompta
Dbromeliae
Dbromelioides
DbromeliaeIV

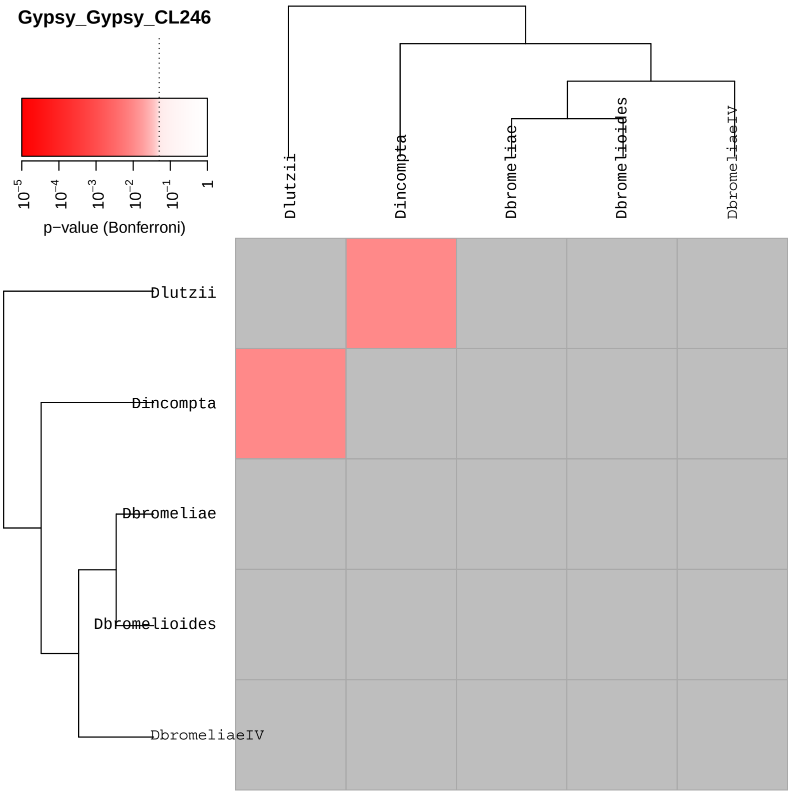
Gypsy_Gypsy_CL246
p−value (Bonferroni)
Dlutzii
Dincompta
Dbromeliae
Dbromelioides
DbromeliaeIV

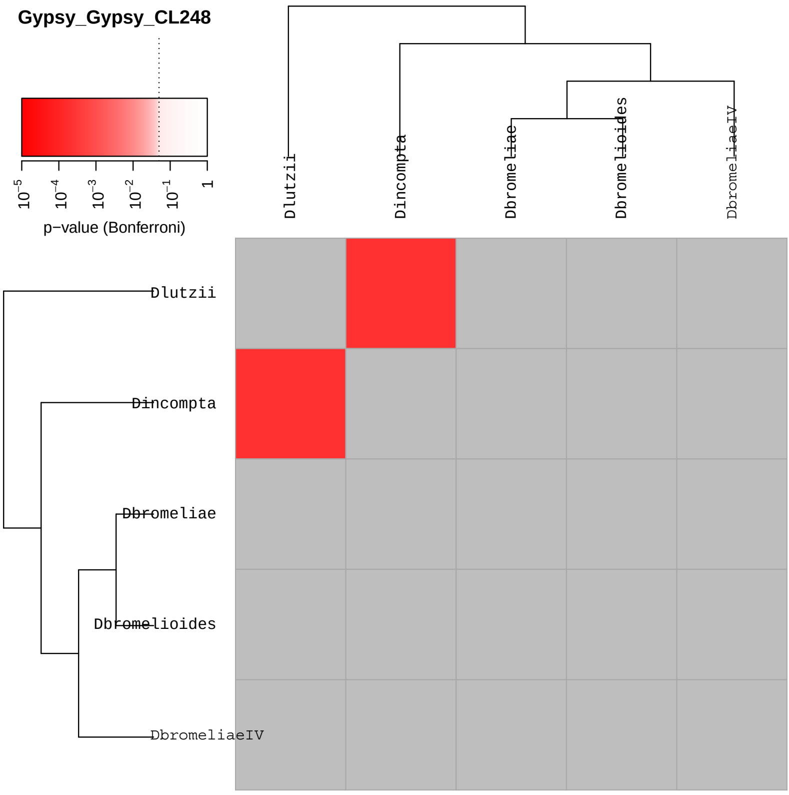
Gypsy_Gypsy_CL248
p−value (Bonferroni)
Dlutzii
Dincompta
Dbromeliae
Dbromelioides
DbromeliaeIV

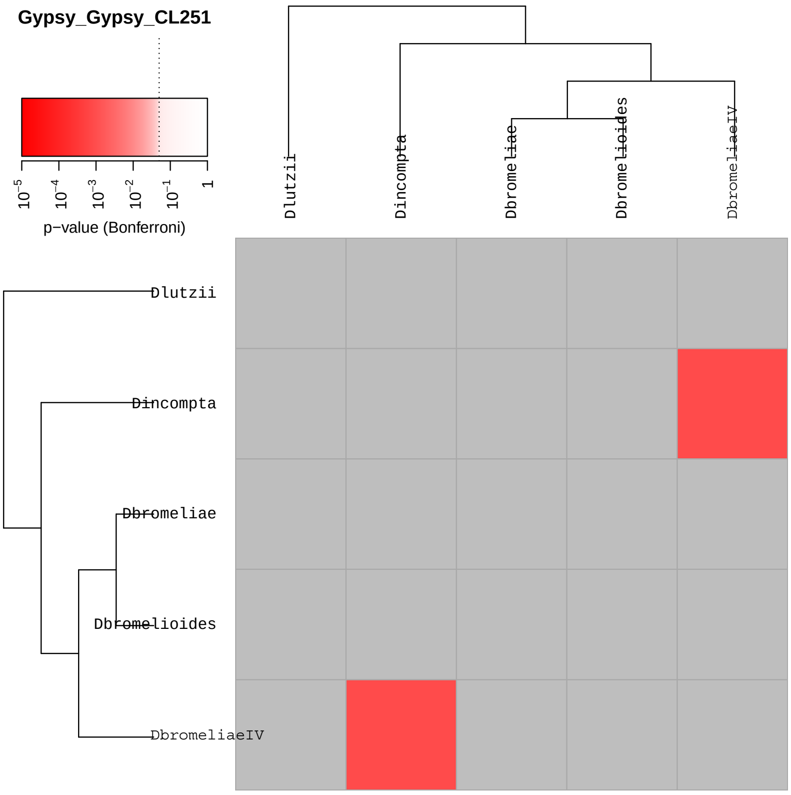
Gypsy_Gypsy_CL251
p−value (Bonferroni)
Dlutzii
Dincompta
Dbromeliae
Dbromelioides
DbromeliaeIV

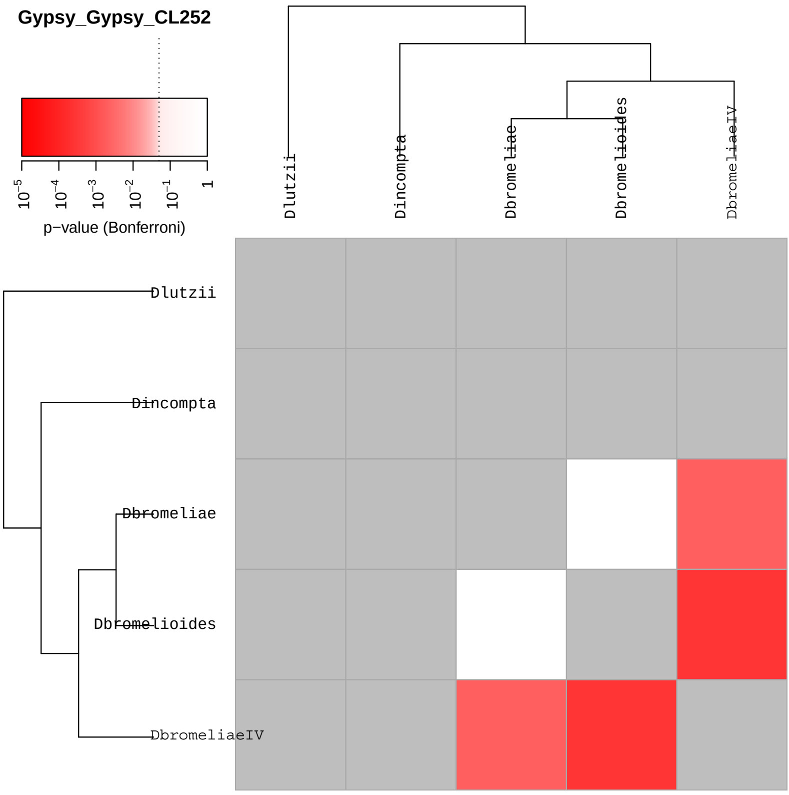
Gypsy_Gypsy_CL252
p−value (Bonferroni)
Dlutzii
Dincompta
Dbromeliae
Dbromelioides
DbromeliaeIV

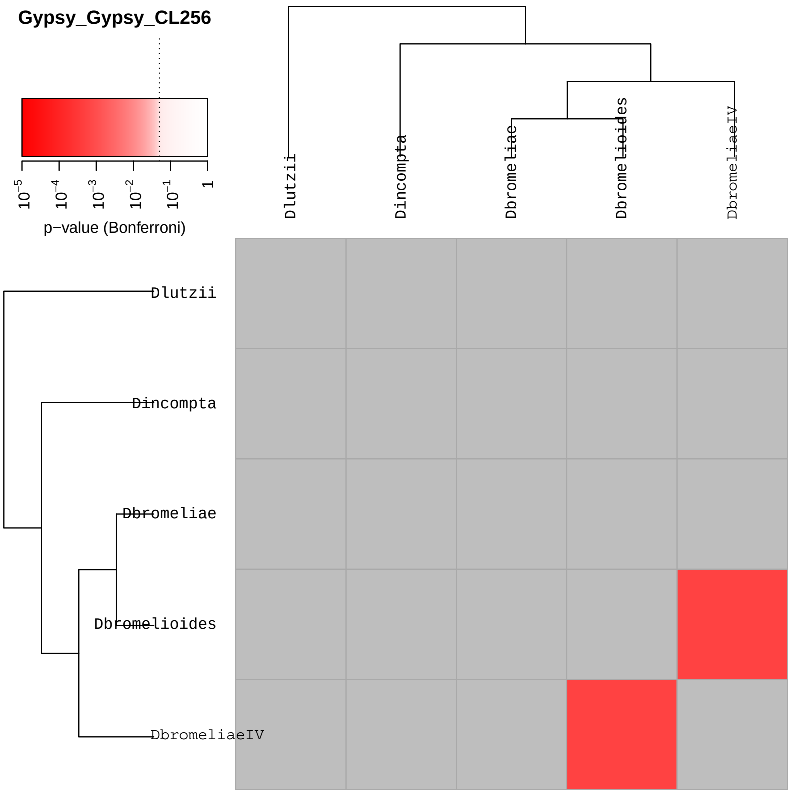
Gypsy_Gypsy_CL256
p−value (Bonferroni)
Dlutzii
Dincompta
Dbromeliae
Dbromelioides
DbromeliaeIV

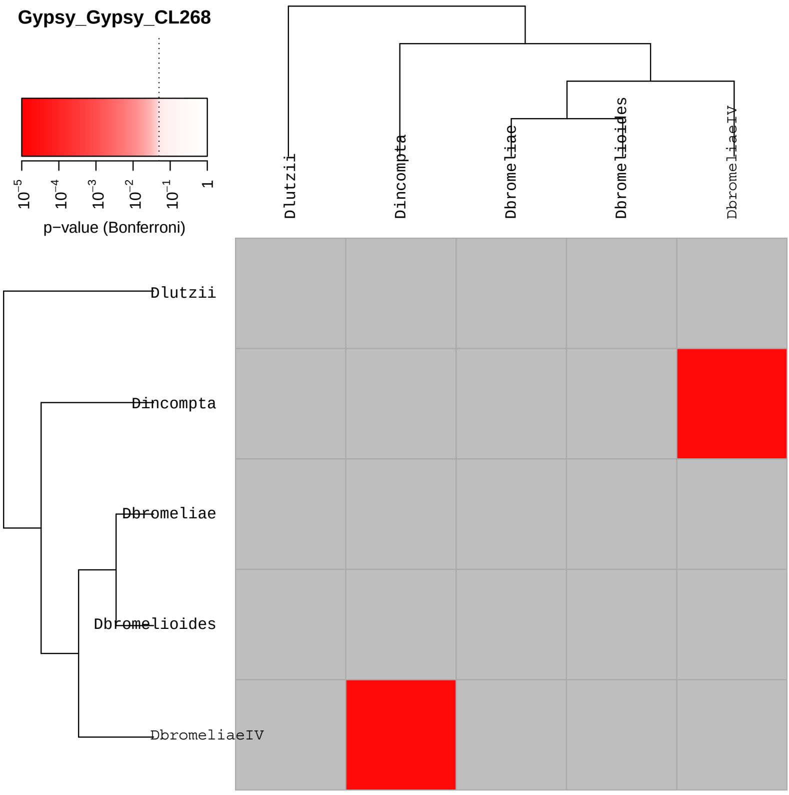
Gypsy_Gypsy_CL268
p−value (Bonferroni)
Dlutzii
Dincompta
Dbromeliae
Dbromelioides
DbromeliaeIV

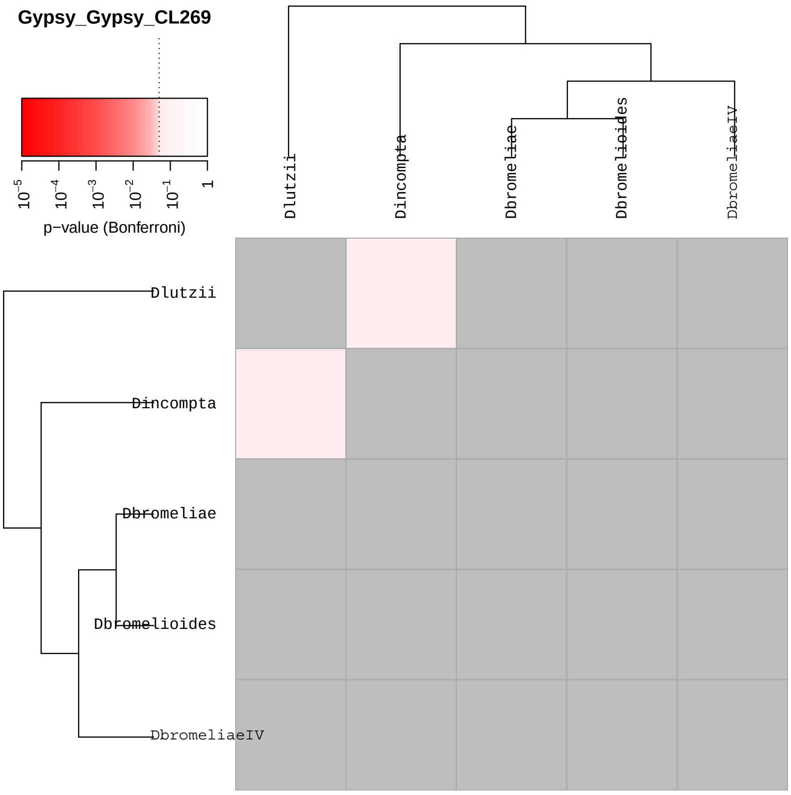
Gypsy_Gypsy_CL269
p−value (Bonferroni)
Dlutzii
Dincompta
Dbromeliae
Dbromelioides
DbromeliaeIV

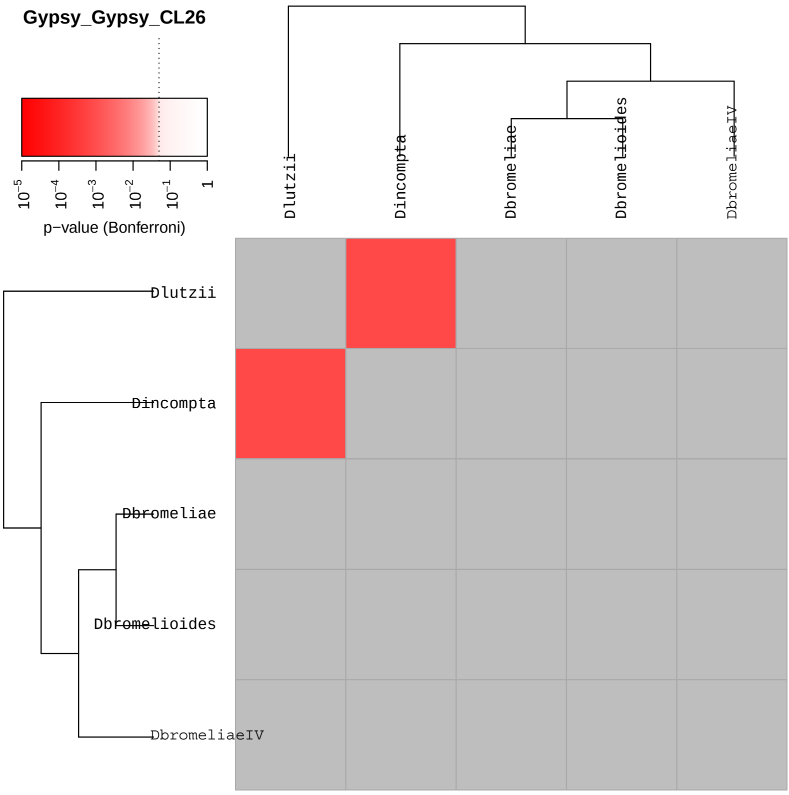
Gypsy_Gypsy_CL26
p−value (Bonferroni)
Dlutzii
Dincompta
Dbromeliae
Dbromelioides
DbromeliaeIV

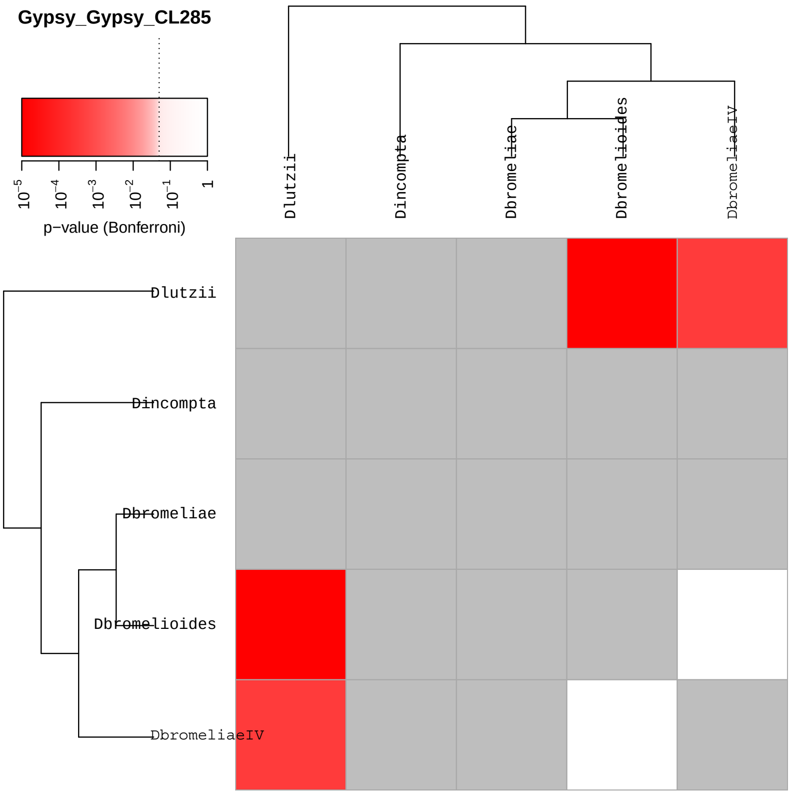
Gypsy_Gypsy_CL285
p−value (Bonferroni)
Dlutzii
Dincompta
Dbromeliae
Dbromelioides
DbromeliaeIV

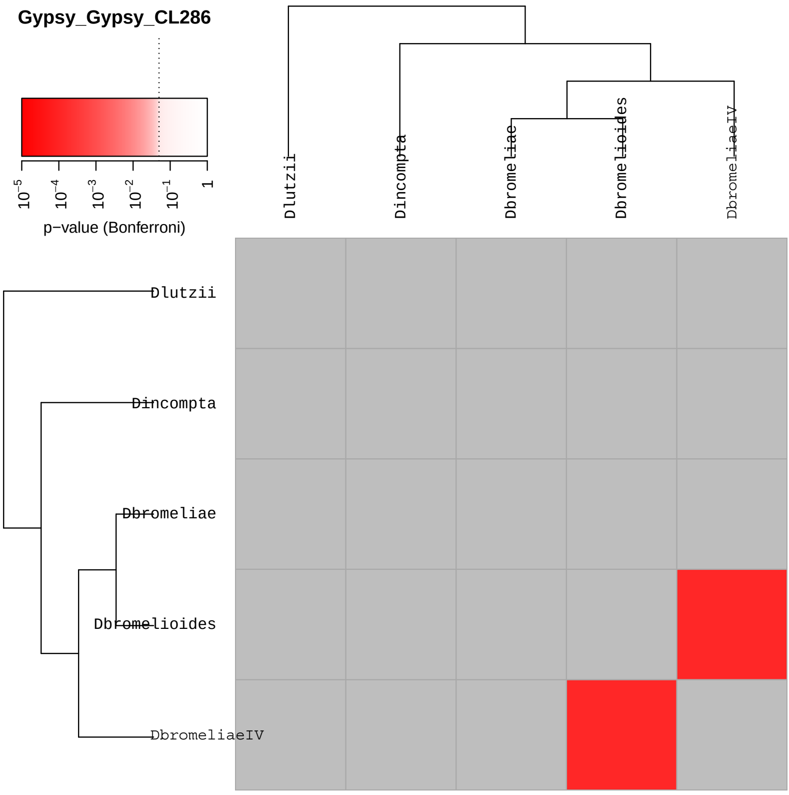
Gypsy_Gypsy_CL286
p−value (Bonferroni)
Dlutzii
Dincompta
Dbromeliae
Dbromelioides
DbromeliaeIV

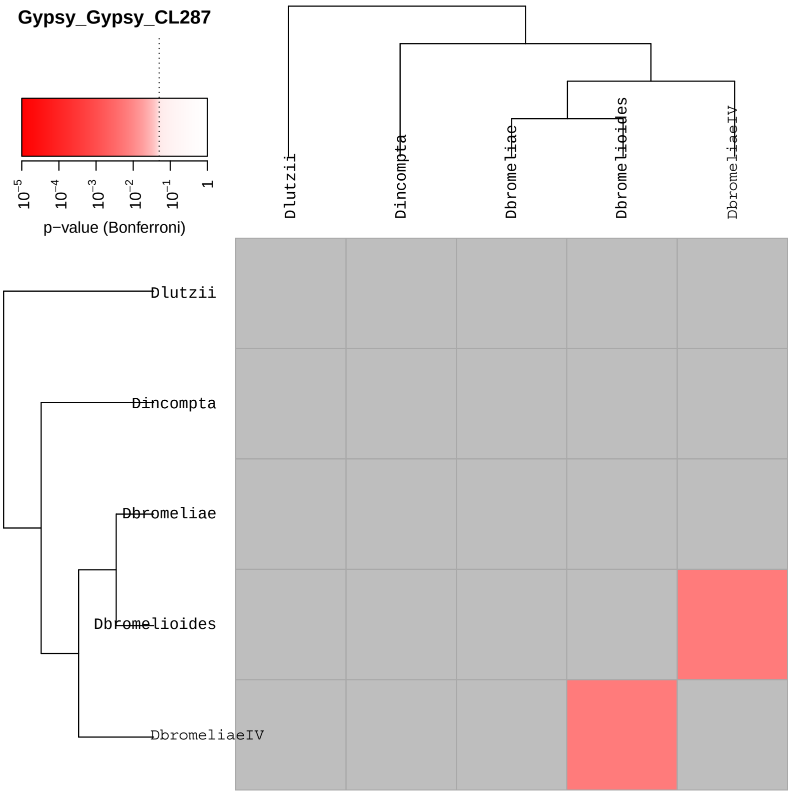
Gypsy_Gypsy_CL287
10^{-5} 10^{-4} 10^{-3} 10^{-2} 10^{-1} 1
p−value (Bonferroni)
Dlutzii
Dincompta
Dbromeliae
Dbromelioides
DbromeliaeIV

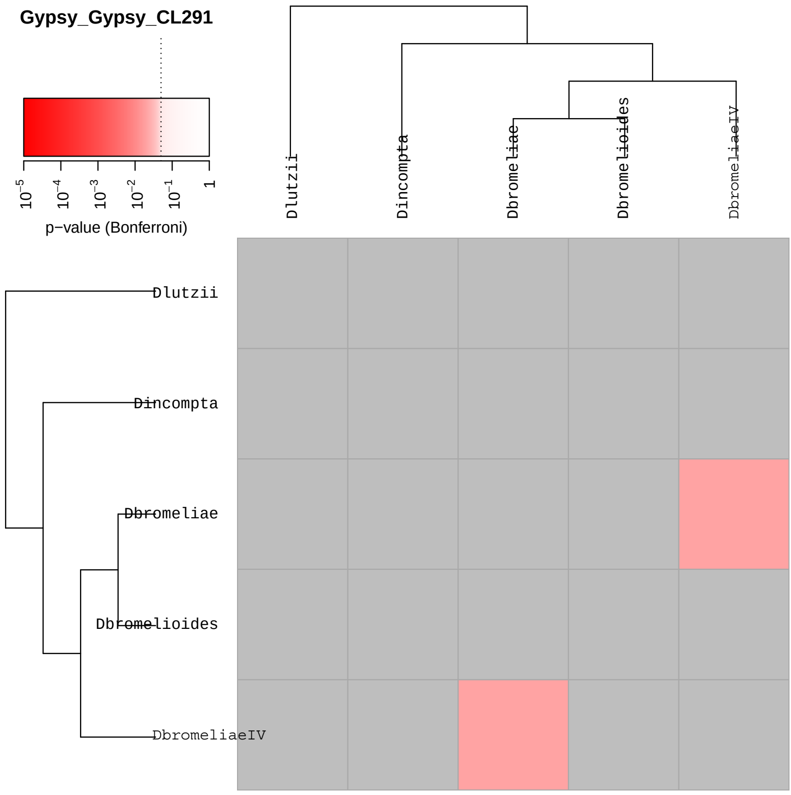
Gypsy_Gypsy_CL291
10^{-5} 10^{-4} 10^{-3} 10^{-2} 10^{-1} 1
p−value (Bonferroni)
Dlutzii
Dincompta
Dbromeliae
Dbromelioides
DbromeliaeIV

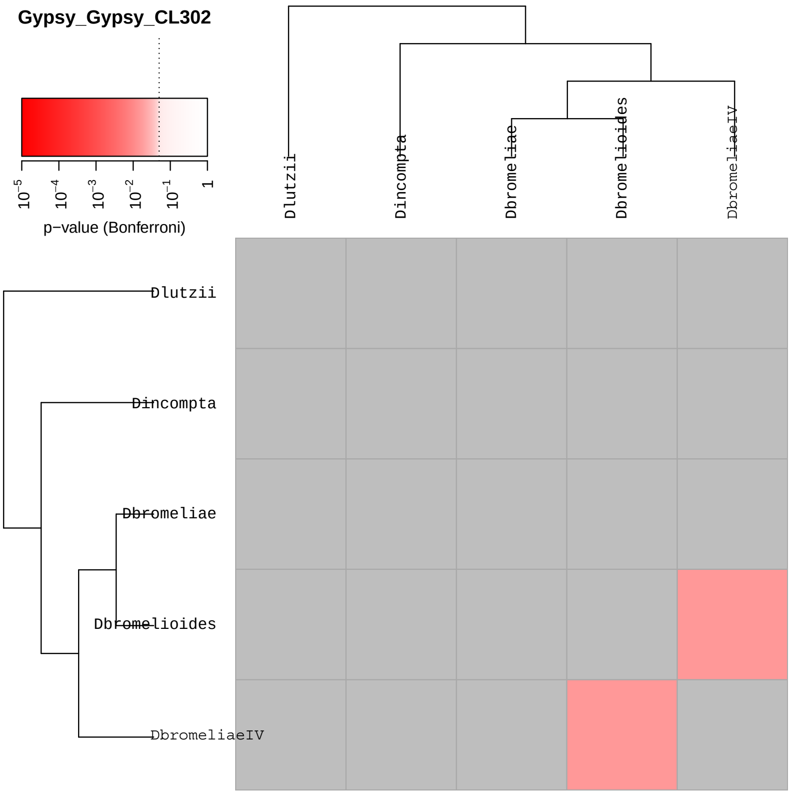
Gypsy_Gypsy_CL302
10^{-5} 10^{-4} 10^{-3} 10^{-2} 10^{-1} 1
p−value (Bonferroni)
Dlutzii
Dincompta
Dbromeliae
Dbromelioides
DbromeliaeIV

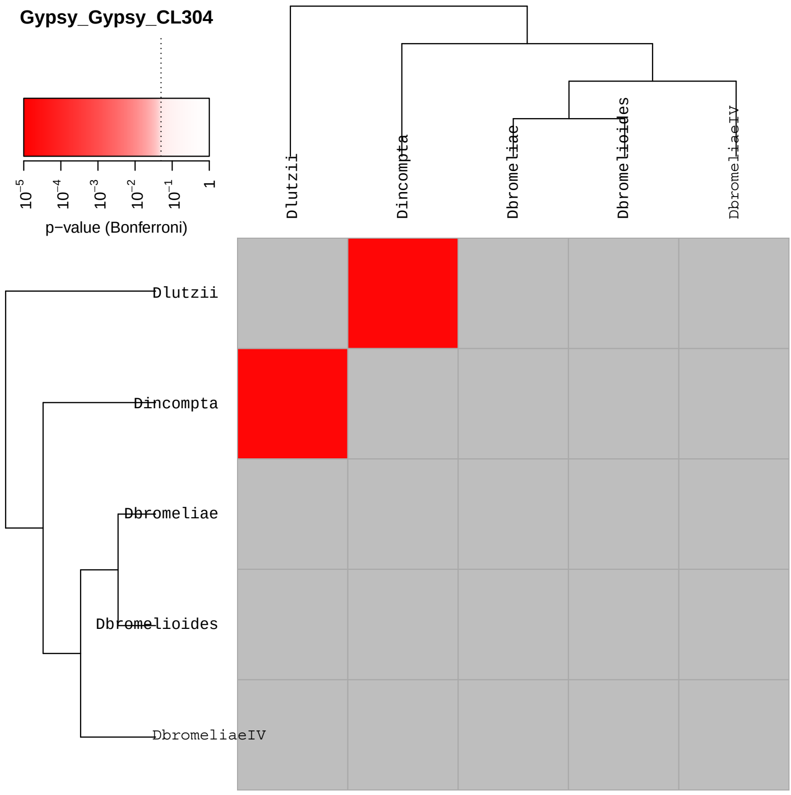
Gypsy_Gypsy_CL304
10^{-5} 10^{-4} 10^{-3} 10^{-2} 10^{-1} 1
p−value (Bonferroni)
Dlutzii
Dincompta
Dbromeliae
Dbromelioides
DbromeliaeIV

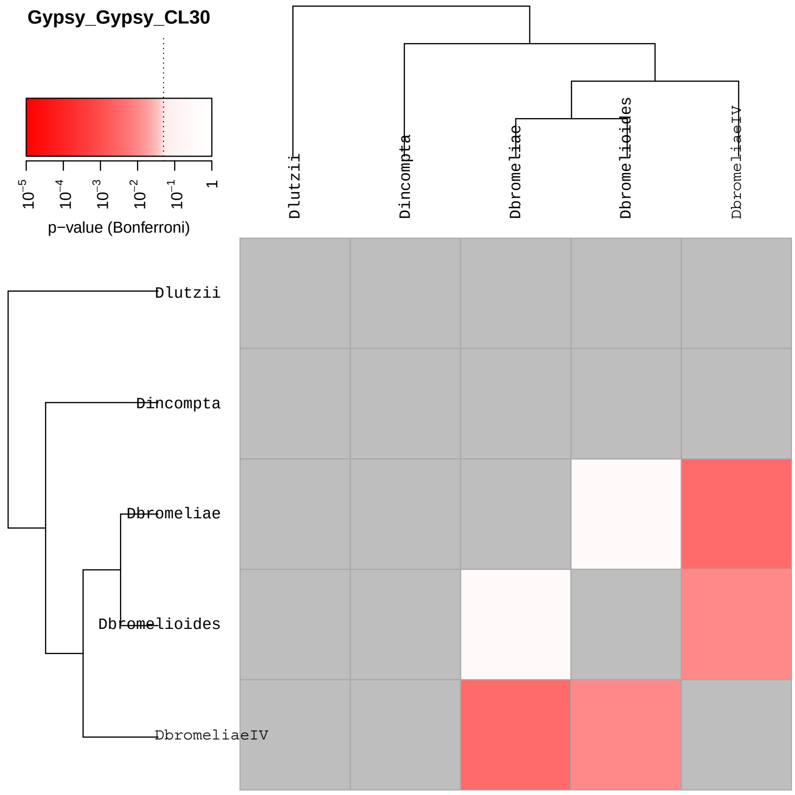
Gypsy_Gypsy_CL30
10^-5 10^-4 10^-3 10^-2 10^-1 1
p−value (Bonferroni)
Dlutzii
Dincompta
Dbromeliae
Dbromelioides
DbromeliaeIV

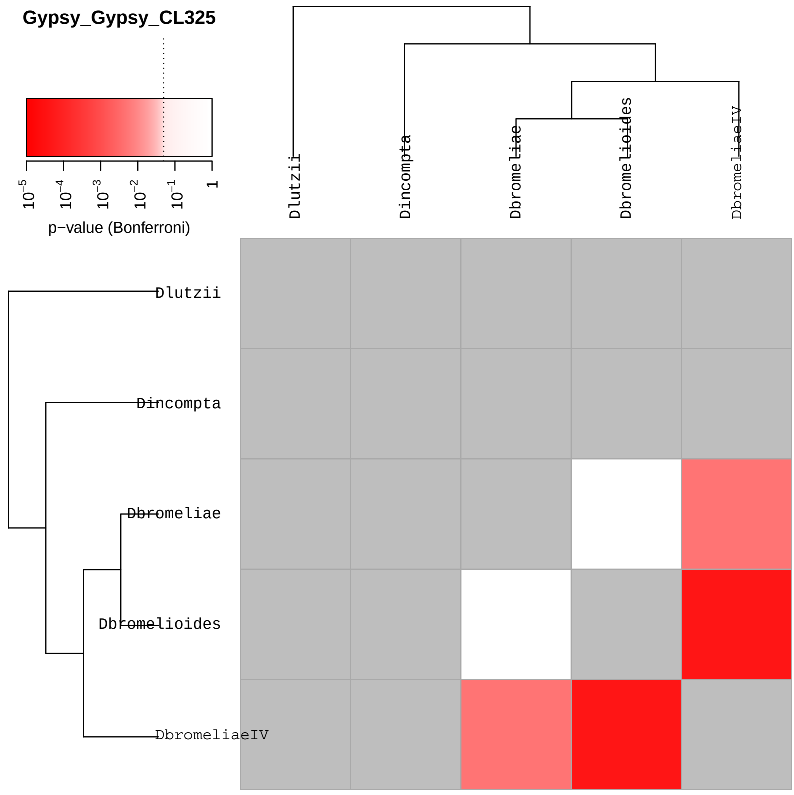
Gypsy_Gypsy_CL325
10^-5 10^-4 10^-3 10^-2 10^-1 1
p−value (Bonferroni)
Dlutzii
Dincompta
Dbromeliae
Dbromelioides
DbromeliaeIV

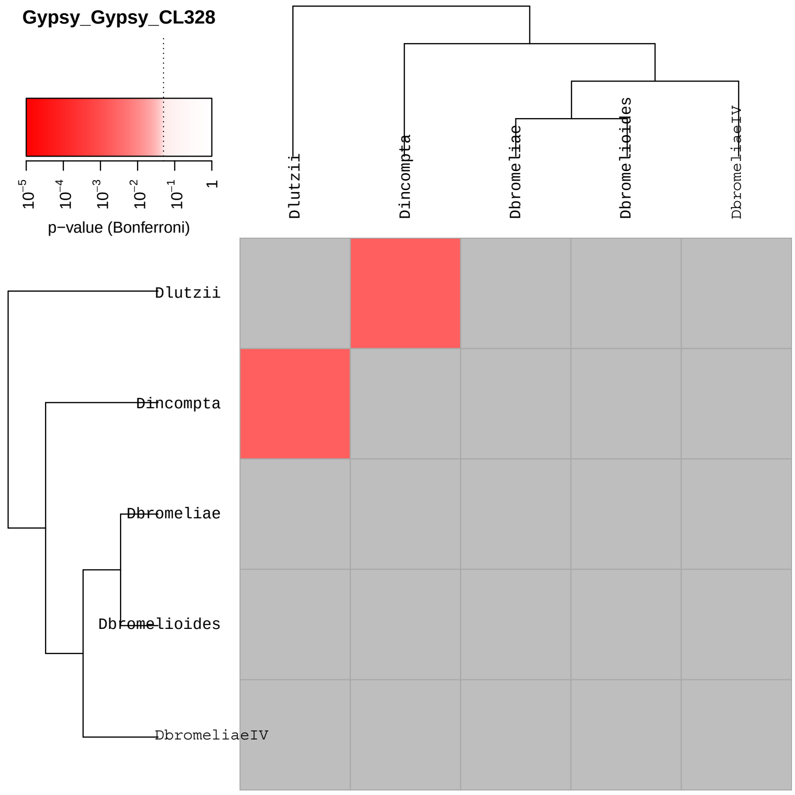
Gypsy_Gypsy_CL328
10^-5 10^-4 10^-3 10^-2 10^-1 1
p−value (Bonferroni)
Dlutzii
Dincompta
Dbromeliae
Dbromelioides
DbromeliaeIV

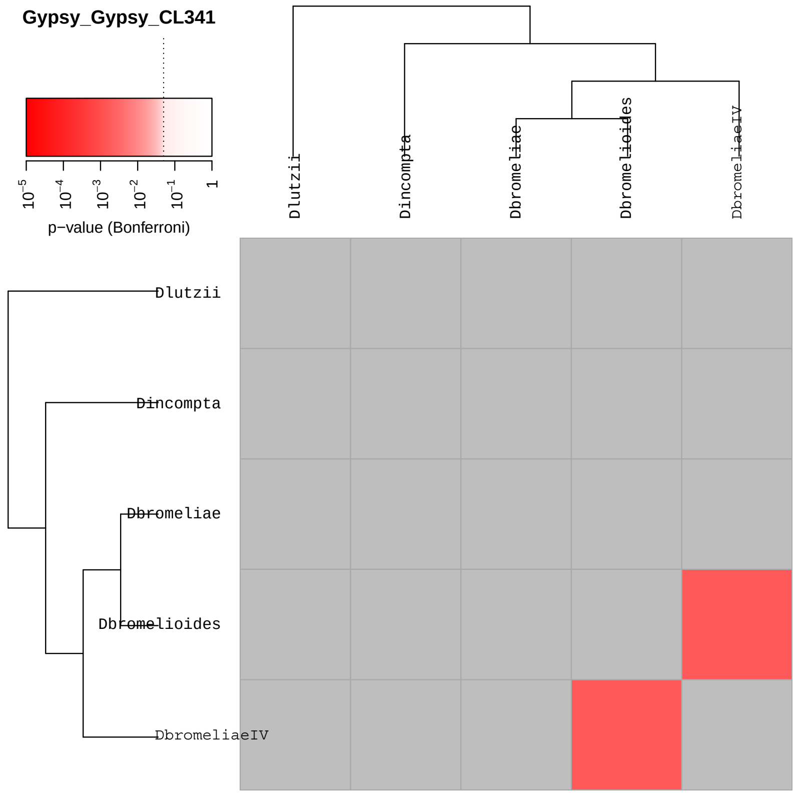
Gypsy_Gypsy_CL341
10^-5 10^-4 10^-3 10^-2 10^-1 1
p−value (Bonferroni)
Dlutzii
Dincompta
Dbromeliae
Dbromelioides
DbromeliaeIV

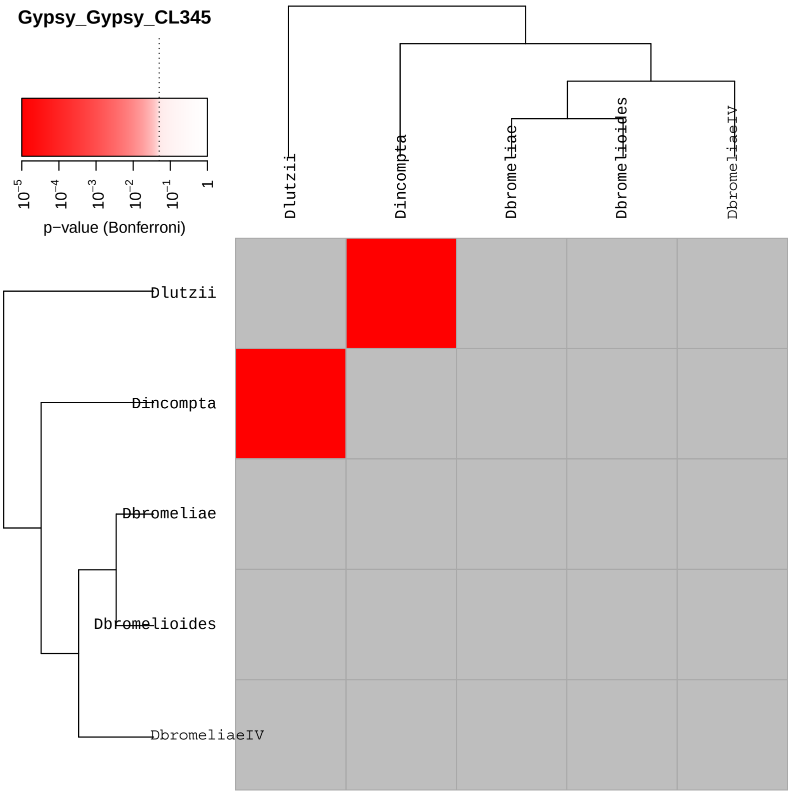
Gypsy_Gypsy_CL345
10^{-5} 10^{-4} 10^{-3} 10^{-2} 10^{-1} 1
p−value (Bonferroni)
Dlutzii
Dincompta
Dbromeliae
Dbromelioides
DbromeliaeIV

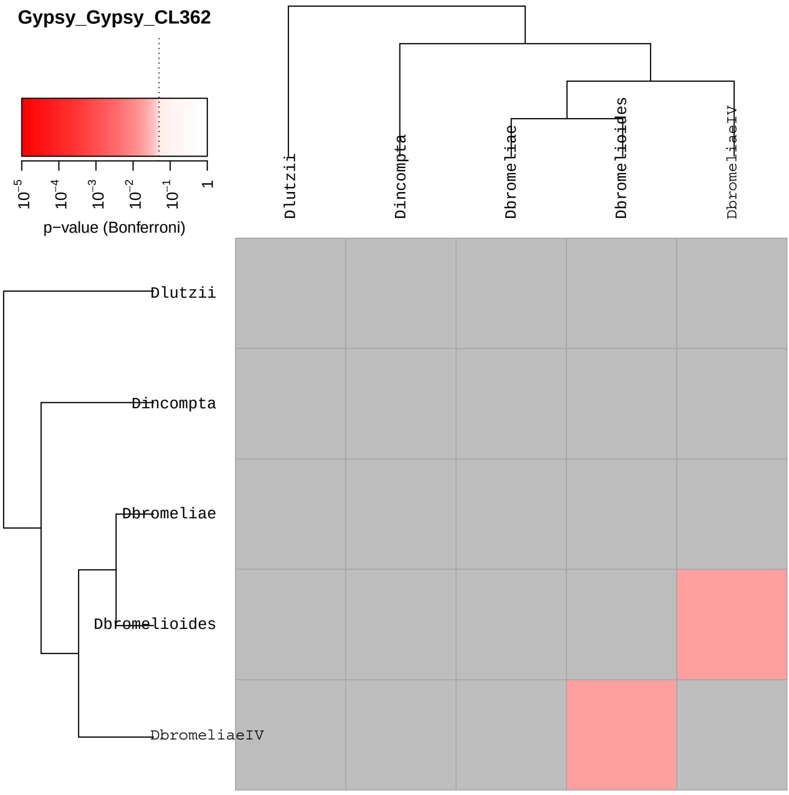
Gypsy_Gypsy_CL362
10^{-5} 10^{-4} 10^{-3} 10^{-2} 10^{-1} 1
p−value (Bonferroni)
Dlutzii
Dincompta
Dbromeliae
Dbromelioides
DbromeliaeIV

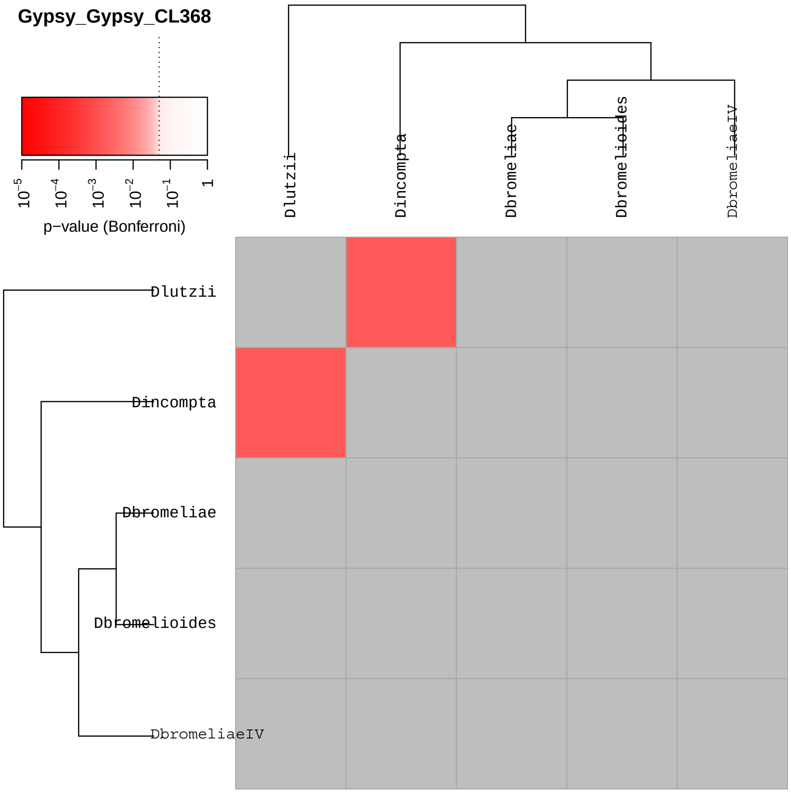
Gypsy_Gypsy_CL368
10^{-5} 10^{-4} 10^{-3} 10^{-2} 10^{-1} 1
p−value (Bonferroni)
Dlutzii
Dincompta
Dbromeliae
Dbromelioides
DbromeliaeIV

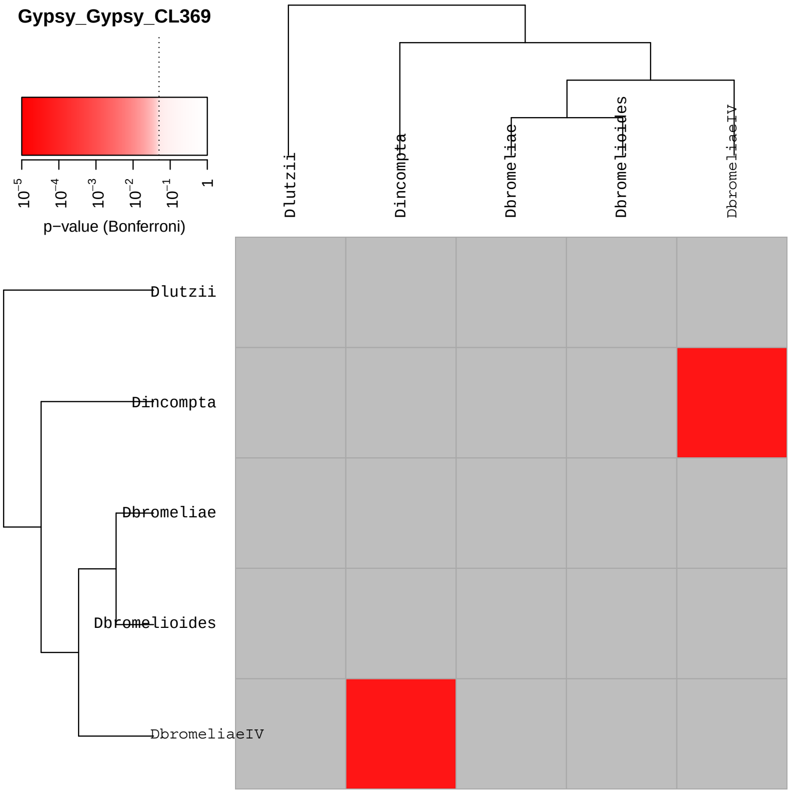
Gypsy_Gypsy_CL369
10^{-5} 10^{-4} 10^{-3} 10^{-2} 10^{-1} 1
p−value (Bonferroni)
Dlutzii
Dincompta
Dbromeliae
Dbromelioides
DbromeliaeIV

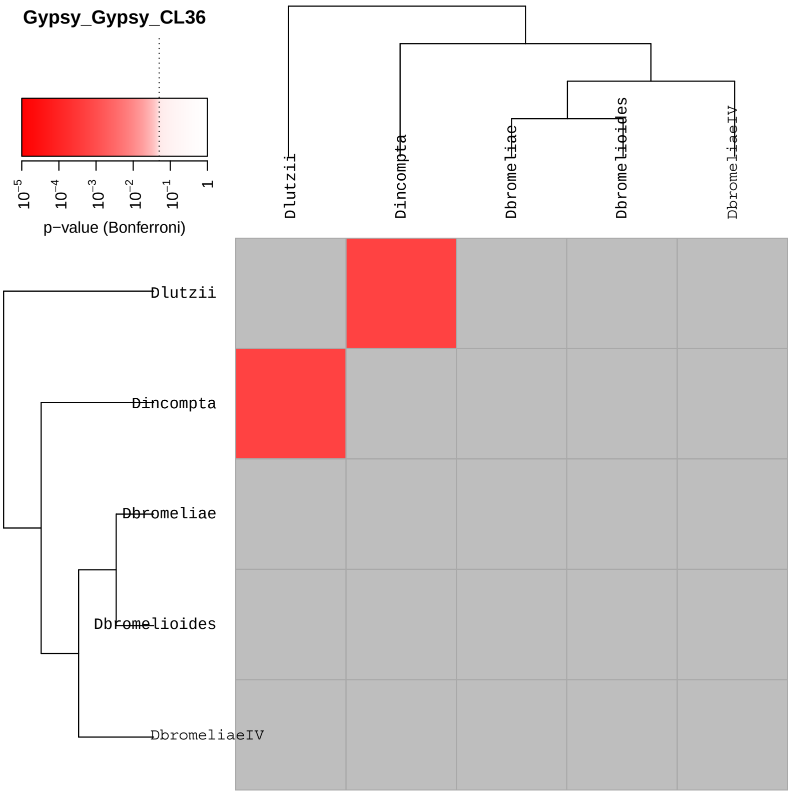
Gypsy_Gypsy_CL36
10^{-5} 10^{-4} 10^{-3} 10^{-2} 10^{-1} 1
p−value (Bonferroni)
Dlutzii
Dincompta
Dbromeliae
Dbromelioides
DbromeliaeIV

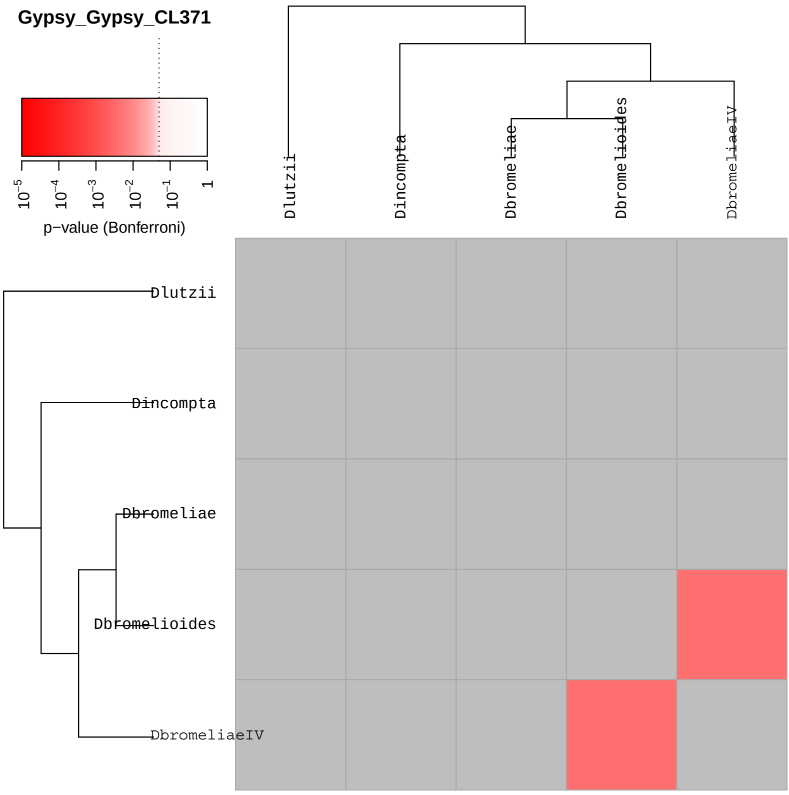
Gypsy_Gypsy_CL371
10^{-5} 10^{-4} 10^{-3} 10^{-2} 10^{-1} 1
p−value (Bonferroni)
Dlutzii
Dincompta
Dbromeliae
Dbromelioides
DbromeliaeIV

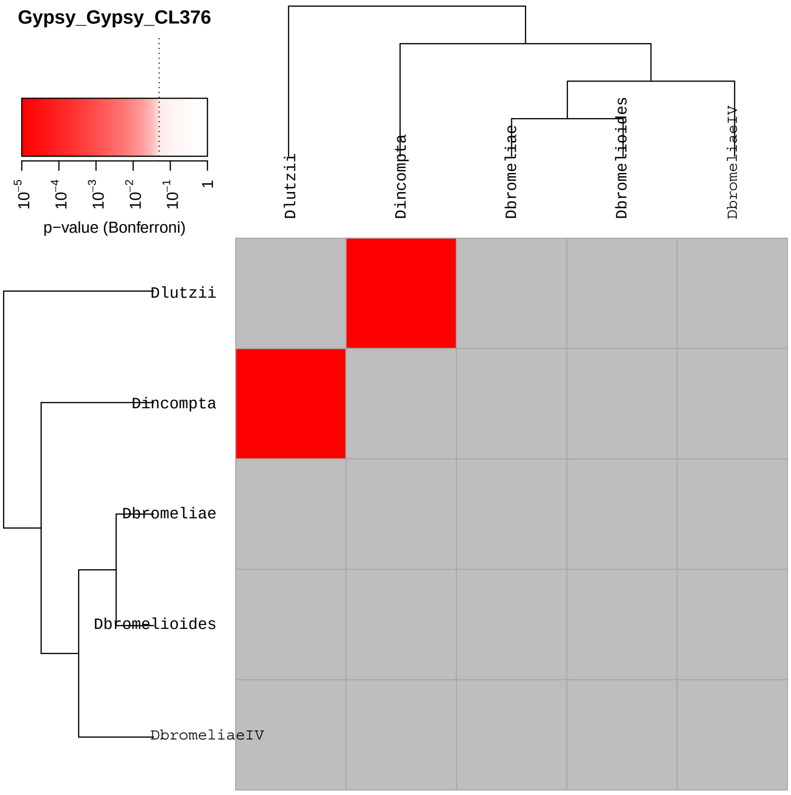
Gypsy_Gypsy_CL376
10^{-5} 10^{-4} 10^{-3} 10^{-2} 10^{-1} 1
p−value (Bonferroni)
Dlutzii
Dincompta
Dbromeliae
Dbromelioides
DbromeliaeIV

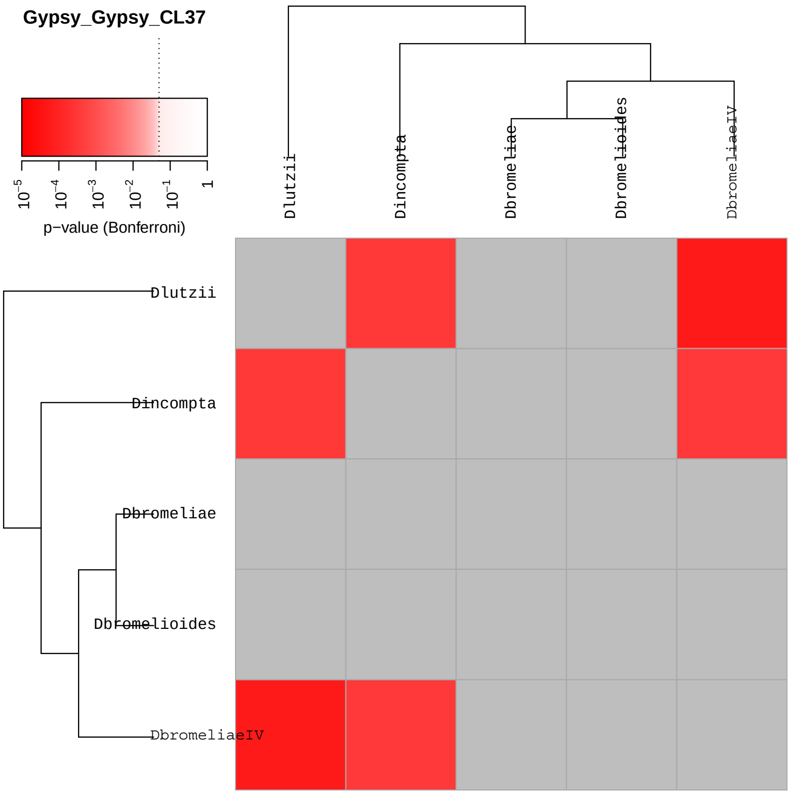
Gypsy_Gypsy_CL37
10^{-5} 10^{-4} 10^{-3} 10^{-2} 10^{-1} 1
p−value (Bonferroni)
Dlutzii
Dincompta
Dbromeliae
Dbromelioides
DbromeliaeIV

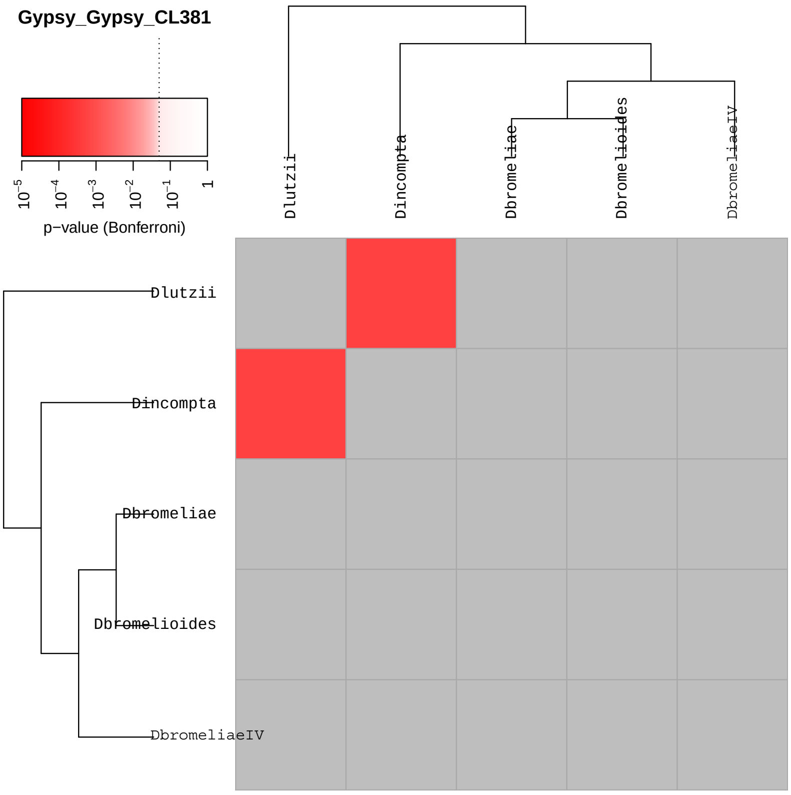
Gypsy_Gypsy_CL381
p−value (Bonferroni)
Dlutzii
Dincompta
Dbromeliae
Dbromelioides
DbromeliaeIV

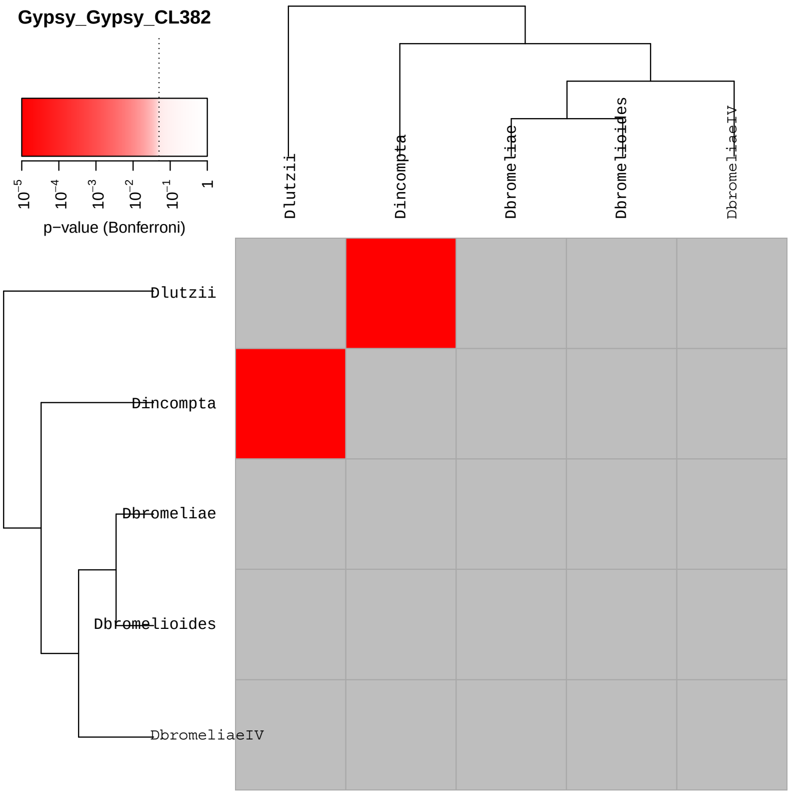
Gypsy_Gypsy_CL382
p−value (Bonferroni)
Dlutzii
Dincompta
Dbromeliae
Dbromelioides
DbromeliaeIV

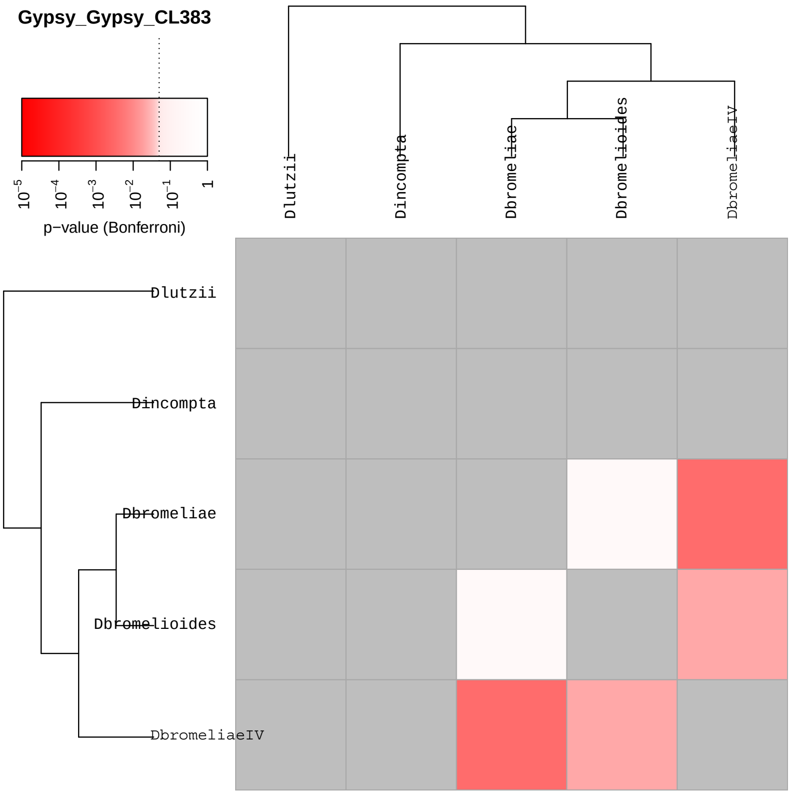
Gypsy_Gypsy_CL383
p−value (Bonferroni)
Dlutzii
Dincompta
Dbromeliae
Dbromelioides
DbromeliaeIV

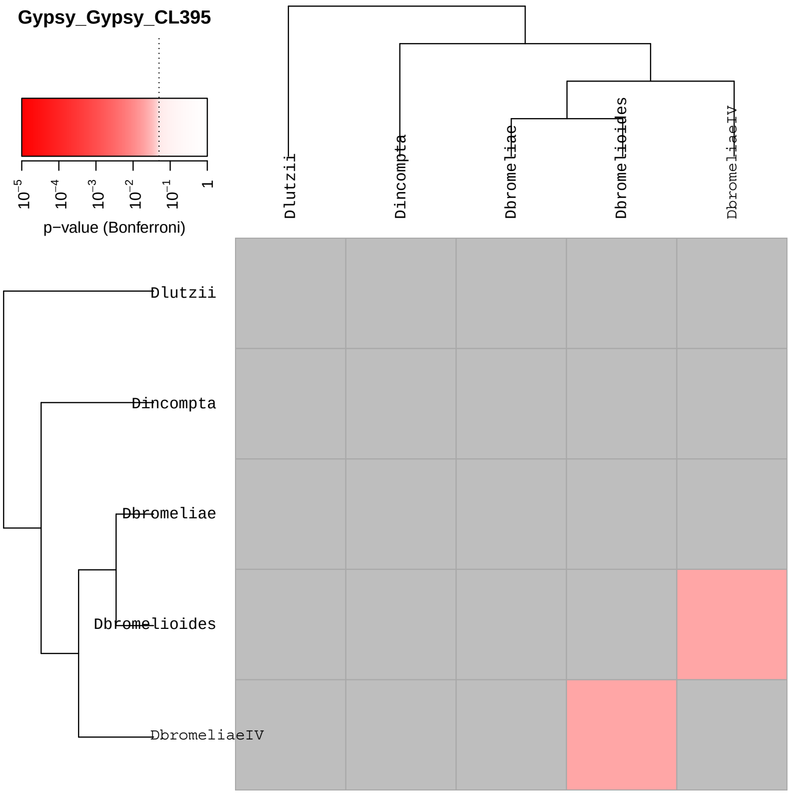
Gypsy_Gypsy_CL395
p−value (Bonferroni)
Dlutzii
Dincompta
Dbromeliae
Dbromelioides
DbromeliaeIV

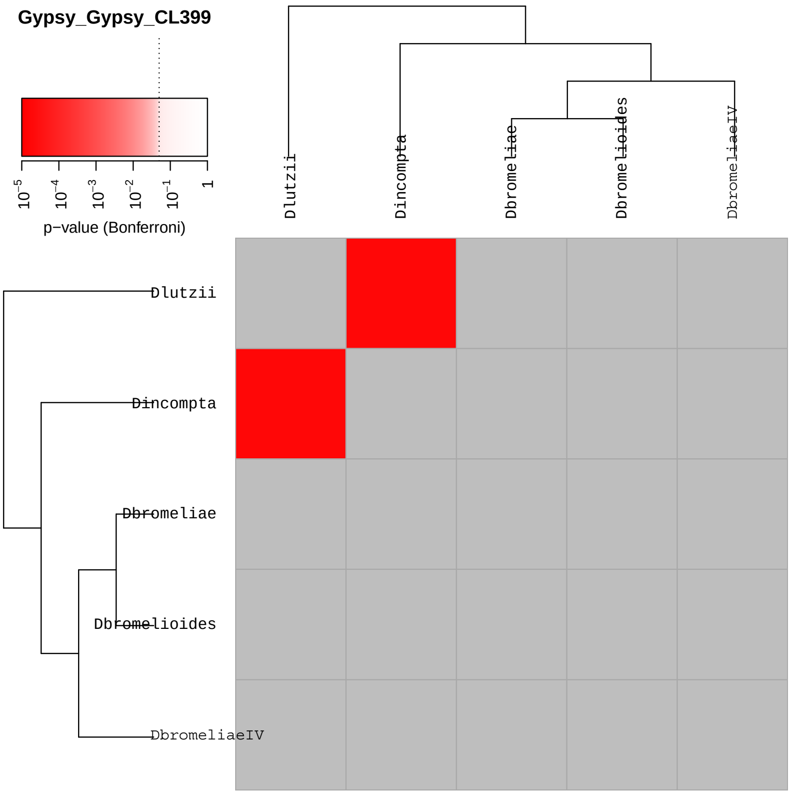
Gypsy_Gypsy_CL399
p−value (Bonferroni)
Dlutzii
Dincompta
Dbromeliae
Dbromelioides
DbromeliaeIV

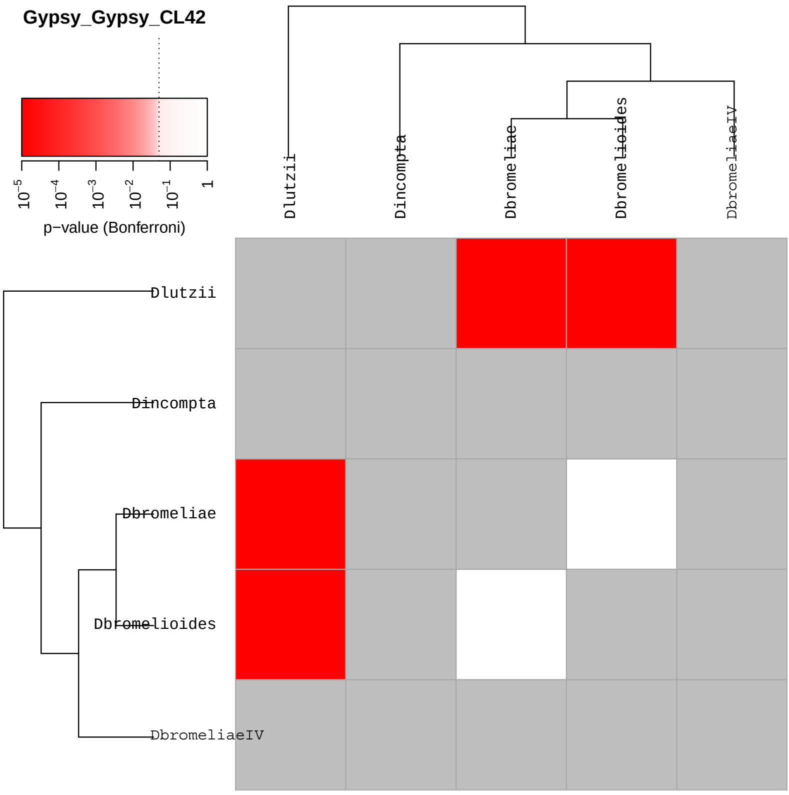
Gypsy_Gypsy_CL42
p−value (Bonferroni)
Dlutzii
Dincompta
Dbromeliae
Dbromelioides
DbromeliaeIV

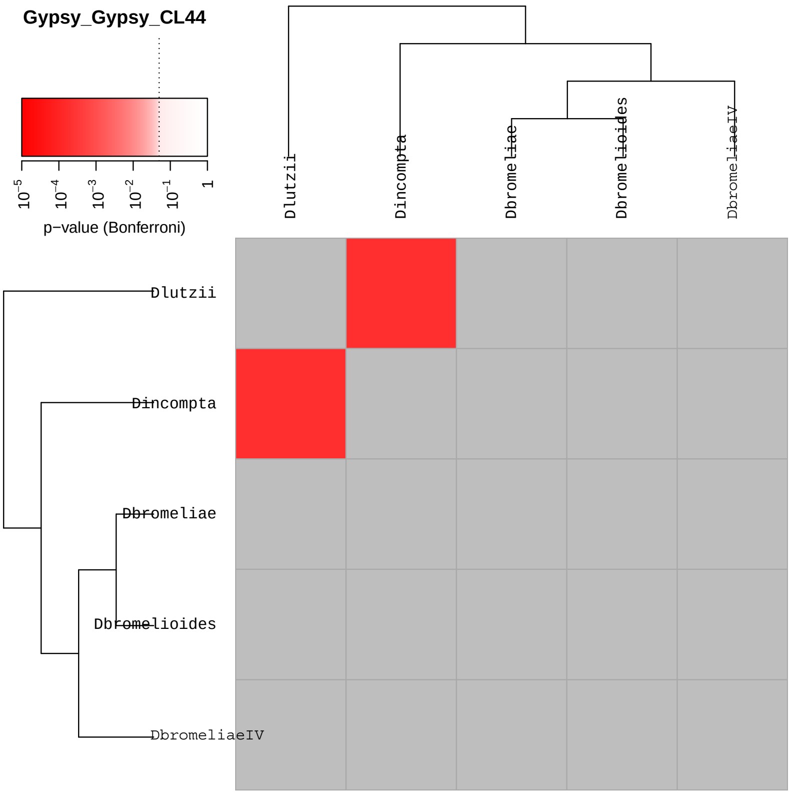
Gypsy_Gypsy_CL44
p−value (Bonferroni)
Dlutzii
Dincompta
Dbromeliae
Dbromelioides
DbromeliaeIV

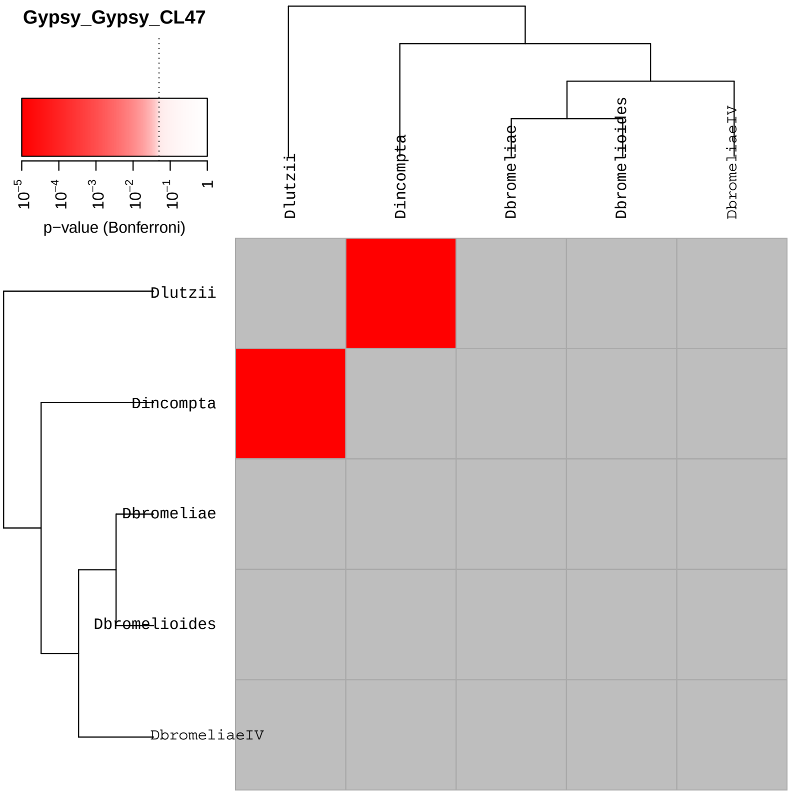
Gypsy_Gypsy_CL47
p−value (Bonferroni)
Dlutzii
Dincompta
Dbromeliae
Dbromelioides
DbromeliaeIV

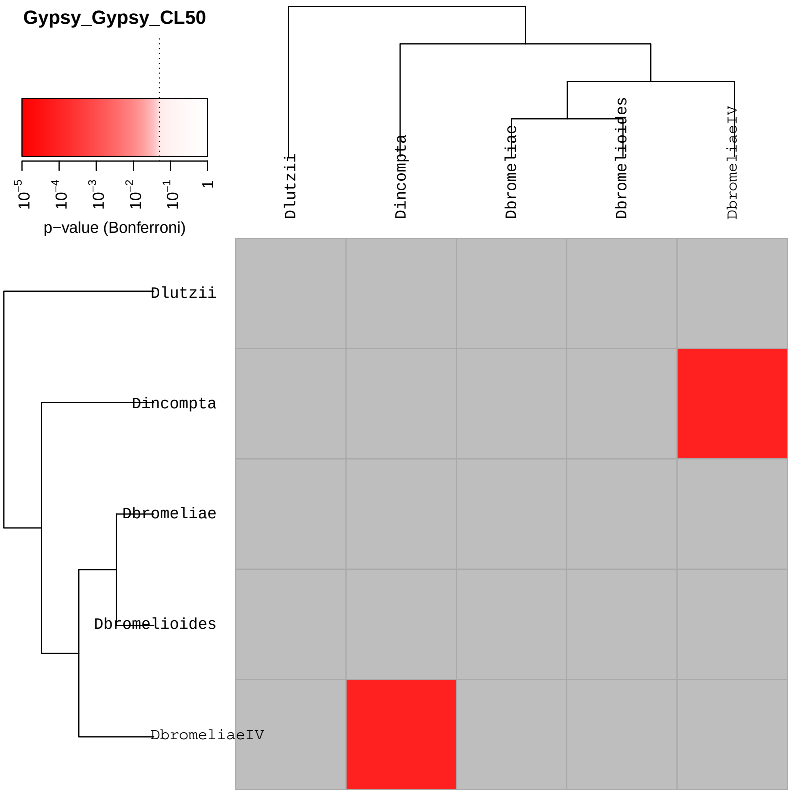
Gypsy_Gypsy_CL50
p−value (Bonferroni)
Dlutzii
Dincompta
Dbromeliae
Dbromelioides
DbromeliaeIV

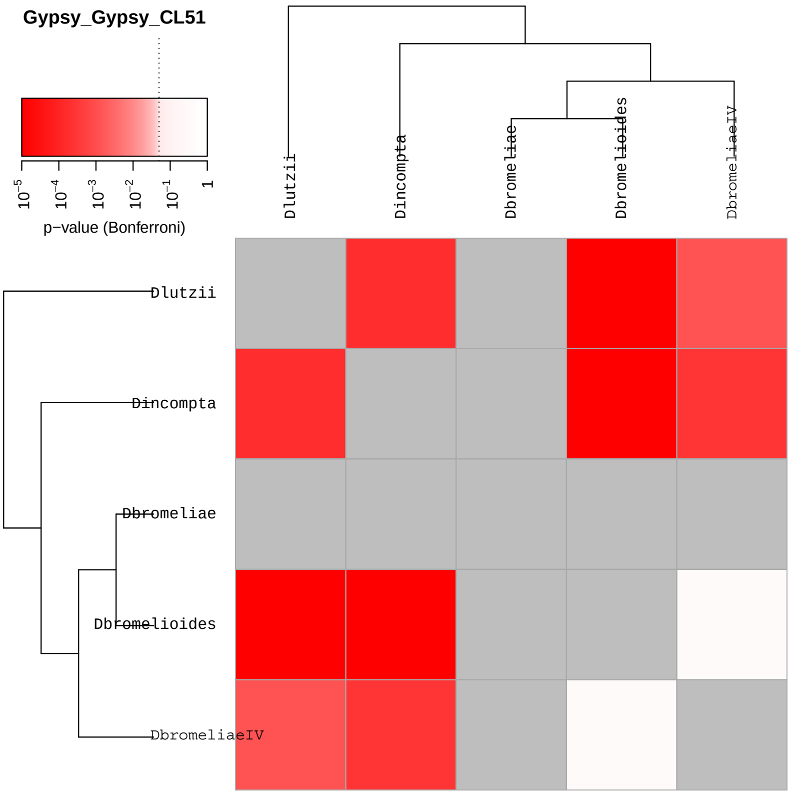
Gypsy_Gypsy_CL51
p−value (Bonferroni)
Dlutzii
Dincompta
Dbromeliae
Dbromelioides
DbromeliaeIV

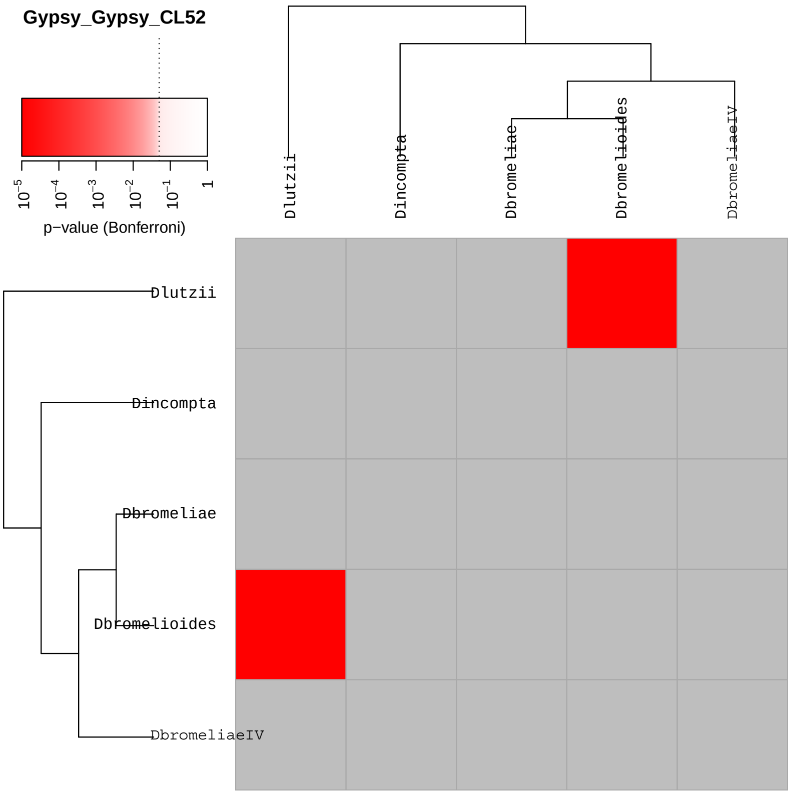
Gypsy_Gypsy_CL52
p−value (Bonferroni)
Dlutzii
Dincompta
Dbromeliae
Dbromelioides
DbromeliaeIV

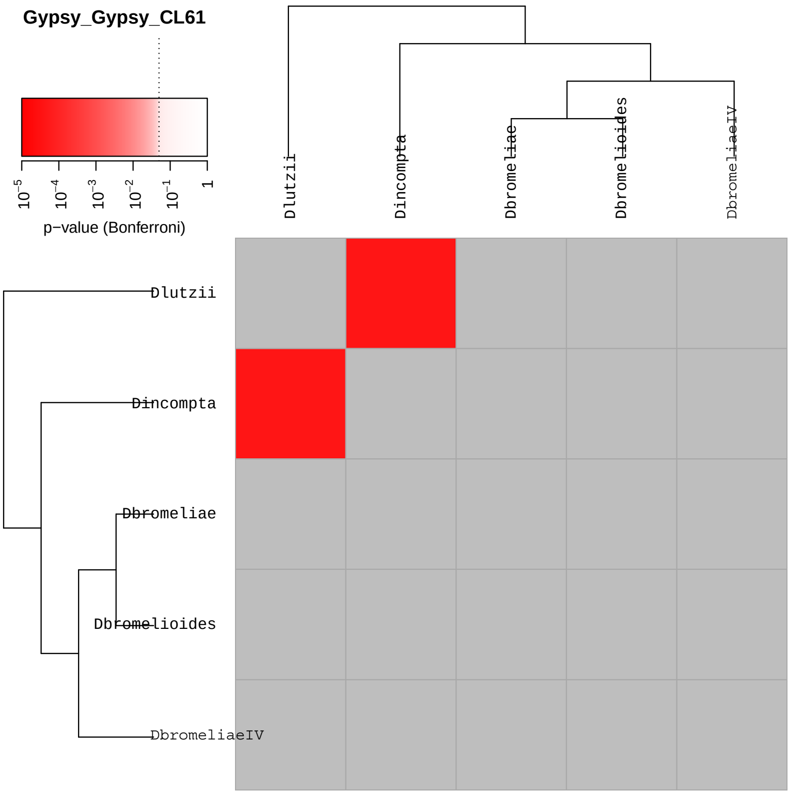
Gypsy_Gypsy_CL61
p−value (Bonferroni)
Dlutzii
Dincompta
Dbromeliae
Dbromelioides
DbromeliaeIV

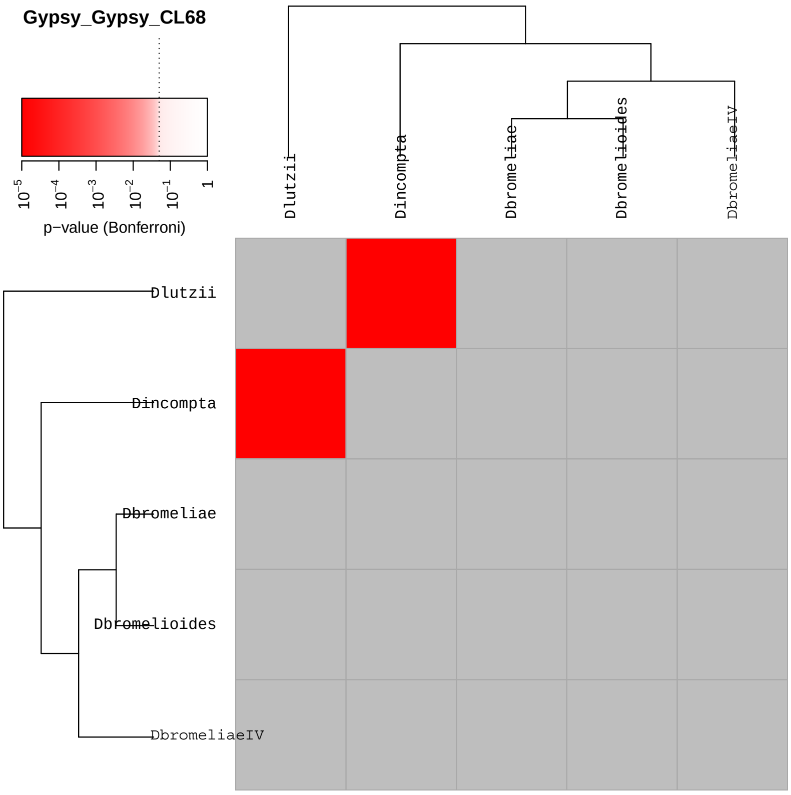
Gypsy_Gypsy_CL68
p−value (Bonferroni)
Dlutzii
Dincompta
Dbromeliae
Dbromelioides
DbromeliaeIV

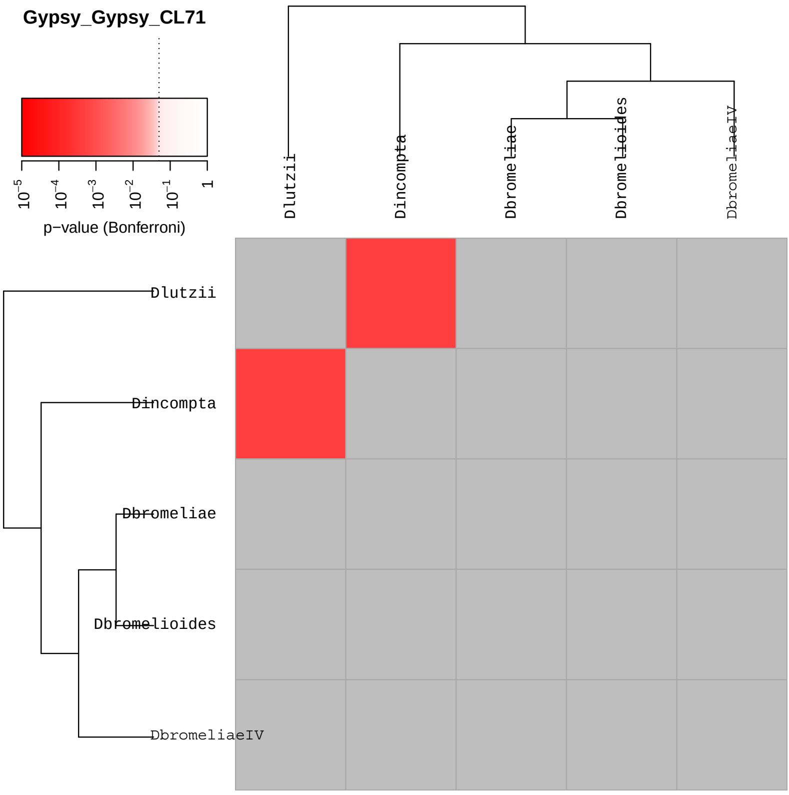
Gypsy_Gypsy_CL71
p−value (Bonferroni)
Dlutzii
Dincompta
Dbromeliae
Dbromelioides
DbromeliaeIV

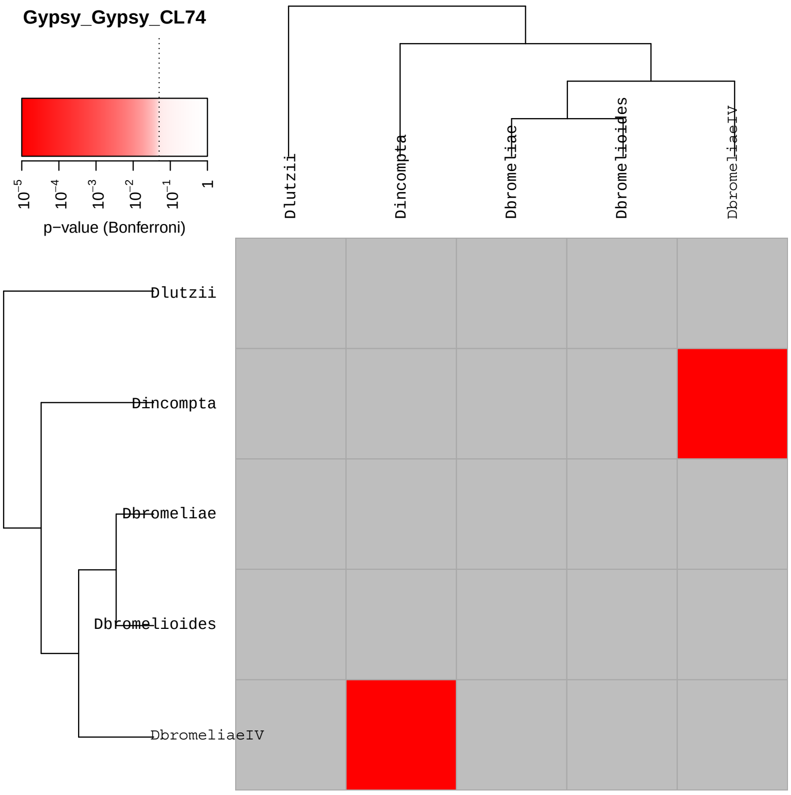
Gypsy_Gypsy_CL74
p−value (Bonferroni)
Dlutzii
Dincompta
Dbromeliae
Dbromelioides
DbromeliaeIV

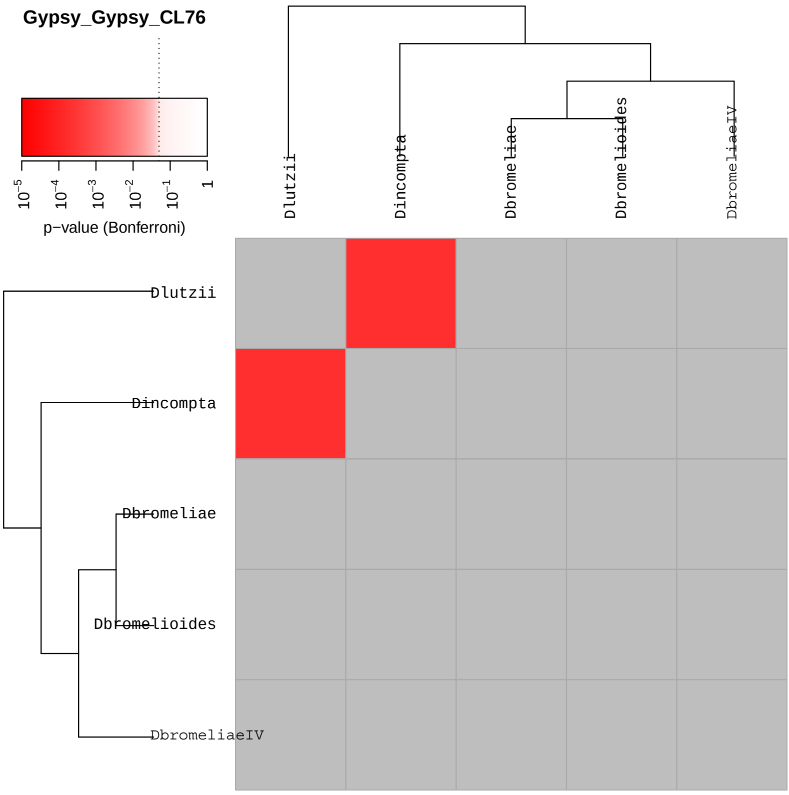
Gypsy_Gypsy_CL76
p−value (Bonferroni)
Dlutzii
Dincompta
Dbromeliae
Dbromelioides
DbromeliaeIV

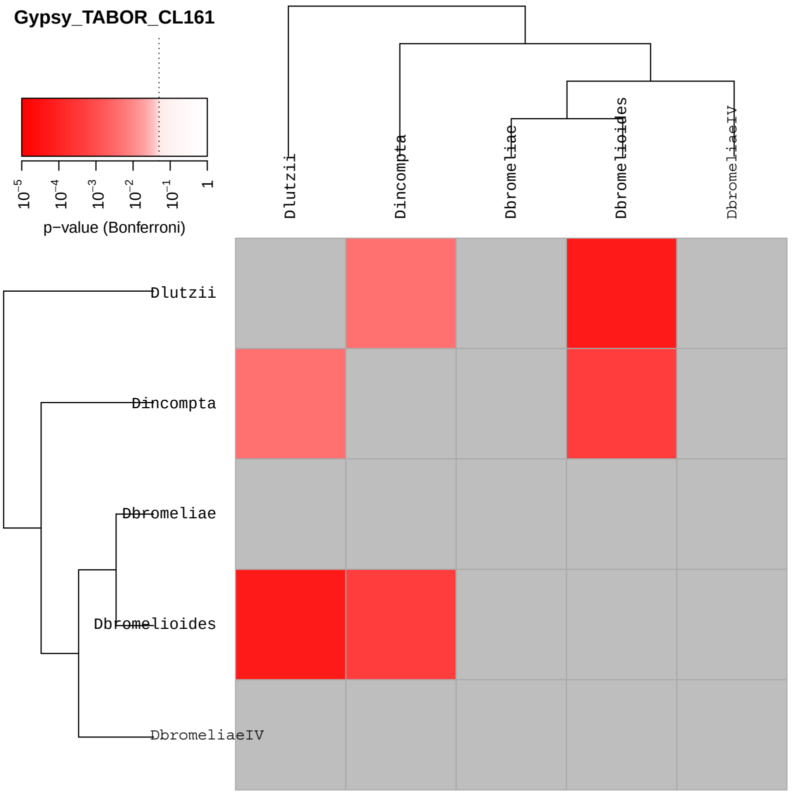
Gypsy_TABOR_CL161
10^{-5} 10^{-4} 10^{-3} 10^{-2} 10^{-1} 1
p−value (Bonferroni)
Dlutzii
Dincompta
Dbromeliae
Dbromelioides
DbromeliaeIV

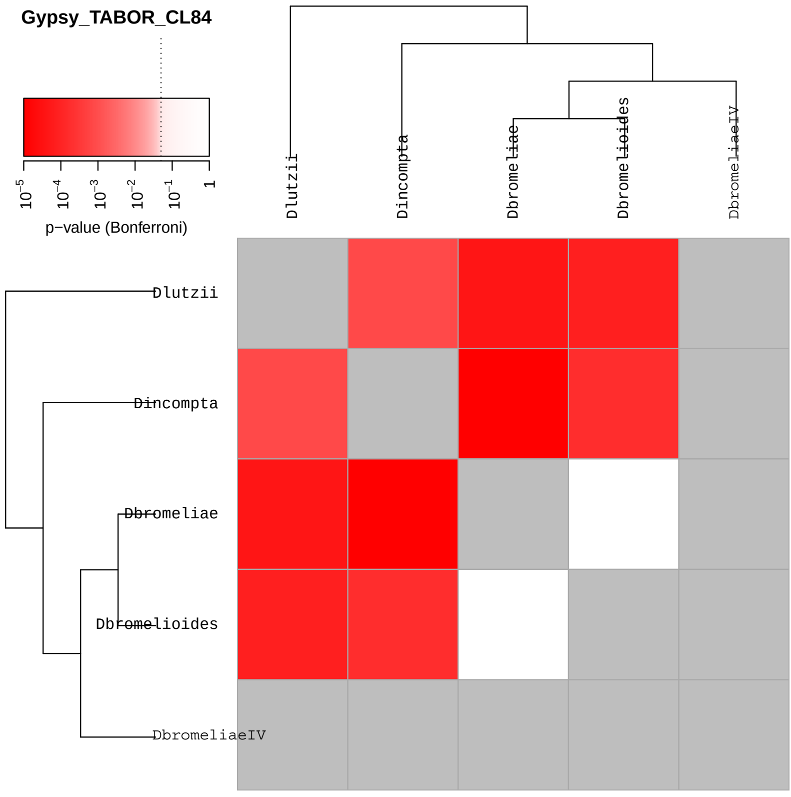
Gypsy_TABOR_CL84
10^{-5} 10^{-4} 10^{-3} 10^{-2} 10^{-1} 1
p−value (Bonferroni)
Dlutzii
Dincompta
Dbromeliae
Dbromelioides
DbromeliaeIV

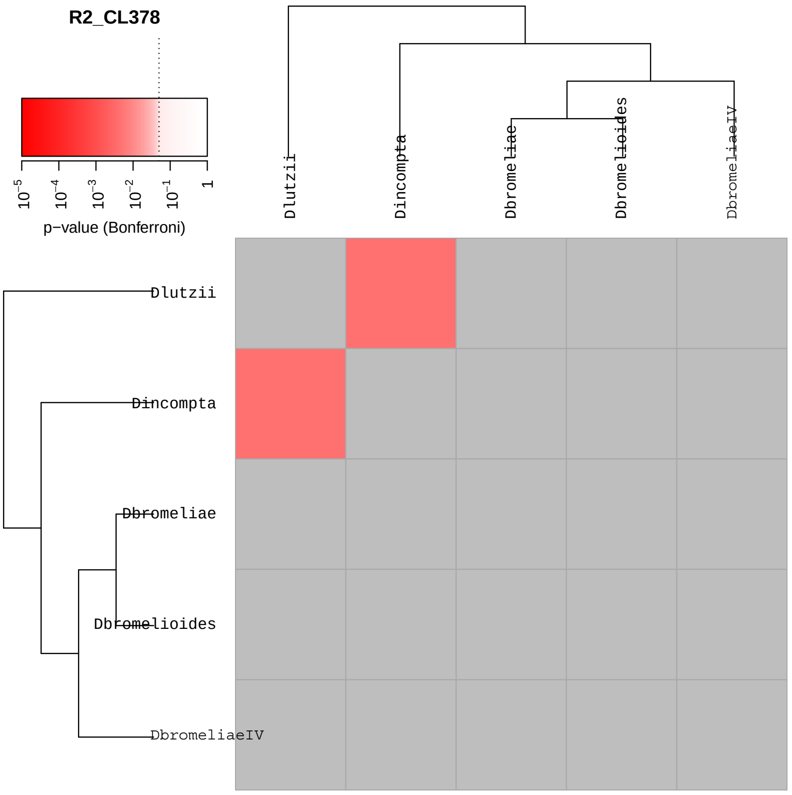
R2_CL378
10^{-5} 10^{-4} 10^{-3} 10^{-2} 10^{-1} 1
p−value (Bonferroni)
Dlutzii
Dincompta
Dbromeliae
Dbromelioides
DbromeliaeIV

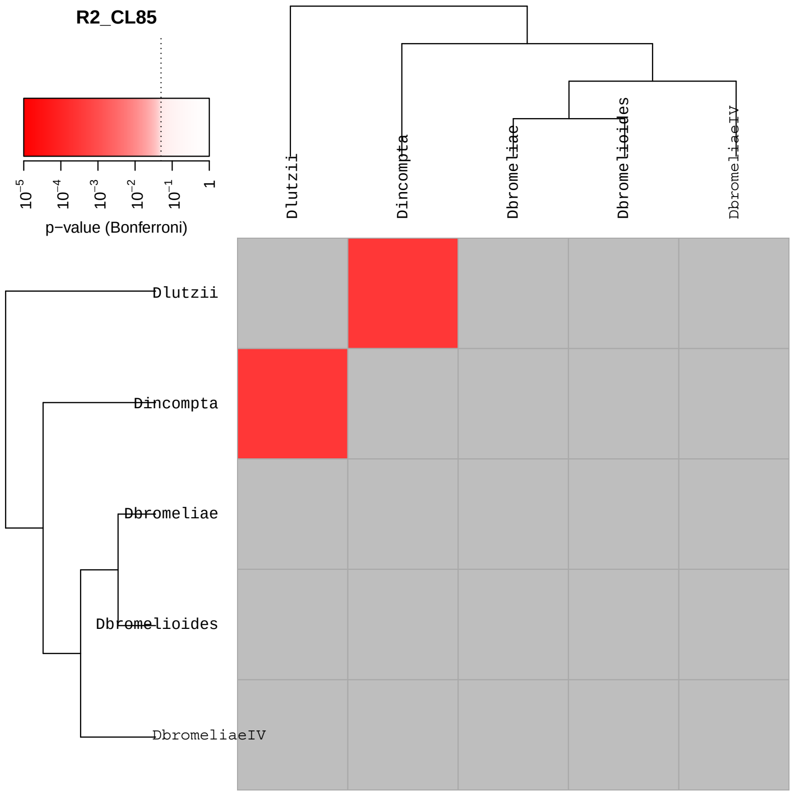
R2_CL85
10^{-5} 10^{-4} 10^{-3} 10^{-2} 10^{-1} 1
p−value (Bonferroni)
Dlutzii
Dincompta
Dbromeliae
Dbromelioides
DbromeliaeIV

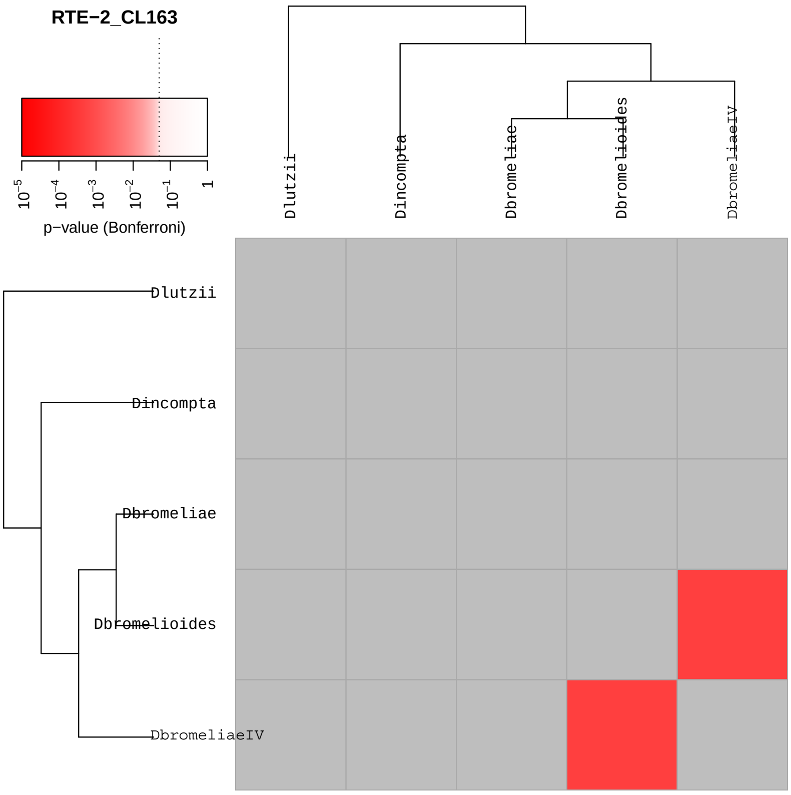
RTE−2_CL163
p−value (Bonferroni)
Dlutzii
Dincompta
Dbromeliae
Dbromelioides
DbromeliaeIV

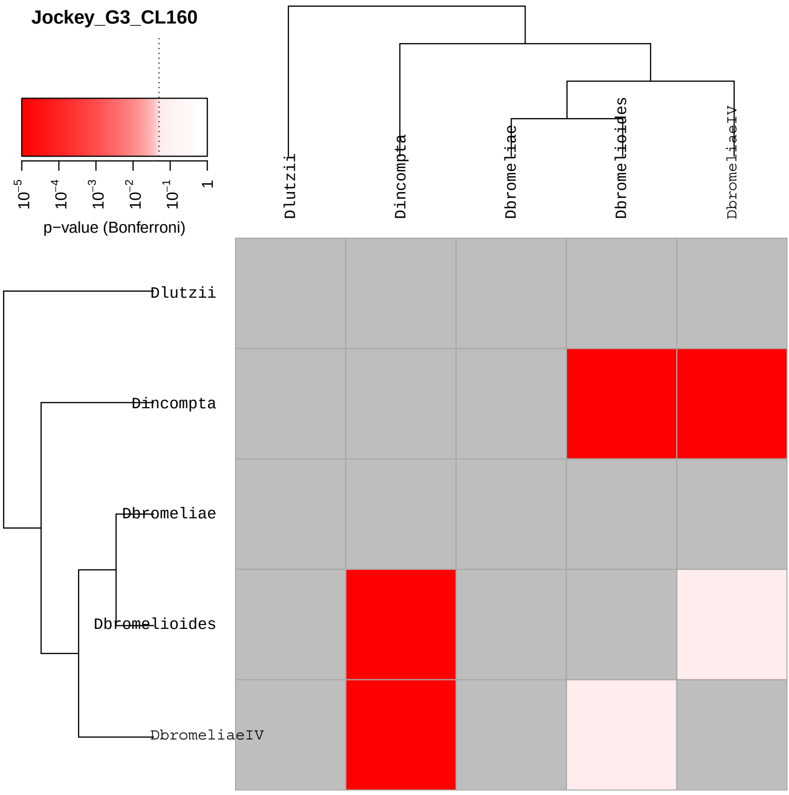
Jockey_G3_CL160
p−value (Bonferroni)
Dlutzii
Dincompta
Dbromeliae
Dbromelioides
DbromeliaeIV

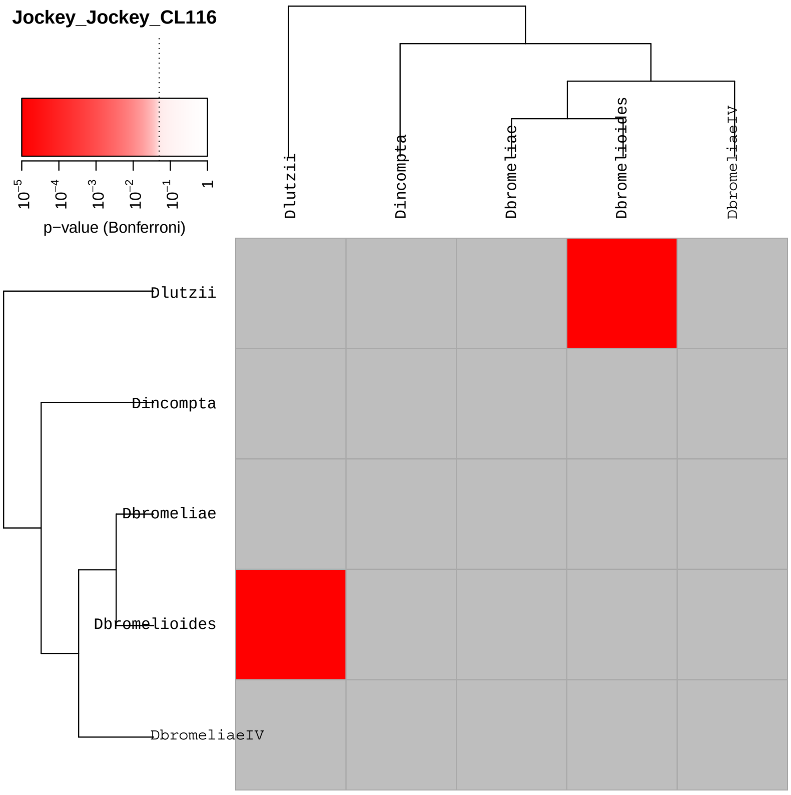
Jockey_Jockey_CL116
p−value (Bonferroni)
Dlutzii
Dincompta
Dbromeliae
Dbromelioides
DbromeliaeIV

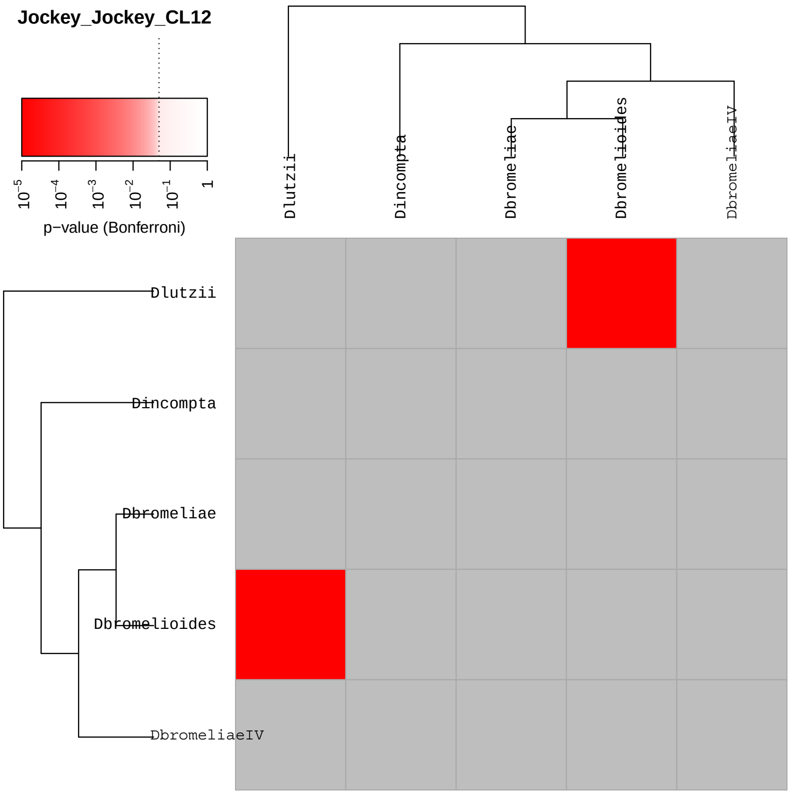
Jockey_Jockey_CL12
p−value (Bonferroni)
Dlutzii
Dincompta
Dbromeliae
Dbromelioides
DbromeliaeIV

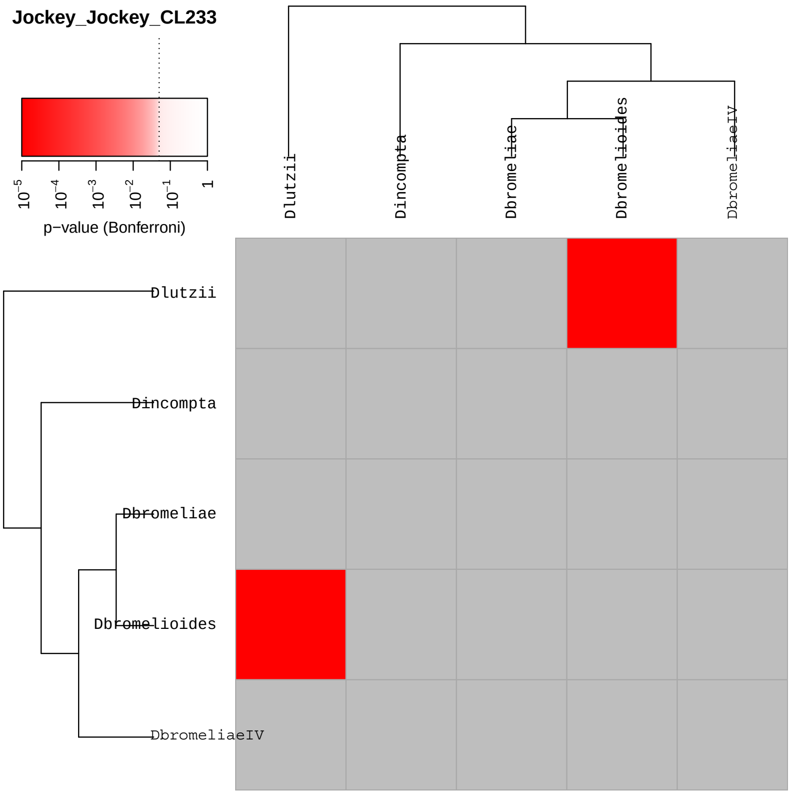
Jockey_Jockey_CL233
10^-5
10^-4
10^-3
10^-2
10^-1
1
p−value (Bonferroni)
Dlutzii
Dincompta
Dbromeliae
Dbromelioides
DbromeliaeIV

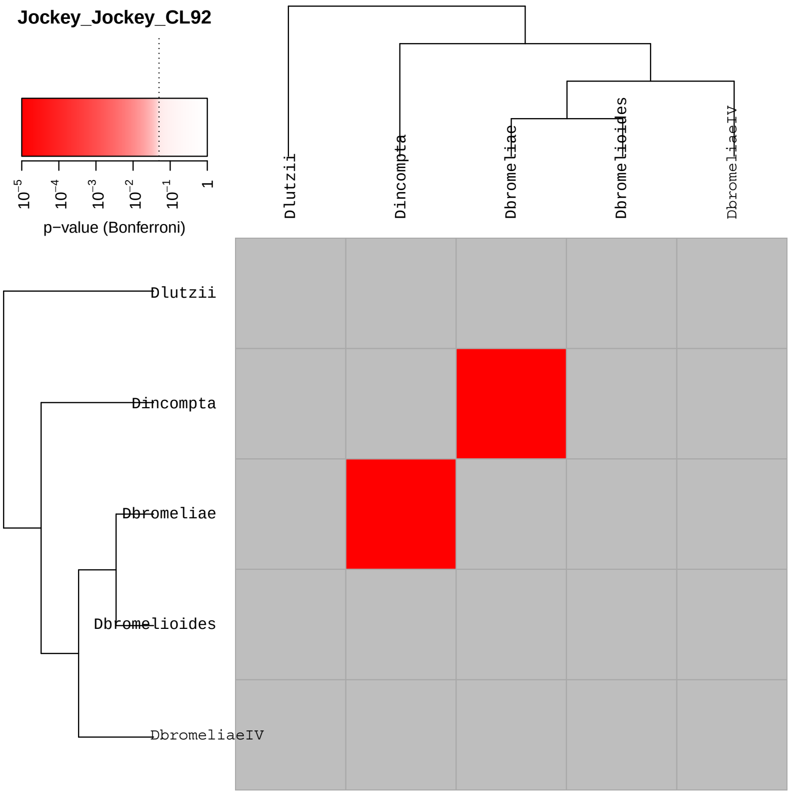
Jockey_Jockey_CL92
10^-5
10^-4
10^-3
10^-2
10^-1
1
p−value (Bonferroni)
Dlutzii
Dincompta
Dbromeliae
Dbromelioides
DbromeliaeIV

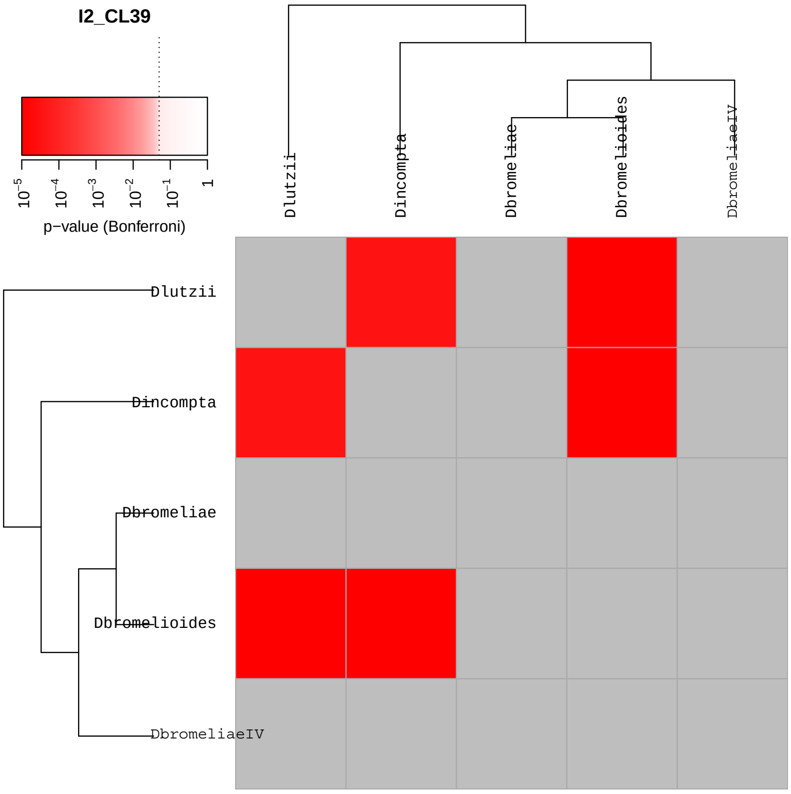
I2_CL39
10^-5
10^-4
10^-3
10^-2
10^-1
1
p−value (Bonferroni)
Dlutzii
Dincompta
Dbromeliae
Dbromelioides
DbromeliaeIV

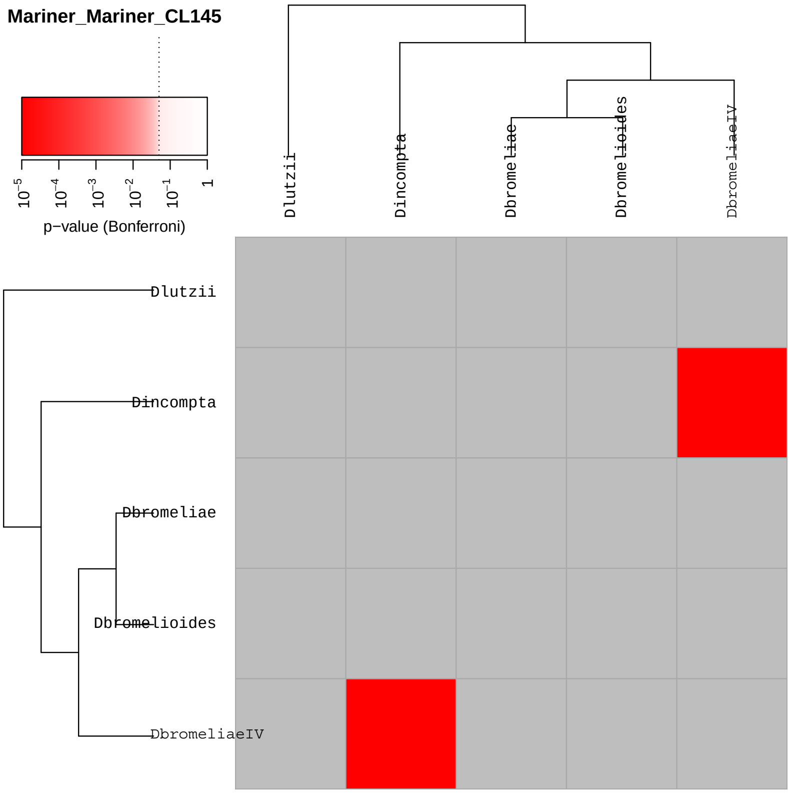
Mariner_Mariner_CL145
10^-5
10^-4
10^-3
10^-2
10^-1
1
p−value (Bonferroni)
Dlutzii
Dincompta
Dbromeliae
Dbromelioides
DbromeliaeIV

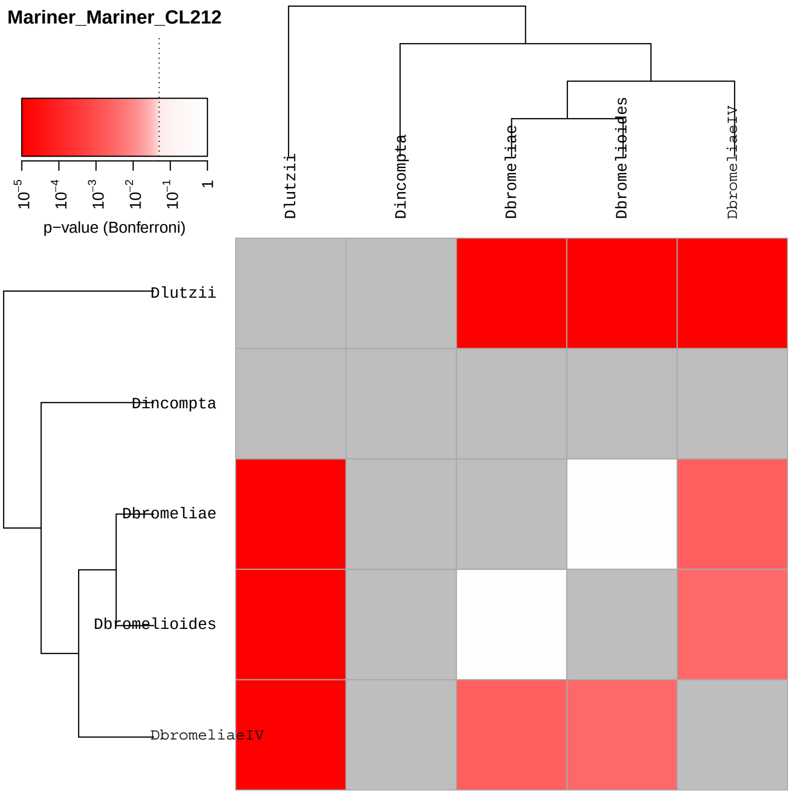
Mariner_Mariner_CL212
p−value (Bonferroni)
Dlutzii
Dincompta
Dbromeliae
Dbromelioides
DbromeliaeIV

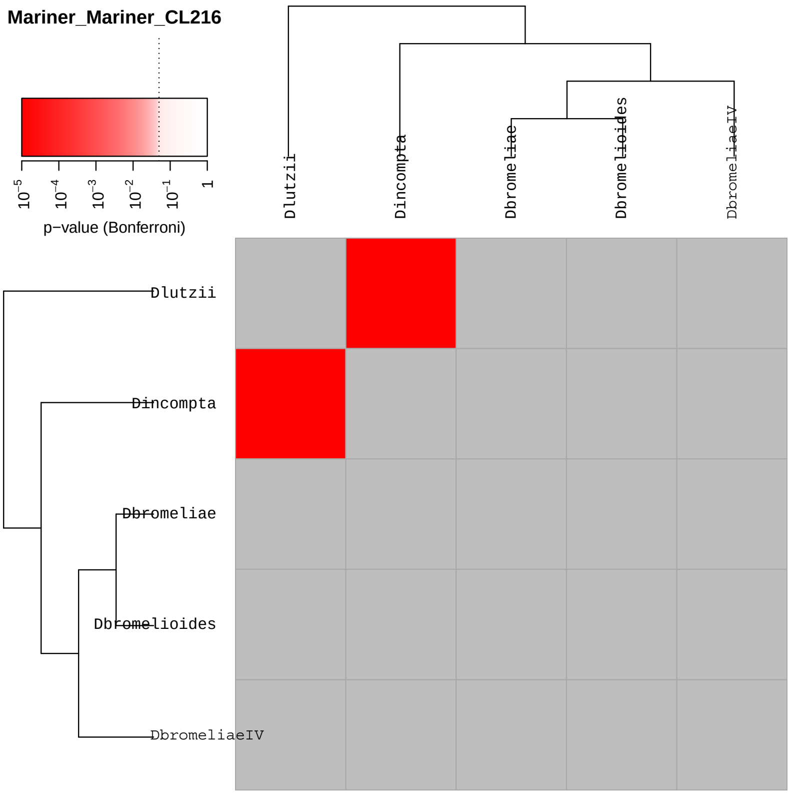
Mariner_Mariner_CL216
p−value (Bonferroni)
Dlutzii
Dincompta
Dbromeliae
Dbromelioides
DbromeliaeIV

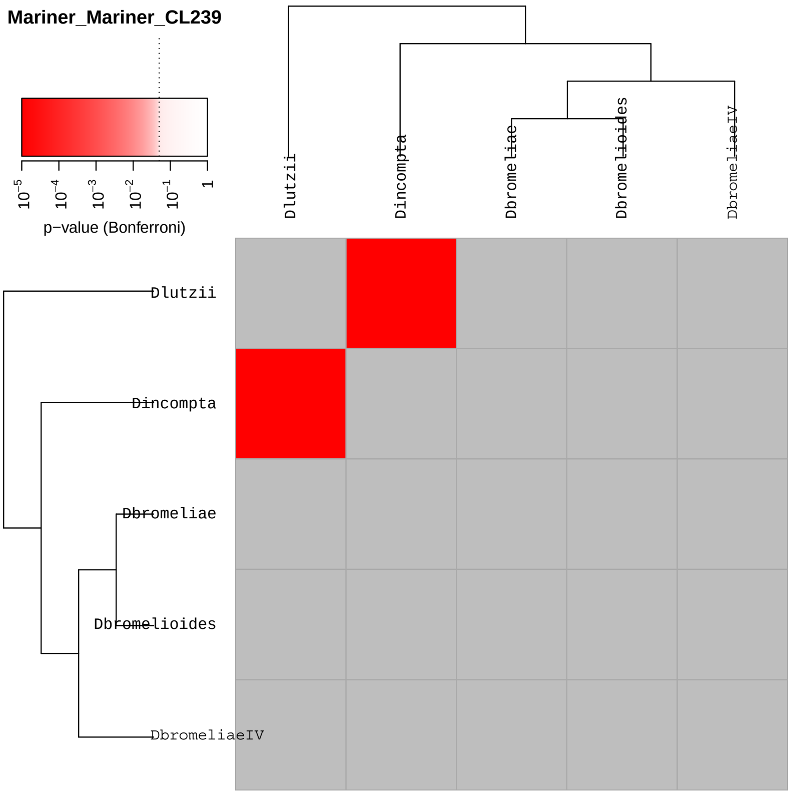
Mariner_Mariner_CL239
p−value (Bonferroni)
Dlutzii
Dincompta
Dbromeliae
Dbromelioides
DbromeliaeIV

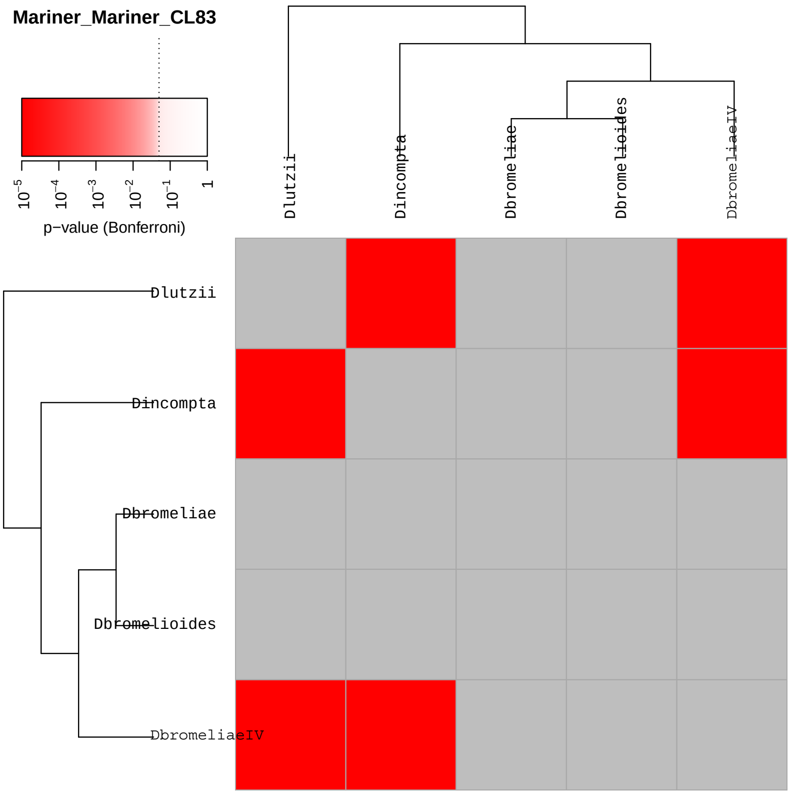
Mariner_Mariner_CL83
p−value (Bonferroni)
Dlutzii
Dincompta
Dbromeliae
Dbromelioides
DbromeliaeIV

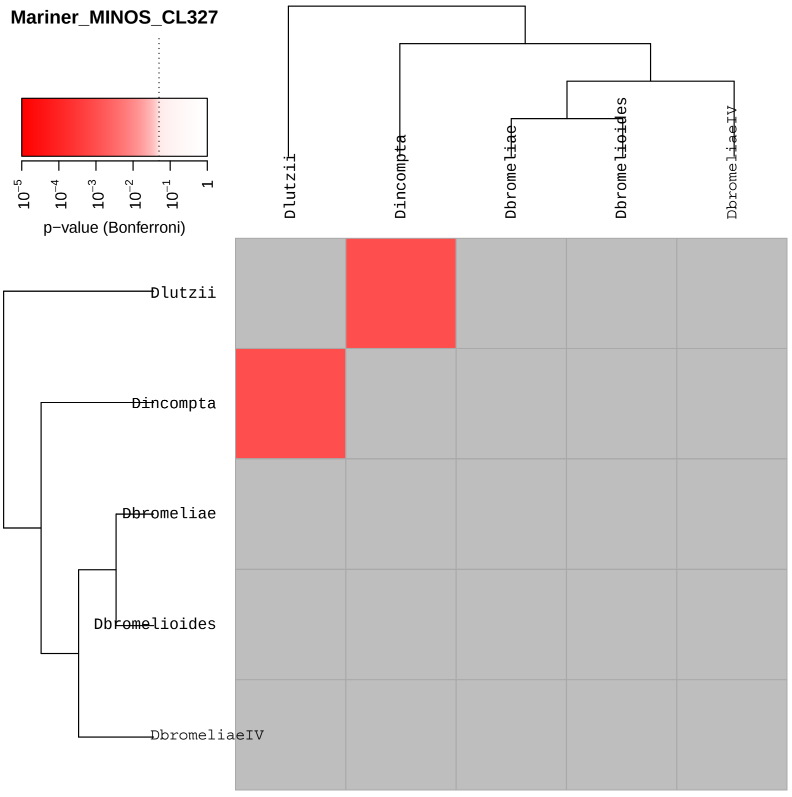
Mariner_MINOS_CL327
10^-5 10^-4 10^-3 10^-2 10^-1 1
p−value (Bonferroni)
Dlutzii
Dincompta
Dbromeliae
Dbromelioides
DbromeliaeIV

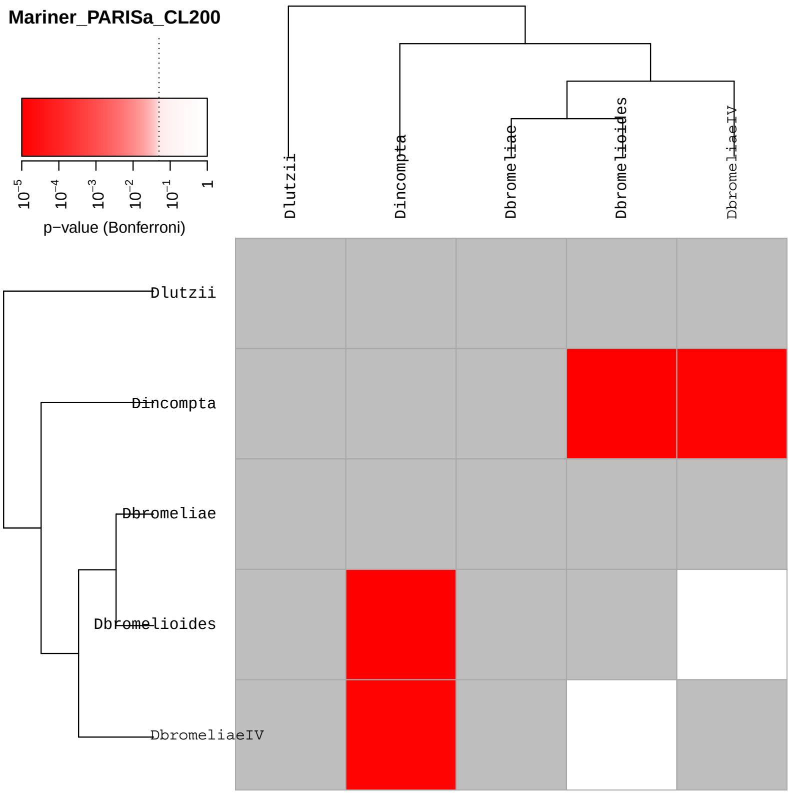
Mariner_PARISa_CL200
10^-5 10^-4 10^-3 10^-2 10^-1 1
p−value (Bonferroni)
Dlutzii
Dincompta
Dbromeliae
Dbromelioides
DbromeliaeIV

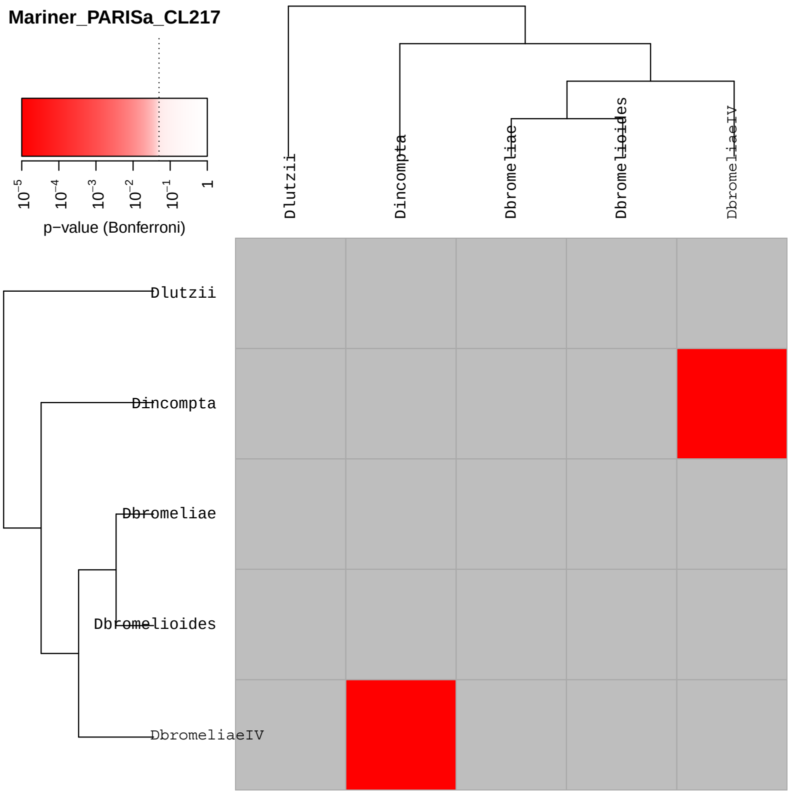
Mariner_PARISa_CL217
10^-5 10^-4 10^-3 10^-2 10^-1 1
p−value (Bonferroni)
Dlutzii
Dincompta
Dbromeliae
Dbromelioides
DbromeliaeIV

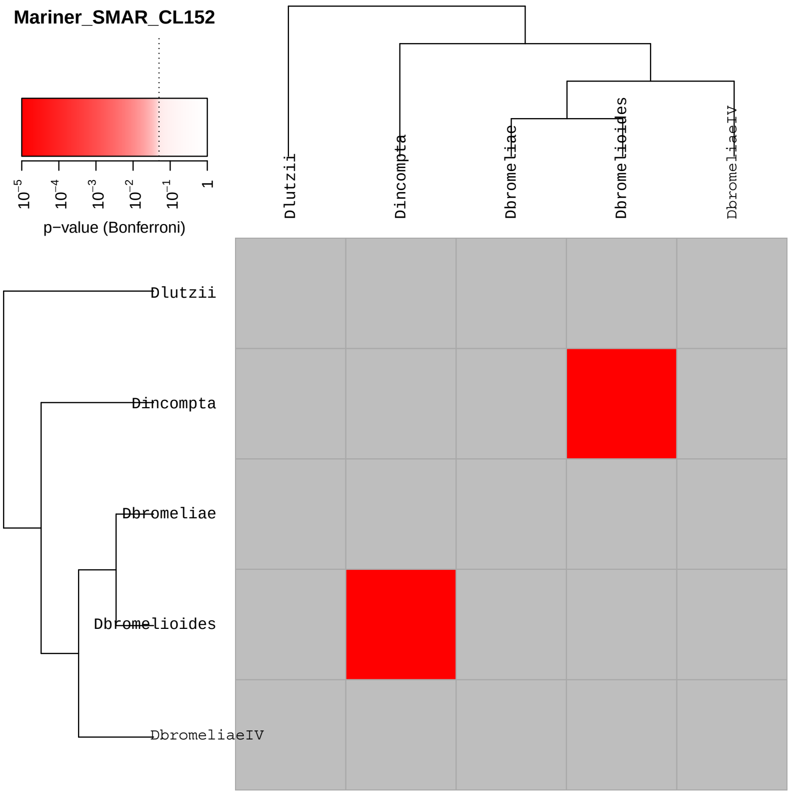
Mariner_SMAR_CL152
10^-5 10^-4 10^-3 10^-2 10^-1 1
p−value (Bonferroni)
Dlutzii
Dincompta
Dbromeliae
Dbromelioides
DbromeliaeIV

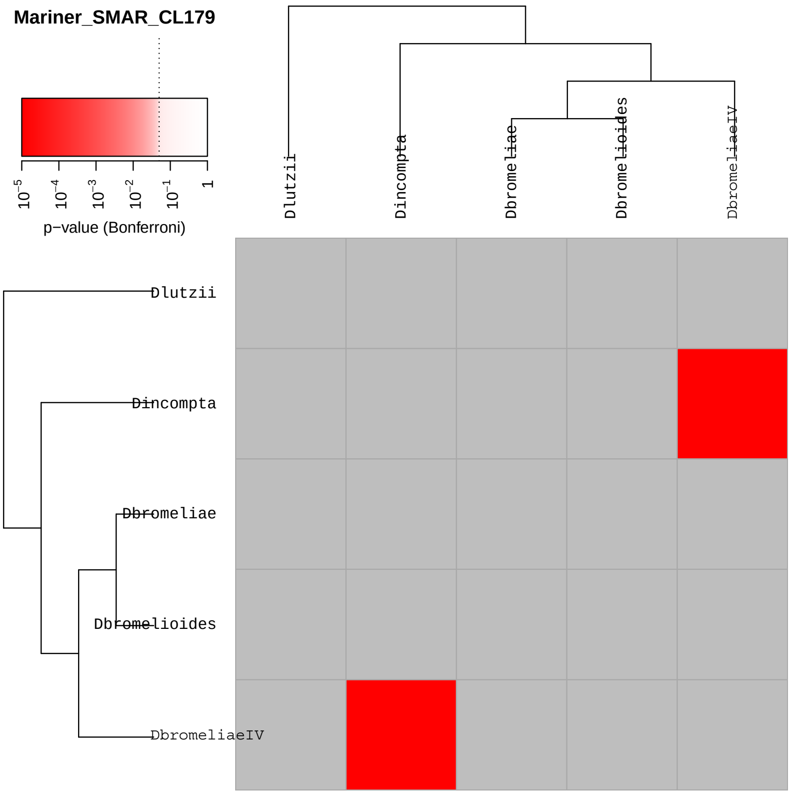
Mariner_SMAR_CL179
p−value (Bonferroni)
Dlutzii
Dincompta
Dbromeliae
Dbromelioides
DbromeliaeIV

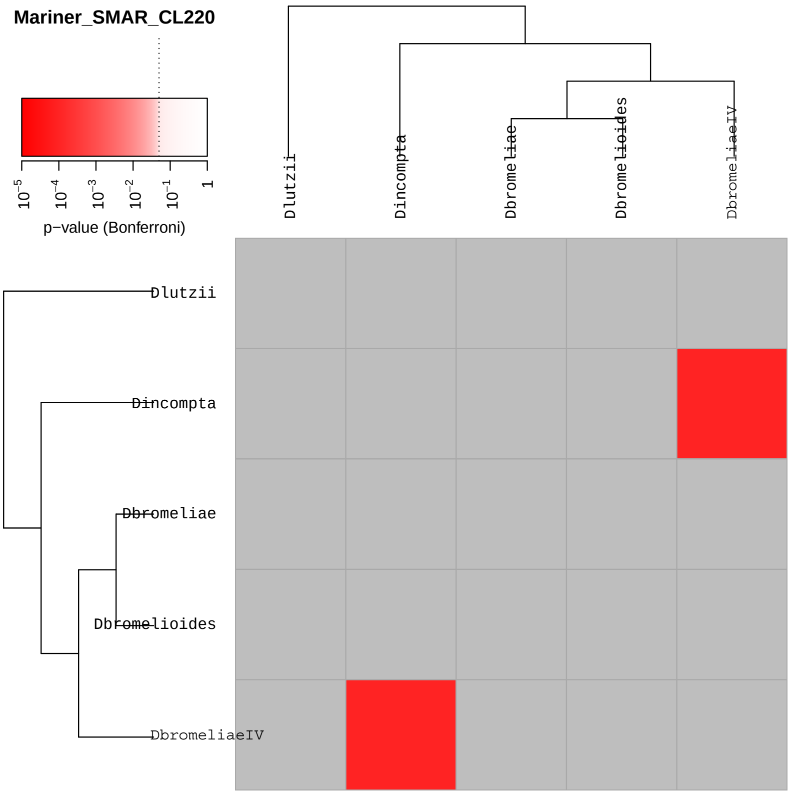
Mariner_SMAR_CL220
p−value (Bonferroni)
Dlutzii
Dincompta
Dbromeliae
Dbromelioides
DbromeliaeIV

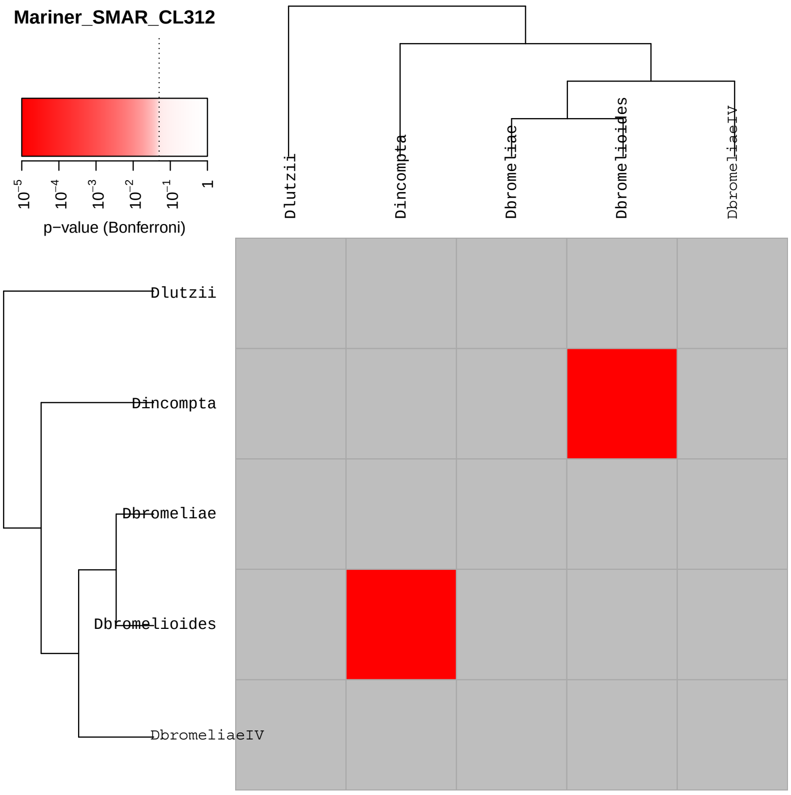
Mariner_SMAR_CL312
p−value (Bonferroni)
Dlutzii
Dincompta
Dbromeliae
Dbromelioides
DbromeliaeIV

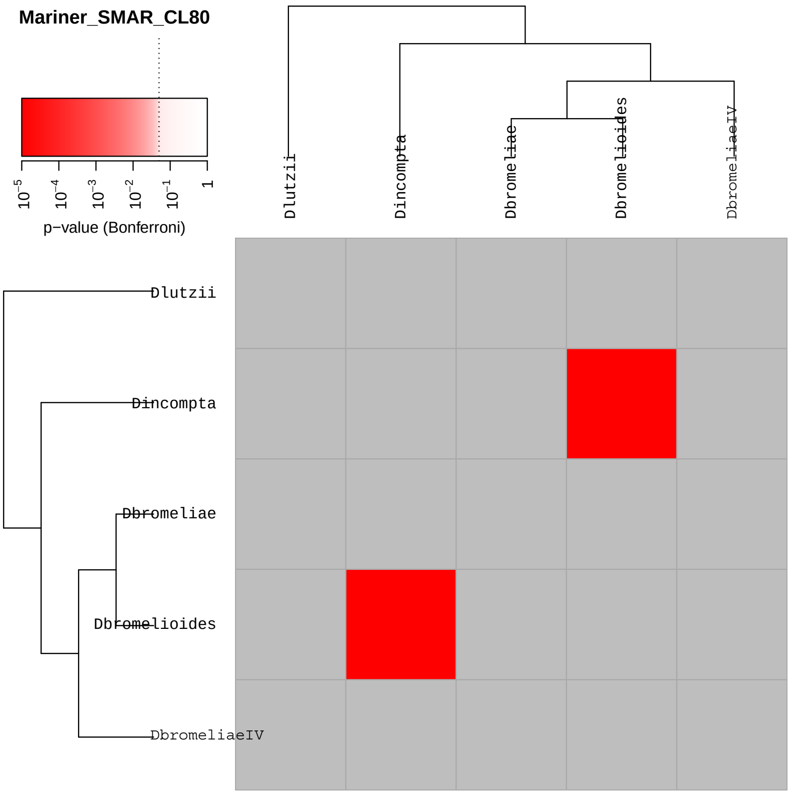
Mariner_SMAR_CL80
p−value (Bonferroni)
Dlutzii
Dincompta
Dbromeliae
Dbromelioides
DbromeliaeIV

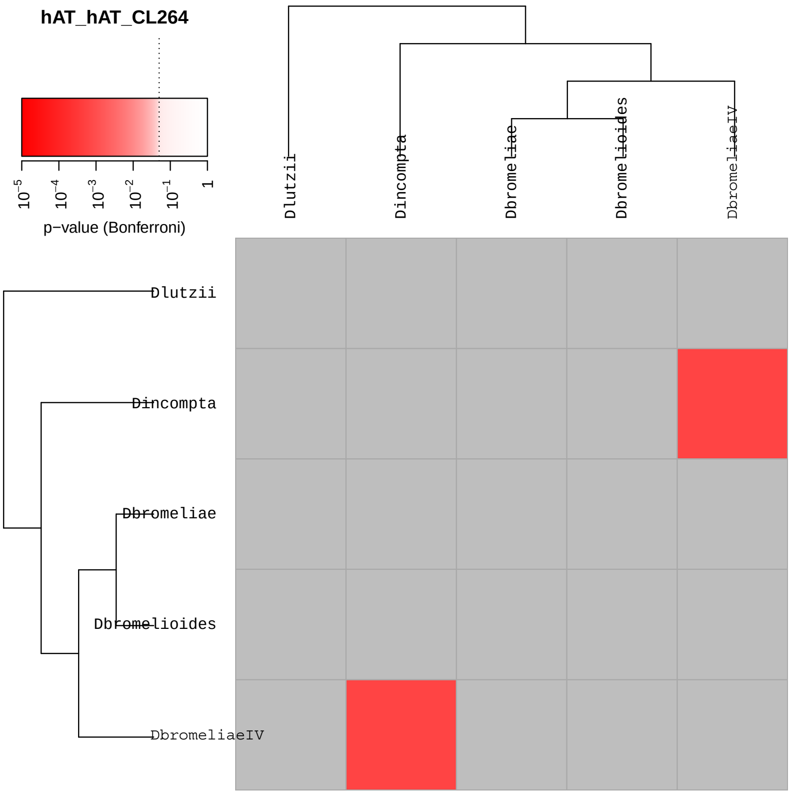
hAT_hAT_CL264
p−value (Bonferroni)
Dlutzii
Dincompta
Dbromeliae
Dbromelioides
DbromeliaeIV

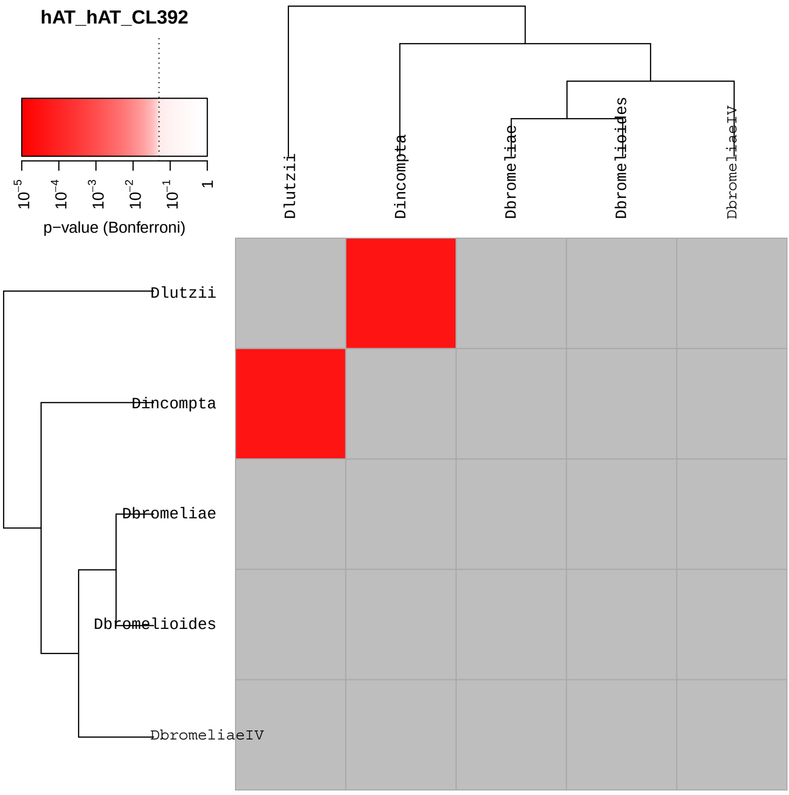
hAT_hAT_CL392
p−value (Bonferroni)
Dlutzii
Dincompta
Dbromeliae
Dbromelioides
DbromeliaeIV

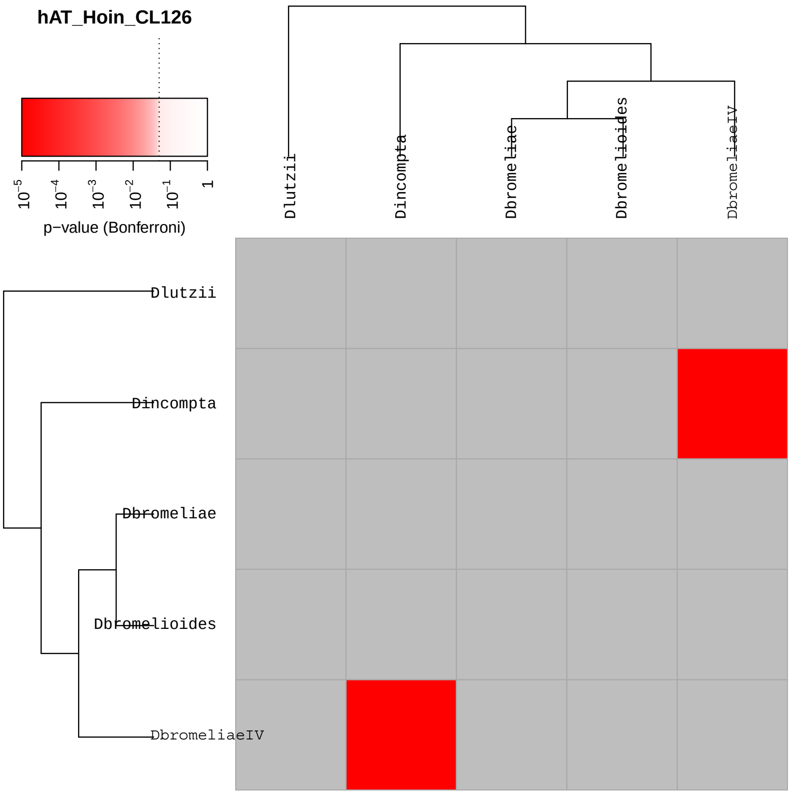
hAT_Hoin_CL126
p−value (Bonferroni)
Dlutzii
Dincompta
Dbromeliae
Dbromelioides
DbromeliaeIV

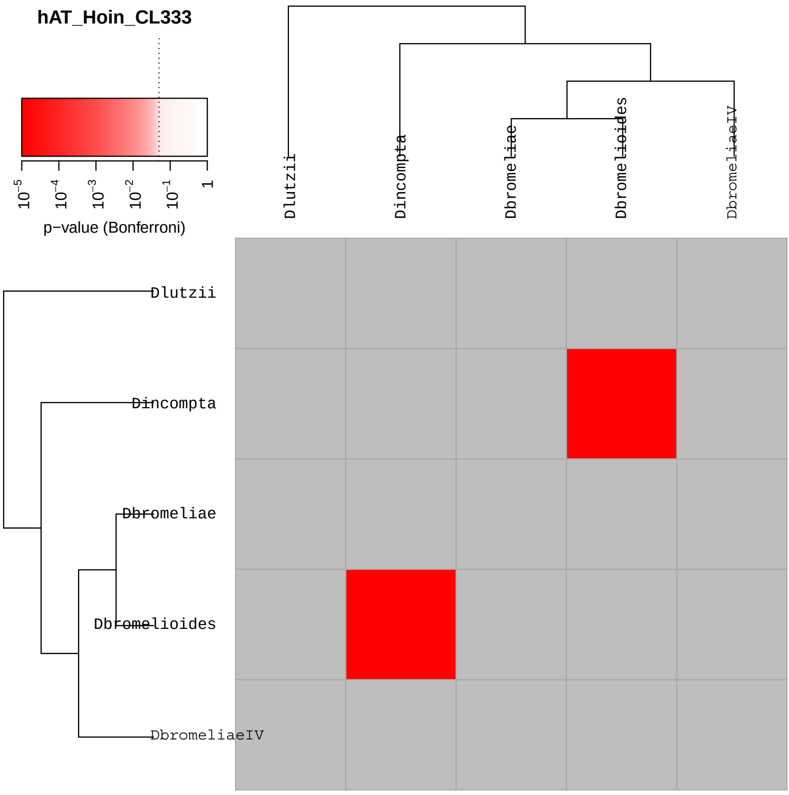
hAT_Hoin_CL333
p−value (Bonferroni)
Dlutzii
Dincompta
Dbromeliae
Dbromelioides
DbromeliaeIV

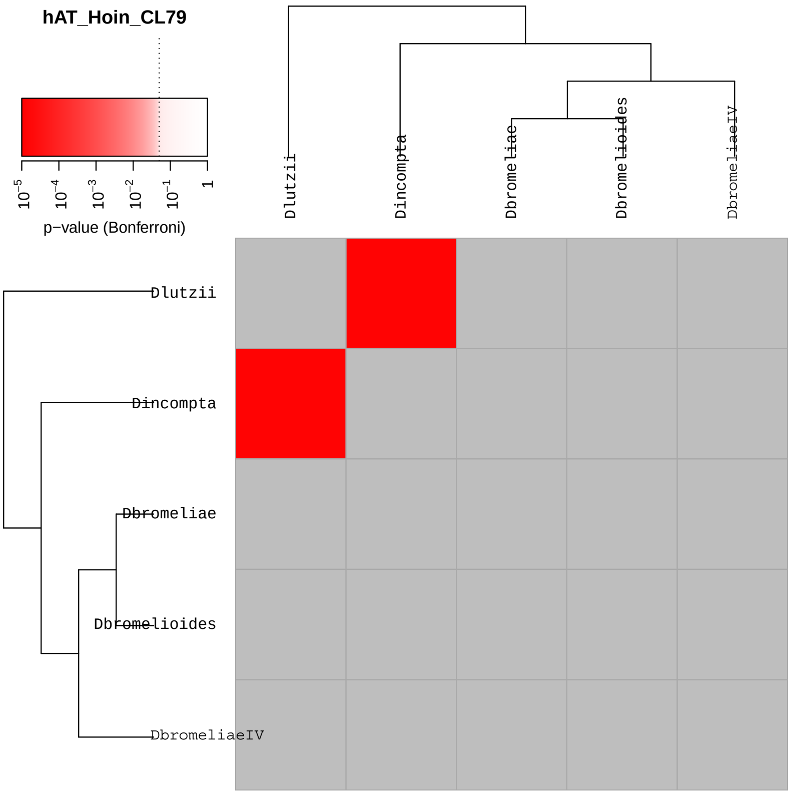
hAT_Hoin_CL79
10−5 10−4 10−3 10−2 10−1 1
p−value (Bonferroni)
Dlutzii
Dincompta
Dbromeliae
Dbromelioides
DbromeliaeIV

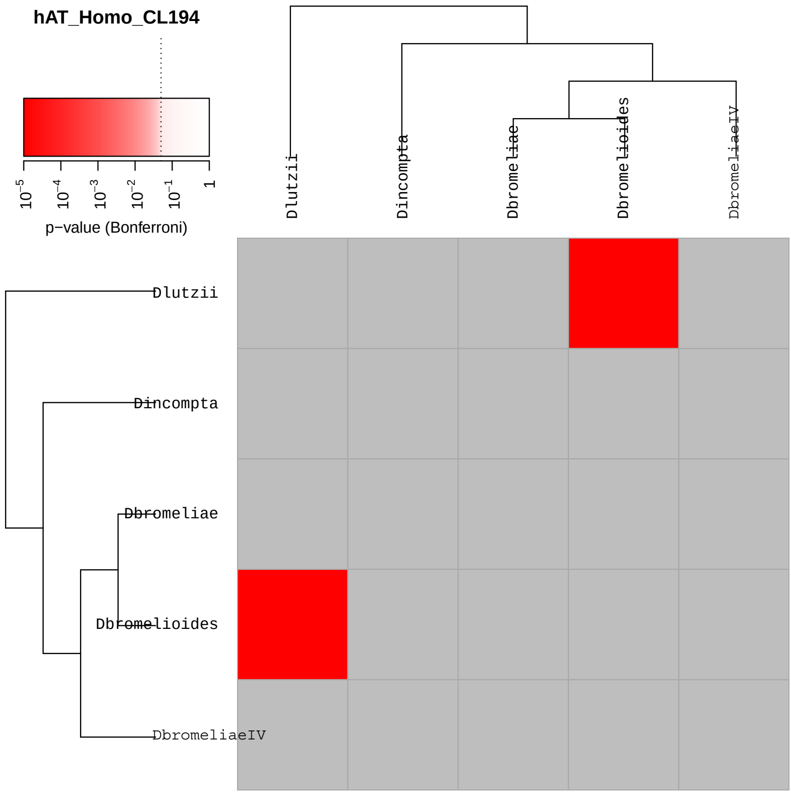
hAT_Homo_CL194
10−5 10−4 10−3 10−2 10−1 1
p−value (Bonferroni)
Dlutzii
Dincompta
Dbromeliae
Dbromelioides
DbromeliaeIV

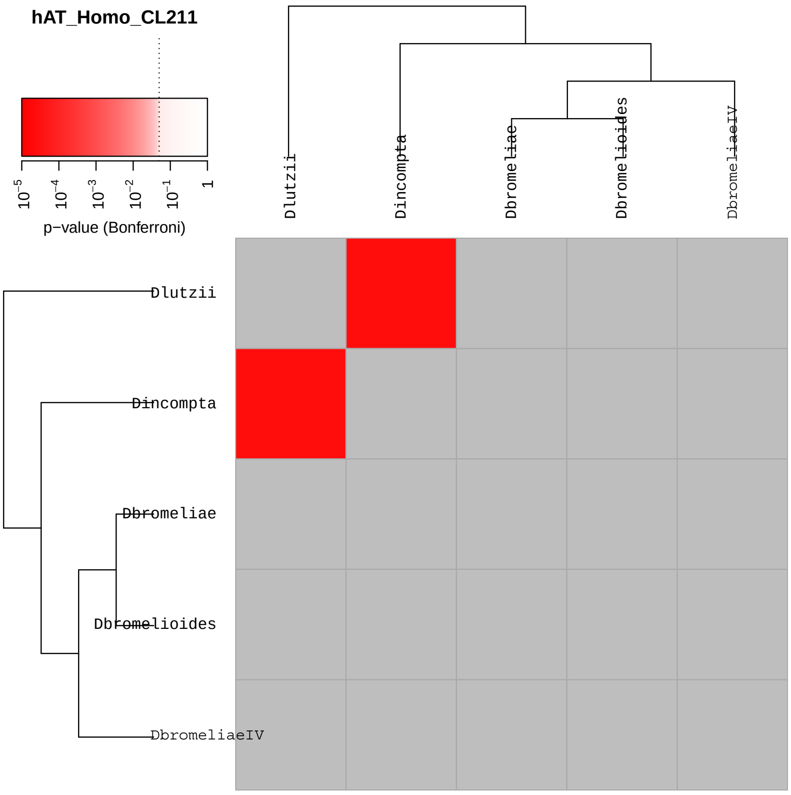
hAT_Homo_CL211
10−5 10−4 10−3 10−2 10−1 1
p−value (Bonferroni)
Dlutzii
Dincompta
Dbromeliae
Dbromelioides
DbromeliaeIV

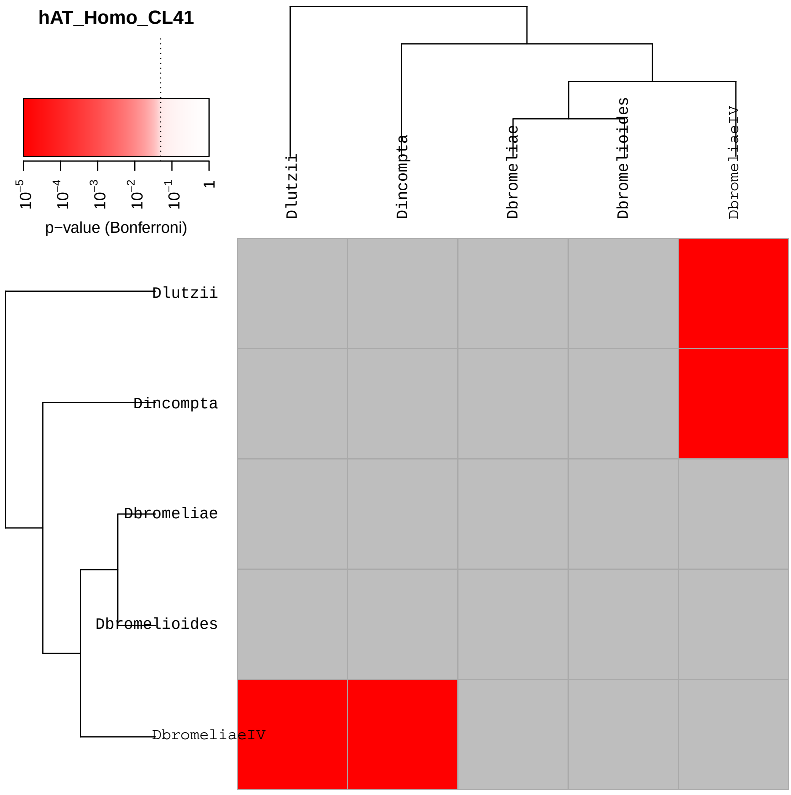
hAT_Homo_CL41
10−5 10−4 10−3 10−2 10−1 1
p−value (Bonferroni)
Dlutzii
Dincompta
Dbromeliae
Dbromelioides
DbromeliaeIV

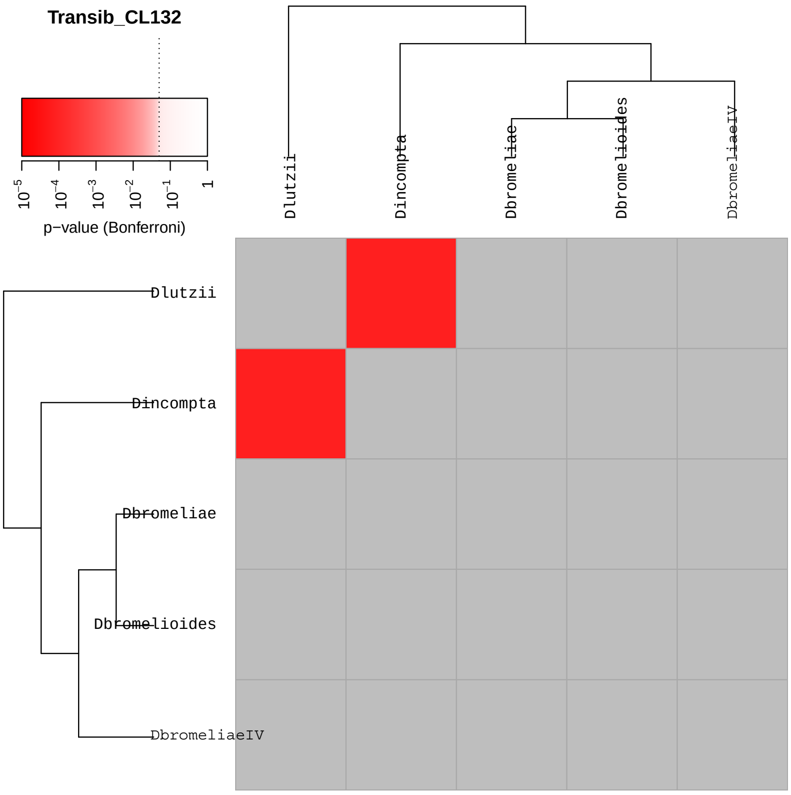
Transib_CL132
10^{-5} 10^{-4} 10^{-3} 10^{-2} 10^{-1} 1
p−value (Bonferroni)
Dlutzii
Dincompta
Dbromeliae
Dbromelioides
DbromeliaeIV

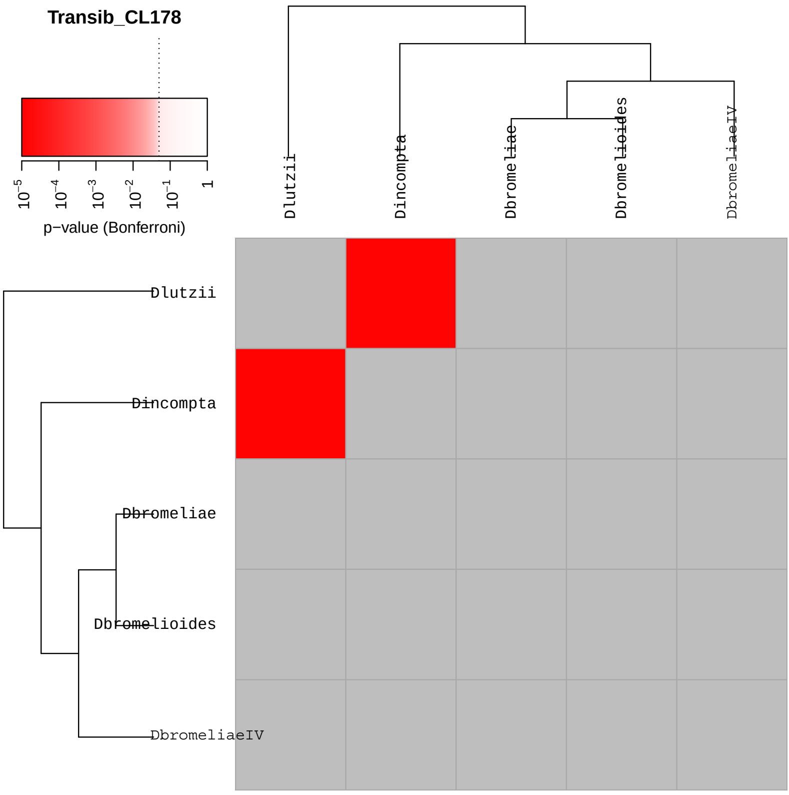
Transib_CL178
10^{-5} 10^{-4} 10^{-3} 10^{-2} 10^{-1} 1
p−value (Bonferroni)
Dlutzii
Dincompta
Dbromeliae
Dbromelioides
DbromeliaeIV

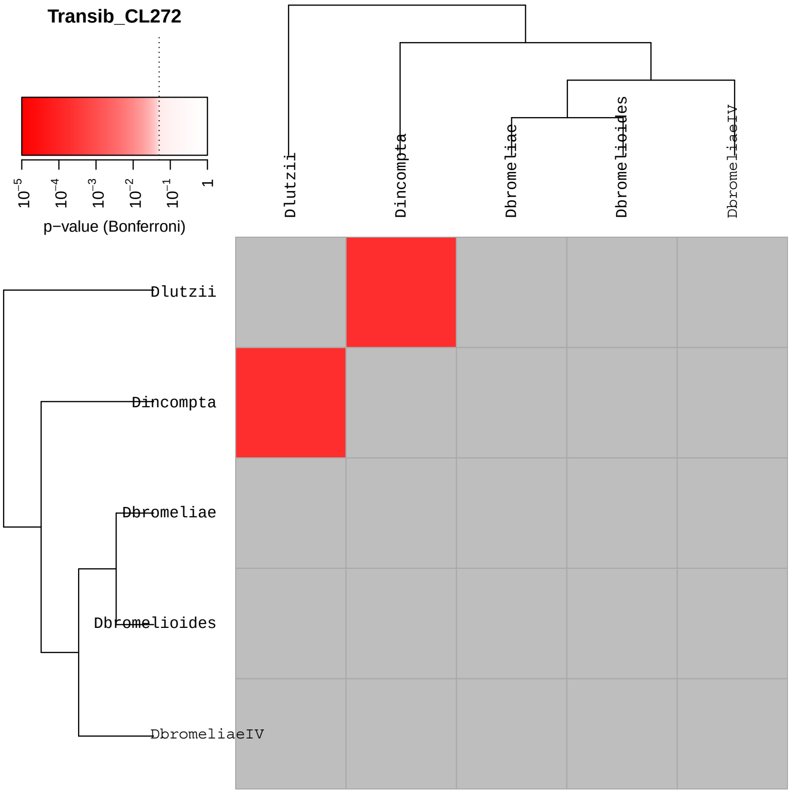
Transib_CL272
10^{-5} 10^{-4} 10^{-3} 10^{-2} 10^{-1} 1
p−value (Bonferroni)
Dlutzii
Dincompta
Dbromeliae
Dbromelioides
DbromeliaeIV

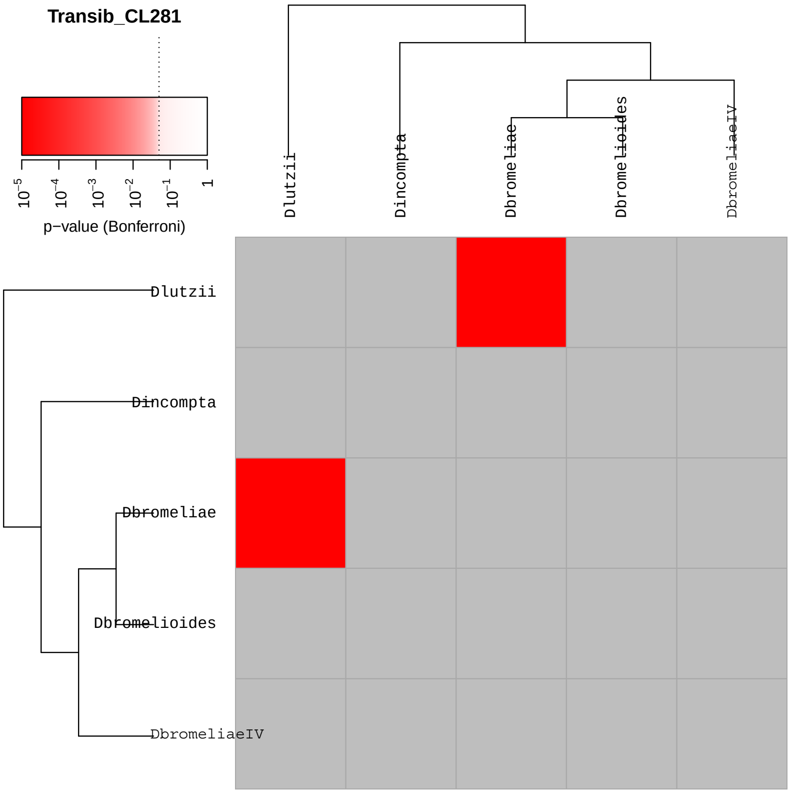
Transib_CL281
10^{-5} 10^{-4} 10^{-3} 10^{-2} 10^{-1} 1
p−value (Bonferroni)
Dlutzii
Dincompta
Dbromeliae
Dbromelioides
DbromeliaeIV

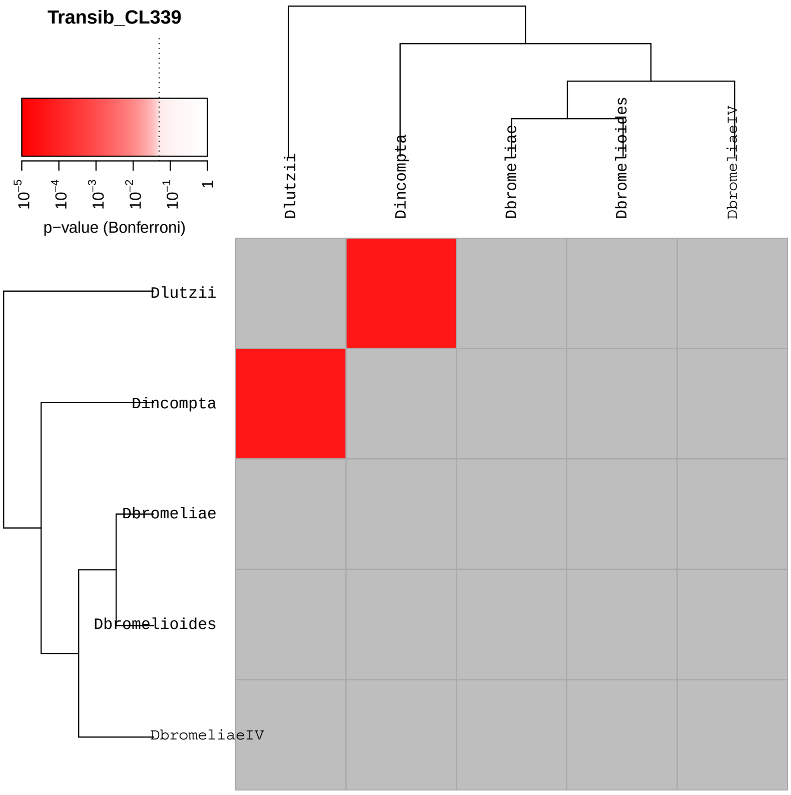
Transsib_CL339
p−value (Bonferroni)
Dlutzii
Dincompta
Dbromeliae
Dbromelioides
DbromeliaeIV

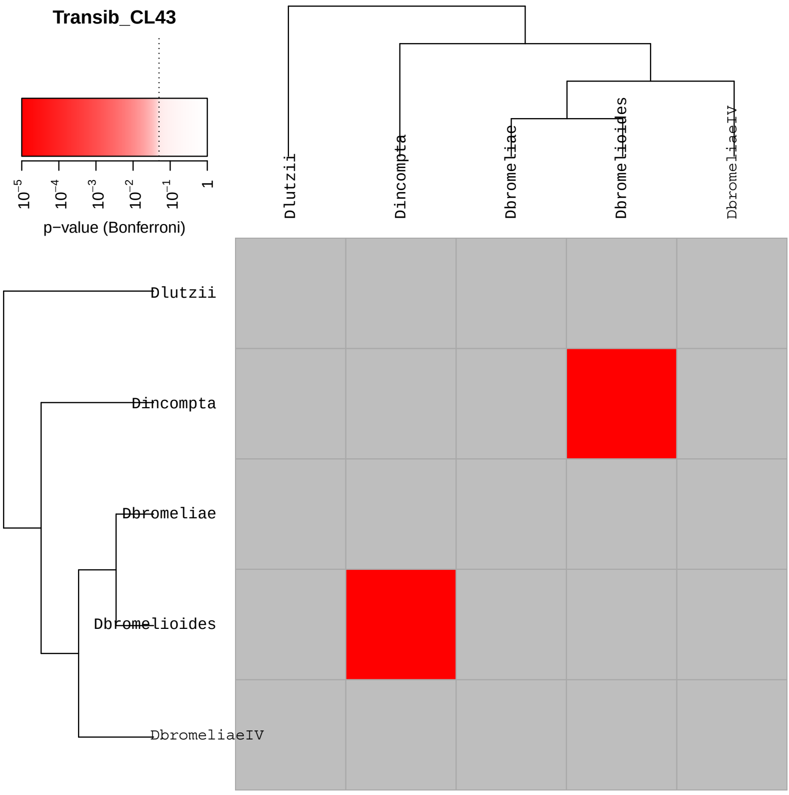
Transib_CL43
p−value (Bonferroni)
Dlutzii
Dincompta
Dbromeliae
Dbromelioides
DbromeliaeIV

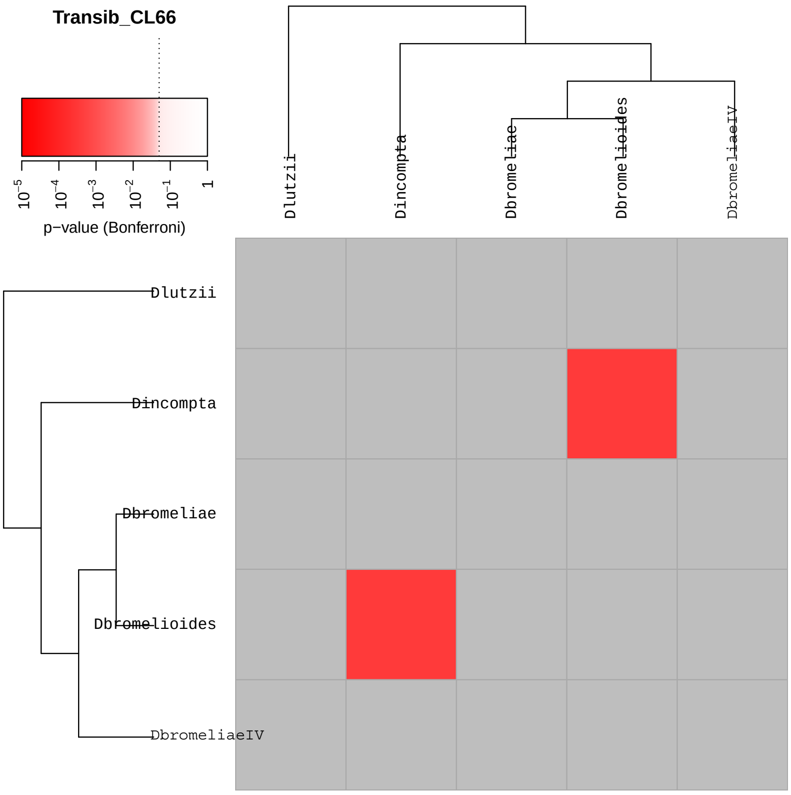
Transib_CL66
p−value (Bonferroni)
Dlutzii
Dincompta
Dbromeliae
Dbromelioides
DbromeliaeIV

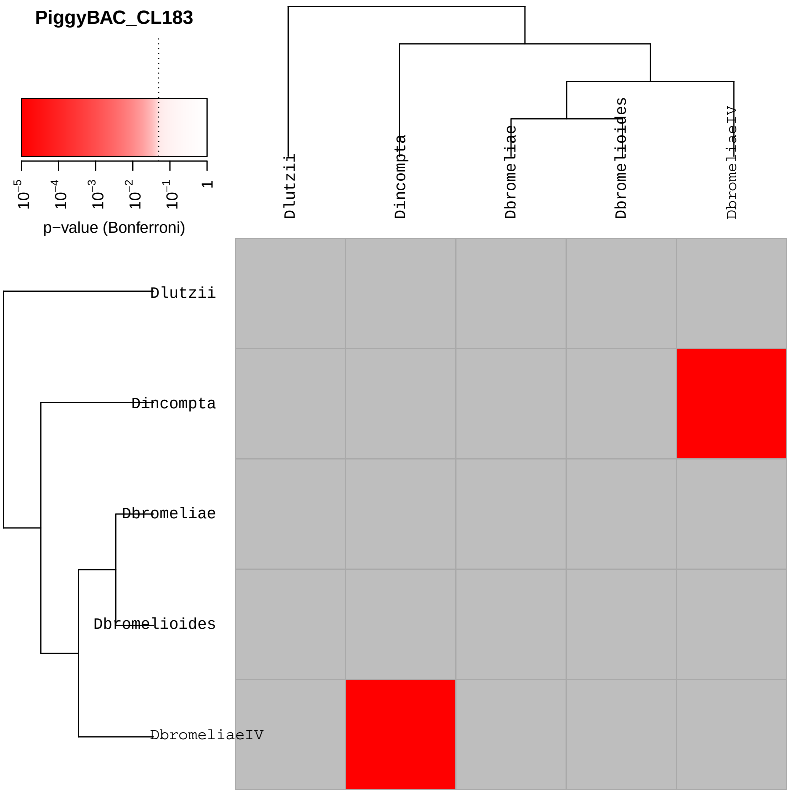
PiggyBAC_CL183
p−value (Bonferroni)
Dlutzii
Dincompta
Dbromeliae
Dbromelioides
DbromeliaeIV

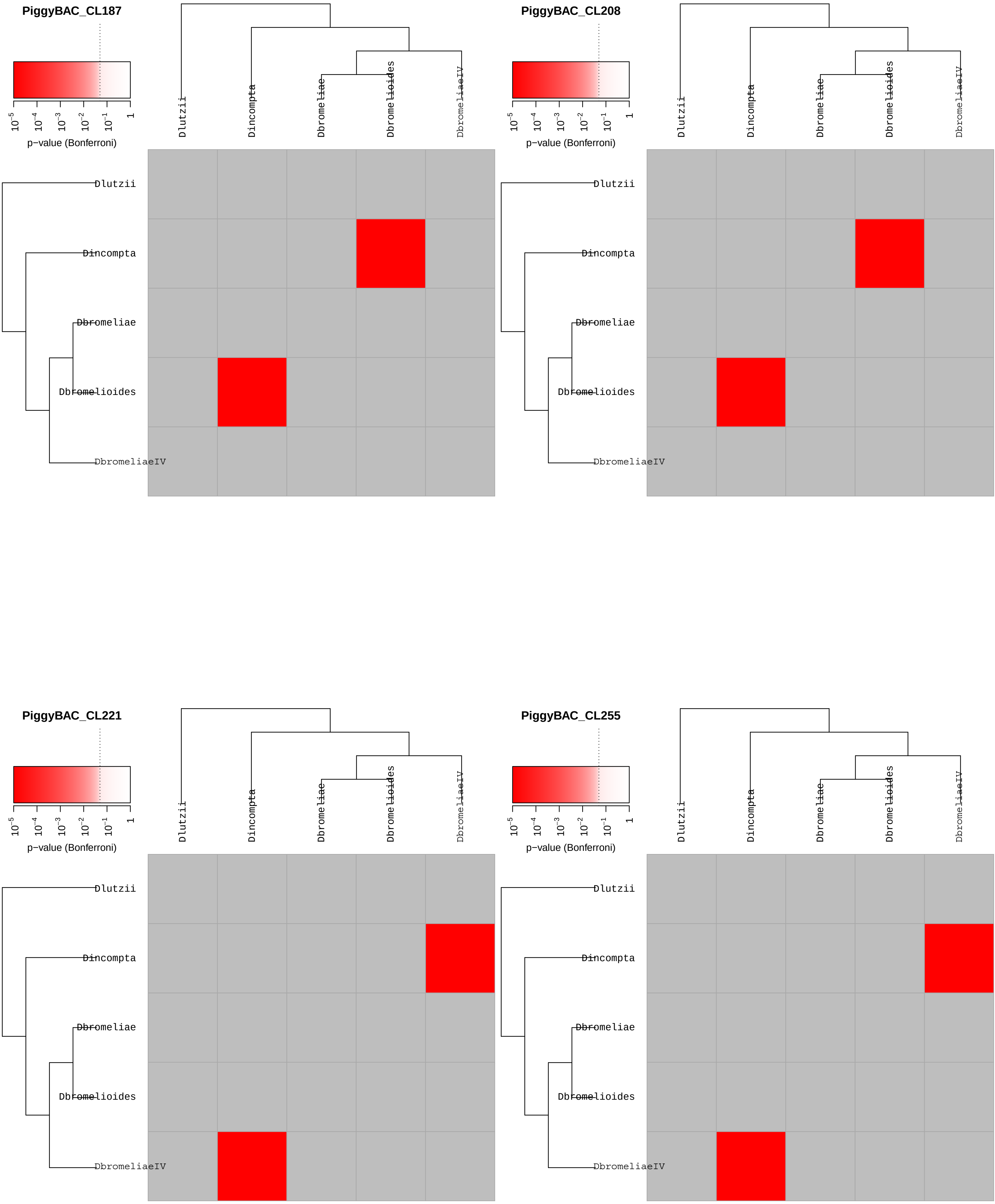
PiggyBAC_CL187
p−value (Bonferroni)
Dlutzii
Dincompta
Dbromeliae
Dbromelioides
DbromeliaeIV
PiggyBAC_CL208
p−value (Bonferroni)
PiggyBAC_CL221
p−value (Bonferroni)
PiggyBAC_CL255
p−value (Bonferroni)

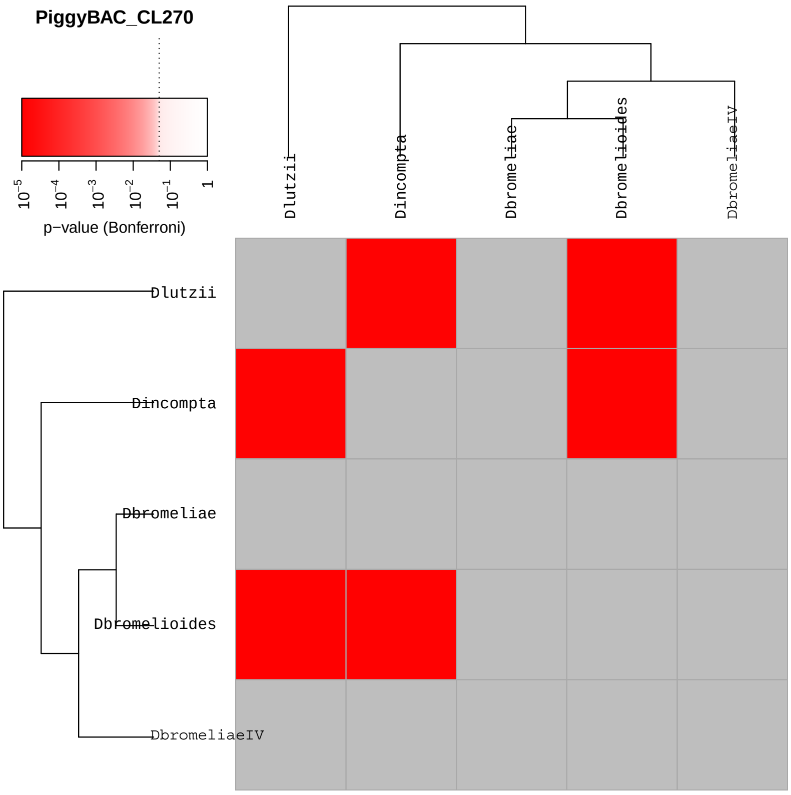
PiggyBAC_CL270
p−value (Bonferroni)
Dlutzii
Dincompta
Dbromeliae
Dbromelioides
DbromeliaeIV

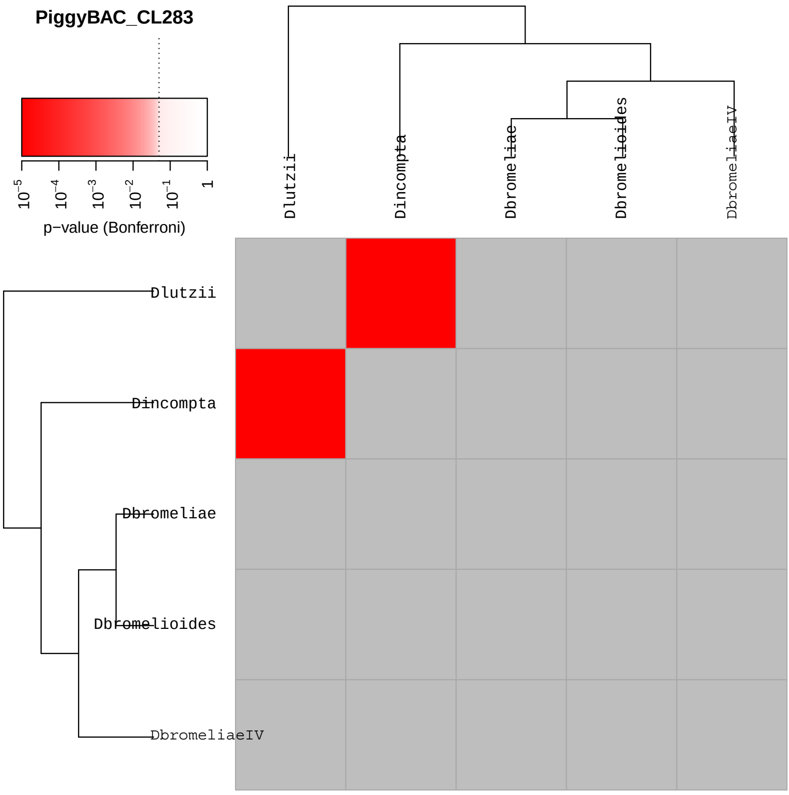
PiggyBAC_CL283
p−value (Bonferroni)
Dlutzii
Dincompta
Dbromeliae
Dbromelioides
DbromeliaeIV

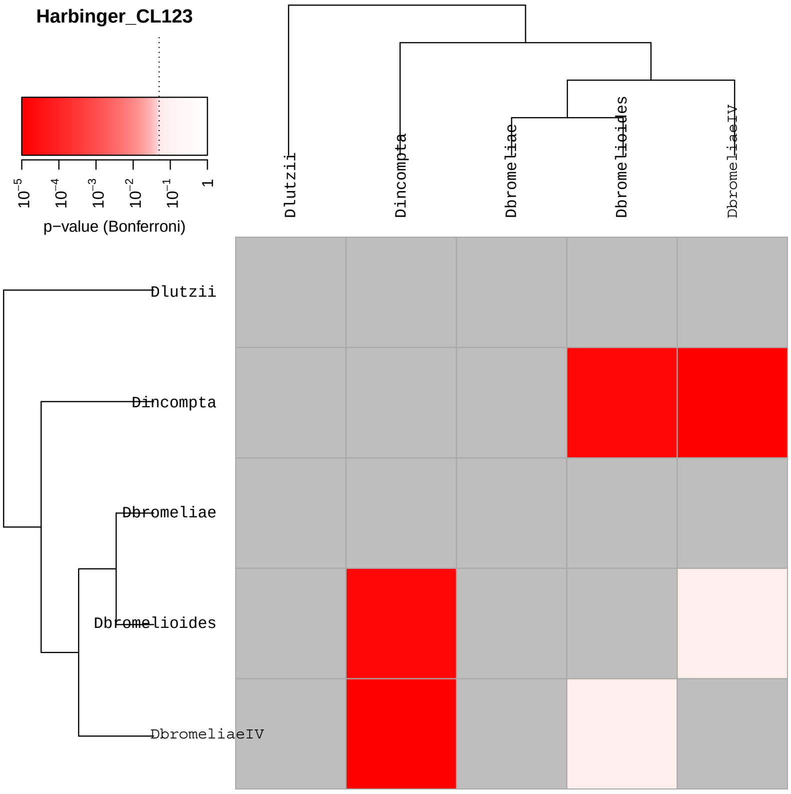
Harbinger_CL123
p−value (Bonferroni)
Dlutzii
Dincompta
Dbromeliae
Dbromelioides
DbromeliaeIV

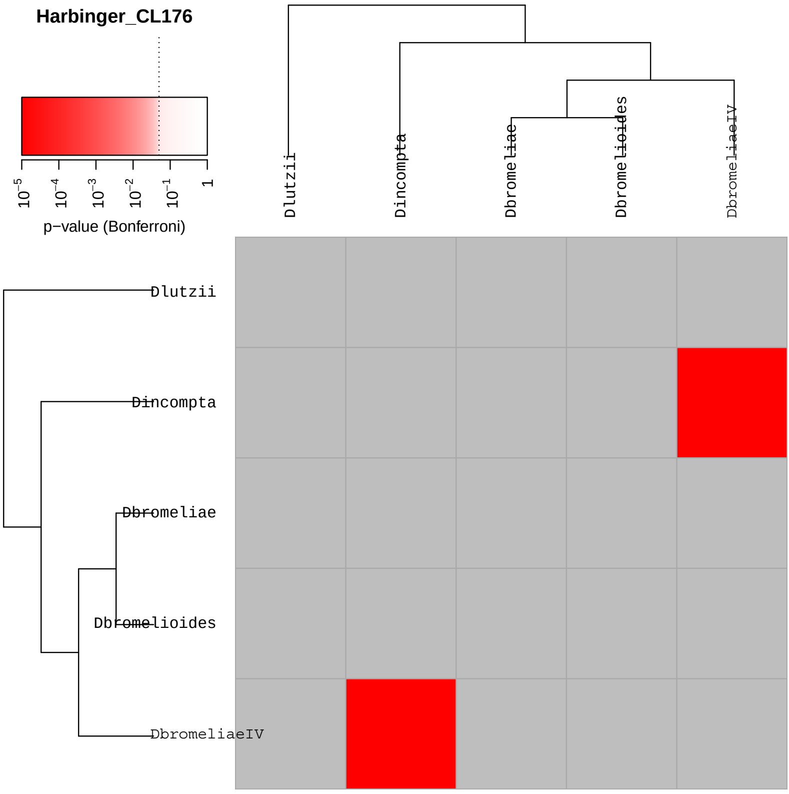
Harbinger_CL176
p−value (Bonferroni)
Dlutzii
Dincompta
Dbromeliae
Dbromelioides
DbromeliaeIV

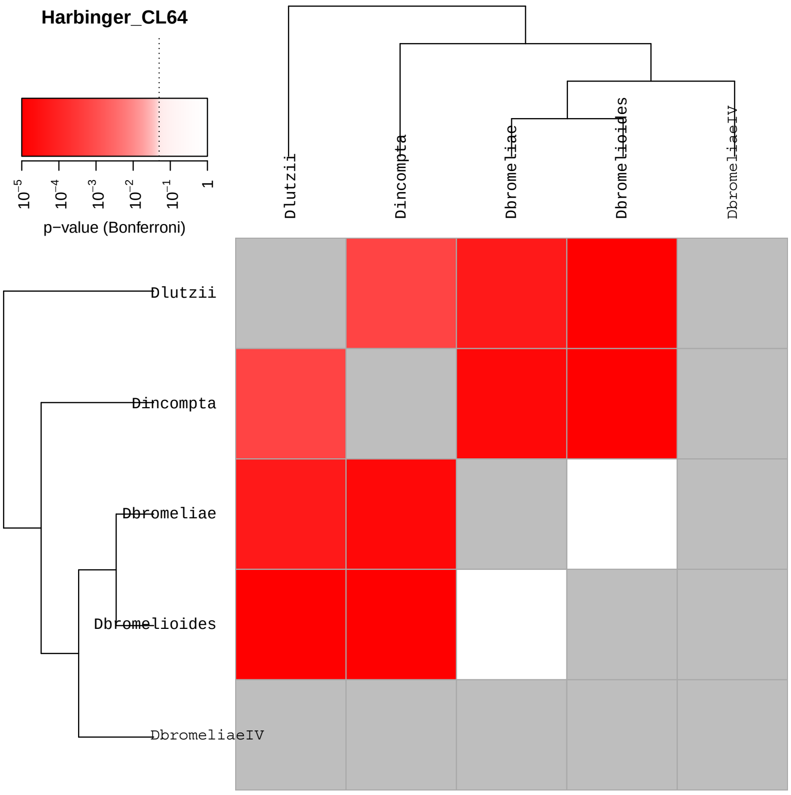
Harbinger_CL64
10^{-5} 10^{-4} 10^{-3} 10^{-2} 10^{-1} 1
p−value (Bonferroni)
Dlutzii
Dincompta
Dbromeliae
Dbromelioides
DbromeliaeIV
Dlutzii
Dincompta
Dbromeliae
Dbromelioides
DbromeliaeIV

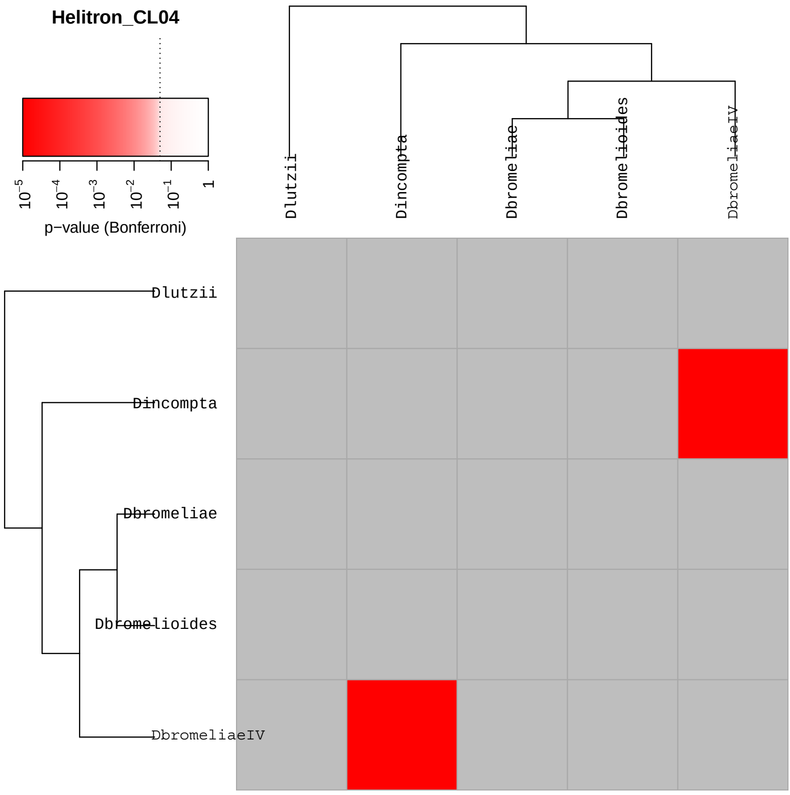
Helitron_CL04
10^{-5} 10^{-4} 10^{-3} 10^{-2} 10^{-1} 1
p−value (Bonferroni)
Dlutzii
Dincompta
Dbromeliae
Dbromelioides
DbromeliaeIV
Dlutzii
Dincompta
Dbromeliae
Dbromelioides
DbromeliaeIV

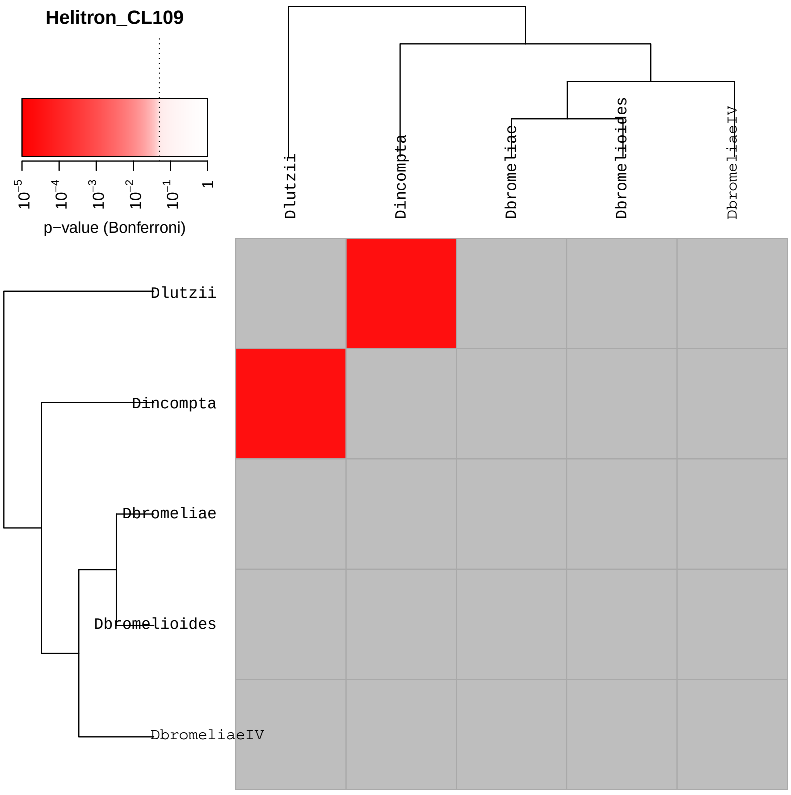
Helitron_CL109
10^{-5} 10^{-4} 10^{-3} 10^{-2} 10^{-1} 1
p−value (Bonferroni)
Dlutzii
Dincompta
Dbromeliae
Dbromelioides
DbromeliaeIV
Dlutzii
Dincompta
Dbromeliae
Dbromelioides
DbromeliaeIV

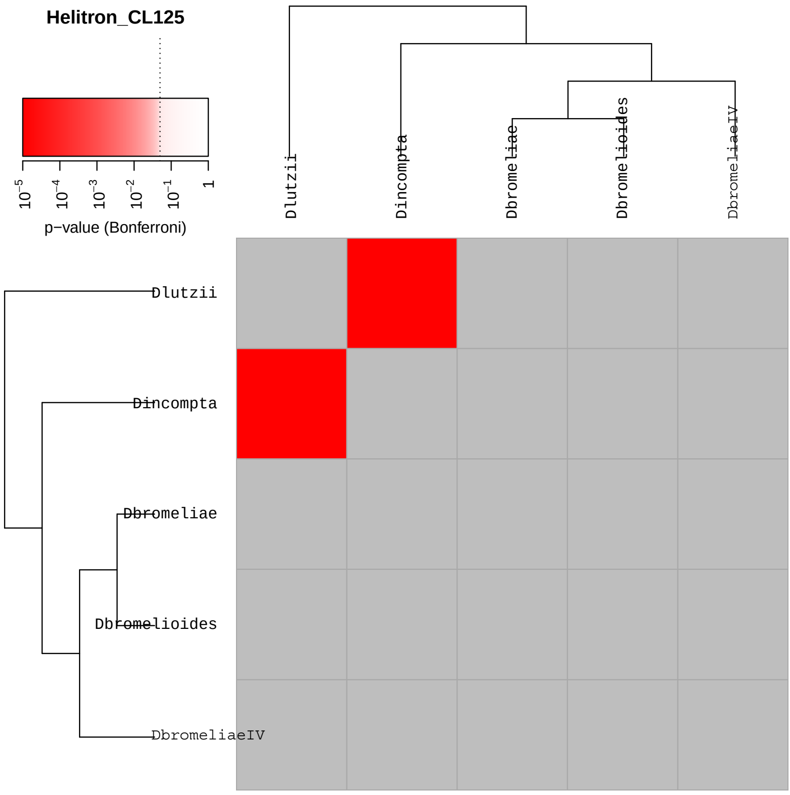
Helitron_CL125
10^{-5} 10^{-4} 10^{-3} 10^{-2} 10^{-1} 1
p−value (Bonferroni)
Dlutzii
Dincompta
Dbromeliae
Dbromelioides
DbromeliaeIV
Dlutzii
Dincompta
Dbromeliae
Dbromelioides
DbromeliaeIV

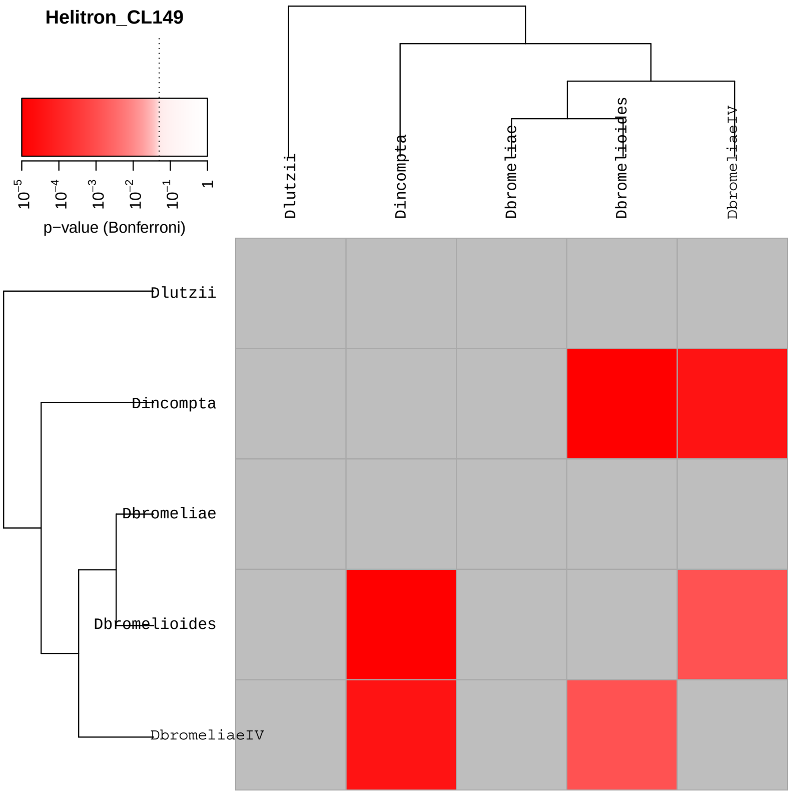
Helitron_CL149
p−value (Bonferroni)
Dlutzii
Dincompta
Dbromeliae
Dbromelioides
DbromeliaeIV

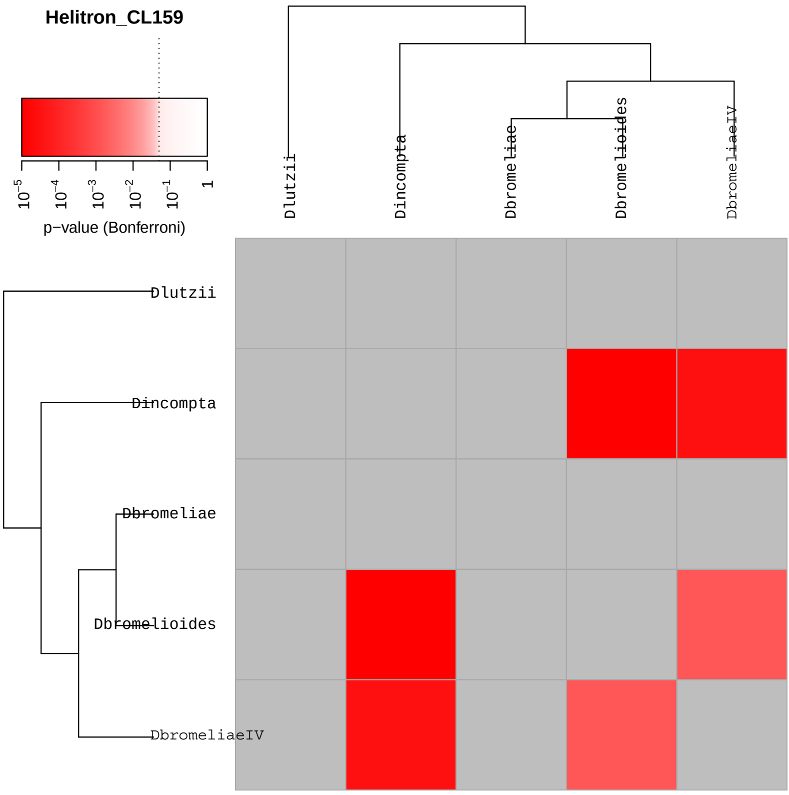
Helitron_CL159
p−value (Bonferroni)
Dlutzii
Dincompta
Dbromeliae
Dbromelioides
DbromeliaeIV

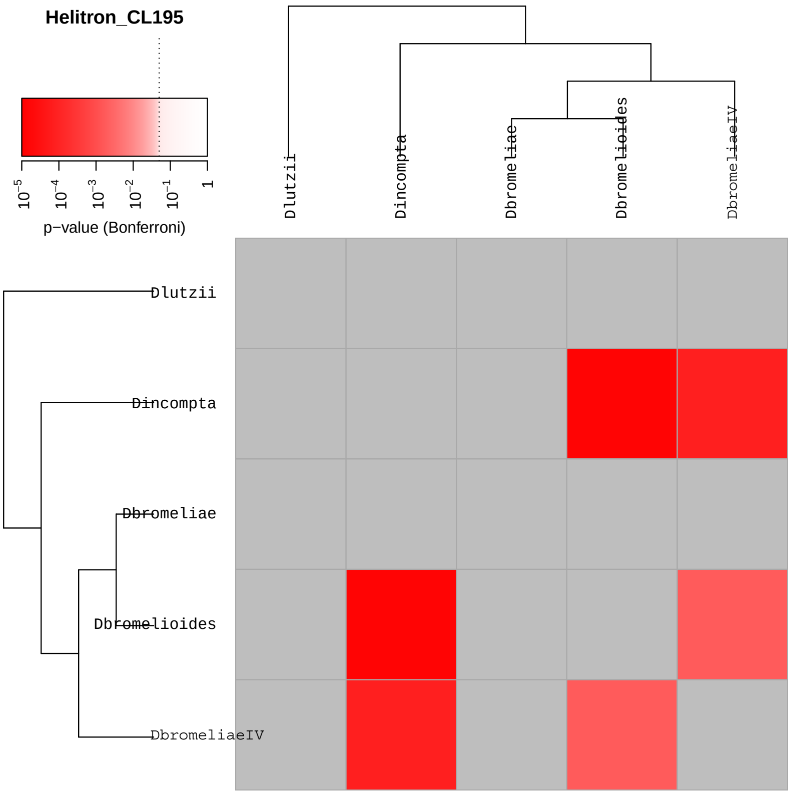
Helitron_CL195
p−value (Bonferroni)
Dlutzii
Dincompta
Dbromeliae
Dbromelioides
DbromeliaeIV

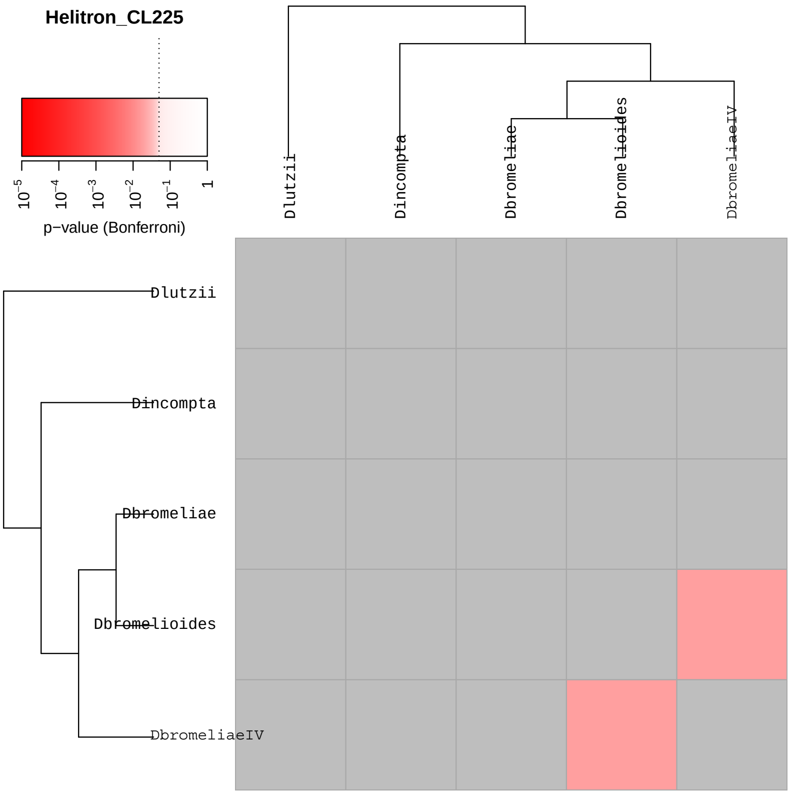
Helitron_CL225
p−value (Bonferroni)
Dlutzii
Dincompta
Dbromeliae
Dbromelioides
DbromeliaeIV

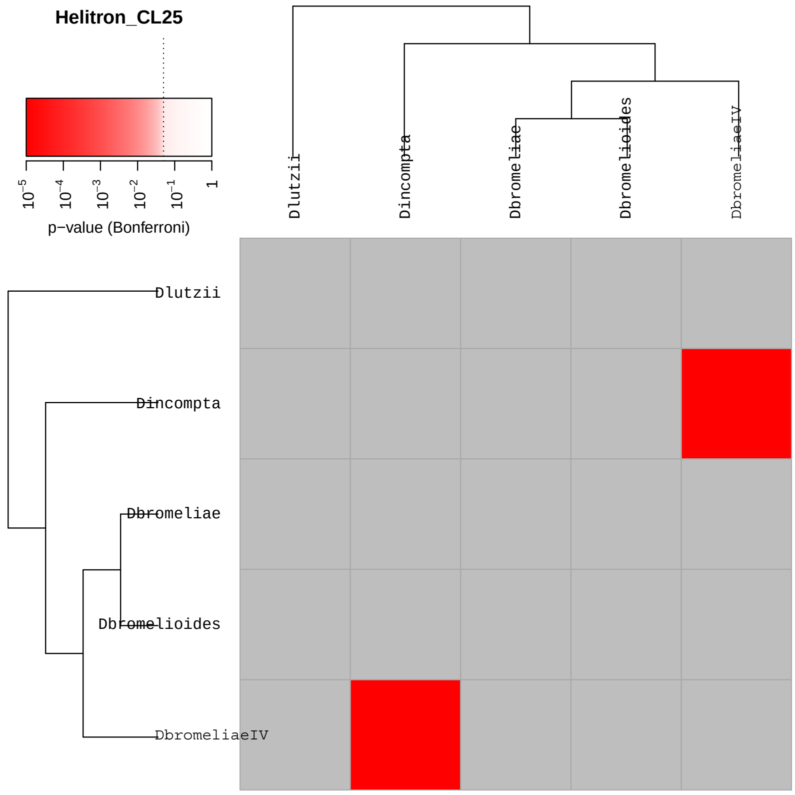
Helitron_CL25
10^{-5} 10^{-4} 10^{-3} 10^{-2} 10^{-1} 1
p−value (Bonferroni)
Dlutzii
Dincompta
Dbromeliae
Dbromelioides
DbromeliaeIV

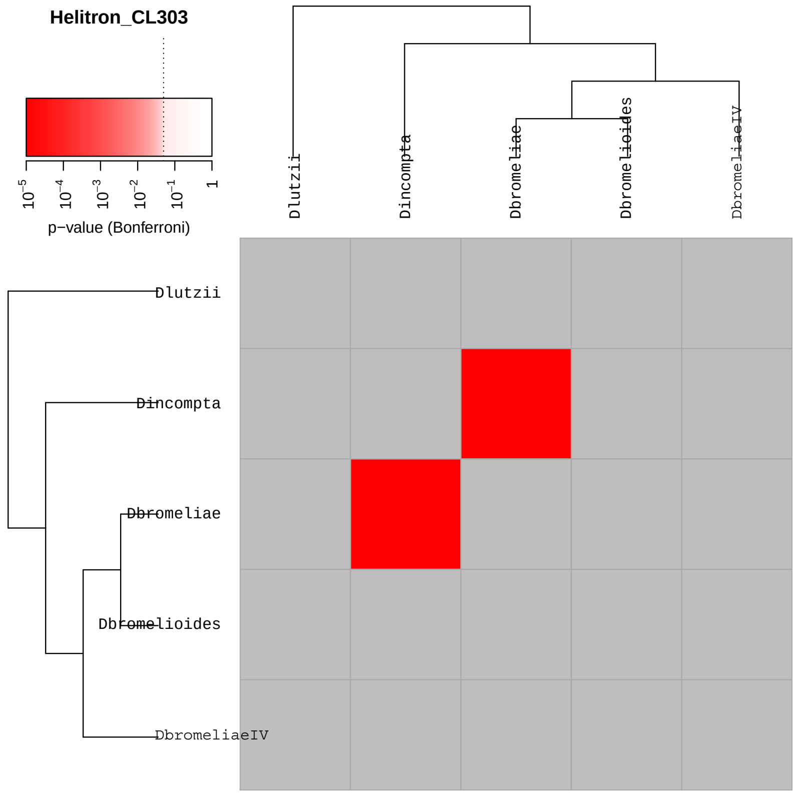
Helitron_CL303
10^{-5} 10^{-4} 10^{-3} 10^{-2} 10^{-1} 1
p−value (Bonferroni)
Dlutzii
Dincompta
Dbromeliae
Dbromelioides
DbromeliaeIV

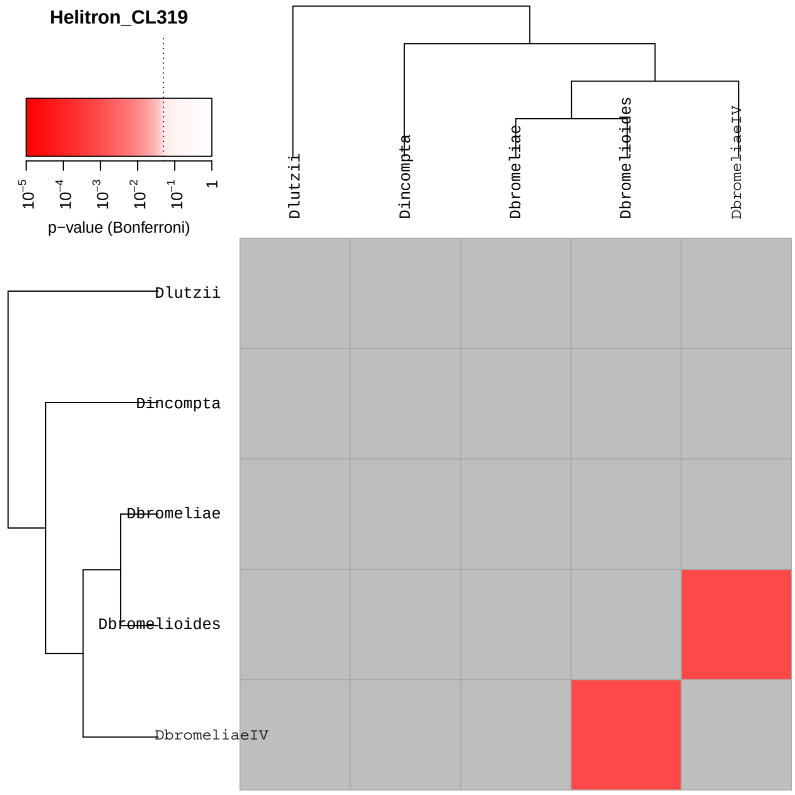
Helitron_CL319
10^{-5} 10^{-4} 10^{-3} 10^{-2} 10^{-1} 1
p−value (Bonferroni)
Dlutzii
Dincompta
Dbromeliae
Dbromelioides
DbromeliaeIV

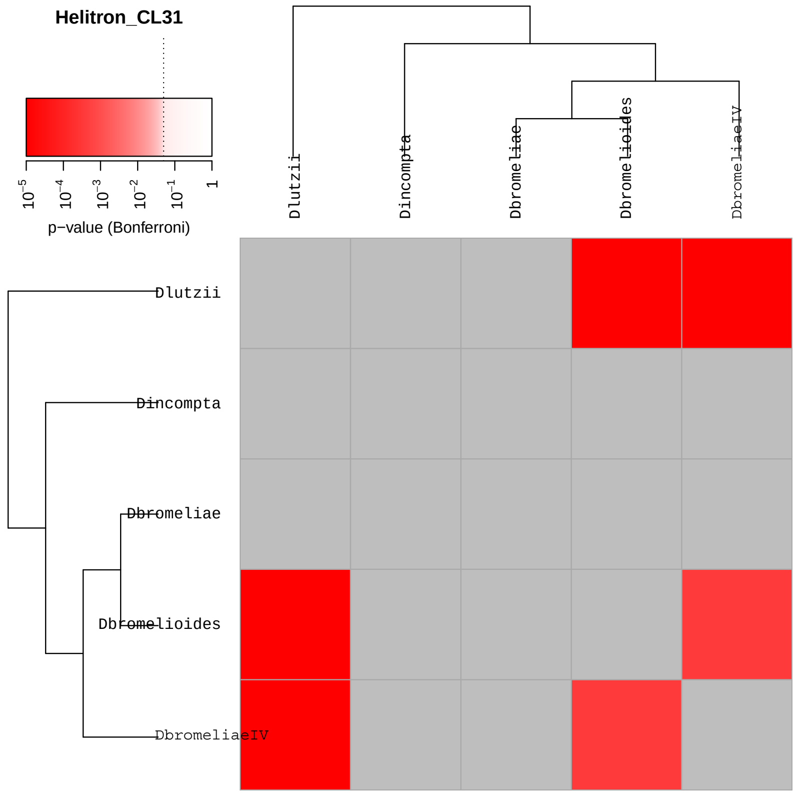
Helitron_CL31
10^{-5} 10^{-4} 10^{-3} 10^{-2} 10^{-1} 1
p−value (Bonferroni)
Dlutzii
Dincompta
Dbromeliae
Dbromelioides
DbromeliaeIV

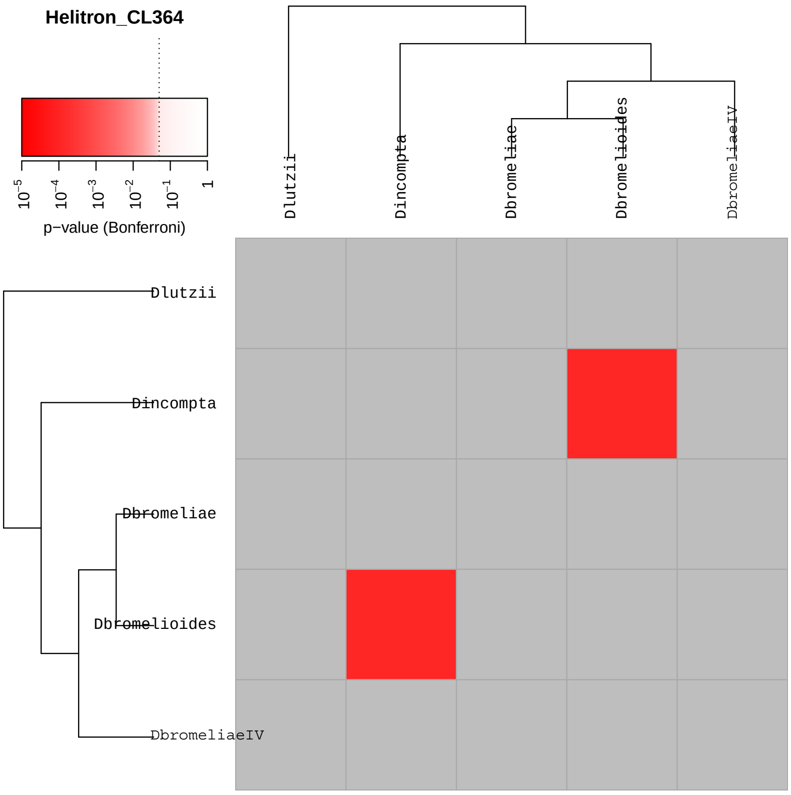

Helitron_CL364
p−value (Bonferroni)
Dlutzii
Dincompta
Dbromeliae
Dbromelioides
DbromeliaeIV

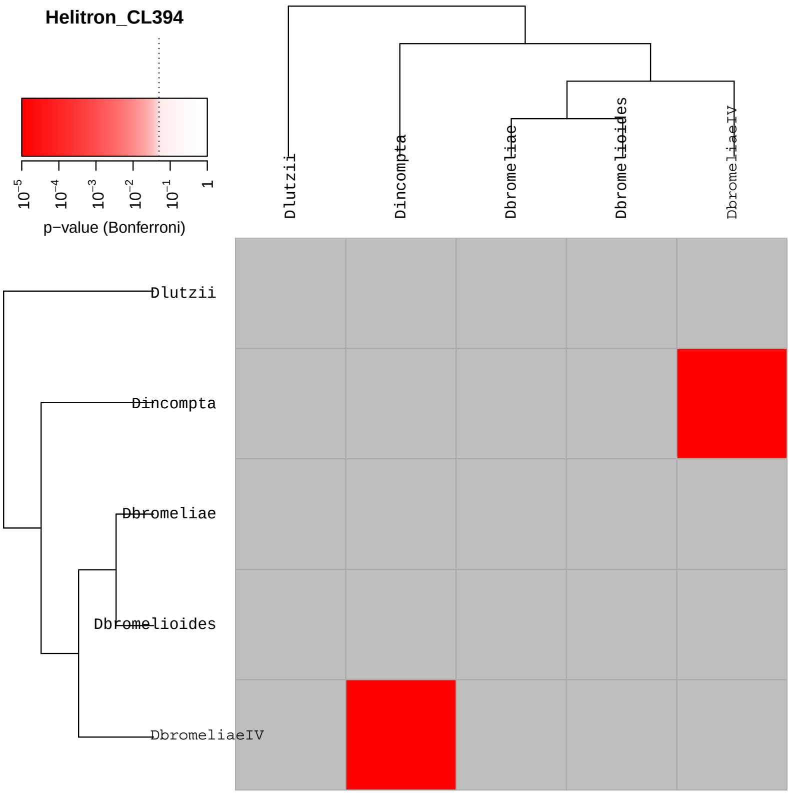

Helitron_CL394
p−value (Bonferroni)
Dlutzii
Dincompta
Dbromeliae
Dbromelioides
DbromeliaeIV

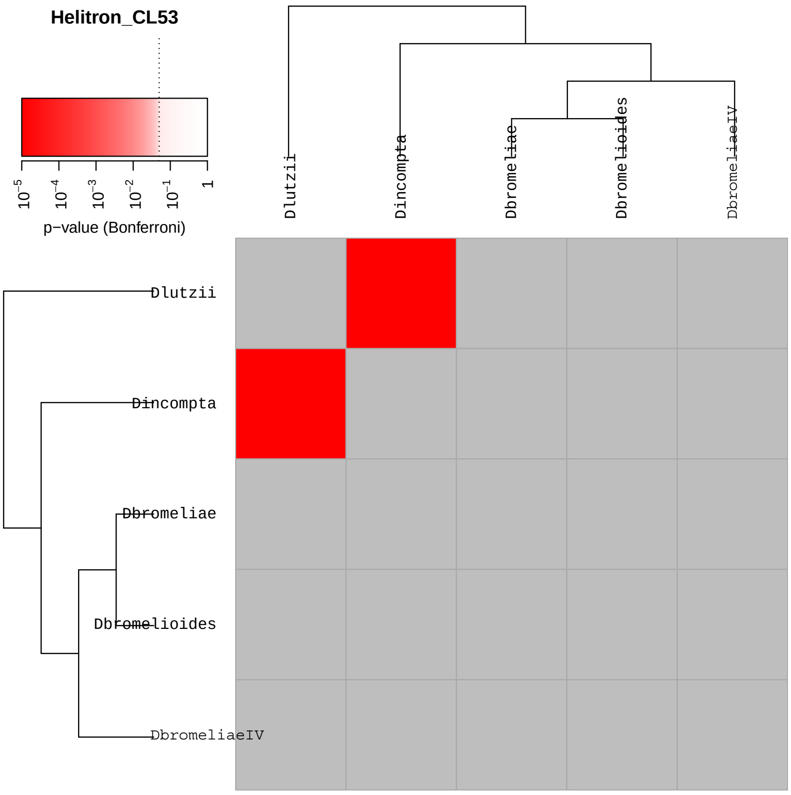

Helitron_CL53
p−value (Bonferroni)
Dlutzii
Dincompta
Dbromeliae
Dbromelioides
DbromeliaeIV

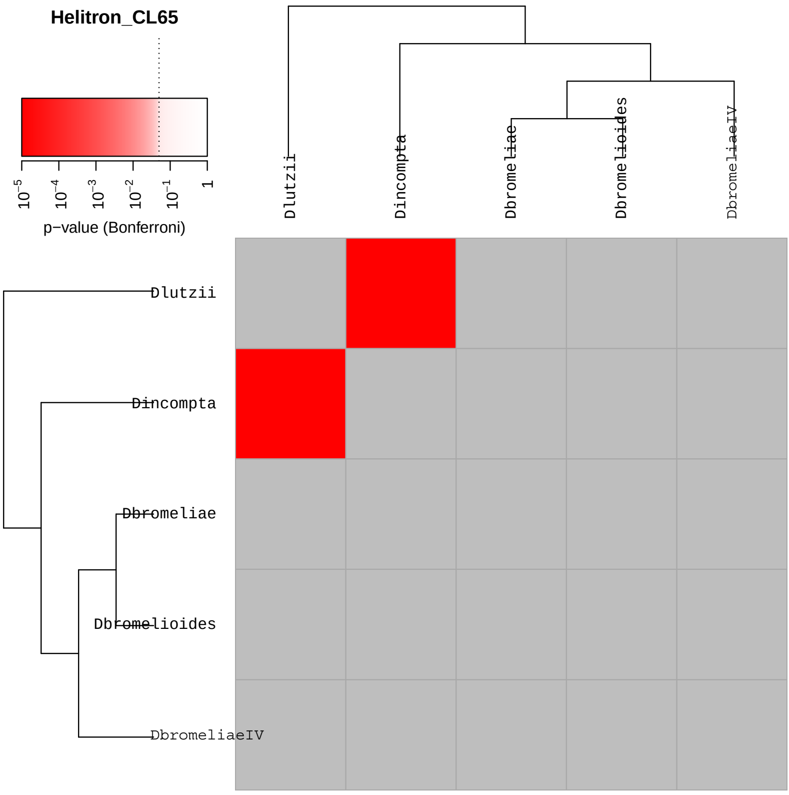

Helitron_CL65
p−value (Bonferroni)
Dlutzii
Dincompta
Dbromeliae
Dbromelioides
DbromeliaeIV

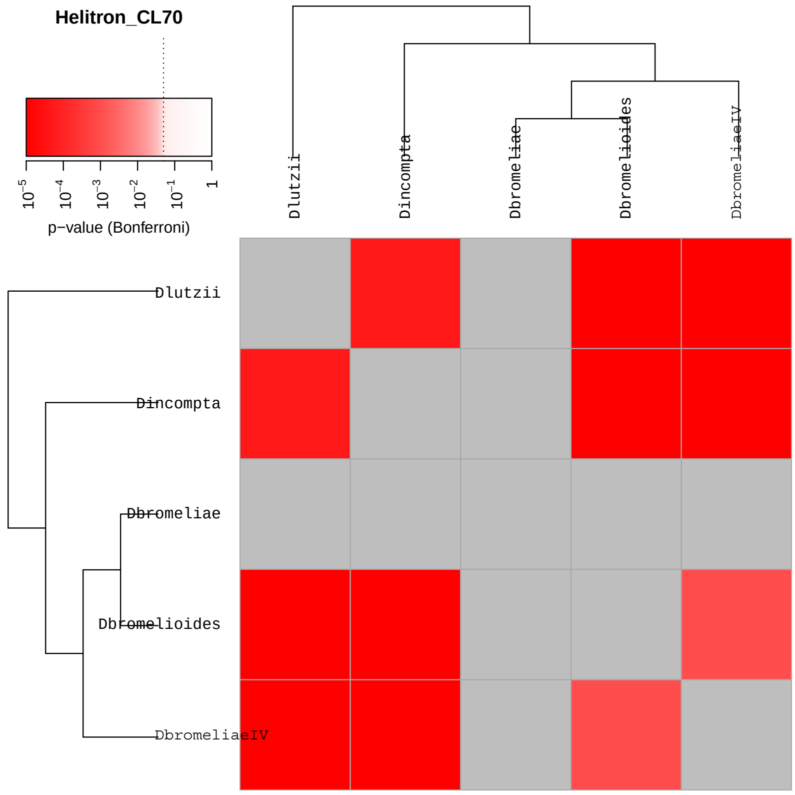
Helitron_CL70
10^{-5} 10^{-4} 10^{-3} 10^{-2} 10^{-1} 1
p−value (Bonferroni)
Dlutzii
Dincompta
Dbromeliae
Dbromelioides
DbromeliaeIV

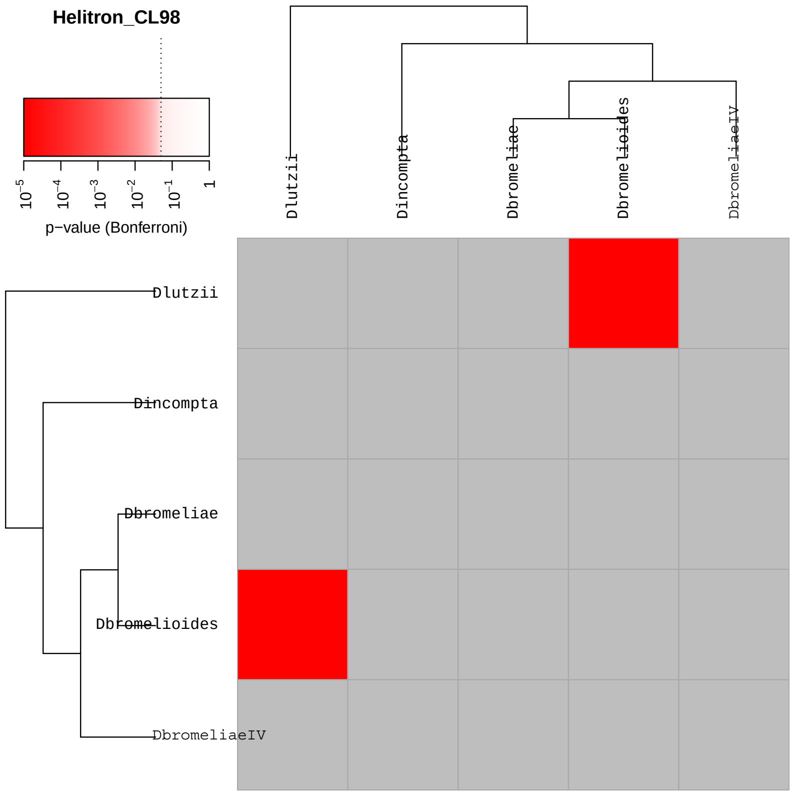
Helitron_CL98
10^{-5} 10^{-4} 10^{-3} 10^{-2} 10^{-1} 1
p−value (Bonferroni)
Dlutzii
Dincompta
Dbromeliae
Dbromelioides
DbromeliaeIV

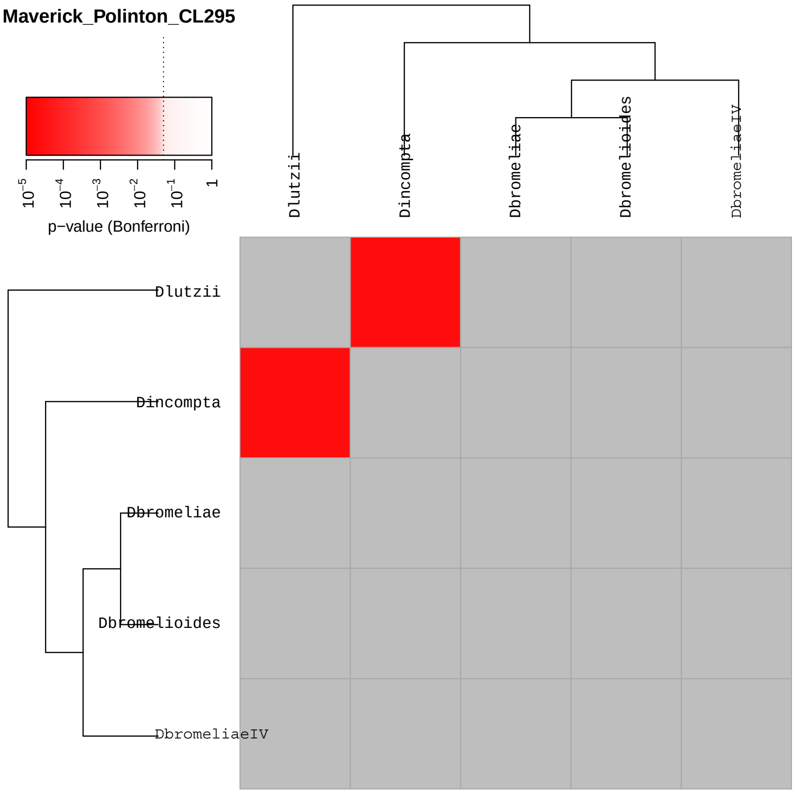
Maverick_Polinton_CL295
10^{-5} 10^{-4} 10^{-3} 10^{-2} 10^{-1} 1
p−value (Bonferroni)
Dlutzii
Dincompta
Dbromeliae
Dbromelioides
DbromeliaeIV

File S2. Results of Generalized Linear Mixed Models performed with the lme4 package of R to infer the best biological model explaining the potential for HTTs among the five FBD species.

```
> library("lme4")
> require ("car")
> data <- read.table("table_S8", header=T, dec=",")
> data

> model <-
lmer(logit(effect)~abiotic_niche_overlap+geographic_overlap+biotic_niche_overlap+log10(niche_breadth)+phylogenetic_signal+(1|acting)+(1|target), data=data, REML=F)
> summary (model)
Linear mixed model fit by maximum likelihood  ['lmerMod']
Formula: logit(effect) ~ abiotic_niche_overlap + biotic_niche_overlap +
    geographic_overlap + log10(niche_breadth) + phylogenetic_signal +
    (1 | acting) + (1 | target)
   Data: data

     AIC      BIC   logLik deviance df.resid
    11.4     20.3      3.3     -6.6       11

Scaled residuals:
    Min      1Q  Median      3Q     Max
-1.8783 -0.4212  0.2728  0.5376  1.0277

Random effects:
 Groups   Name        Variance Std.Dev.
 acting   (Intercept) 0.533533 0.73043
 target   (Intercept) 0.014239 0.11933
 Residual             0.006181 0.07862
Number of obs: 20, groups:  acting, 5; target, 5
```

```
Fixed effects:
                    Estimate Std. Error t value
(Intercept)          -2.102435   0.919200  -2.287
abiotic_niche_overlap  1.777662   0.512231   3.470
biotic_niche_overlap  -1.815615   0.557298  -3.258
geographic_overlap    -0.047633   0.111580  -0.427
log10(niche_breadth)   0.925196   0.917286   1.009
phylogenetic_signal    0.001842   0.004726   0.390

Correlation of Fixed Effects:
            (Intr) abtc__ btc_n_ ggrph_ l10(_)
abtc_nch_vr -0.470
btc_nch_vrl  0.017 -0.141
ggrphc_vrlp -0.157  0.152  0.170
lg10(nch_b)  0.793  0.014 -0.052 -0.043
phylgntc_sg -0.360  0.651 -0.241 -0.017  0.012

> require ("MuMIn")
> options(na.action = "na.fail")
> model_combinations<-dredge(model)
> model_selection<-model.avg(model_combinations, beta='partial.sd')
> summary(model_selection)

Call:
model.avg(object = get.models(object = model_combinations, subset = NA),
   beta = "partial.sd")

Component model call:
lmer(formula = logit(effect) ~ <32 unique rhs>, data = data, REML = F)
```

Component models:

	df	logLik	AICc	delta	weight
1	5	1.61	11.06	0.00	0.41
12	6	2.72	13.02	1.95	0.15
(Null)	4	-1.66	13.98	2.91	0.09
14	6	1.94	14.59	3.53	0.07
15	6	1.63	15.20	4.14	0.05
13	6	1.61	15.23	4.17	0.05
5	5	-1.12	16.53	5.46	0.03
4	5	-1.33	16.94	5.88	0.02
124	7	3.17	17.00	5.93	0.02
2	5	-1.49	17.27	6.21	0.02
3	5	-1.64	17.56	6.50	0.02
123	7	2.80	17.73	6.66	0.01
125	7	2.78	17.78	6.72	0.01
145	7	1.95	19.43	8.36	0.01
134	7	1.94	19.45	8.39	0.01
45	6	-0.80	20.05	8.99	0.00
135	7	1.63	20.07	9.01	0.00
25	6	-1.01	20.48	9.41	0.00
35	6	-1.11	20.69	9.62	0.00
24	6	-1.14	20.74	9.68	0.00
34	6	-1.30	21.07	10.01	0.00
23	6	-1.45	21.37	10.31	0.00
1234	8	3.26	22.57	11.51	0.00
1245	8	3.23	22.63	11.57	0.00
1235	8	2.86	23.38	12.31	0.00
245	7	-0.65	24.62	13.56	0.00
345	7	-0.78	24.90	13.84	0.00

```
1345    8   1.96 25.18 14.11   0.00
235     7  -0.96 25.25 14.19   0.00
234     7  -1.09 25.50 14.44   0.00
12345   9   3.32 29.36 18.30   0.00
2345    8  -0.56 30.21 19.15   0.00
```

Term codes:

```
abiotic_niche_overlap  biotic_niche_overlap    geographic_overlap   log10(niche_breadth)
                1                    2                    3                    4
  phylogenetic_signal
                5
```

Model-averaged coefficients:

(full average)

```
                        Estimate Std. Error Adjusted SE z value Pr(>|z|)
(Intercept)            0.0000000  0.0000000   0.0000000     NaN      NaN
abiotic_niche_overlap  0.0906355  0.0628299   0.0643018   1.410    0.159
biotic_niche_overlap  -0.0175150  0.0357249   0.0362451   0.483    0.629
log10(niche_breadth)   0.0371449  0.1480374   0.1568025   0.237    0.813
phylogenetic_signal   -0.0017525  0.0153338   0.0163523   0.107    0.915
geographic_overlap    -0.0008781  0.0191063   0.0208641   0.042    0.966
```

(conditional average)

```
                       Estimate Std. Error Adjusted SE z value Pr(>|z|)
(Intercept)            0.000000   0.000000    0.000000     NaN      NaN
abiotic_niche_overlap  0.113031   0.048905    0.051235   2.206   0.0274 *
biotic_niche_overlap  -0.074586   0.034325    0.036573   2.039   0.0414 *
log10(niche_breadth)   0.266719   0.310047    0.339581   0.785   0.4322
phylogenetic_signal   -0.014865   0.042420    0.045533   0.326   0.7441
geographic_overlap    -0.008487   0.058853    0.064364   0.132   0.8951
```

```
---
Signif. codes:  0 ‘***’ 0.001 ‘**’ 0.01 ‘*’ 0.05 ‘.’ 0.1 ‘ ’ 1

> best_model <-
lmer(logit(effect)~abiotic_niche_overlap+geographic_overlap+biotic_niche_overlap+lo
g10(niche_breadth)+phylogenetic_signal+(1|acting)+(1|target), data=data, REML=F)

> summary(best_model)
Linear mixed model fit by maximum likelihood  ['lmerMod']
Formula: logit(effect) ~ abiotic_niche_overlap + biotic_niche_overlap +
    log10(niche_breadth) + geographic_overlap + phylogenetic_signal +
    (1 | acting) + (1 | target)
   Data: data

     AIC      BIC   logLik deviance df.resid
    11.4     20.3      3.3     -6.6       11

Scaled residuals:
    Min      1Q  Median      3Q     Max
-1.8783 -0.4212  0.2728  0.5376  1.0277

Random effects:
 Groups   Name        Variance Std.Dev.
 acting   (Intercept) 0.533533 0.73043
 target   (Intercept) 0.014239 0.11933
 Residual             0.006181 0.07862
Number of obs: 20, groups:  acting, 5; target, 5

Fixed effects:
                       Estimate Std. Error t value
(Intercept)           -2.102435   0.919200  -2.287
```

```
abiotic_niche_overlap  1.777662   0.512231   3.470
biotic_niche_overlap  -1.815615   0.557298  -3.258
log10(niche_breadth)   0.925196   0.917286   1.009
geographic_overlap    -0.047633   0.111580  -0.427
phylogenetic_signal    0.001842   0.004726   0.390

Correlation of Fixed Effects:
            (Intr) abtc__ btc_n_ l10(_) ggrph_
abtc_nch_vr -0.470
btc_nch_vrl  0.017 -0.141
lg10(nch_b)  0.793  0.014 -0.052
ggrphc_vrlp -0.157  0.152  0.170 -0.043
phylgntc_sg -0.360  0.651 -0.241  0.012 -0.017

>Anova(best_model)
Analysis of Deviance Table (Type II Wald chisquare tests)

Response: logit(effect)
                        Chisq Df Pr(>Chisq)
abiotic_niche_overlap 12.0439  1  0.0005196 ***
biotic_niche_overlap  10.6138  1  0.0011225 **
log10(niche_breadth)   1.0173  1  0.3131553
geographic_overlap     0.1822  1  0.6694566
phylogenetic_signal    0.1519  1  0.6966890
---
Signif. codes:  0 ‘***’ 0.001 ‘**’ 0.01 ‘*’ 0.05 ‘.’ 0.1 ‘ ’ 1
```
